# Projecting social contact matrices in 152 countries using contact surveys and demographic data

**DOI:** 10.1371/journal.pcbi.1005697

**Published:** 2017-09-12

**Authors:** Kiesha Prem, Alex R. Cook, Mark Jit

**Affiliations:** 1 Saw Swee Hock School of Public Health, National University of Singapore and National University Health System, Singapore, Singapore; 2 Program in Health Services and Systems Research, Duke-NUS Graduate Medical School, Singapore, Singapore; 3 Department of Statistics and Applied Probability, National University of Singapore, Singapore, Singapore; 4 Department of Infectious Disease Epidemiology, London School of Hygiene & Tropical Medicine, London, United Kingdom; 5 Modelling and Economics Unit, Health Protection Agency Centre for Infections, London, United Kingdom; Emory University, UNITED STATES

## Abstract

Heterogeneities in contact networks have a major effect in determining whether a pathogen can become epidemic or persist at endemic levels. Epidemic models that determine which interventions can successfully prevent an outbreak need to account for social structure and mixing patterns. Contact patterns vary across age and locations (e.g. home, work, and school), and including them as predictors in transmission dynamic models of pathogens that spread socially will improve the models’ realism. Data from population-based contact diaries in eight European countries from the POLYMOD study were projected to 144 other countries using a Bayesian hierarchical model that estimated the proclivity of age-and-location-specific contact patterns for the countries, using Markov chain Monte Carlo simulation. Household level data from the Demographic and Health Surveys for nine lower-income countries and socio-demographic factors from several on-line databases for 152 countries were used to quantify similarity of countries to estimate contact patterns in the home, work, school and other locations for countries for which no contact data are available, accounting for demographic structure, household structure where known, and a variety of metrics including workforce participation and school enrolment. Contacts are highly assortative with age across all countries considered, but pronounced regional differences in the age-specific contacts at home were noticeable, with more inter-generational contacts in Asian countries than in other settings. Moreover, there were variations in contact patterns by location, with work-place contacts being least assortative. These variations led to differences in the effect of social distancing measures in an age structured epidemic model. Contacts have an important role in transmission dynamic models that use contact rates to characterize the spread of contact-transmissible diseases. This study provides estimates of mixing patterns for societies for which contact data such as POLYMOD are not yet available.

## Author summary

The risk of infectious disease transmission varies in different settings, for instance at home, at work or in the community, as a result of the different social structures and mixing patterns in those locations. These social structures vary across countries in different stages of development and with different demographics. Social contact patterns have been measured in a small number of countries, but in large swathes of the world, contact patterns are unmeasured, which makes it challenging to build mathematical or computer models of disease spread and control. In this work, we developed a modelling framework to combine social contact data from the past studies of contact patterns within eight countries in the EU with data from multiple data sources including the Demographic Household Surveys, World Bank and UN Statistics Division, to provide validated approximations to age-and-location-specific contact rates for 152 countries covering 95.9% of the world’s population.

## Introduction

Events over the last decade have highlighted the threat posed internationally by contact-transmissible infectious diseases such as hand, foot and mouth disease [1,2], MERS-CoV [3], Ebola [4], influenza [5], and Tuberculosis [6], putting pressure on governments and public health institutes [6] to ensure countries are pandemic-prepared. Research in social networks has shown that transmissibility, and hence the effectiveness of many interventions, is determined by the intensity of human-to-human interactions [12]. Heterogeneities in contact networks—in the sense of clustering of contacts within triadic structures and the existence of individuals or groups with many more contacts than average—has been shown in modelling studies (i) to have an effect on determining whether a pathogen can become epidemic [7–9] or can persist at endemic levels [10], (ii) to exert selective pressure for low virulence [11], and (iii) to determine which interventions can possibly mitigate an outbreak [8,12–14] or even eradicate a disease from the population [13]. While epidemic models that do not account for contact structure suffice for some research questions, such as prediction [15] or determining minimum vaccine coverage if there are no pockets of high transmission intensity or low coverage [16–19], assessing the effectiveness of interventions that specifically target social networks, such as school closure [20–22], requires models that explicitly account for such social structure. Determining contact patterns within and between different segments of the population is therefore vital to evidence-based prediction and planning with sufficient realism to inform good policy making.

To this end, a seminal study by Mossong et al. in 2008 [23] measured the social structure of ~100 000 contacts across eight European countries using paper diaries as part of the POLYMOD project. The study illuminated the strong assortativity of social contacts with age, with children in particular driving the early period of an epidemic, a finding that has been reproduced in other studies [24]. Similar studies to measure the assortativity of contacts have been conducted in a few other locations: Viet Nam [25], Taiwan [26], southern China [27], Peru [28] South Africa [29], Kenya [30], Russia [31] and Thailand [32]. However, the age and social structure of countries with different levels of socioeconomic development, family structure and at different stages of demographic transition differ substantially and contact patterns concomitantly vary across countries. The findings from the POLYMOD and limited number of other countries cannot therefore be directly applied to models of socially-transmitted infections, such as influenza, in other settings [32]. The lack of reliable contact studies in most of the world almost a decade after the original POLYMOD study highlights the logistical challenges of conducting such studies, particularly in low income countries which account for most of the infectious disease burden globally [33]. Although it is preferable for models in such a country to be based on empirical contact data from that country directly, in the absence of such data, modelers have to decide how to modify estimates from another country to fill the gap.

One approach to tailor the POLYMOD estimates to another setting would be to adjust them based on the differences between the age-profiles of that country and the eight countries from the EU that contributed to POLYMOD, inflating the projected number of contacts with younger individuals, for instance, for a country with a young age-profile, and concurrently reducing the projected number of contacts with the elderly. However, because the POLYMOD survey collected information on where each contact occurred, a more refined projection can be obtained by incorporating data on household structures, labor-force participation rates, and school enrolment, and separately projecting contacts in different locations. This has the added benefit of providing a means to model location-specific interventions.

The objective of this paper is to provide projected age-specific contact rates for countries in different stages of development and with different demographic structures to those studied in POLYMOD, which provide validated approximations to social contact patterns when directly measured data are not available. To this end, we combined data from POLYMOD, from the large scale Demographic Household Surveys (DHS), from the UN population division and from various international indicators, to project household structures and school and labor force participation rates for most countries of the world, and thereby to provide baseline projections of age-specific contact patterns in settings where contact surveys have yet to be conducted, until empirical estimates become available.

## Materials and methods

### Ethics statement

The POLYMOD data collection was approved by national institutional review boards, as previously described [23]. As no identifying information was provided, institutional review was not required for reanalysis.

### Overview of the methodology

**Fig 1:**
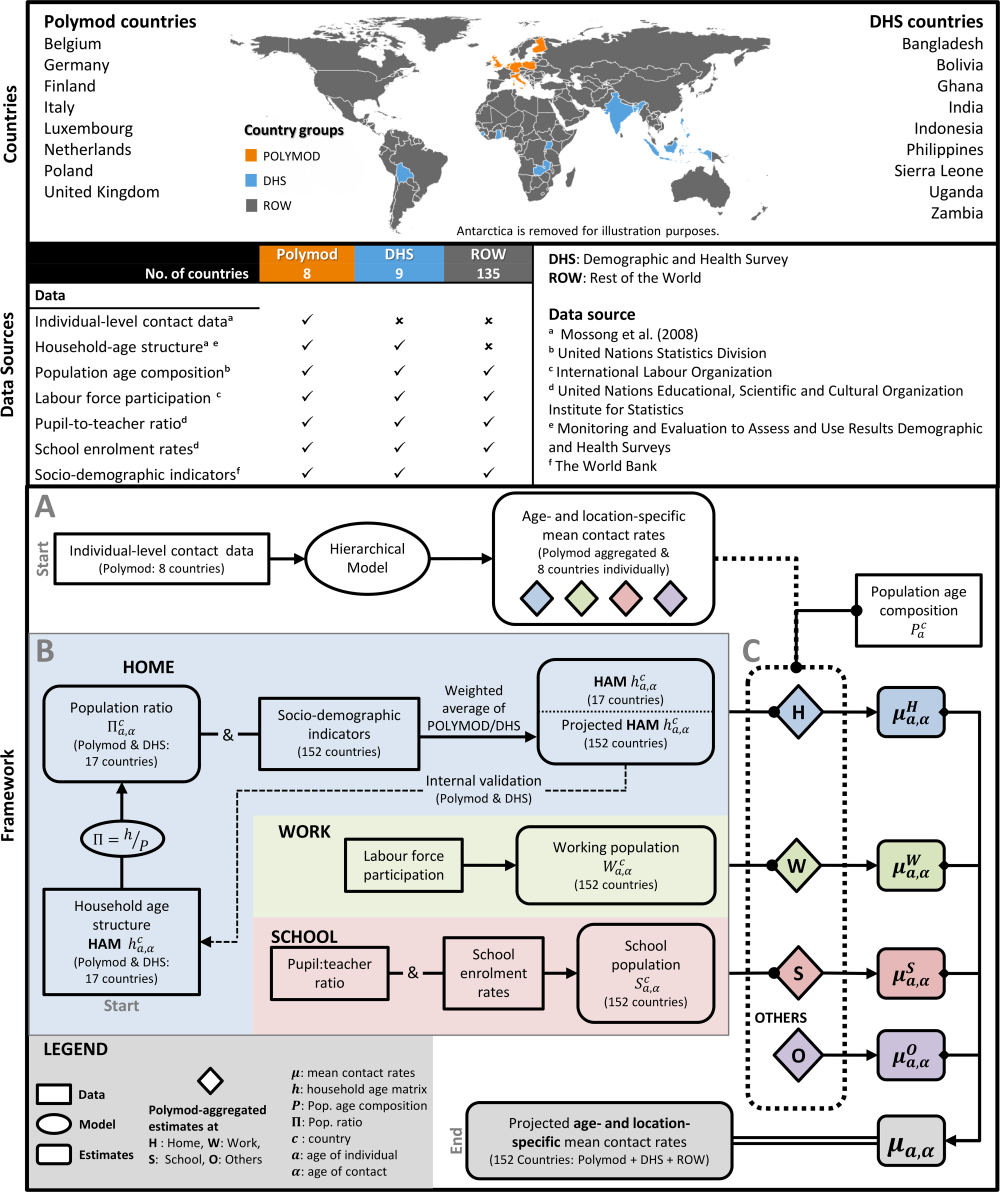
Methodology and data. Overview of the data sources and model framework in the manuscript is presented in this flow chart. The categories of the 152 countries are depicted on the world map (i.e. POLYMOD, Demographic and Health Survey (DHS), and Rest of the World (ROW) countries) and their data sources are listed in the table. A summary of the methodology is represented by the model framework: (A) POLYMOD model, (B) construction age-structured populations at home, work, and school in the 152 countries, and (C) projection of global estimates.

The overall approach is to use data fusion to combine various data sources to project age- and location-specific contact rates for a spectrum of countries in different stages of development and with different demographic structures. Fig 1 gives an overview of the data sources discussed in the following section, and the major steps of the modelling framework described in the sections that follow that. The countries (n = 152, 95.9% of the world’s population) are categorized into (i) POLYMOD, (ii) Demographic and Health Survey (DHS), and (iii) Rest of the World (ROW) countries, which are illustrated on the world map. Data availability, as used in our study, varies across the country categories: with POLYMOD having the most data available and the ROW countries having the least. The quality of the data from the same source is consistent across countries. The modelling framework starts with a Bayesian hierarchical model (A) built for the POLYMOD contact data. This model estimates age- and location-specific contact rates for the POLYMOD countries collectively and independently. The ensuing subsections detail the methodology adopted to construct age-structured populations at home, work, and school (B) is combined with the population age structure and the POLYMOD aggregated estimates to get the global projections (C). Internal validation using leave-one-out validation was conducted to verify that the household age matrices describing household structure could be reverse-engineered for countries (POLYMOD and DHS) for which empirical household age matrices were available. Not featured in this flowchart are the external validation and an example application (i.e. age-specific Susceptible-Infected-Removed modelling) that demonstrates the potential utility of these projections.

### Data

The POLYMOD study design has been fully described elsewhere [23]. In brief, cross-sectional surveys were conducted across 8 countries then in the European Union (Belgium (BE), Germany (DE), Finland (FI), the United Kingdom (UK), Italy (IT), Luxembourg (LU), the Netherlands (NL), and Poland (PL)) by commercial survey companies and public health institutes, between May 2005 and September 2006. 7,290 participants documented their contacts as they occurred on a randomly assigned 24 hour period in a paper diary, including age and gender of contacted person, and the type (physical or nonphysical), duration, location and frequency of the contact. Participant demographics were also recorded. Diaries of young children were completed by a parent or guardian. The study collected information of 97,904 contacts. In the analysis, 52 participants (0.7%) and 148 contacts (0.2%) were excluded for data quality reasons as detailed in the **S1 Text**. Because participants and contacts were mostly from the general community, the resulting contact patterns are suitable for models of diseases that are socially spread, such as influenza, but not directly usable for diseases with other routes of transmission, such as nosocomial infections.

To extend the contact rate model to other countries of the world, we synthesized the POLYMOD data with four other data sources that either inform contact patterns in households, workplaces and schools, or provide a measure of the similarity of countries. (i) The Monitoring and Evaluation to Assess and Use Results Demographic and Health Surveys (DHS) provide data for lower-income countries. We extracted household structure data for nine countries—Bangladesh, Bolivia, Ghana, Indonesia, India, the Philippines, Sierra Leone, Uganda and Zambia—for which there were no usage or copyright restrictions. (ii) The population age compositions for all countries of the world were obtained from the United Nations Statistics Division. (iii) The labor force participation rate by sex and 5-year age groups for most countries of the world were obtained from the International Labor Organization on-line database. (iv) The pupil:teacher ratio in education and the enrolment rates of students at various level of education were obtained from United Nations Educational, Scientific and Cultural Organization Institute for Statistics (UIS).

### Model

#### Hierarchical model of POLYMOD contact data

To address the multi-level structure of the data, with repeat measurements of contacts made in different settings by the same individual, we employed Bayesian hierarchical modelling to estimate the proclivity of age-specific and location specific contact patterns in each of the POLYMOD countries, as this provided a flexible framework to estimate both individual-level and population-level parameters. Throughout, we used non-informative prior distributions for all parameters, presented in the **S1 Text**, unless otherwise noted. $X_{i,\alpha }^{L}$, the number of contacts made by individual *i* at a particular location *L* (home, work, school or other) with someone in age group *α*, is assumed to be Poisson with mean $\mu_{i,\alpha }^{L}$, with a random effect *σ*_*i*_ for individual *i*. The mean was $\mu_{i,\alpha }^{H}=\sigma_{i}\;\lambda_{a_{i},\alpha }^{H}\;(\nu_{i,\alpha }+\delta_{H})$ for home contacts, $\mu_{i,\alpha }^{W}=\sigma_{i}\;\lambda_{a_{i},\alpha }^{W}\;(w_{i}+\delta_{W})$ for work contacts, $\mu_{i,\alpha }^{S}=\sigma_{i}\;\lambda_{a_{i},\alpha }^{S}\;(s_{i}+\delta_{S})$ for school contacts and $\mu_{i,\alpha }^{O}=\sigma_{i}\;\lambda_{a_{i},\alpha }^{O}$ for contacts at all other locations. The number of cohabitants of *i* of age *α*, *ν*_*i*,*α*_, represents household age structure, while *w*_*i*_ and *s*_*i*_ indicate if *i* went to work or school on the day of the survey. Contact with visitors at home, workplace or school is allowed via background contact parameters, *δ*_*H*_, *δ*_*W*_ and *δ*_*S*_. The parameter $\lambda_{a_{i},\alpha }^{L}$ quantifies typical contact rates between individuals of age groups *a*_*i*_ and *α* at location *L* and is the key estimand in the model.

The $\lambda_{a_{i},\alpha }^{L}$ parameter was given an hierarchical prior [34] to impose smoothness between successive age groups, i.e.:

$$\log\;\lambda_{a_{i},\alpha }^{L}=\sum_{A,A:A,A\in N_{a_{i},\alpha }}\frac{\epsilon_{A,A}^{L}}{\left|N_{a_{i},\alpha }\right|}$$ (1)

where $\epsilon_{A,A}^{L}$ is a hyperparameter of $\lambda_{a_{i},\alpha }^{L}$ and $N_{a_{i},\alpha }$ is the set of (up to) 4 adjacent age groups together with (*a*_*i*_,*α*) itself.

The posterior distributions of the parameters were estimated via Markov chain Monte Carlo simulation [35]. The inference was implemented using Just Another Gibbs Sampler (JAGS) [36] within the R statistical environment (R Core Team, 2013) using the rjags package [37] with 100 000 iterations for each of the eight POLYMOD countries independently and collectively. Convergence of the Markov chain Monte Carlo samplers using the Heidelberger-Welch diagnostic which was passed for 89% of parameters (median effective sample size 52 000, inter-quartile range 20 000–78 000).

#### Extension to the non-POLYMOD countries: Projection of age-specific contacts at home

To derive typical contact patterns at home for countries not present in POLYMOD or DHS (rest of world, or ROW), and hence without household structure data, we projected the household age matrix (HAM) for country ***c***, $\left(h_{a,\alpha }^{c}\right)$, equal to the mean number of household members of age ***α*** of an individual aged ***a***. We then combined the HAM for POLYMOD/DHS countries with data on the age structure of the population of the country as a whole for both POLYMOD/DHS and ROW countries.

We define the population ratio matrix $\left(\Pi_{a,\alpha }^{c}\right)$—which measures the propensity of an individual aged *a* having a household member aged *α* after adjusting for the population age structure of country *c*—of the 17 POLYMOD/DHS countries using

$$\Pi_{a,\alpha }^{c}=h_{a,\alpha }^{c}/P_{a}^{c},$$ (2)

i.e. dividing the elements of HAM by the proportion of the population of country *c* aged *a*, $P_{a}^{c}$.

As $P_{a}^{c}$ is known for all countries, we can project the household age structure for ROW countries by extrapolating the relationship between population age profile and household age structure as estimated from the POLYMOD/DHS countries, and applying that to the country’s population age profile. To account for differences in the mapping due to developmental and social differences, we used a weighted average across POLYMOD/DHS countries, where weights were derived for 152 countries of the world (covering 95.9% of the world’s population) using nine indicators: gross domestic product (GDP) per capita, total fertility rate (TFR), population density, population growth rate, under-five mortality rate (U5MR), health expenditure per capita, life expectancy of males, as a proxy for overall health, Internet penetration rate, and urbanization rate in the country, all standardized by z-scoring. Specifically, we projected the HAM of the ROW countries using the weighted mean of the population ratio matrices of the 17 POLYMOD/DHS countries. To determine the weights, we calculated the pairwise Euclidean distances of each of the nine standardized variables of these 135 ROW countries with the 17 POLYMOD/DHS countries. Because $P_{a}^{c}$ should be proportional to $\sum_{\alpha }\;P_{\alpha }^{c}h_{\alpha ,a}^{c}$, we improved the raw projections by generating 10 000 bootstrap samples of the pairwise distances between countries (between indicators) as a ‘prior’ distribution, and selected the combination that maximized the correlation between $P_{a}^{c}$ and $\sum_{\alpha }\;P_{\alpha }^{c}h_{\alpha ,a}^{c}$.

Having projected the household age matrix $\left(h_{a,\alpha }^{c}\right)$ for ROW countries, for both DHS and ROW countries, the age-specific contact at home $\mu_{a,\alpha }^{H,c}$ for country *c* is then set to:

$$\mu_{a,\alpha }^{H,c}=\lambda_{a,\alpha }^{H}\times h_{a,\alpha }^{c}.$$ (3)

Here, $\lambda_{a,\alpha }^{H}$ is the overall POLYMOD estimates obtained from analyzing the data from the original POLYMOD study.

#### Age-specific contacts in the workplace

We allowed the number of age-specific contacts made at the workplace to depend on the age structure of the work force. The labor force participation rate of age *a* in country *c*, $w_{a}^{c}$, was obtained from the International Labor Organization and used to compute a joint distribution of the working population, (*W*^*c*^), a square matrix with elements describing the probability of encounters between two ages in the workforce, $W_{a,\alpha }^{c}=w_{a}^{c}\times w_{\alpha }^{c}$.

After obtaining the working population distribution of ages, (*W*^*c*^), we projected the age-specific contact patterns in the workplace for non-POLYMOD countries by multiplying the aggregate POLYMOD's estimates of $\lambda_{a,\alpha }^{W}$ with the working population and the ratio of the population age structures:

$$\mu_{a,\alpha }^{W,c}=\lambda_{a,\alpha }^{W}\times W_{a,\alpha }^{c}\times \frac{P_{\alpha }^{c}}{P_{\alpha }^{POLYMOD}}.$$ (4)

#### Age-specific contacts in school

Similarly to the approach used for workplaces, we projected the age-specific proportion of the population in school, including teachers, before modelling the contact patterns in the school. Information on enrolment rates by education level and age ranges of education were obtained from UIS. With the population age structure $P_{a}^{c}$ and the enrolment levels by age $e_{a}^{c}$ we projected the number of students aged *a* to be $s_{a}^{c}=P_{a}^{c}\times e_{a}^{c}$.

We determined the number of teachers $n_{t}^{c}$ in country *c* using data on pupil-teacher ratios (class size $C^{c}$) from UIS and number of students $\sum_{a}s_{a}^{c}$ by the equation

$$n_{t}^{c}=\frac{C^{c}}{\sum_{a}s_{a}^{c}}.$$ (5)

We then projected the number of teachers by age

$$t_{a}^{c}=P_{a}^{c}n_{t}^{c}w_{a}^{c}.$$ (6)

The school population distribution of ages (*S*^*c*^) with elements signifying the probability of encounters between two ages within school was obtained using

$$S_{a,\alpha }^{c}=\left(\frac{s_{a}^{c}}{\sum_{i}s_{i}^{c}}+\frac{t_{a}^{c}}{\sum_{i}t_{i}^{c}}\right)\times \left(\frac{s_{\alpha }^{c}}{\sum_{i}s_{i}^{c}}+\frac{t_{\alpha }^{c}}{\sum_{i}t_{i}^{c}}\right)$$ (7)

although for most age groups, at least one of $s_{a}^{c}$ and $t_{a}^{c}$ will be 0. With the population in school known we can now deduce the possible age-specific contact patterns in school by the following expression:

$$\mu_{a,\alpha }^{S,c}=\lambda_{a,\alpha }^{S}\times \frac{P_{\alpha }^{c}}{P_{\alpha }^{POLYMOD}}\times S_{a,\alpha }^{c}.$$ (8)

#### Age-specific contacts at other locations

We determined age-specific contact pattern at other (non-home, school or work) locations $\mu_{a,\alpha }^{O,c}$ by multiplying the ratio of population age structure of the country and the POLYMOD countries to the estimates of $\lambda_{a,\alpha }^{O}$ obtained from the POLYMOD analysis,

$$\mu_{a,\alpha }^{O,c}=\lambda_{a,\alpha }^{O}\times \frac{P_{\alpha }^{c}}{P_{\alpha }^{POLYMOD}}.$$ (9)

Graphs were created in the grid package [38] in R.

#### Validation

Two forms of validation were conducted. *Leave-one-out validation* was performed to reconstruct the HAM of one POLYMOD or DHS country at a time. This involved projecting that country’s household age structure as if it were unknown and comparing against the actual structure. To validate the ROW procedure, we treated the POLYMOD countries as an ROW country to obtain the projected contact matrices. These projections were compared to the empirical POLYMOD data (**S1 Text**), We also *externally validated* the projected contact matrices by comparing the projected matrices to recently conducted contact surveys in five low and middle income countries in three continents (Kenya, Peru, Russia, South Africa, and Viet Nam [25,28–31]), as described in the **S1 Text**. For most of the projections or data from these countries, we computed the mean contact rates by age of individuals and contrasted the estimates and the projected contact matrices generated using the method described above.

#### Age-specific SIR modelling

To demonstrate the application of the projected contact matrices, we performed age-structured Susceptible-Infected-Removed (SIR) modelling [39] using the derived age-specific contact matrices for countries of different levels of development. For two pandemic influenza scenarios (*R*_0_ = 1.2 and 1.5), the age-specific final epidemic size and the percent reduction in infection were calculated for three scenarios: No intervention (total contacts calculated as a sum of contacts made at home, work, school and other), School closure and social distancing of younger individuals (zero contribution from school contacts and reduction in contacts at other locations with individuals below 20 years and a small increase in contacts made at home) and Workplace distancing (reduction of work contacts by a half). These were obtained by scaling the contact matrices to obtain the scenario’s *R*_0_, setting the removal rate without loss of generality to one. We initialize the SIR model with starting immunity levels derived from age-specific susceptibility data from the study by Miller et al. [40]. More details are in the **S1 Text**.

## Results

### Contact patterns at home

**Fig 2:**
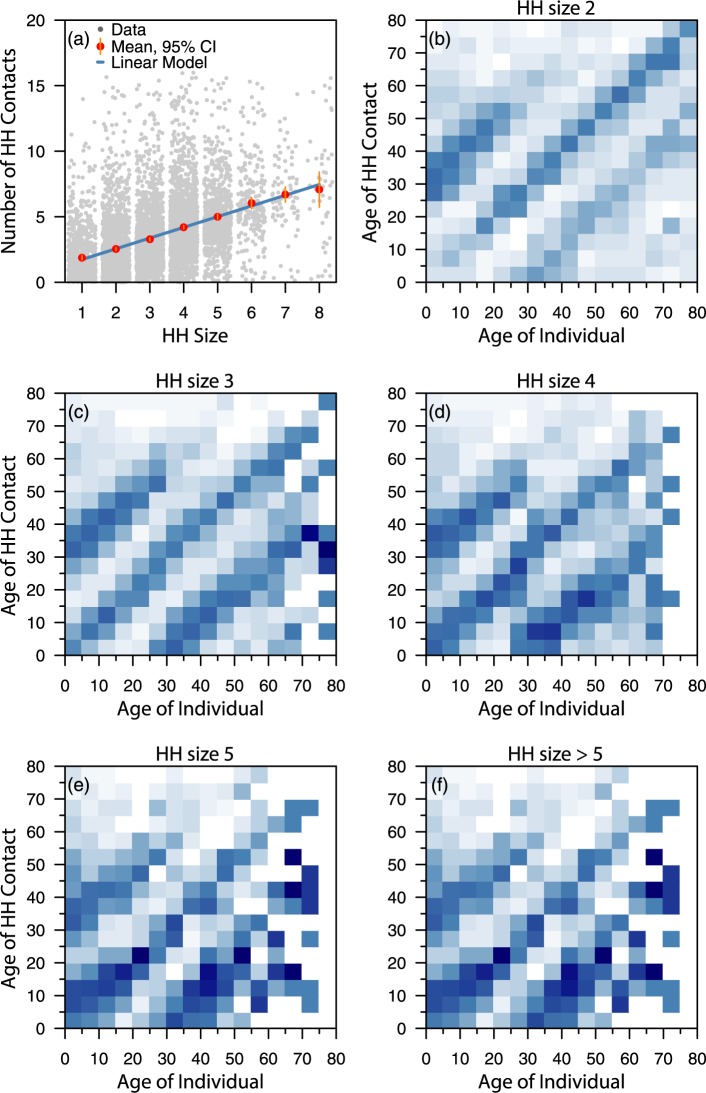
Contacts at home stratified by household size. Panel a shows the number of contacts made by individuals at home (grey dots) in the POLYMOD study stratified by household sizes. The estimated mean number of contacts (red dots) made by an individual and the 95% confidence interval (orange lines) are shown. By observation, a near linear relationship exists between the household size and the number of contacts. The blue line represents a fitted linear model. The panels b–f show the age-specific contact patterns at home of individuals stratified by household sizes. Darker color intensities indicate more contacts were made.

Fig 2 (panel a) shows the number of contacts at home made by individuals in the POLYMOD study stratified by household sizes. A near linear relationship is observed between the household size and the number of contacts, suggesting a frequency dependent relationship, rather than a density dependent one [41]. The age-specific contact patterns at home, including with visitors to the household, for the POLYMOD countries collectively are presented in Fig 2, stratified by household size. A central diagonal is present for all household sizes, indicating assortativity of mixing with age, with secondary diagonals in households with at least 2 members. These secondary diagonals become more prominent with increasing household sizes.

**Fig 3:**
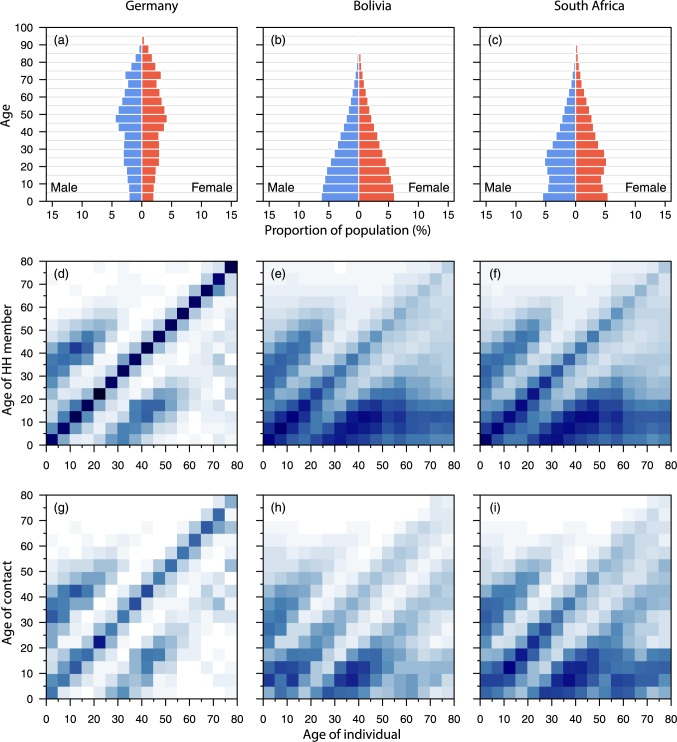
Population and household age distribution, and age-specific contacts at home. The population pyramids by age and gender (panels a–c), household age matrices (panels d–f) and age-specific contact patterns (panels g–i) are presented for Germany (first column, as a representative of the POLYMOD countries), Bolivia (second column, as a representative of DHS) and South Africa (third column, as a representative of ROW). The population pyramids, panels a–c, and household age matrices (for only POLYMOD and DHS), panels d–e, are observed data. The age-specific contacts at home for Germany (g) is estimated from our hierarchical model. The household age matrix for South Africa (f) and the age-specific contacts at home for Bolivia (h) and South Africa (i) were projected using the described methods. Darker color intensities indicate more likely events i.e. greater tendency of having a household member of that age, higher proclivity of making the age-specific contact.

To illustrate some of the results, Germany, Bolivia, and South Africa are arbitrarily selected as country representatives of the POLYMOD, DHS and ROW, respectively. Results for the other 149 countries in the study can be found in the **S1 Text**. The population pyramids of Bolivia (a DHS country, panel b) and South Africa (a ROW country, panels c) in Fig 3 have the triangular shape common to countries still undergoing the demographic transition, while that of Germany (in POLYMOD, panel a), with its narrow base, indicative of sub-replacement fertility, is similar to other aging populations. For all three countries, both the household age matrices (panels d–f) and age-specific contact patterns at home (panels g–i) have similar features: (i) a prominent central diagonal corresponding to interactions with siblings (for younger individuals) and partners (for adults) and (ii) two parallel secondary ridges about one generation distant from the main diagonal, which start around age 25 and reflect parent-child contacts. Together, these suggest that the contacts are dominated by two-generation familial structures for these three countries, although other countries such as India display evidence of three-generational structures. Results for India and 151 other countries are presented in the **S1 Text**. Average household sizes vary across countries, with some Asian and African countries having larger households than those in the POLYMOD study.

**Fig 4:**
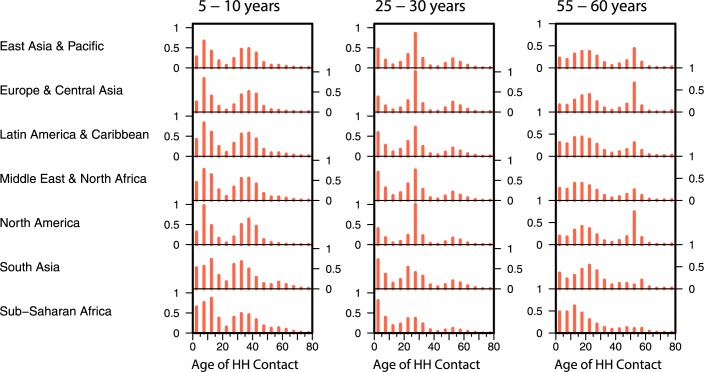
Inferred regional contact patterns at home. Countries of the world were group into 7 regions (East Asia & Pacific, Europe & Central Asia, Latin America & Caribbean, Middle East & North Africa, North America, South Asia and Sub-Saharan Africa). The regional mean age-specific contact patterns at home (inferred) of individuals aged 5–10 (first column), 25–30 (second column) and 55–60 (third column) years were represented as bars.

We grouped all countries into 7 regions according to the World Bank’s definition (East Asia & the Pacific, Europe & Central Asia, Latin America & the Caribbean, the Middle East & North Africa, North America, South Asia and Sub-Saharan Africa) [42]. To obtain projected regional norms for contact patterns at home we computed the mean contact rates that were projected or inferred earlier within the region weighted by each country’s population size. Fig 4 shows the age-specific contact patterns at home of individuals aged 5–10, 25–30 and 55–60 years by region. Proportionally more contacts are made by primary school aged individuals at home in Latin America & the Caribbean, South Asia and Sub-Saharan Africa are higher than of the other regions, a reflection of the larger households in these regions. The same age group also makes more contacts with those in the older age groups than their peers in other regions. For individuals in the age group 55–60, the mean number of contacts made with younger individuals in South Asia is significantly higher than those in other regions. Contact matrices for India and Bangladesh have additional tertiary bands (**S1 Text**), suggesting a greater preponderance of three-generation households. There were several countries, such as Sierra Leone and Burkina Faso, where we infer a deviation from that pattern as a result of skewed population structure (**S1 Text**).

### Non-household contacts

**Fig 5:**
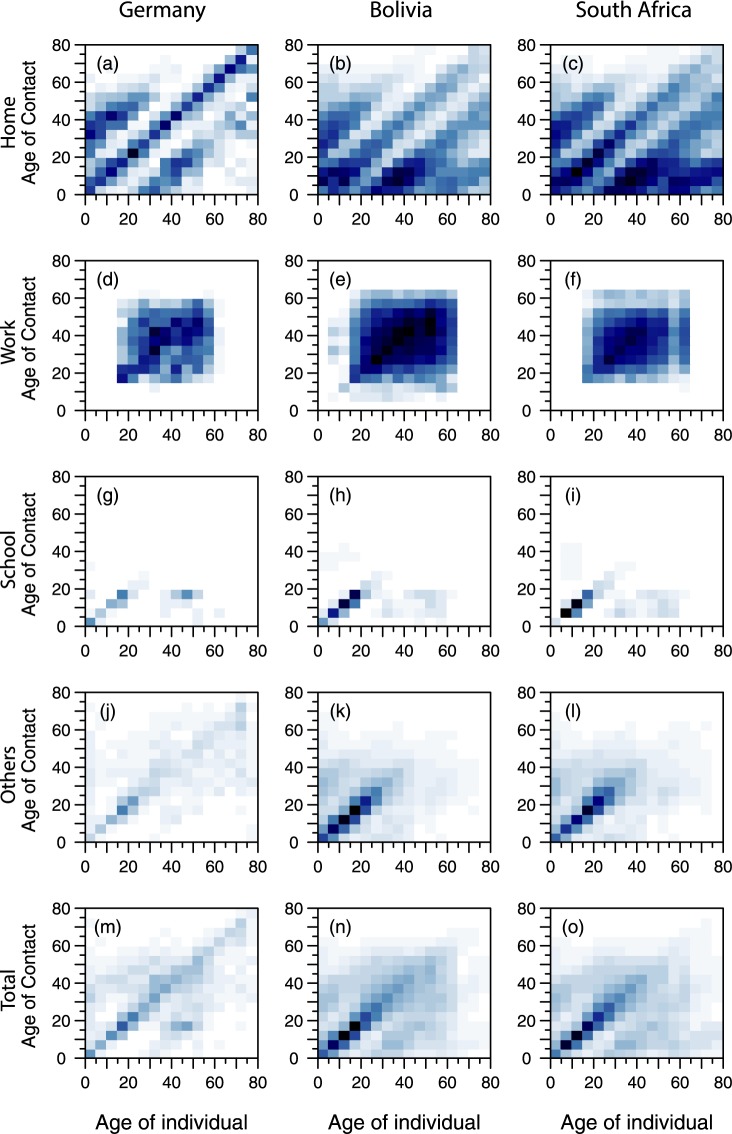
Age-specific contact patterns by location. The age-specific contact patterns at home (panels a–c), at the workplace (panels d–f), in school (panels g–i) and at other locations (panels j–l) are projected from the model. The contact pattern at all locations (panels m–o) is the sum across the four locations (home, work, school and others). Contact matrices for Bolivia (DHS country; in panels b,e,h,k) and South Africa (ROW country; in panels c,f,i,l) were projected and the age-specific mean contact rates for Germany (part of the POLYMOD; in panels a,d,g,j) were estimated from the German contact data. A comparison between the German empirical and modelled estimates can be found in the **S1 Text**. Darker color intensities indicate higher proclivity of making the age-specific contact.

Modelled projections of contact patterns in different locations—home, work, school, other and all—for Bolivia, Germany and South Africa, are plotted in Fig 5. In Germany, for which it was explicitly measured, the age-specific contact patterns (panels d–f) at the workplace show wide clusters of contacts among working ages (20–60), indicating relatively homogenous mixing in this setting. Adapting this finding to Bolivia and South Africa, accounting for the age structure of their labor forces, led to similar homogeneity in workforce contacts there. Unlike in households, the presence of more diverse age structures in workplaces could provide a ready channel for transmission between distinct age groups, separated in the main from each other within the home setting.

Intense mixing, indicated by the pronounced central diagonal, is present in the age-specific school contact pattern (panels g–i), suggesting the importance of this milieu for transmission potential within this age group. Within schools, the assortativity of mixing patterns is more pronounced in younger individuals (below the age of 25), while those of working-age (teachers and support staff aged 30–60) have more moderate contact rates between themselves and younger individuals, the latter characterizing student-teacher interactions. This pattern was present across other countries (**Supporting Information**).

Similar to the home and school contact patterns, a strong central diagonal band and at times weak secondary diagonals can be observed in the projected contacts made at other (non-home, non-work, non-school) locations. This assortativity contrasts with the work environment. However, apart from the leading diagonal, the contact patterns within this other grouping vary across countries in a non-systematic way.

Aggregating contact rates from the four locations (home, work, school and others) indicates that the strong central diagonal due to school and home contacts dominates overall contact rates.

#### Validation

The empirical household age-structures for the POLYMOD and DHS countries were reconstructed with high fidelity (median correlation between inferred and empirical 0.93, inter-quartile range 0.91–0.95; plotted in the **S1 Text**). The projected contact matrices for the POLYMOD countries when treated as an ROW country were similar to the POLYMOD contact data, thus validating the ROW procedure. The age-specific contact matrices observed in five contact surveys in non-POLYMOD countries were compared against our projected matrices (**S1 Text**). The two have a generally close correspondence, within the constraints of the small sample sizes when stratified by age in the Kenyan and Vietnamese studies, which lead to relatively large standard errors and more of an apparent discrepancy.

### Age-specific final epidemic sizes

**Fig 6:**
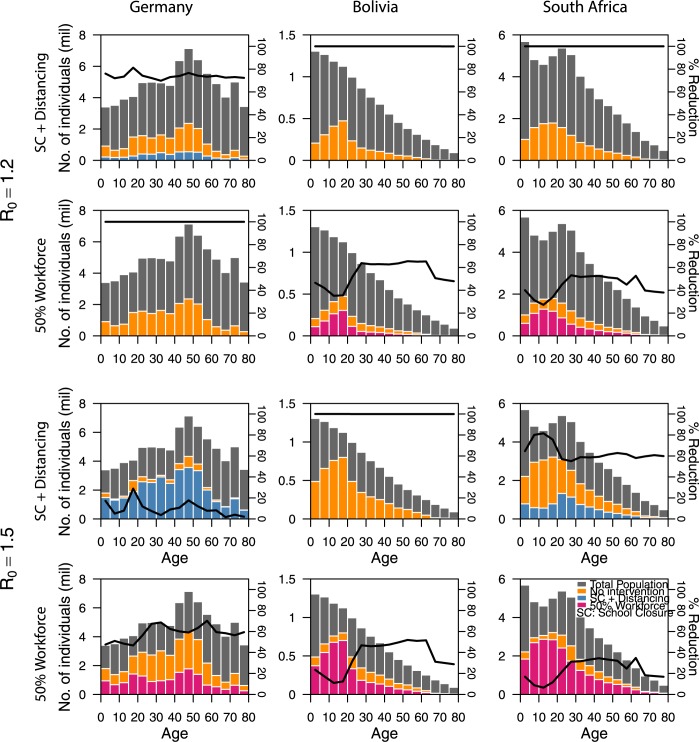
Age-specific final epidemic size and percentage reduction. The age-specific final epidemic size and percentage reduction of infection for Germany (first column), Bolivia (second column) and South Africa (third column) are shown for the three interventions: No intervention (sum of orange and pink/blue bars), School closure and social distancing of younger individuals (blue bars) and Workplace distancing (pink bars) for two epidemics with *R*_0_ of 1.2 and 1.5. The percentage reduction of infection for the various intervention and *R*_0_ values are represented by the black lines.

The distribution of age of infected cases under two pandemic scenarios is presented in Fig 6 for Germany, Bolivia and South Africa. Germany’s relatively older population leads to more infection among adults than the younger countries of Bolivia and South Africa, and also to a lower effectiveness of school closure (which is modelled to reduce infection rates to ~80% for *R*_0_ = 1.2 and less than 20% for *R*_0_ = 1.5). However, school closure and social distancing of younger individuals is expected to be effective in preventing an outbreak entirely in younger populations like Bolivia and South Africa, Workplace distancing has a greater impact on older populations like Germany (preventing an outbreak entirely for *R*_0_ = 1.2 and reducing its size by ~50–70% for *R*_0_ = 1.5) than younger populations like Bolivia and South Africa (~30–70% for *R*_0_ = 1.2 and ~10–50% for *R*_0_ = 1.5).

## Discussion

A decade after POLYMOD, age-structured contact matrices based on random population-based samples have been published for only a limited spectrum of countries: eight European Union countries [23], a handful in Asia [25–27,31,32], one in Latin America [28] and two in Sub-Saharan Africa [29,30]. The risk of emerging diseases spreading from other animals to humans is not restricted to these areas [43] (as the recent MERS and Ebola events illustrate [3,4]), and there is a pressing need for contact matrices representing more diverse countries around the world for models of socially-spread diseases to be built. This study presents data-driven contact matrices for 152 countries of the world for the first time.

Individuals’ interactions are non-random and, because they are contingent on the physical presence of the individual and contact, vary by location. As in the original analysis of these data [23], we found that household, workplace and school structures across the world are consistent with age-specific contacts made by individuals, in that they are highly assortative, with much more frequent interactions with others of a similar age group. However, these precise age-dependent patterns differed by location and across the countries we studied.

High assortativity of contacts is observed in schools but, at least in the POLYMOD countries, is less apparent in working-age individuals in the workplace. The former was expected, but the patterns of contacts in the workplace, with greater heterogeneity in the age of contacts reflecting a more diverse age structure, may provide a route for transmission to spread between families with school-age children and the rest of the population, in a similar way to the bridging role of bisexuals between hetero- and homosexual networks [44]: our estimates assume the same patterns apply in other countries, and future research should validate this. Glass and Glass [24] found similar assortativity among younger age groups, and proposed that this assortativity made those in younger age groups the transmission backbone of respiratory epidemics. Simulation studies using the POLYMOD data [23] and others [26,45,46] support this conclusion and explain why children and teenagers were the major channels for the initial transmission of infection during the 2009 influenza A(H1N1) pandemic [47], and why school closure is one of the main non-pharmaceutical interventions considered for pandemic mitigation [20,48]. Merler et al. found that spatiotemporal spread of the H1N1 2009 pandemic in Europe were influenced by the age-mixing patterns and social structures [49]. While age-mixing patterns shape the transmission of infectious disease [49], analyses of the 2009 H1N1 influenza pandemic [50,51] suggest that it is crucial to account for age-specific susceptibility to infection.

For most of the countries considered, three pronounced diagonal bands were observed in the contact matrices at home. This is partly a reflection of the structure found in the POLYMOD survey itself [23], but is also consistent with the much larger DHS surveys which provide detailed data on household structures directly [52]. For the Asian countries in the DHS samples, noticeable tertiary diagonals, reflecting three generation households, were present, which highlight the limitations of using data from Europe to represent non-European societies without adjustments similar to those performed in this analysis. Interactions between school-going children and the elderly have important public health implications, as the former may have high infection rates while the latter are vulnerable to complications from infections, such as pneumonia [53,54]. Evidence for the effect of transmission from children to the elderly comes from Japan, where the cessation of the school influenza vaccination program led to a rise in mortality among the elderly [55]. Although we have used contact data only from the POLYMOD to build the global contact matrices, contact studies in Asia, Latin America and Sub-Saharan Africa (from Viet Nam [25], Taiwan [26], Southern China [27], Peru [28], South Africa [29] and Kenya [30], Russia [31]) found similar evidence of tertiary diagonals in the contact matrices at home. The consistency of their survey-based findings with the emergent properties of our model provides some degree of empirical support to our findings. Synthetic contact matrices have been generated by individual-based model simulations [56] or derived from socio-demographic variables [57,58] and validated on serological data of H1N1 Influenza [56,58], varicella and parvo-virus [57] using age-structured SIR models. Some of these synthetic contact matrices were created for only one country (Hong Kong [56] and Italy [57]), while Ref. [58] estimated contact matrices for 26 European countries. The Fumanelli estimates [58] in particular did not reproduce the narrowness of the leading diagonal in the contact matrix observed in the POLYMOD study, in contrast to our approach (more in the **S1 Text**). Despite the lack of social contact surveys, synthetic contact matrices have only developed for higher-incomed countries [56–59], with large proportions lower-and-middle-income countries unrepresented.

There are several assumptions underlying our study. We assumed the number of contacts in each location was Poisson, with over-dispersal accounted for using an individual-level random effect term that was assumed to govern contacts in all four locations, but in principle additional heterogeneities might be present leading to mischaracterization of correlations, dispersion [60], and the number of zeros. As in the original POLYMOD study, we only quantified age-specific dyadic contacts, as eliciting higher order contacts in a survey is challenging both cognitively and practically. Triadic contacts—where A contacts B contacts C contacts A—are important for disease propagation in contact network models [12,61] and other methods to measure these, such as radio frequency tagging [61,62], or more sophisticated structural models accounting temporal presence within the household [63], may be needed to characterize higher degrees of contact. However, it remains to be demonstrated empirically that the differences in the patterns of transmission of an infection between two countries can be explained by differences in their contact patterns, although our simulation study in three countries suggested that the effect of interventions can vary substantially based solely on changes in contacts driven by age structure. The short simulation study in this paper made simplistic assumptions about how social distancing measures would translate into a reduction in contact rates, as well as assuming that heterogeneities in contact patterns could be well-described by an age-structured but otherwise mean-field model. In actual usage, the translation of a policy into a specific change in contact rates should be supported by evidence, while individual-based simulation models [64] provide a more flexible framework to capture shared contacts between individuals and allows policies such as reactive school closure to be assessed[65].

The primary limitation of the paper is that the projected contact matrices are derived from assumptions about the social structure in countries for which contact data are unavailable, so that the projected contact patterns cannot in most cases be directly validated. Our extension of POLYMOD to most countries of the world involved two main modelling steps with inherent assumptions, elaborated below.

The first main modelling step was the creation of household structures and the demographics of workforce/school participation for countries for which this was not measured. For households, this involved identifying similarity to countries for which household data were available (using POLYMOD or DHS), using a mixture of indicators on the economy and demography of each country, and applying a mapping from the age pyramids of countries to their household structure. The variables used gave more weight to pairs of countries that for the most part make intuitive sense (for instance, Germany (POLYMOD) is assigned high weight in constructing Austria’s (ROW) contact matrices). This approach did, however, mean excluding several small countries with missing information, covering 4.1% of the world’s population. The variables were arbitrarily selected to span measures of health and social structure (fertility, mortality, growth, and health expenditure), as well as development (income, internet penetration and urbanization). The scree plot of the eigenvalue decomposition of these variables suggested that beyond 4 or 5 dimensions, additional variables did not add much new information, but alternative measures of distance would have resulted in some differences in the projected matrices. On validation, although there were some systematic discrepancies (for instance, the slightly off-center leading diagonal for some Asian countries, which we suspect might be corrected by accounting for gender in future work), this approach reconstructed the empirical household structures with otherwise high accuracy for both the POLYMOD and DHS countries. We had substantially less data on potential school and work contacts, because direct samples from these environments were not available, even in POLYMOD, and so the approach extrapolated from indirect measures (workforce and school participation rates, teacher to student ratios). This means that the number and age-profile of contacts outside the home are inherently less well projected by our approach than those within the home. It is not clear how this approach could be improved for these location types, or for ‘other’ locations, however, without collecting substantial amounts of new data. The original POLYMOD study found highly assortative mixing in ‘other’ locations, and while the study in Russia [31] found a similar pattern, it is not clear whether this would be observed in other, non-European or Eurasian settings.

The second major modelling step was to project from POLYMOD to non-POLYMOD countries the relationship between (i) household structure or workforce/school participation and (ii) contact rates within those locations. This approach implicitly assumes that given a potential contact in a given location (for instance, the existence of a cohabitant), the chance of an actual interaction is the same in POLYMOD and non-POLYMOD countries. The approximate validity of this assumption is supported by the broad similarities between our projections and estimates from the limited number of contact studies conducted outside Europe [25–31], but some discrepancies were found in this validation. It is unclear to what extent these are due to violations of the assumptions made in this paper, due to differences in study design—such as interviewer- versus self-administered questionnaires, or translation effects—or due to differences between study populations and the country as a whole due to recruitment in a limited geographical area. Further empirical studies in other countries would provide additional validation.

Those limitations notwithstanding, the projected contact matrices outlined in this paper provide a basis for model-based analyses to inform public health policy making around the world, until comprehensive studies can be carried out that cover a greater fraction of the world’s population.

## Supporting information

S1 Text Supporting information. (PDF)

S1 Dataset Age-and-location-specific contact matrices for 152 countries. (ZIP)
