# Supplementary material for: Projecting social contact matrices in 152 countries using contact surveys and demographic data
Source: PLoS Comput Biol. 2017 Sep 12;13(9):e1005697. doi: 10.1371/journal.pcbi.1005697 (PMC5609774; doi:10.1371/journal.pcbi.1005697)
Supplement: S1 Text — (PDF) [file pcbi.1005697.s001.pdf]

Supporting Information

Projected contact matrices in 152 countries for models of contact-transmissible infectious diseases

Kiesha Prem, Alex R Cook, Mark Jit

Contents

1 POLYMOD Contact Patterns **2**

1.1 Model 5

1.2 Prior distributions for parameters 5

1.3 Posterior distribution of parameters 6

2 Validation **7**

2.1 Weights for the HAM projection 7

2.2 HAM Validation for POLYMOD and DHS Countries 8

2.3 POLYMOD Validation 10

2.4 External Validation 15

2.5 Outlying Countries 16

2.6 Comparison with Fumanelli et al., 2012 18

3 Application of Contact Matrices **19**

3.1 Age-specific SIR Modelling 19

Annex A: Validation Plots of Household Age Matrices **22**

Annex B: Validation Plots of Contact Matrices with Surveys **28**

Annex C: Comparison with Fumanelli et al., 2012 **28**

Annex D: Projected Contact Matrices of 152 countries **28**

1 POLYMOD Contact Patterns

Details of the mathematical model used to estimate the proclivity of age-specific and location specific contact patterns in each of the POLYMOD countries are provided below.

The data collection procedure in the POLYMOD study by Mossong et al. (2008) involved eight European countries listed in Table 1. 7 290 participants were recruited and recorded information for 97 904 of their contacts. However, 52 participants (0.7% of the total; 36 reported no contacts and 16 reported contacts with missing ages) and 148 contacts (0.2% of total; 120 with missing age and 28 with missing location) were excluded in the analysis for data quality reasons

There are variations in absolute numbers of contacts across the POLYMOD countries (Supplementary Figure 1) which may reflect slight differences in study design such as questionnaire translation. However, the magnitude of variation between countries is substantially less than the variability between individuals, which we accommodate via a random effect.

Country	Country code	n
Belgium	BE	750
Germany	DE	1,341
Finland	FI	1,006
United Kingdom	UK	1,012
Italy	IT	849
Luxembourg	LU	1,051
Netherlands	NL	269
Poland	PL	1,012

Table 1: Number of individuals in each country.

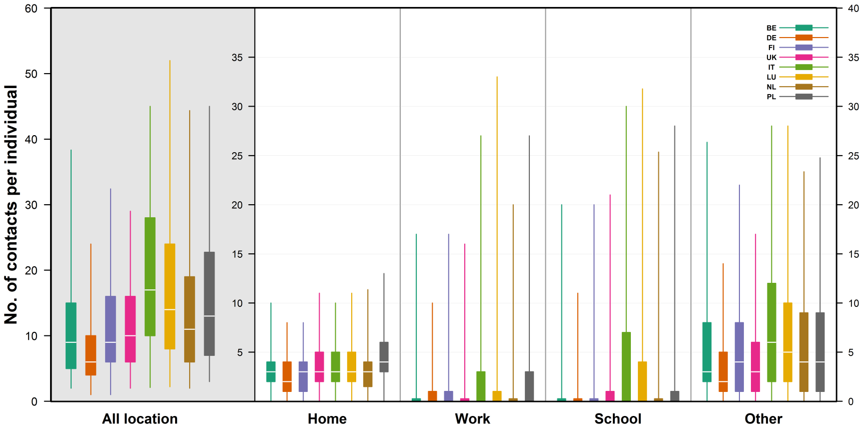

Supplementary Figure 1: Number of contacts per individual by location. Country-specific contact distribution by location are shown in the boxplots, with boxes bounded by the interquartile range (25th and 75th percentiles), median in white and, whiskers spanning the 2.5–97.5th percentiles. The boxplots show that the variability between individuals is substantially more than the variability between countries in the POLYMOD study.

The distribution of the number of age-specific contacts made at school for school-going individuals in the 8 Polymod countries (in Supplementary Figure 2) is suggestive of assortative (similar ages) mixing in schools. Discrepancies observed could be due to the different organizational structures of the schools. Teacher-student interactions are also observed (contacts that are about 2 age groups or more away).

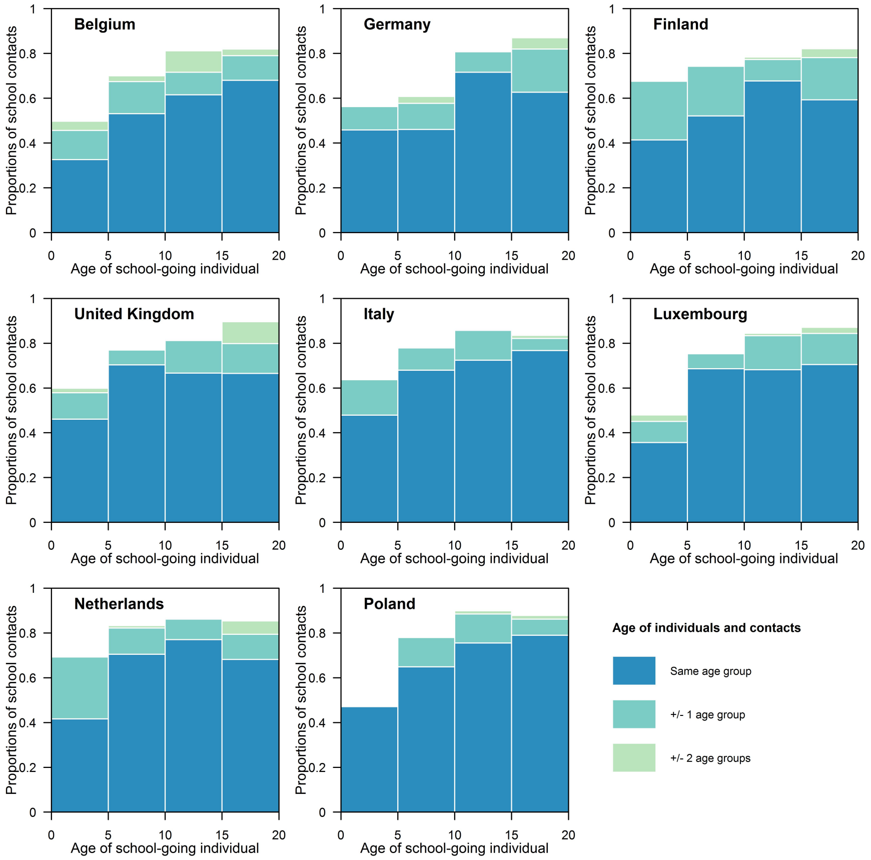

Supplementary Figure 2: Mean number of age-specific contacts made at school for school-going individuals in the 8 Polymod countries. Age groups are in five year intervals.

1.1 Model

The number of contacts made by individual i at a particular location L with someone in age group α, $X^L_{i,\alpha}$, is modelled to be Poisson with mean $\mu^L_{i,\alpha}$,

$$X^L_{i,\alpha} \sim \text{Po}(\mu^L_{i,\alpha}) \tag{1}$$

where

- the ages of individual i, a_i, and his contact, α, are categorised into 5-year age intervals, $\{1, 2, \ldots, 16\}$;
- L indicates the location of the contact namely home (L=H), work (W), school (S) and other (O).

The Poisson mean parameter has the general form $\mu^L_{i,\alpha} = \sigma_i \lambda^L_{a_i,\alpha}$ and varies across locations. In this model, σ_i is a random effect belonging to individual i which characterises differences in social activity levels across locations and allows for greater than Poisson variability in the number of contacts. The parameter $\lambda^L_{a_i,\alpha}$ quantifies typical contact rates between individuals of age groups a_i and α at location L and is the key estimand in the model.

Rationale for Hierarchical Model

We employed Bayesian hierarchical modelling to estimate the proclivity of age-specific and location specific contact patterns in each of the POLYMOD countries to address the multi-level structure of the data, with repeat measurements of contacts made in different settings by the same individual. This provides a flexible framework to estimate both individual-level and population-level parameters.

The $\lambda^L_{a_i,\alpha}$ parameter was given an hierarchical prior to impose smoothness between successive age groups, i.e.:

$$\log \lambda^L_{a_i,\alpha} = \sum_{\mathcal{A},A:\mathcal{A},A \in \mathcal{N}_{a_i,\alpha}} \frac{\epsilon^L_{\mathcal{A},A}}{|\mathcal{N}_{a_i,\alpha}|} \tag{2}$$

where $\epsilon^L_{\mathcal{A},A}$ is a hyperparameter of $\lambda^L_{a_i,\alpha}$ and $\mathcal{N}_{a_i,\alpha}$ is the set of (up to) 4 adjacent age groups together with (a_i, α) itself.

1.2 Prior distributions for parameters

Non-informative prior densities were assumed for the all the parameters and hyperparameters in the model, as tabulated in Table 2.

Parameter	Location	Notation	Prior Density
Sociability parameter	All	σ_i	$\sigma_i \sim \text{Ga}(\theta, \theta)$
Sociability dispersion	All	θ	$\theta \sim \text{Exp}(0.0001)$
Background contact parameter	Home	δ_H	$\delta_H \sim \text{Exp}(0.0001)$
	Work	δ_W	$\delta_W \sim \text{Exp}(0.0001)$
	School	δ_S	$\delta_S \sim \text{Exp}(0.0001)$
Smoothing parameter	Home	$\epsilon^H_{\mathcal{A},A}$	$\epsilon^H_{\mathcal{A},A} \sim \text{N}(0, 100^2)$
	Work	$\epsilon^W_{\mathcal{A},A}$	$\epsilon^W_{\mathcal{A},A} \sim \text{N}(0, 100^2)$
	School	$\epsilon^S_{\mathcal{A},A}$	$\epsilon^S_{\mathcal{A},A} \sim \text{N}(0, 100^2)$
	Others	$\epsilon^O_{\mathcal{A},A}$	$\epsilon^O_{\mathcal{A},A} \sim \text{N}(0, 100^2)$
Proclivity parameter	All	$\lambda^L_{a_i,\alpha}$	$\log \lambda^L_{a_i,\alpha} = \sum_{\mathcal{A},A:\mathcal{A},A \in \mathcal{N}_{a_i,\alpha}} \frac{\epsilon^L_{\mathcal{A},A}}{\lvert\mathcal{N}_{a_i,\alpha}\rvert}$

Table 2: **Prior densities of the parameters and hyperparameters.** The Gamma distribution is parameterised by its shape and rate, Exponential by its rate, and Normal by its mean and variance.

1.3 Posterior distribution of parameters

The posterior distribution is estimated via Markov chain Monte Carlo simulation. The inference was implemented using Just Another Gibbs Sampler (JAGS) within the R statistical environment using 100 000 iterations for the eight POLYMOD countries independently and also collectively as the EU. The model was specified in the JAGS dialect of the BUGS language and later constructed in R via the `rjags` package. With the model, data and initial values properly specified, we initialised the model using the `jags.model` function found in `rjags`.

The convergence of the Markov chain Monte Carlo samplers was assessed using Geweke's convergence diagnostic and the Heidelberger and Welch convergence diagnostic in R. The convergence of the MCMC simulations gave satisfactory convergence diagnostics, with 89% of the parameters passing the convergence tests (median effective sample size 52 000, inter-quartile range 20 000 to 78 000).

We calulated the posterior means for all the $\lambda^L_{a_i,\alpha}$ and δ_L parameters. They are used in the computations of the mean parameters $\mu^L_{a_i,\alpha}$ described in the paper. The estimates of $\mu^L_{a_i,\alpha}$ are displayed in the synthetic contact matrices for 152 countries of the world (**Annex D**).

2 Validation

2.1 Weights for the HAM projection

The household age structure is not known for the 135 ROW countries. In this section, we briefly describe the procedure adopted to calculate the weights needed for the projection the household age matrices (HAM) of these countries. We used a weighted average across POLYMOD/DHS countries, where weights were derived for all the 152 countries using nine indicators to account for differences in the mapping due to developmental and social differences. These indicators include gross domestic product (GDP) per capita, total fertility rate (TFR), population density, population growth rate, under-five mortality rate (U5MR), health expenditure per capita, life expectancy of males, as a proxy for overall health, Internet penetration rate, and urbanisation rate in the country, all standardised by z-scoring. Bootstrap samples of the pairwise distances between countries (between indicators) were computed (Supplementary Figure 3). The population age structure of a country c, P_a^c, should be proportional to $\sum_\alpha P_a^c \; h_{\alpha,a}^c$, where $h_{\alpha,a}^c$ is the HAM of country c. We improved the raw estimates by generating 10 000 bootstrap samples of the pairwise distances between countries (between indicators) as a 'prior' distribution, and selected the combination that maximised the correlation between P_a^c and $\sum_\alpha P_a^c \; h_{\alpha,a}^c$.

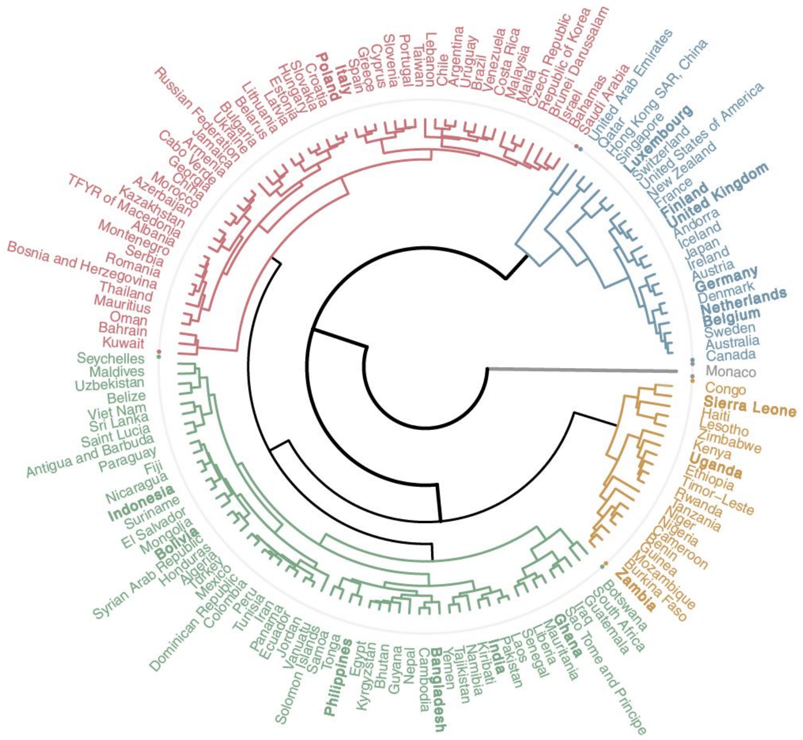

Supplementary Figure 3: Clustering of the 152 countries for the household age matrices projection. The 152 countries were clustered based on pairwise distances of nine socio-demographic indicators. These indicators include gross domestic product (GDP) per capita, total fertility rate (TFR), population density, population growth rate, under-five mortality rate (U5MR), health expenditure per capita, life expectancy of males, as a proxy for overall health, Internet penetration rate, and urbanisation rate in the country, all standardised by z-scoring. The 17 POLYMOD/DHS countries are contrasted with bold fonts.

2.2 HAM Validation for POLYMOD and DHS Countries

Household structure data was made available for 8 POLYMOD (Belgium, Germany, Finland, the United Kingdom, Italy, Luxembourg, the Netherlands, and Poland)[1] and 9 DHS countries (Bangladesh, Bolivia, Ghana, Indonesia, India, the Philippines, Sierra Leone, Uganda and Zam-

[1] Mossong J, Hens N, Jit M, Beutels P, Auranen K, Mikolajczyk R, et al. Social Contacts and Mixing Patterns Relevant to the Spread of Infectious Diseases. PLoS Med. 2008;5: e74. doi:10.1371/journal.pmed.0050074

bia)[2]. In the model, household age matrix (HAM) is needed to estimate the age-specific contacts at home. While this can be easily computed for the 17 POLYMOD and DHS countries, it is not straightforward for the rest of the world (ROW countries). In the main paper, we highlight the methods we developed to estimate the HAM of the ROW countries which is the weighted mean of the population ratio matrices of the 17 POLYMOD/DHS countries.

Leave-one-out validation was performed to reconstruct the HAM of one POLYMOD or DHS country at a time. This involved reverse-engineering that country's household age structure by treating it as if it were unknown and comparing against the actual structure to assess the method's performance.

The plots in **Annex A** show the empirical HAM (i.e. from the data) in the first panel and the modelled HAM (i.e. assuming it is unknown like an ROW country) for the POLYMOD and DHS countries in the second panel. In the third panel, a scatter plot of the entries in the observed and modelled HAM. The empirical HAM for the POLYMOD and DHS countries could be reconstructed with high fidelity (median correlation between inferred and empirical 0.93, interquartile range 0.91–0.95). The reverse engineered contact patterns for countries we omit from the model building and keep for validation show a very close correspondence between model and empirical estimates.

[2]The DHS Program. Available: http://www.dhsprogram.com/Data/

2.3 POLYMOD Validation

In this study, individual-level contact data were available for the 8 POLYMOD countries, allowing us to validate the model's performance. We validated (i) the Bayesian hierarchical model and (ii) the projection procedure with the POLYMOD contact data. In the second step, we treated POLYMOD countries as ROW countries where contact data and household age structure were not made available. The household age matrices, working and school populations were then projected and the location-specific contacts inferred for the POLYMOD countries using the ROW procedure.

The mean location-specific contact rates by age of individuals were computed from the POLYMOD contact data and compared to the estimates derived from (ii) the Bayesian hierarchical model described in section 1.1 (blue dots in the Supplementary Figures 4–7), and (iii) projection if the POLYMOD countries were ROW countries (red triangles in the Supplementary Figures 4–7).

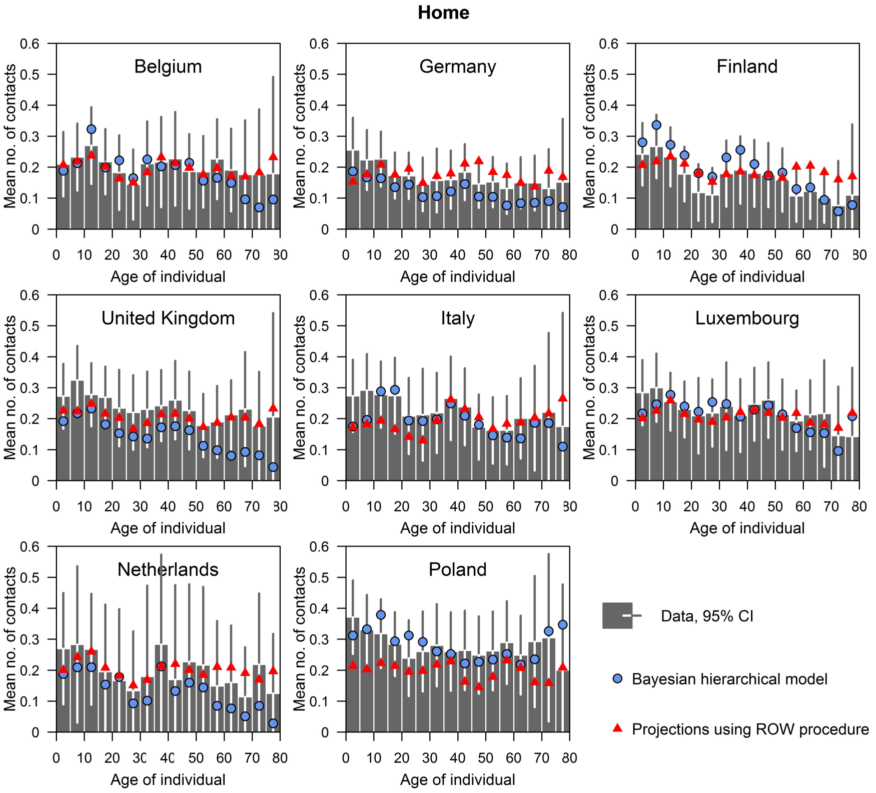

Supplementary Figure 4: Validation of the mean contacts at home for the 8 POLYMOD countries. The distribution of the mean of contact rates made at home by age of individuals were computed from the POLYMOD contact data (grey bars), with accompanying 95% confidence intervals. The mean estimates from the Bayesian hierarchical model (blue dots) and the projections using ROW-procedure (red triangles) are compared the data.

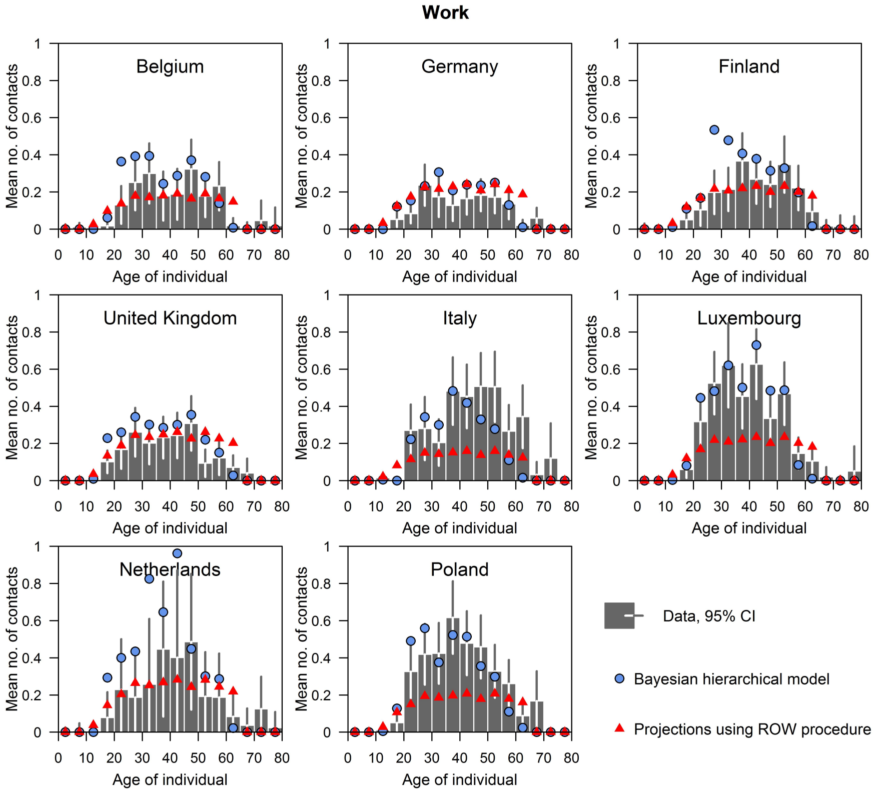

Supplementary Figure 5: Validation of the mean contacts at work for the 8 POLYMOD countries. The distribution of the mean of contact rates made at work by age of individuals were computed from the POLYMOD contact data (grey bars), with accompanying 95% confidence intervals. The mean estimates from the Bayesian hierarchical model (blue dots) and the projections using ROW-procedure (red triangles) are compared the data.

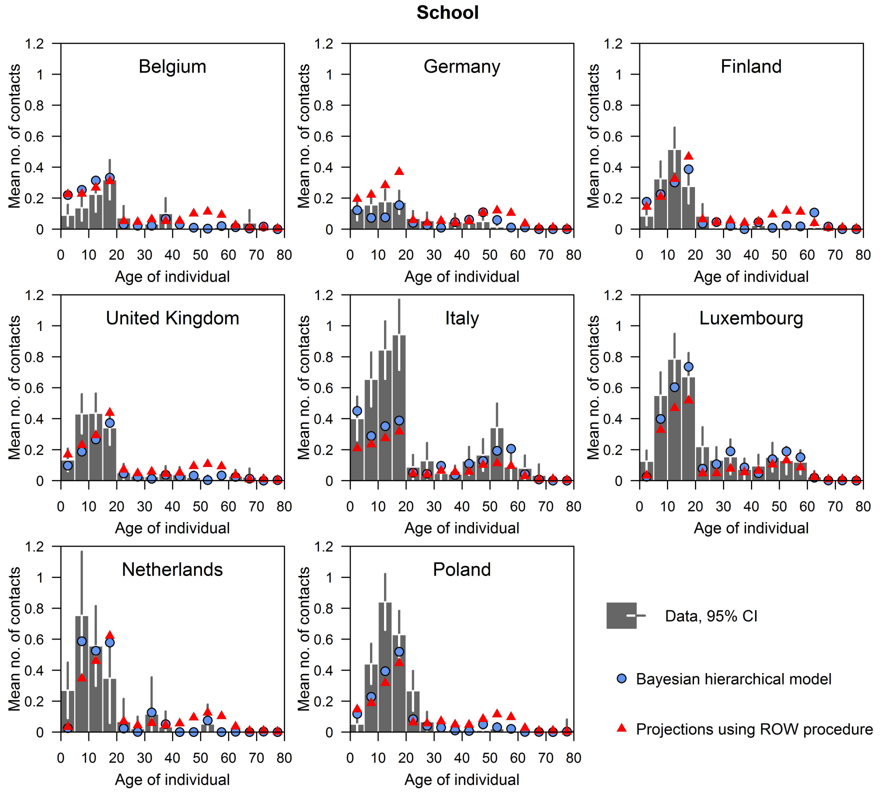

Supplementary Figure 6: Validation of the mean contacts at school for the 8 POLYMOD countries. The distribution of the mean of contact rates made at school by age of individuals were computed from the POLYMOD contact data (grey bars), with accompanying 95% confidence intervals. The mean estimates from the Bayesian hierarchical model (blue dots) and the projections using ROW-procedure (red triangles) are compared the data.

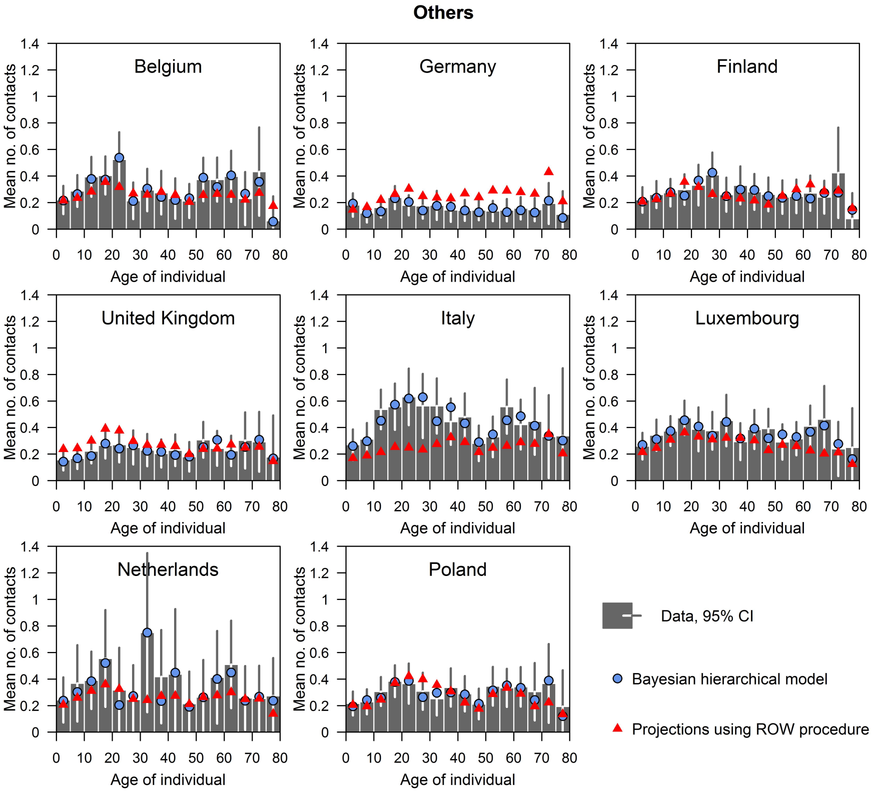

Supplementary Figure 7: Validation of the mean contacts at other locations for the 8 POLYMOD countries. The distribution of the mean of contact rates made at other locations by age of individuals were computed from the POLYMOD contact data (grey bars), with accompanying 95% confidence intervals. The mean estimates from the Bayesian hierarchical model (blue dots) and the projections using ROW-procedure (red triangles) are compared the data.

2.4 External Validation

Projected contact matrices were determined using the methods described in the main paper. They were externally validated against the estimated contact matrices from empirical contact surveys conducted in five low-to-middle-incomed countries (Kenya[3], Peru[4], Russia[5], South Africa[6], and Viet Nam[7]).

Numerical estimates or data were available for four of the five countries (all but Peru). For Peru, we digitized age-specific contact matrices (aggregated over location) and to get the estimated contact matrices. As age- and location-specific contact rates were available for Russia, we were able to validate our age- and location-specific estimates with theirs. For both the survey and our projected estimates, we computed the mean number of location-specific contacts across the age of the individuals. For the other countries where only age-specific contact rates aggregated over all locations, we could only compare our projected matrices aggregated over location. We compared the mean number of location-specific contacts across the age of the individuals.

The survey and projected contact matrices generally have close correspondence. The constraints of small sample sizes when stratified by age lead to relatively large standard errors and more of a discrepancy in the Kenyan and Vietnamese studies. In general, the contact matrices are highly dependent on the population age structure which varies substantially between countries. The correlation between the survery estimates and our projections were 0.83 for Kenya, 0.51 for Peru, 0.76 for Viet Nam, 0.76 for Russia and 0.90 for South Africa.

For two studies, there is a marked discrepancy between the projections and estimates, i.e. Kenya and Peru. In those two studies in particular, the sample sizes are fairly small and the methodology used differed substantially from POLYMOD (e.g. in the Peruvian study, only households

[3]Kiti MC, Kinyanjui TM, Koech DC, Munywoki PK, Medley GF, Nokes DJ. Quantifying Age-Related Rates of Social Contact Using Diaries in a Rural Coastal Population of Kenya. PLoS ONE. 2014;9: e104786.

[4]Grijalva CG, Goeyvaerts N, Verastegui H, Edwards KM, Gil AI, Lanata CF, et al. A Household-Based Study of Contact Networks Relevant for the Spread of Infectious Diseases in the Highlands of Peru. PLOS ONE. 2015;10: e0118457.

[5]Ajelli M, Litvinova M. Estimating contact patterns relevant to the spread of infectious diseases in Russia. J Theor Biol. 2017;419: 1-7.

[6]Johnstone-Robertson SP, Mark D, Morrow C, Middelkoop K, Chiswell M, Aquino LDH, et al. Social Mixing Patterns Within a South African Township Community: Implications for Respiratory Disease Transmission and Control. Am J Epidemiol. 2011;174: 1246-1255.

[7]Horby P, Thai PQ, Hens N, Yen NTT, Mai LQ, Thoang DD, et al. Social Contact Patterns in Vietnam and Implications for the Control of Infectious Diseases. PLoS ONE.

with children were recruited and respondents were directly interviewed by field workers; while in the Kenyan study participants <10y or who were illiterate were 'shadowed' by another person who recorded their contacts), while the settings do not fully reflect the country as a whole: the Peruvian study was conducted in the remote highlands of Peru which may not be representative of Peruvian contact patterns in general, while the Kenyan study was conducted in rural and semi-urban coastal communities.

2.5 Outlying Countries

In most countries, there is a strong leading diagonal in the projected contact matrices indicating assortivity with the same age group, combined with off-diagonals for a one-generation displacement. It is possible for the social contacts to deviate from the assortative mixing patterns observed in POLYMOD countries. Several countries exemplify this, such as Sierra Leone and Burkina Faso, where we project a deviation from that pattern as a result of the highly skewed population structure (Supplementary Figure 8). In general, the contact matrices are highly dependent on the population age structure which varies substantially between countries.

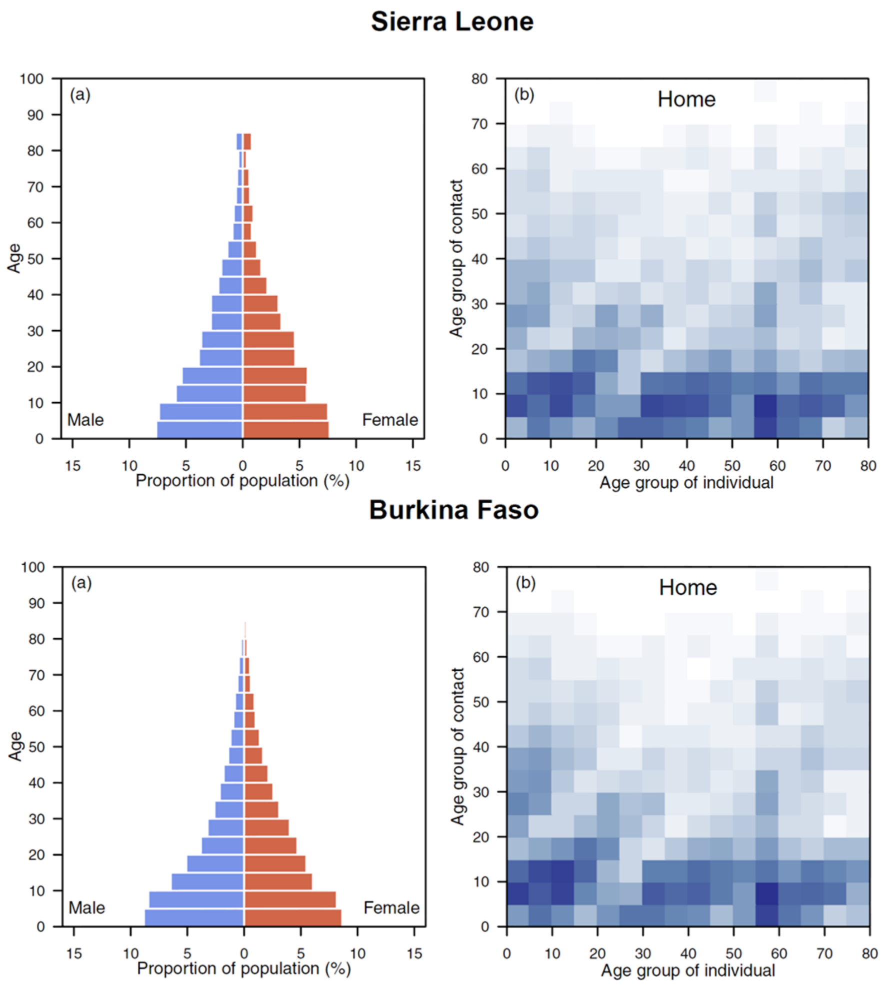

Supplementary Figure 8: Outlying countries. Sierra Leone and Burkina Faso have highly skewed population structures which cause deviation from the primary and secondary diagonals observed we expect to see in age-specific contacts made at home.

2.6 Comparison with Fumanelli et al., 2012

For 25 European countries, the projected contact matrices were compared with contact matrices derived by Fumanelli et al.[8] Virtual populations were constructed using parameters from census and demographic data by Fumanelli et al. to estimate contact matrices.

For contacts made at workplaces and school, the contact matrices from the present study and that of Fumanelli et al. generally have close correspondence, although differences can be discerned between the two home contact matrices and the two contact matrices at other locations. However, for countries in both POLYMOD and the Fumanelli study, the synthetic contact matrices presented in this paper match more closely the empirical age profile from POLYMOD than do Fumanelli et al's estimates (see seven POLYMOD countries in **Annex C**).

[8]Fumanelli L, Ajelli M, Manfredi P, Vespignani A, Merler S. Inferring the Structure of Social Contacts from Demographic Data in the Analysis of Infectious Diseases Spread. PLoS Comput Biol. 2012;8: e1002673.

3 Application of Contact Matrices

3.1 Age-specific SIR Modelling

Deterministic Susceptible-Infected-Removed (SIR) age-specific epidemic models were built to project numerically the age-specific final attack size and the proportionate reduction in infection for countries of different levels of development and at various levels of intervention: (i) No intervention, (ii) School closure, (iii) School closure and social distancing, and (iv) Workplace distancing. We considered two pandemic influenza scenarios ($R_0 = 1.2$ and 1.5) and the derived age-specific contact matrices informed the epidemic models. The age-specific final epidemic size and the percent reduction in infection were obtained by scaling the contact matrices, $\mu_{a,\alpha}$, to obtain the scenario's-specified R_0, setting the removal rate without loss of generality to be one. We initialised the SIR model with starting immunity levels derived from age-specific susceptibility data from the study by Miller et al. (2010)[9]. The age-specific SIR model with contact matrices[10] incorporated is given by the system of ordinary differential equations:

$$
\begin{aligned}
\frac{dS_a}{dt} &= -\beta\ S_a \sum_{\alpha=1}^{16} \mu_{a,\alpha} \frac{I_\alpha}{N_\alpha} \\
\frac{dI_a}{dt} &= \beta\ S_a \sum_{\alpha=1}^{16} \mu_{a,\alpha} \frac{I_\alpha}{N_\alpha} - \gamma I_a \\
\frac{dR_a}{dt} &= \gamma I_a
\end{aligned}
$$

where a and α are the age groups, β is the probability of transmission given a contact, and γ is the rate of recovery; both β and $\gamma$are age-invariant in this simple application. The population in age group α is N_α. The contact matrix, $\mu_{a,\alpha}$, is a linear combination of age-specific contacts at home, work, school and other locations scaled depending on the intervention. The system of ordinary differential equations numerically solved using the `deSolve` package in `R`.

Scenario 1: No intervention

Total contacts were calculated as the unweighted sum of contacts made at home, work, school and other location:

$$\mu_{a,\alpha} = \mu^H_{a,\alpha} + \mu^W_{a,\alpha} + \mu^S_{a,\alpha} + \mu^O_{a,\alpha}.$$

[9]Miller E, Hoschler K, Hardelid P, Stanford E, Andrews N, Zambon M. Incidence of 2009 pandemic influenza A H1N1 infection in England: a cross-sectional serological study. The Lancet. 2010;375: 1100-1108.

[10]Towers S, Feng Z. Social contact patterns and control strategies for influenza in the elderly. Math Biosci. 2012;240: 241-249.

Scenario 2: School closure

The contribution from school contacts to the contact matrix was set to zero:

$$\mu_{a,\alpha} = \mu^H_{a,\alpha} + \mu^W_{a,\alpha} + 0 \cdot \mu^S_{a,\alpha} + \mu^O_{a,\alpha}.$$

Scenario 3: School closure and social distancing

The contribution from school contacts was set to zero together with a 50% reduction in the contribution of contacts made at other locations between school-going individuals (below 20 years i.e. the first four age groups), and a 50% inrease in the contribution of contacts made at home between school-going individuals to the contact matrix:

$$\mu_{a,\alpha} = \mathbf{A}\mu^H_{a,\alpha} + \mu^W_{a,\alpha} + 0 \cdot \mu^S_{a,\alpha} + \mathbf{B}\ \mu^O_{a,\alpha}$$

where **A** and **B** are the diagonal matrices

$$\mathbf{A} = \begin{bmatrix} 1.5 & 0 & 0 & 0 & 0 & \cdots & 0 \\ 0 & 1.5 & 0 & 0 & 0 & \cdots & 0 \\ 0 & 0 & 1.5 & 0 & 0 & \cdots & 0 \\ 0 & 0 & 0 & 1.5 & 0 & \cdots & 0 \\ 0 & 0 & 0 & 0 & 1.1 & \cdots & 0 \\ \vdots & \vdots & \vdots & \vdots & \vdots & \ddots & \vdots \\ 0 & 0 & 0 & 0 & 0 & \cdots & 1.1 \end{bmatrix}$$

and

$$\mathbf{B} = \begin{bmatrix} 0.5 & 0 & 0 & 0 & 0 & \cdots & 0 \\ 0 & 0.5 & 0 & 0 & 0 & \cdots & 0 \\ 0 & 0 & 0.5 & 0 & 0 & \cdots & 0 \\ 0 & 0 & 0 & 0.5 & 0 & \cdots & 0 \\ 0 & 0 & 0 & 0 & 1 & \cdots & 0 \\ \vdots & \vdots & \vdots & \vdots & \vdots & \ddots & \vdots \\ 0 & 0 & 0 & 0 & 0 & \cdots & 1 \end{bmatrix}.$$

Scenario 4: Workplace distancing

We reduced the contribution of contacts made at the workplace by 50% to the contact matrix:

$$\mu_{a,\alpha} = \mu^H_{a,\alpha} + \mathbf{C}\ \mu^W_{a,\alpha} + \mu^S_{a,\alpha} + \mu^O_{a,\alpha}$$

where **C** is the diagonal matrix

$$\mathbf{C} = \begin{bmatrix} 0.5 & 0 & 0 & \cdots & 0 \\ 0 & 0.5 & 0 & \cdots & 0 \\ 0 & 0 & 0.5 & \cdots & 0 \\ \vdots & \vdots & \vdots & \ddots & \vdots \\ 0 & 0 & 0 & \cdots & 0.5 \end{bmatrix}.$$

Annex A

Validation Plots of Household Age Matrices

The empirical household age-structures (i.e. from the data) and the modelled household age-structures (i.e. assuming it is unknown like an ROW country) for each POLYMOD and DHS countries are represented in the first and second panels respectively. In the third panel, a scatter plot of the entries in the observed (x-axis) and modelled (y-axis) HAM. The empirical HAM for the POLYMOD and DHS countries could be reconstructed with high fidelity (median correlation between inferred and empirical 0.93, inter-quartile range 0.91–0.95). The reverse engineered contact patterns for countries we omit from the model building and keep for validation show a very close correspondence between model and empirical estimates.

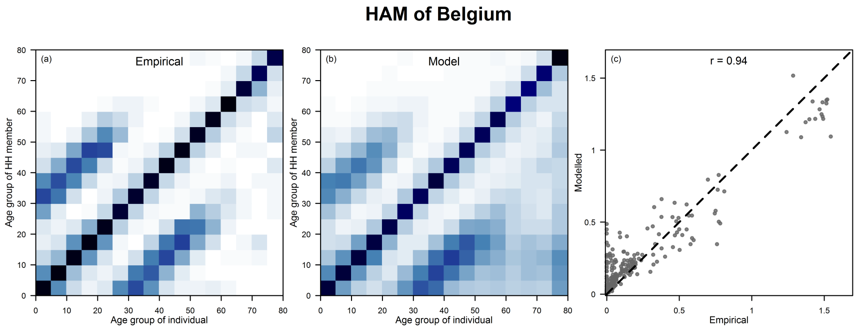

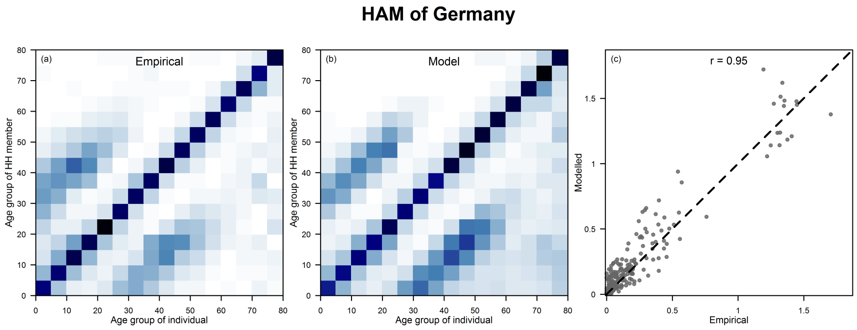

HAM of Finland

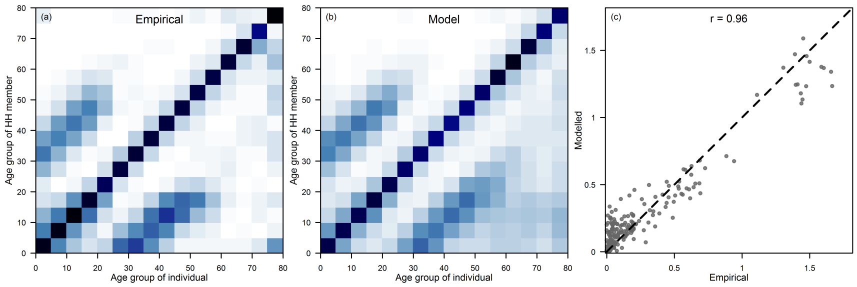

HAM of United Kingdom

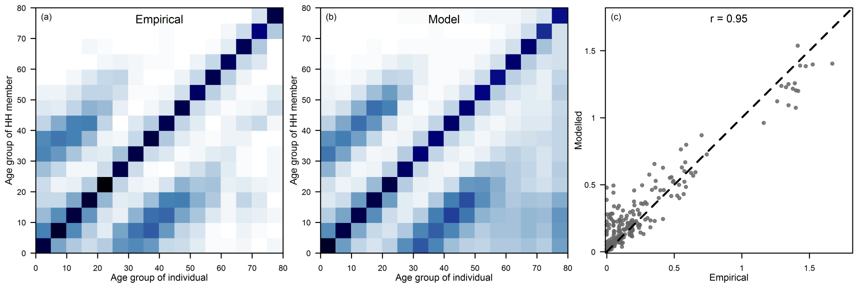

HAM of Italy

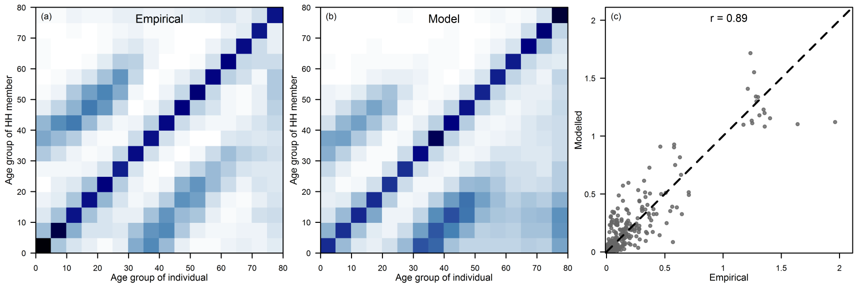

HAM of Luxembourg

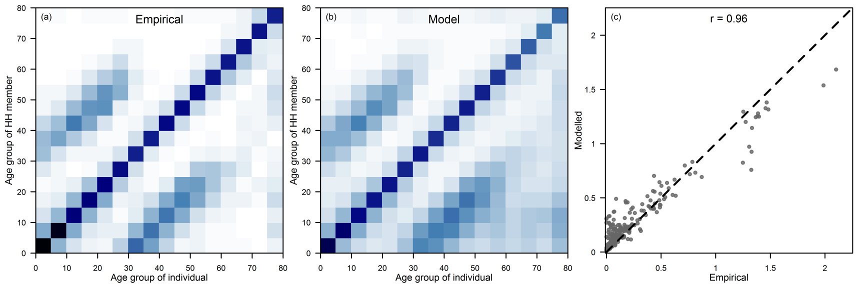

HAM of Netherlands

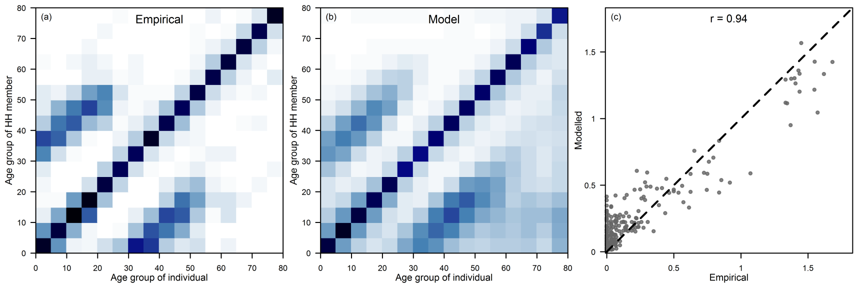

HAM of Poland

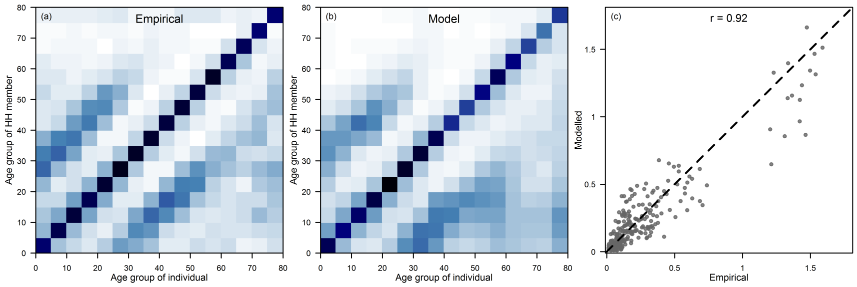

HAM of Bangladesh

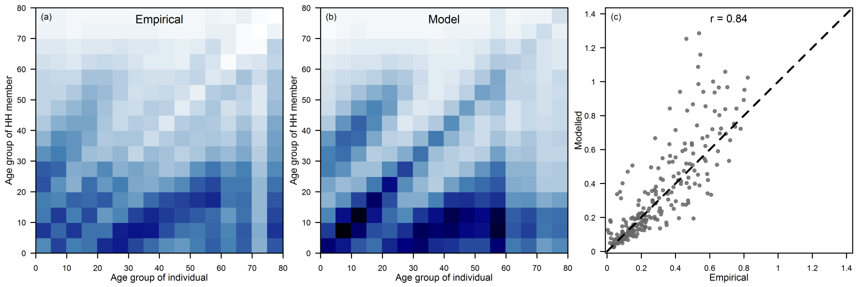

HAM of Bolivia

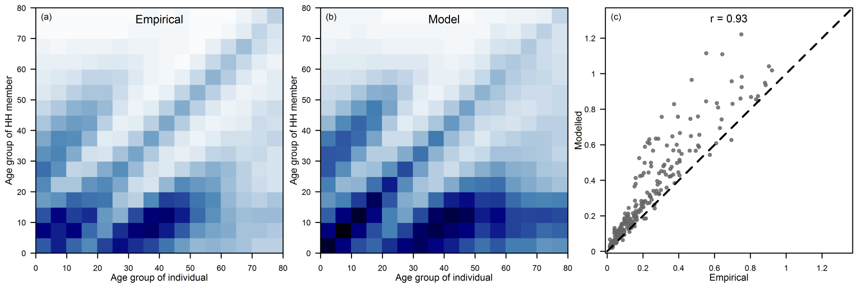

HAM of Ghana

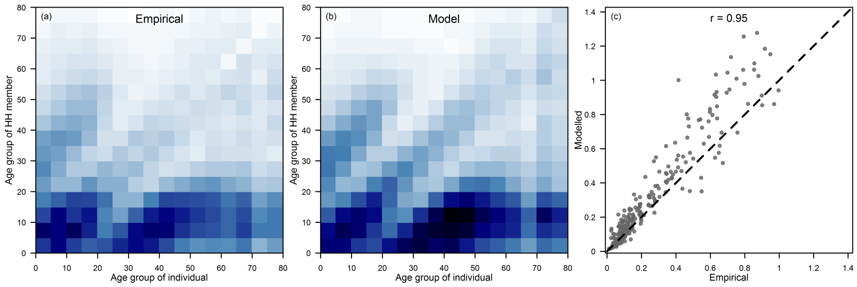

HAM of India

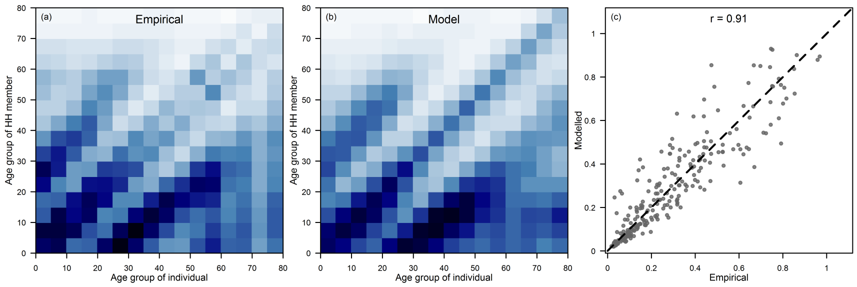

HAM of Indonesia

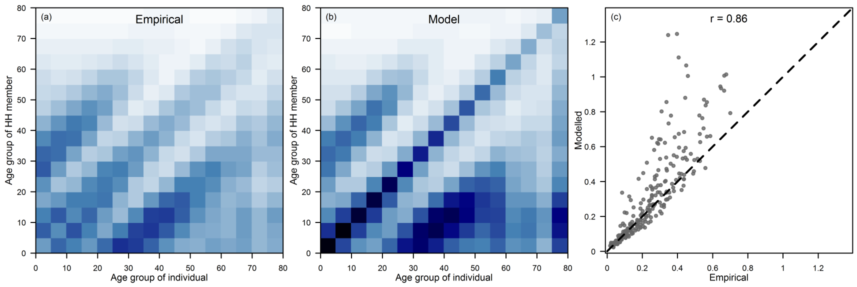

HAM of Philippines

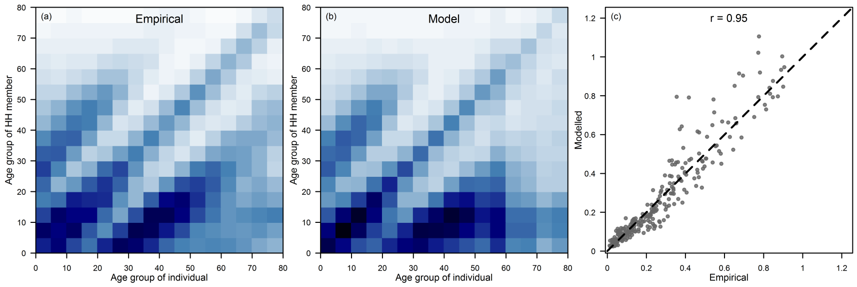

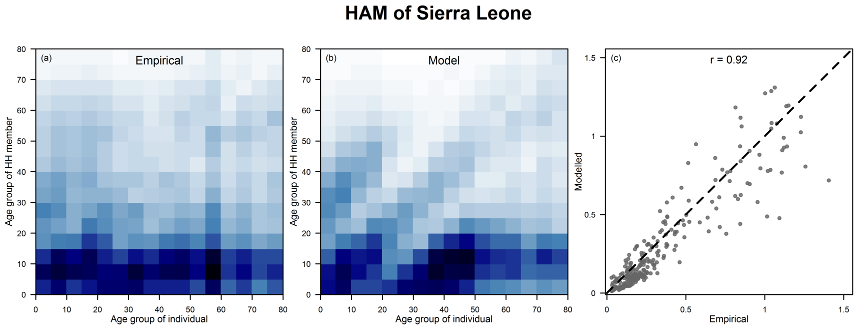
HAM of Sierra Leone
(a) Empirical
(b) Model
(c) r = 0.92
Age group of HH member
Age group of individual
Modelled
Empirical

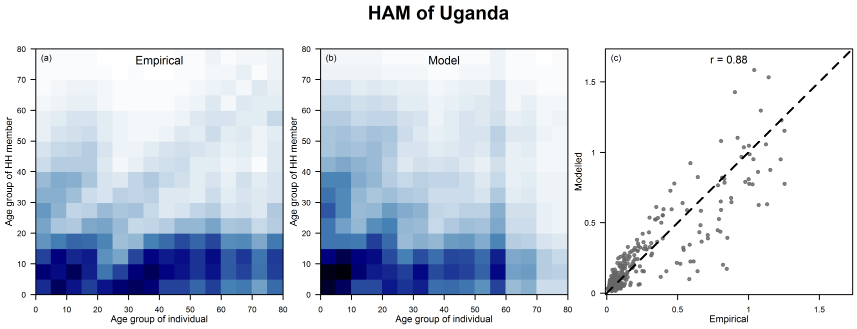
HAM of Uganda
(a) Empirical
(b) Model
(c) r = 0.88
Age group of HH member
Age group of individual
Modelled
Empirical

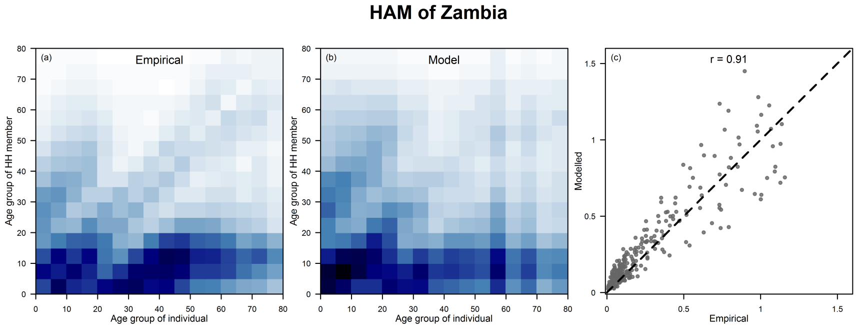
HAM of Zambia
(a) Empirical
(b) Model
(c) r = 0.91
Age group of HH member
Age group of individual
Modelled
Empirical

Annex B

Validation Plots of Contact Matrices

The projected contact patterns were validated against the empirical findings of five countries (Kenya, Peru, Russia, South Africa and Viet Nam).

Annex C

Comparison with Fumanelli et al., 2012

The estimated location and age-specific contact matrices from Fumanelli et al., 2012 and this manuscript are represented in the first and second panels respectively. In the third panel, the empirical location and age-specific contact matrices of seven POLYMOD from Mossong et al., 2008 are plotted.

Annex D

Age and Location-specific Projected Contact Matrices of 152 countries

The population pyramids by age and gender (panel a) and age-specific contact patterns at the various locations namely home, work, school, other and all locations (panels b–f) are presented for all the 152 countries.Age-specific contacts at all locations (panel f) are derived by summing contact rates at home, work, school and other locations. Darker color intensities indicate more likely events i.e. higher proclivity of making the age-specific contact.

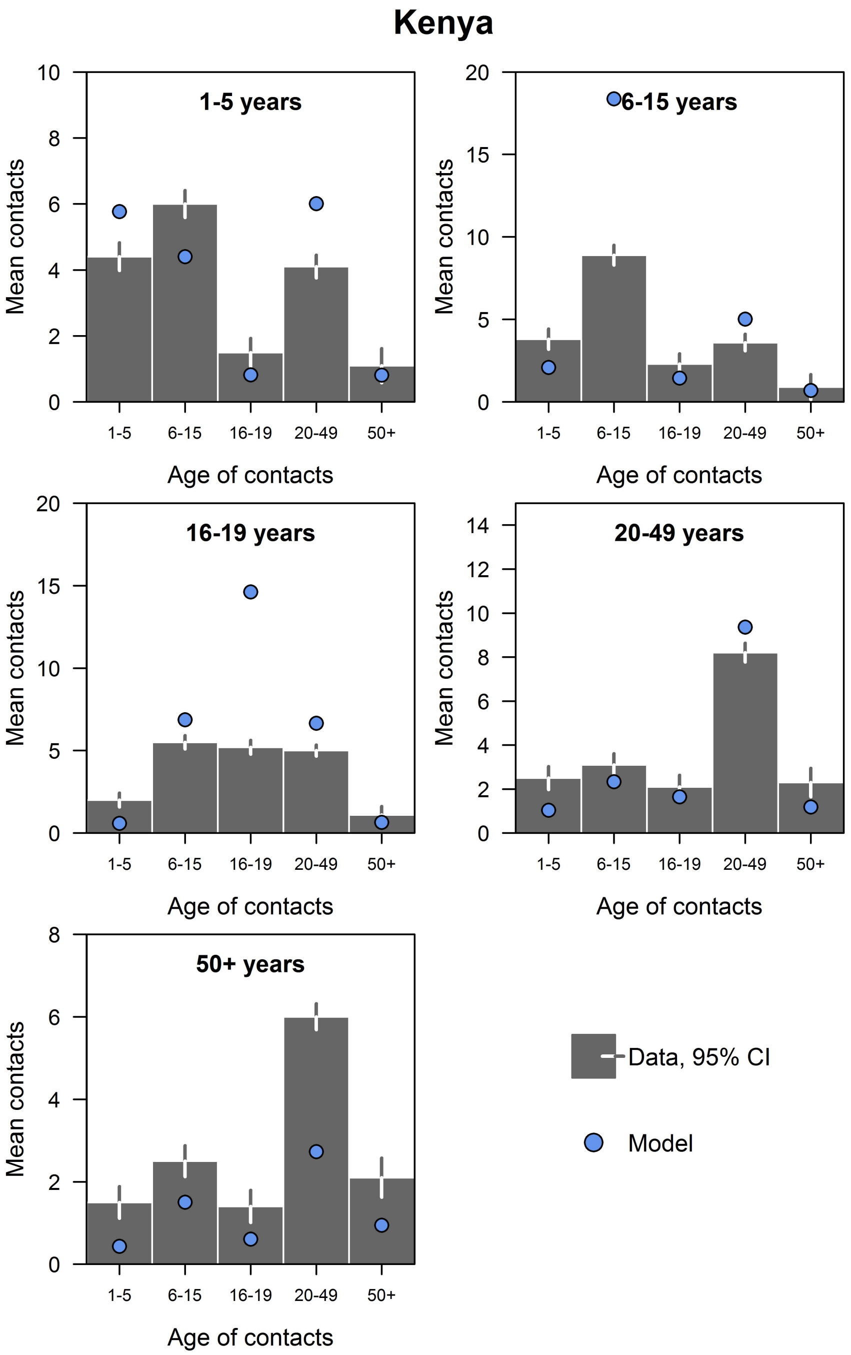

Validation of the projected contact matrices for Kenya. The mean number of contacts by age of individuals from the age-specific contact matrices from the contact survey done in Kenya (grey bars with 95% confidence intervals) to the mean number of contacts computed from our projected age-specific contact matrix (blue dots). Some discrepanies are observed between the emipirical findings and the projections.

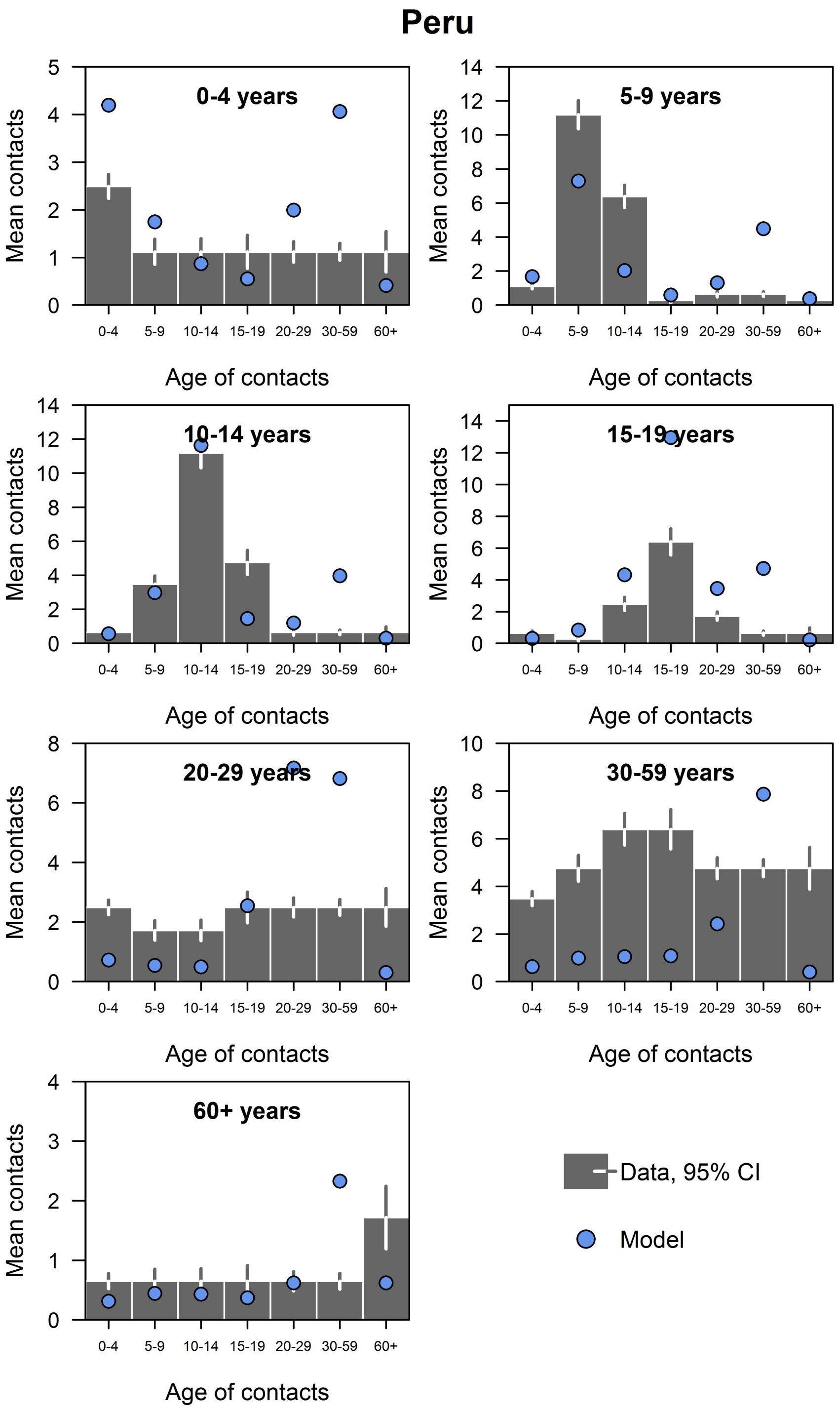

Validation of the projected contact matrices for Peru. The mean number of contacts by age of individuals from the digitised age-specific contact matrices from the contact survey done in the remote highlands of Peru (grey bars with 95% confidence intervals) to the mean number of contacts computed from our projected age-specific contact matrix (blue dots). Discrepanies are observed between the empirical findings and the projections.

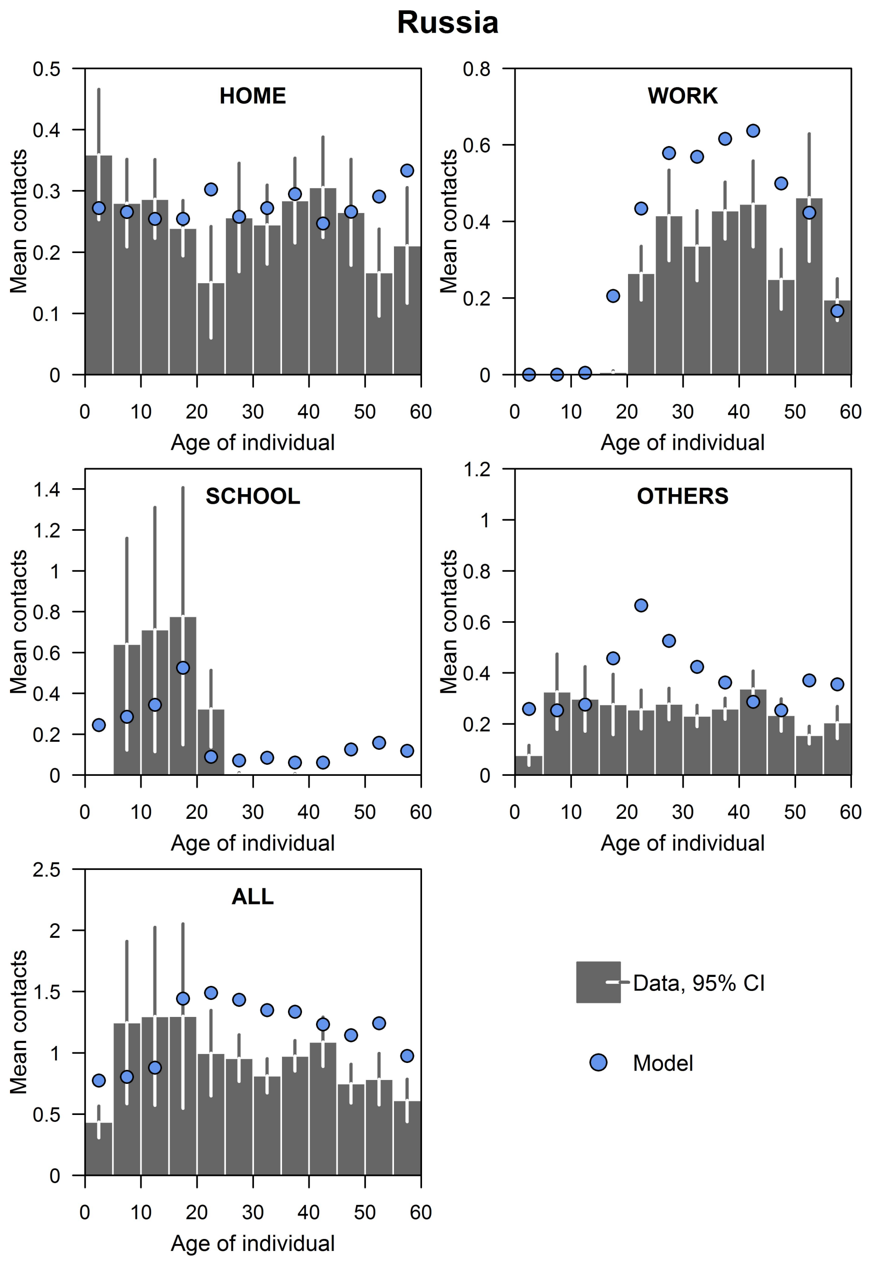

Validation of the projected contact matrices for Russia. The mean number of contacts by age of individuals and location from the contact survey done in Russia (grey bars with 95% confidence intervals) is compared to the mean number of contacts computed from our projected age- and location-specific contact matrix (blue dots). The projected contact matrix developed adequaltely captured the empirical findings in Russia.

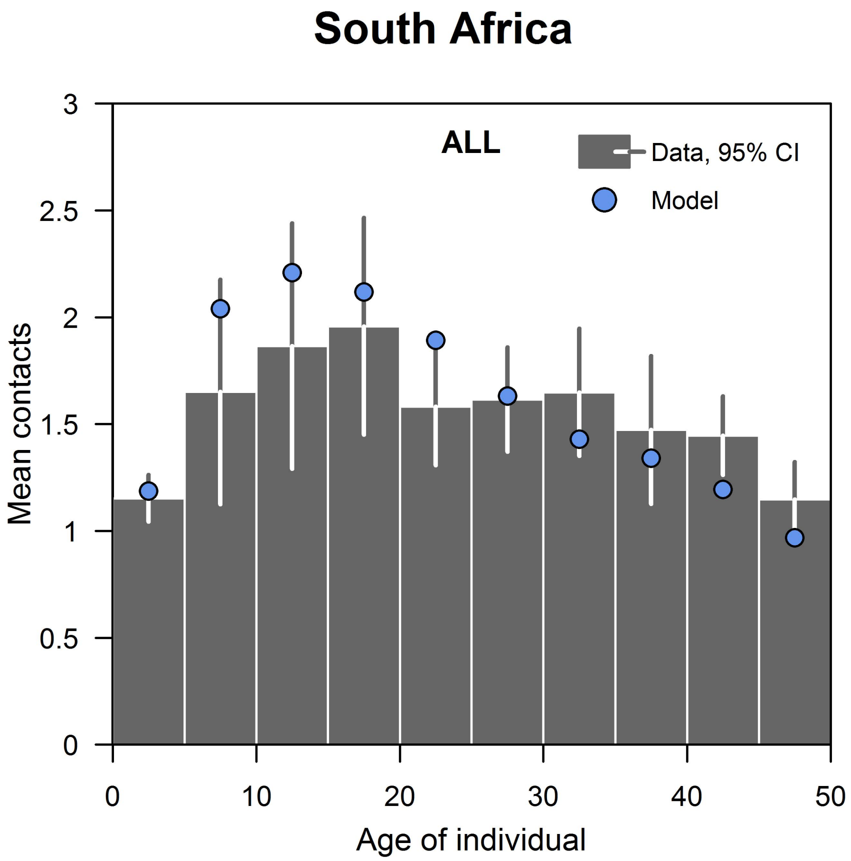

Validation of the projected contact matrices for South Africa. The mean number of contacts by age of individuals from the contact survey done in South Africa (grey bars with 95% confidence intervals) is compared to the mean number of contacts computed from our projected age-specific contact matrix (blue dots). The projected contact matrix developed adequaltely captured the empirical findings in South Africa.

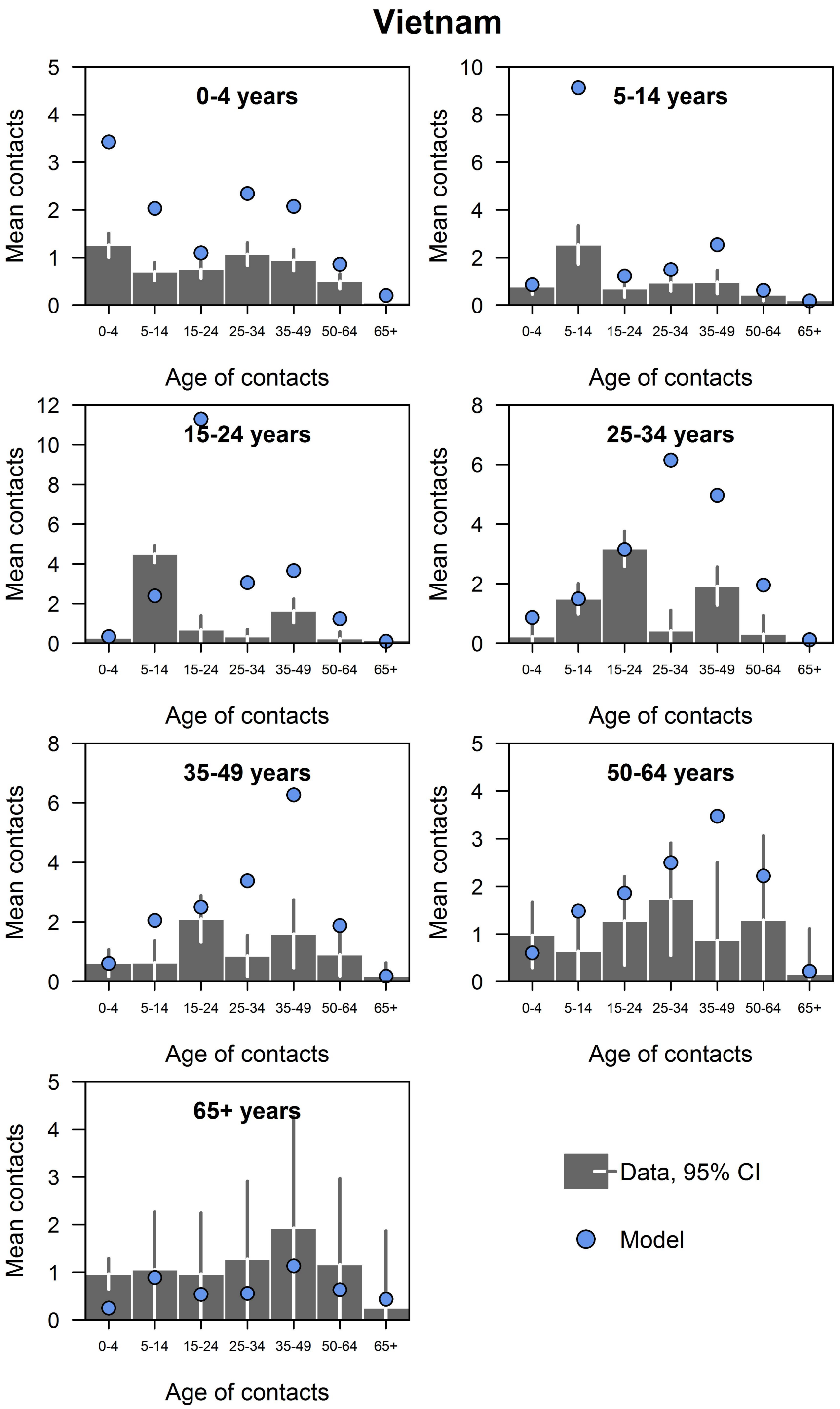

Validation of the projected contact matrices for Viet Nam. The mean number of contacts by age of individuals from the contact survey done in Viet Nam (grey bars with 95% confidence intervals) is compared to the mean number of contacts computed from our projected age-specific contact matrix (blue dots). The projected contact matrix developed adequaltely captured the empirical findings in Viet Nam. 33

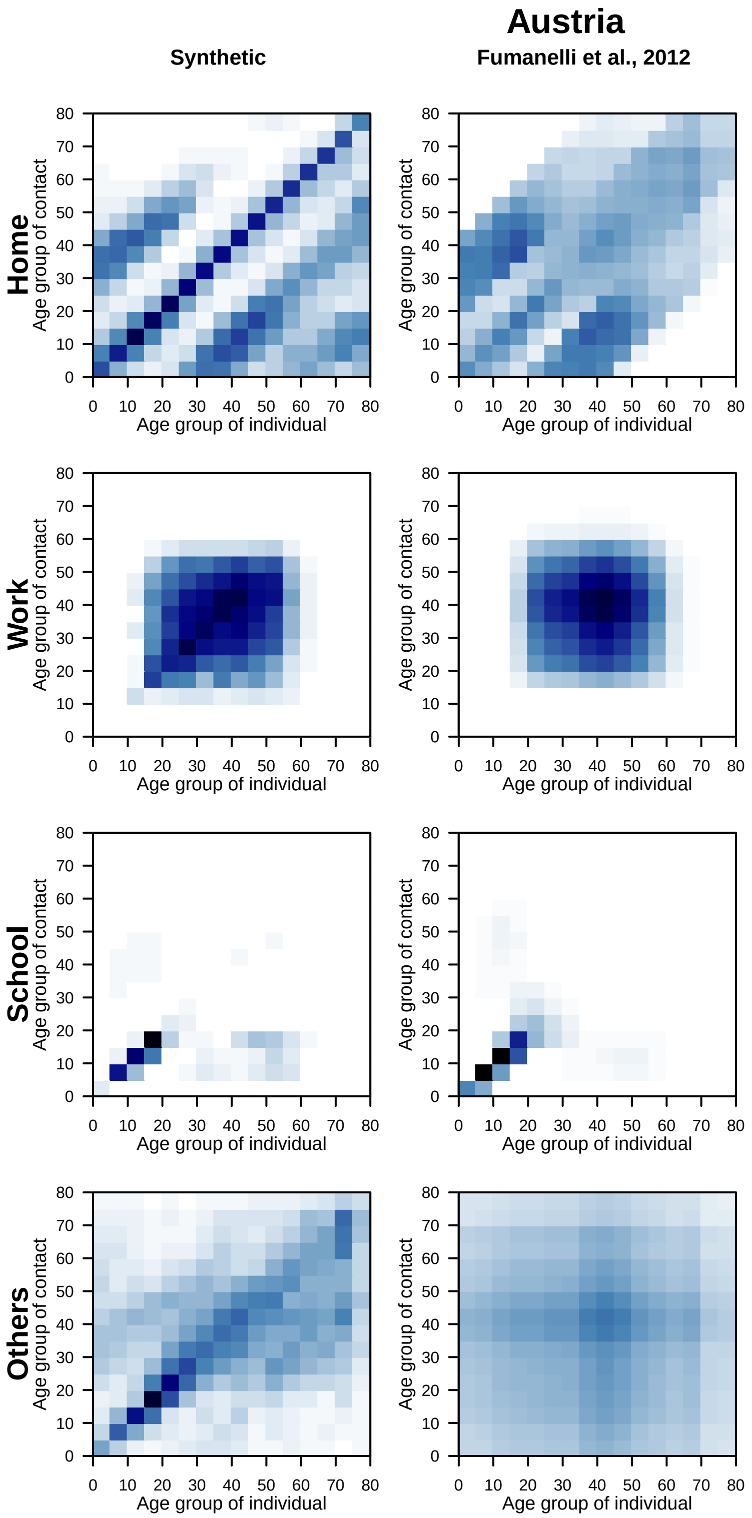
Austria
Synthetic
Fumanelli et al., 2012
Home
Work
School
Others
Age group of contact
Age group of individual
0
10
20
30
40
50
60
70
80

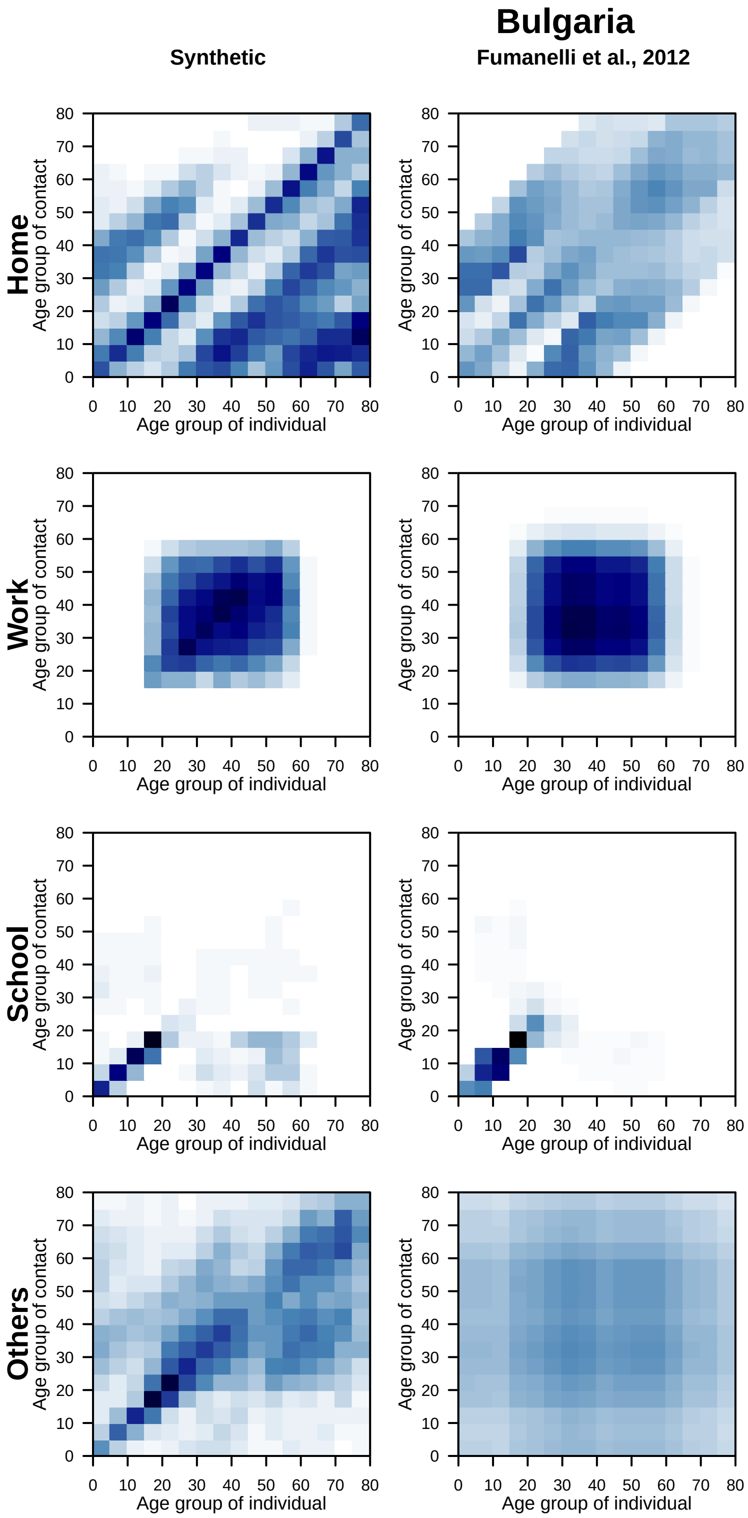
Bulgaria
Synthetic
Fumanelli et al., 2012
Home
Work
School
Others
Age group of contact
Age group of individual
0
10
20
30
40
50
60
70
80

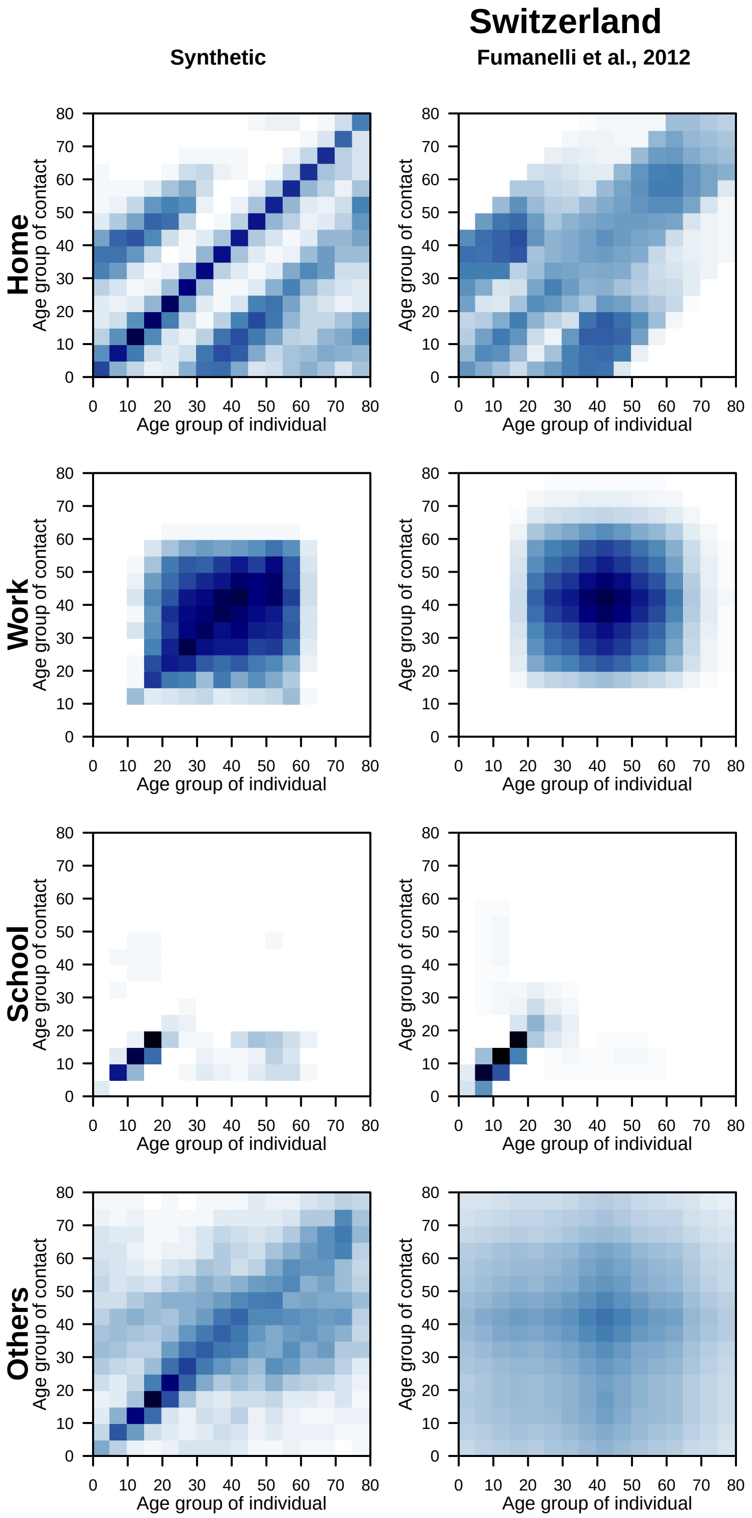
Switzerland
Synthetic
Fumanelli et al., 2012
Home
Work
School
Others
Age group of contact
Age group of individual
0 10 20 30 40 50 60 70 80

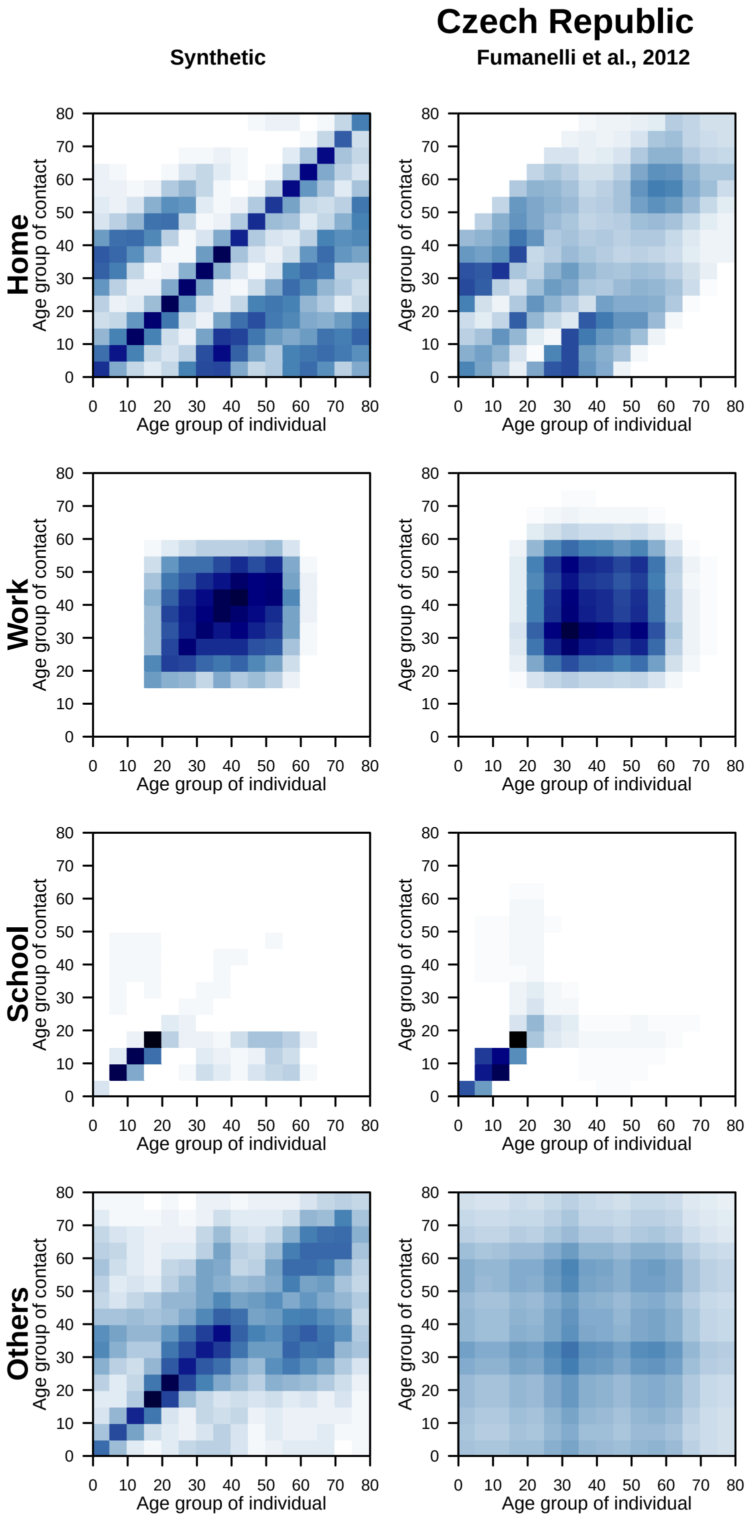
Czech Republic
Synthetic
Fumanelli et al., 2012
Home
Work
School
Others
Age group of contact
Age group of individual
0
10
20
30
40
50
60
70
80

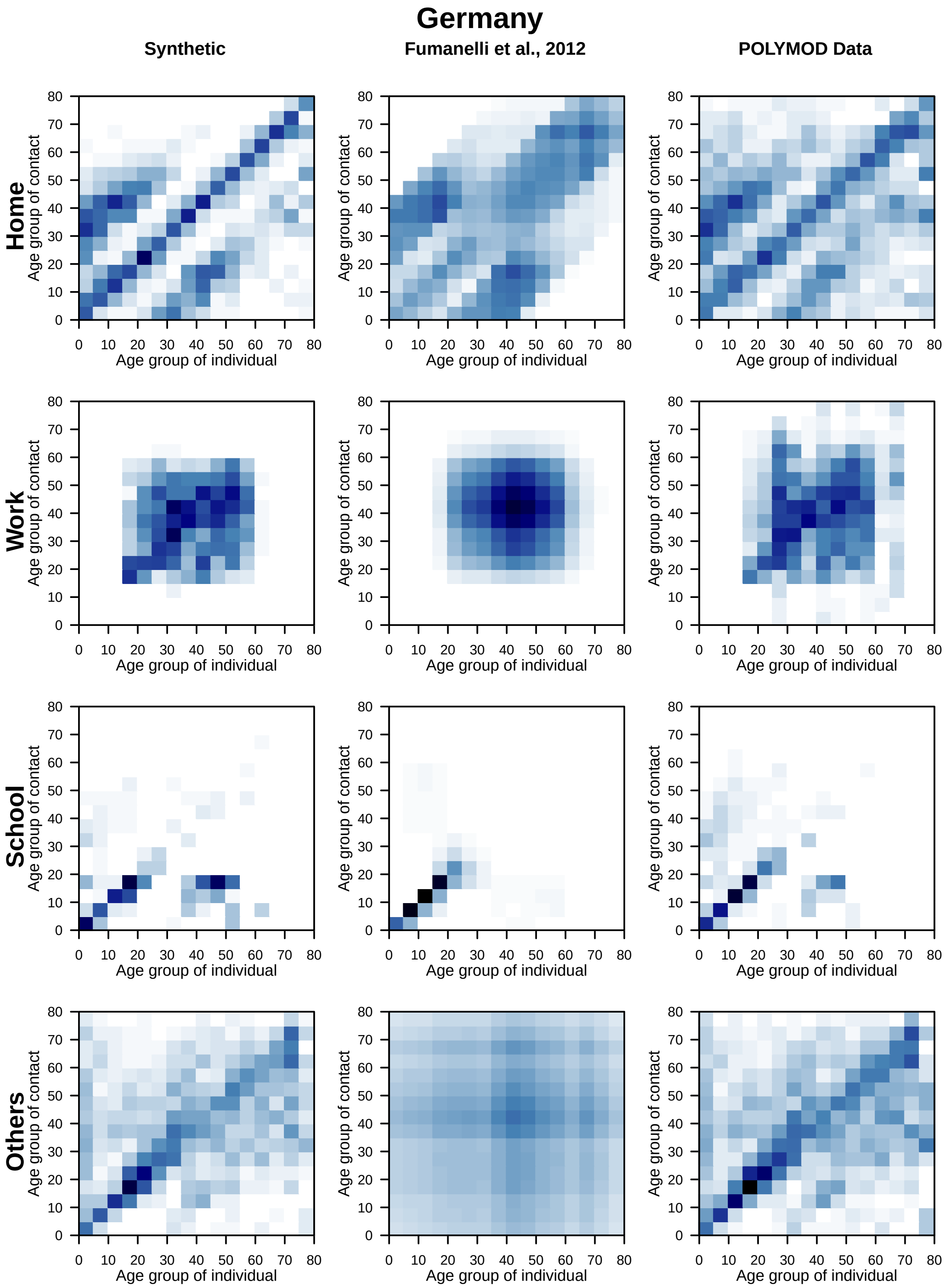

Germany
Synthetic
Fumanelli et al., 2012
POLYMOD Data
Home
Work
School
Others
Age group of contact
Age group of individual
0
10
20
30
40
50
60
70
80

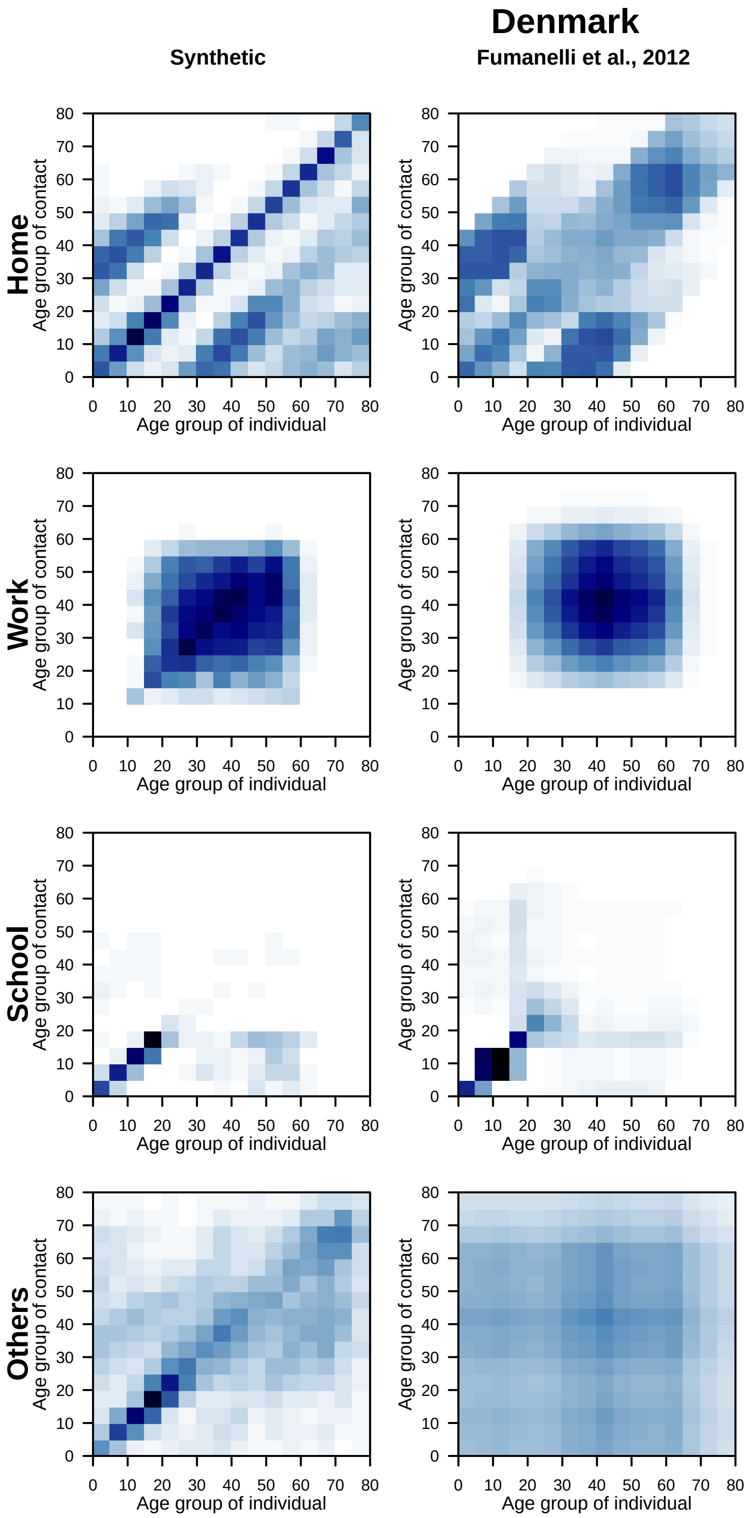

Denmark
Synthetic
Fumanelli et al., 2012
Home
Work
School
Others
Age group of contact
Age group of individual
0
10
20
30
40
50
60
70
80

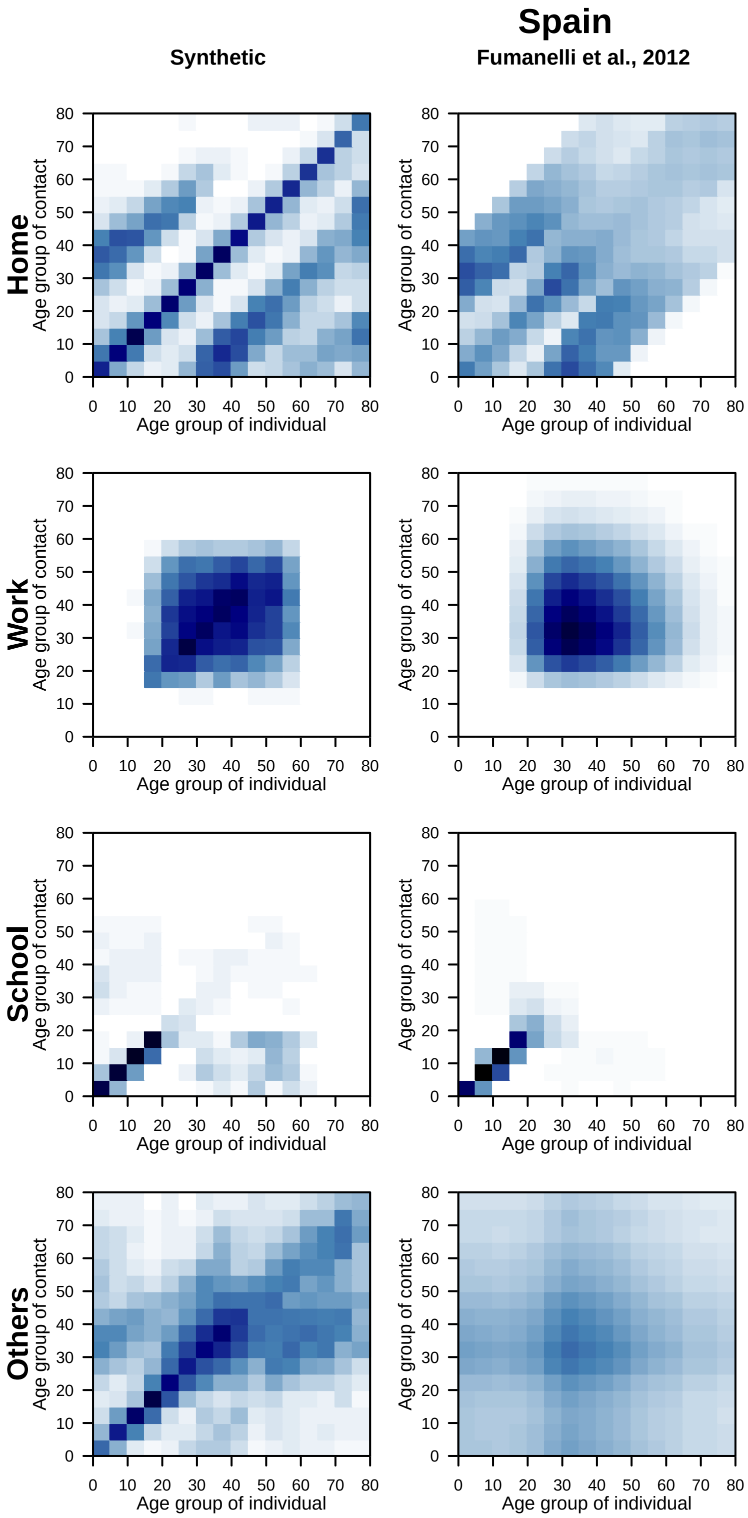
Spain
Synthetic
Fumanelli et al., 2012
Home
Work
School
Others
Age group of contact
Age group of individual
0
10
20
30
40
50
60
70
80

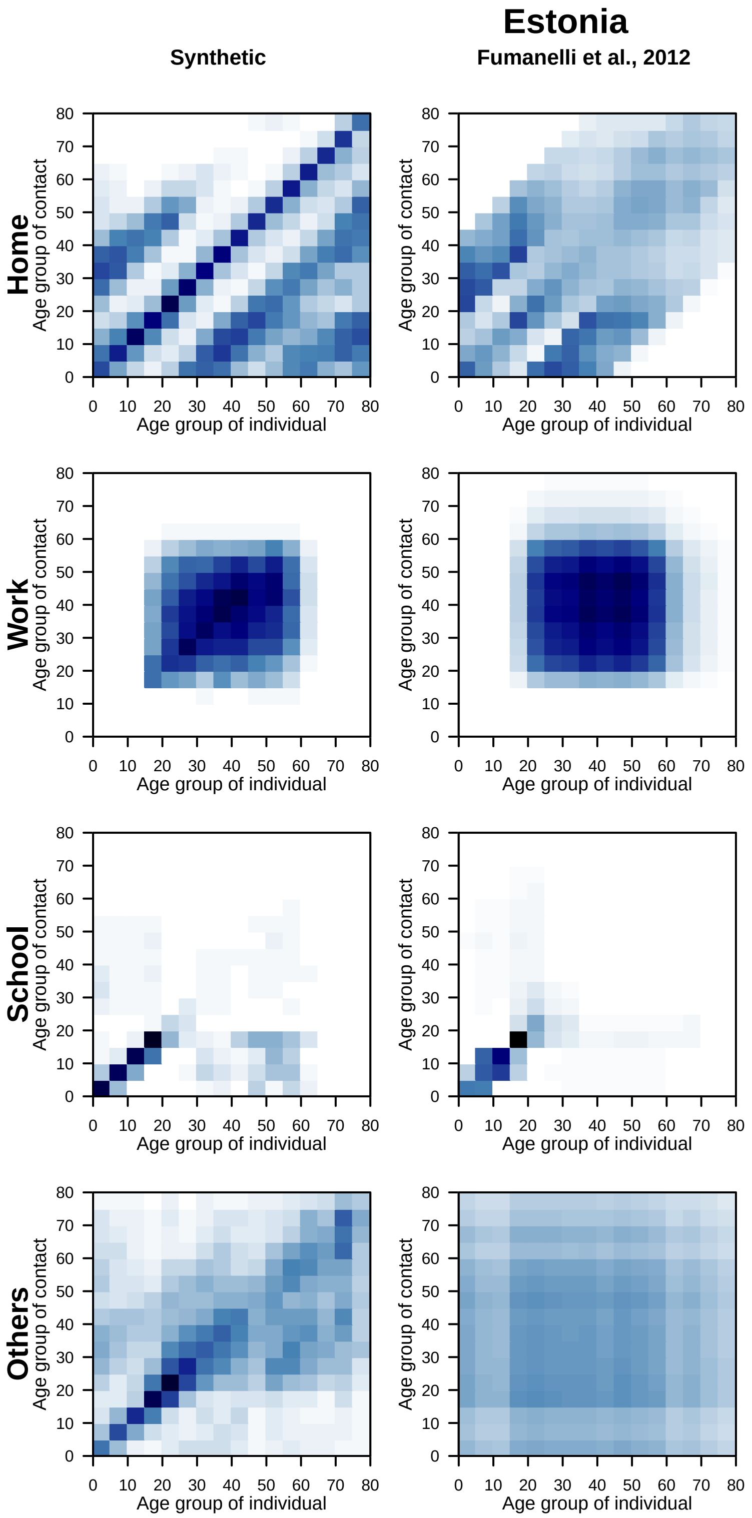
Estonia
Synthetic
Fumanelli et al., 2012
Home
Work
School
Others
Age group of contact
Age group of individual
0
10
20
30
40
50
60
70
80

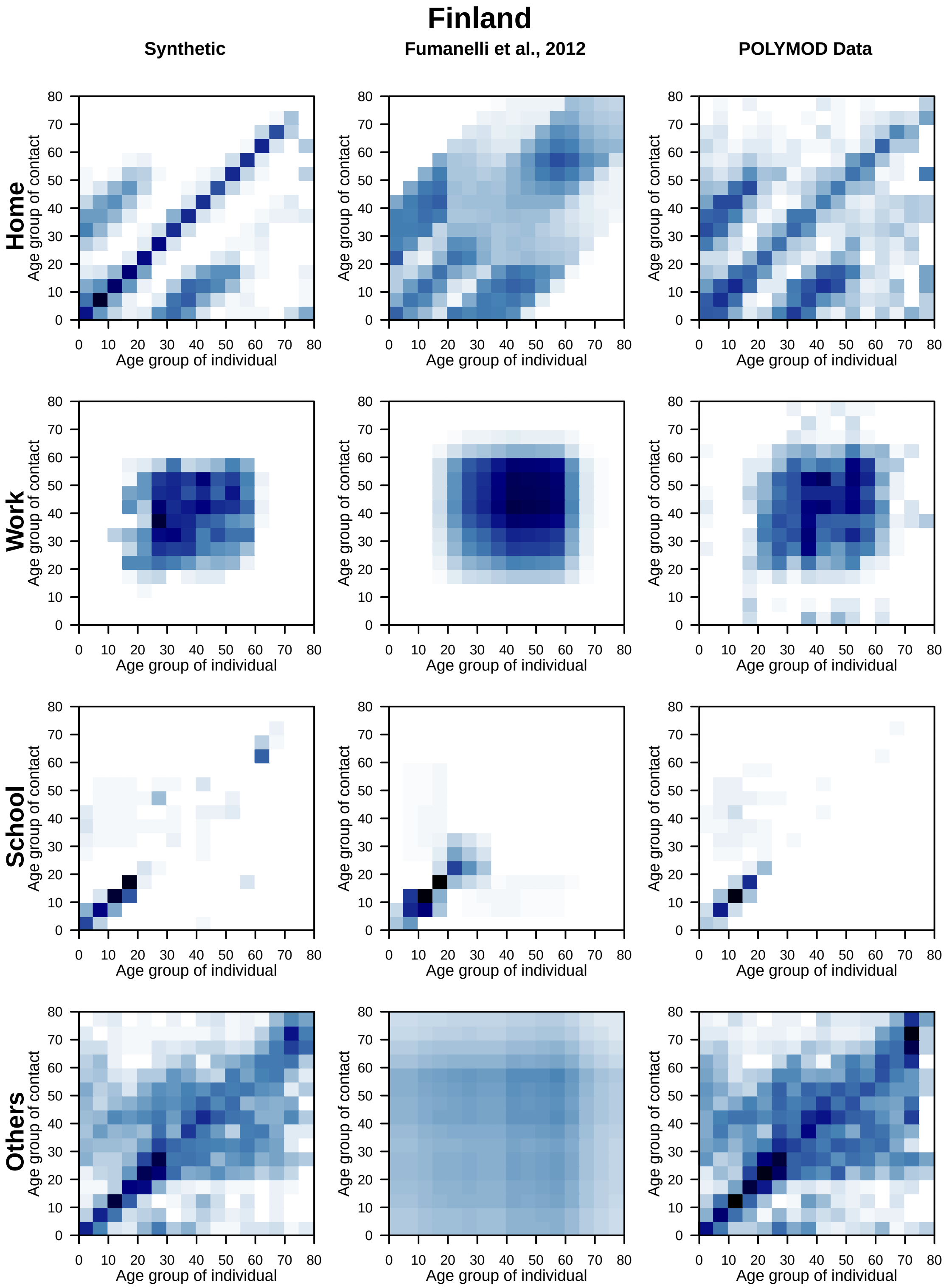
Finland
Synthetic
Fumanelli et al., 2012
POLYMOD Data
Home
Work
School
Others
Age group of contact
Age group of individual
0
10
20
30
40
50
60
70
80

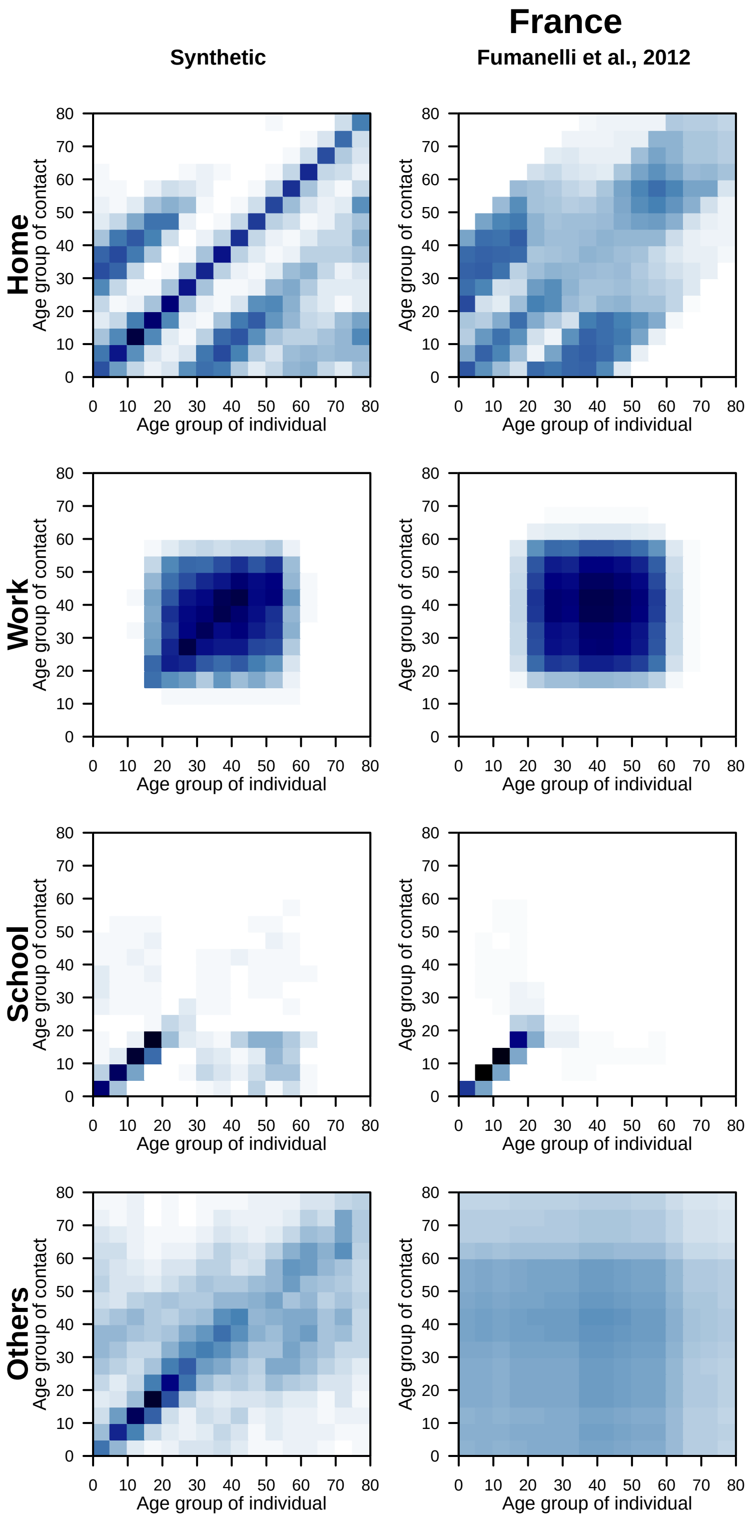
France
Synthetic
Fumanelli et al., 2012
Home
Work
School
Others
Age group of contact
Age group of individual
0
10
20
30
40
50
60
70
80

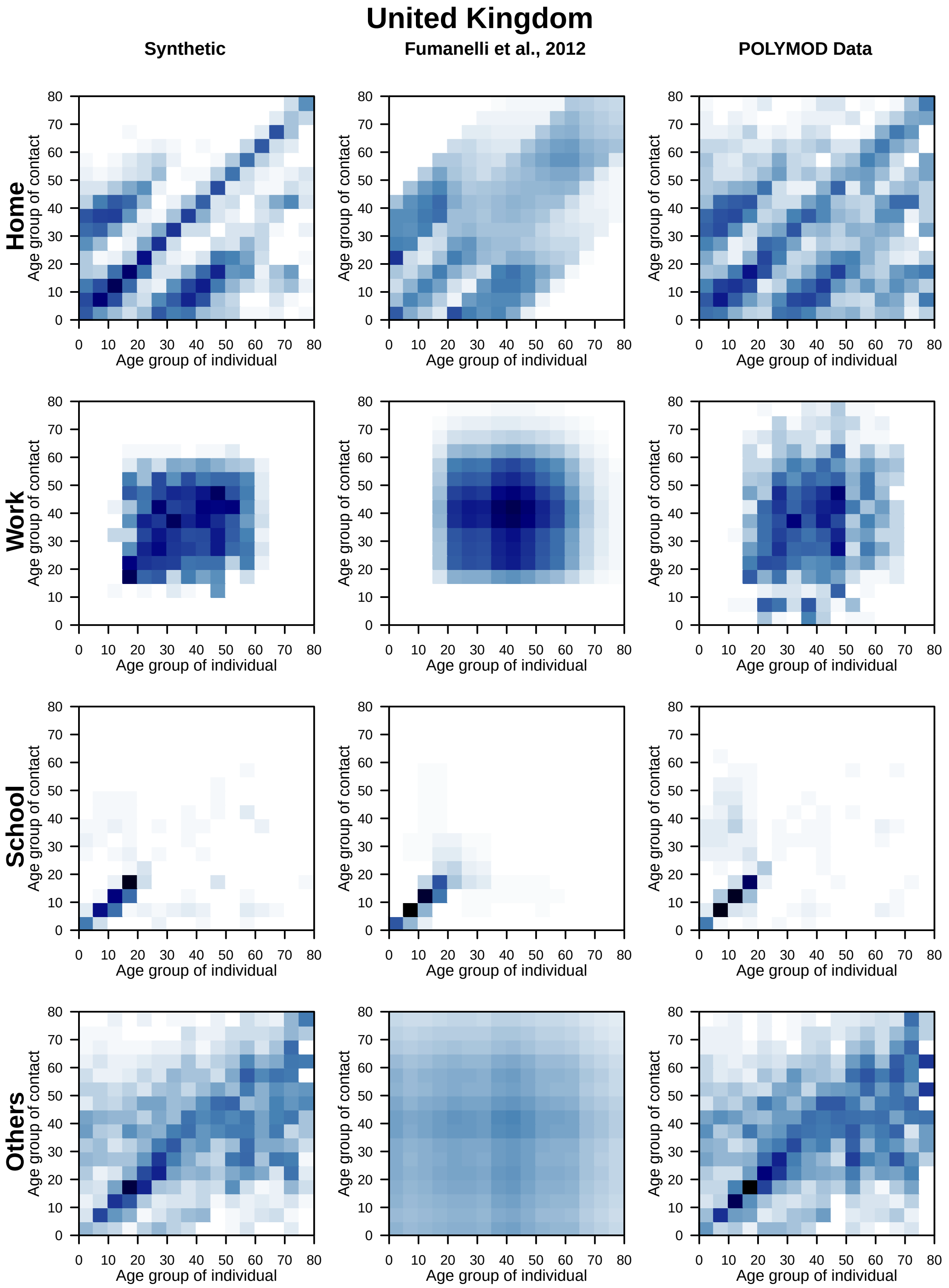
United Kingdom
Synthetic
Fumanelli et al., 2012
POLYMOD Data
Home
Work
School
Others
Age group of contact
Age group of individual
0
10
20
30
40
50
60
70
80

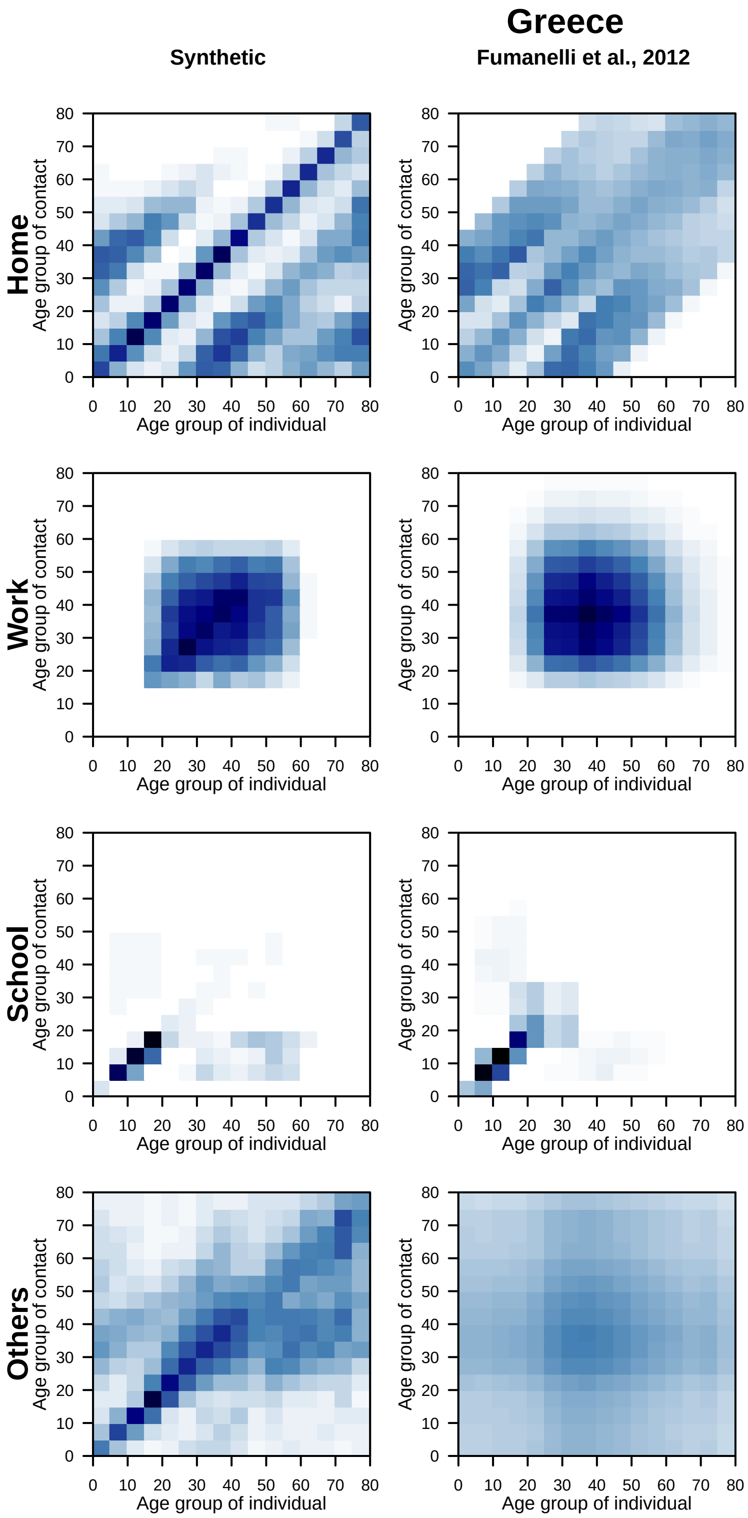

Greece
Synthetic
Fumanelli et al., 2012
Home
Work
School
Others
Age group of contact
Age group of individual
0
10
20
30
40
50
60
70
80

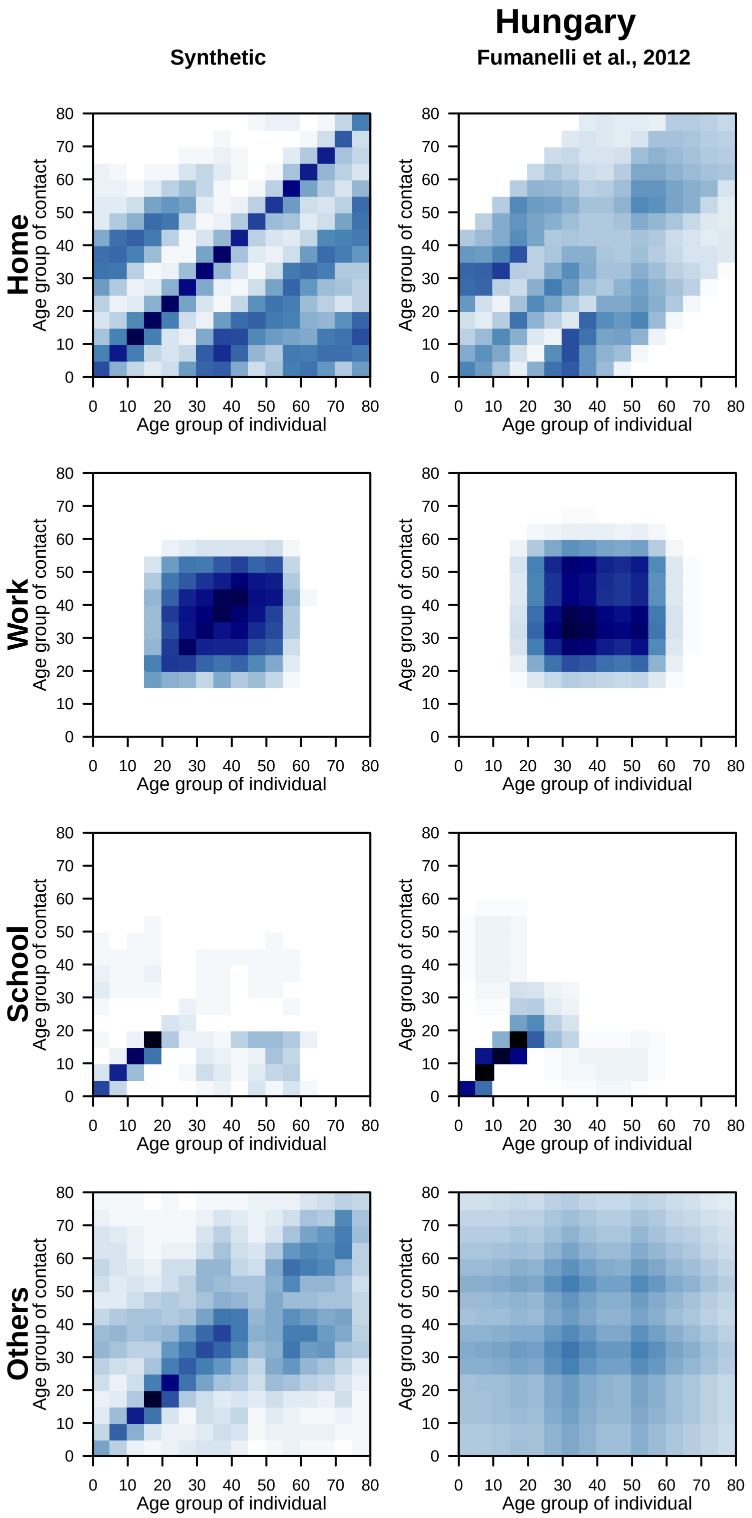
Hungary
Synthetic
Fumanelli et al., 2012
Home
Work
School
Others
Age group of contact
Age group of individual
0
10
20
30
40
50
60
70
80

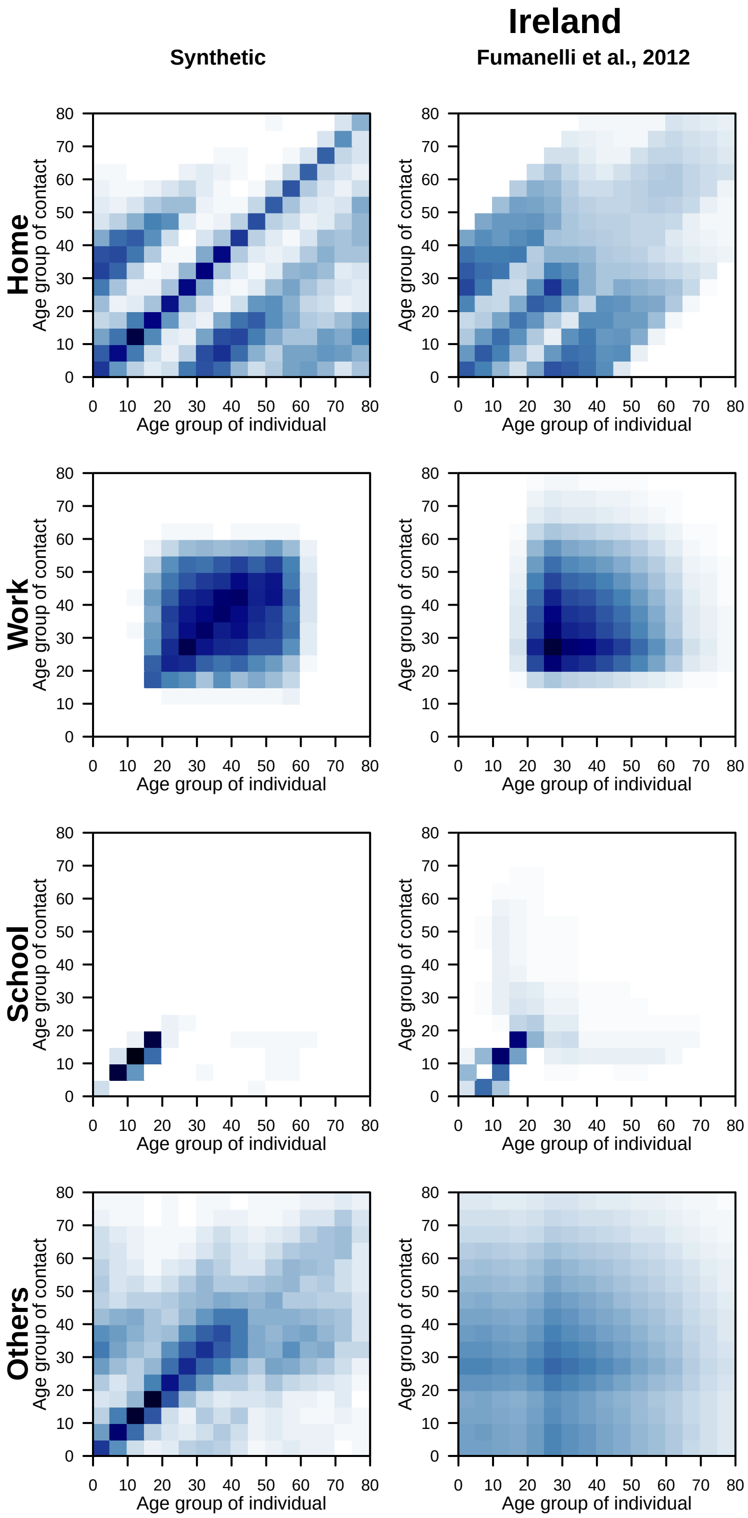

Ireland
Synthetic
Fumanelli et al., 2012
Home
Work
School
Others
Age group of contact
Age group of individual
0
10
20
30
40
50
60
70
80

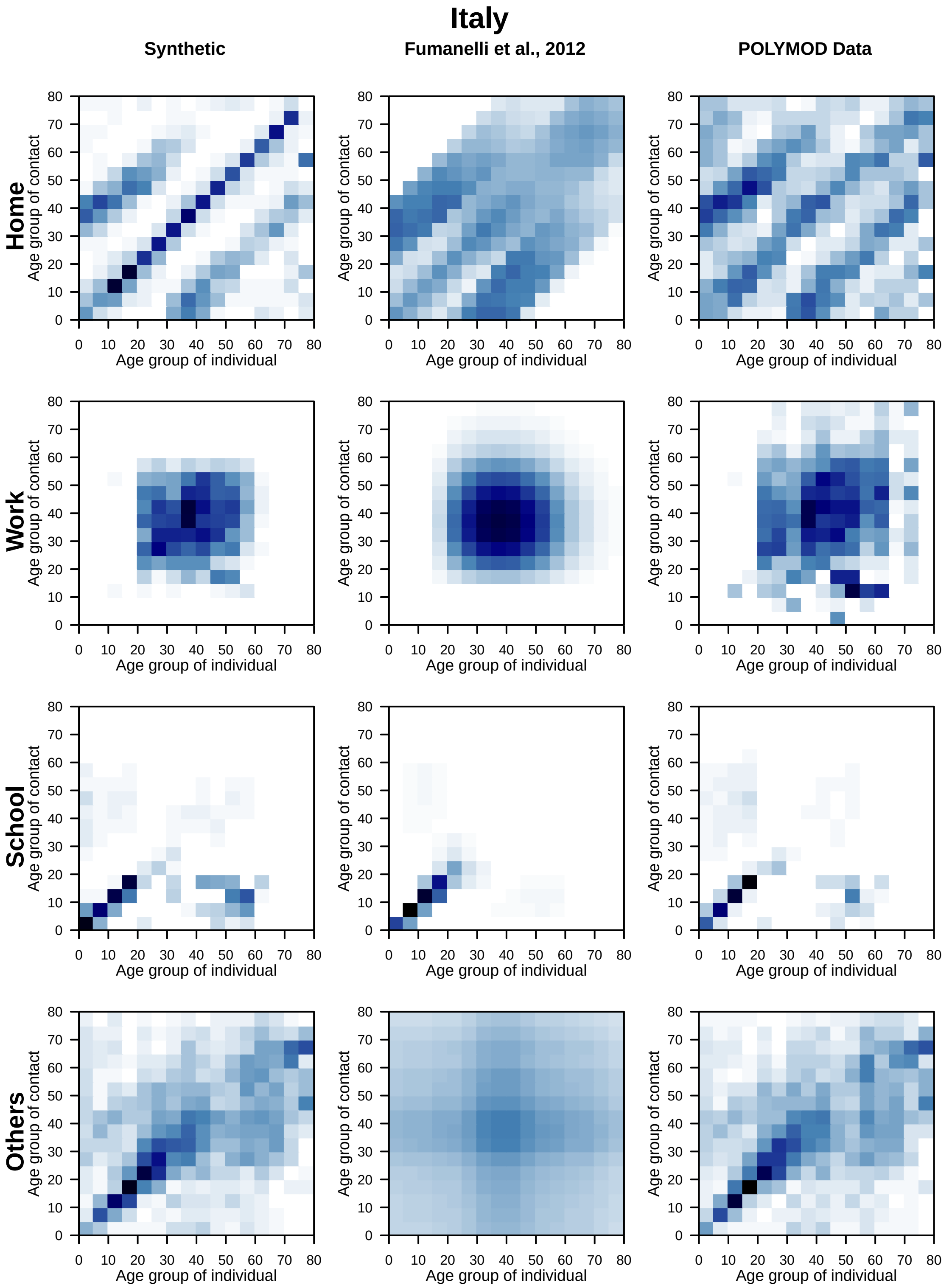
Italy
Synthetic
Fumanelli et al., 2012
POLYMOD Data
Home
Work
School
Others
Age group of contact
Age group of individual
0
10
20
30
40
50
60
70
80

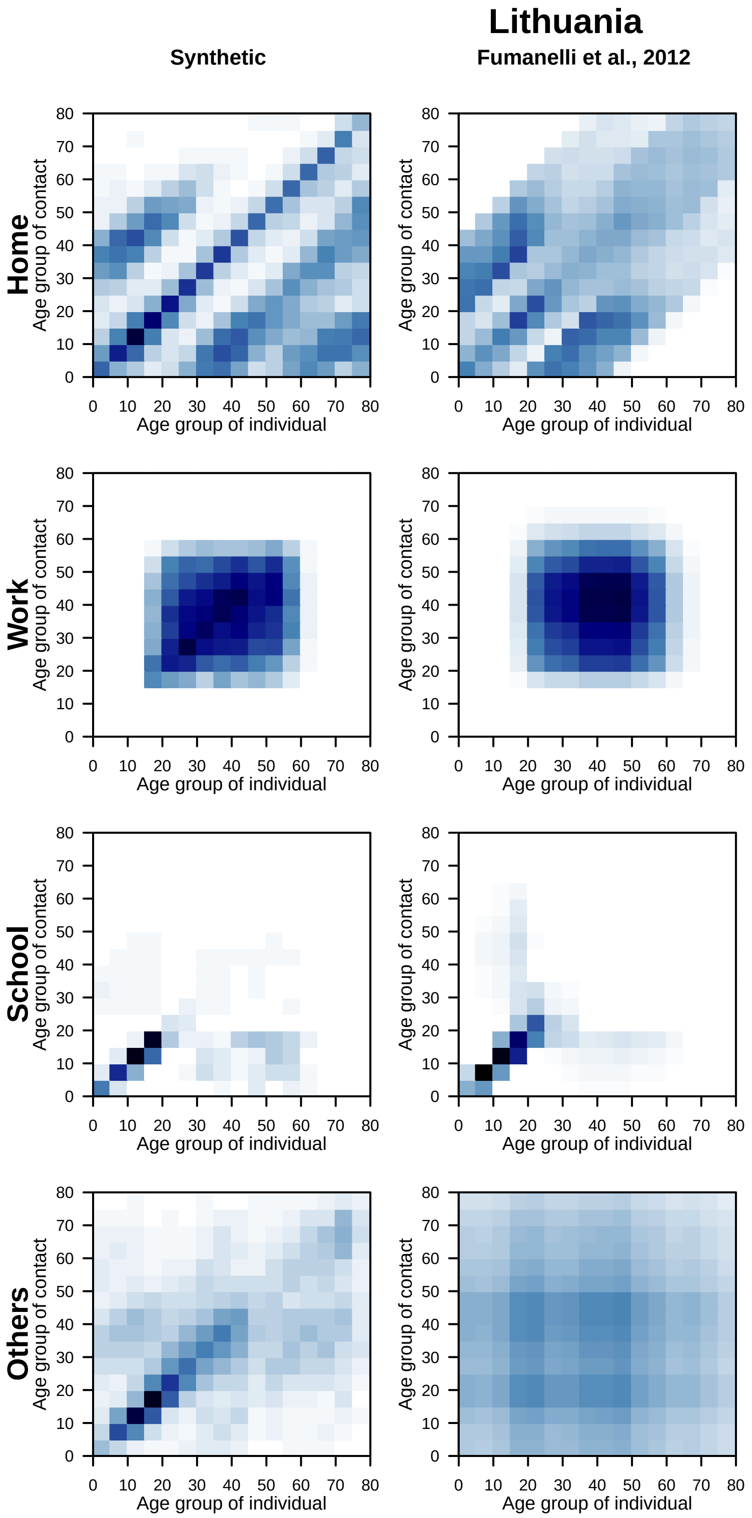

Lithuania
Synthetic
Fumanelli et al., 2012
Home
Work
School
Others
Age group of contact
Age group of individual
0
10
20
30
40
50
60
70
80

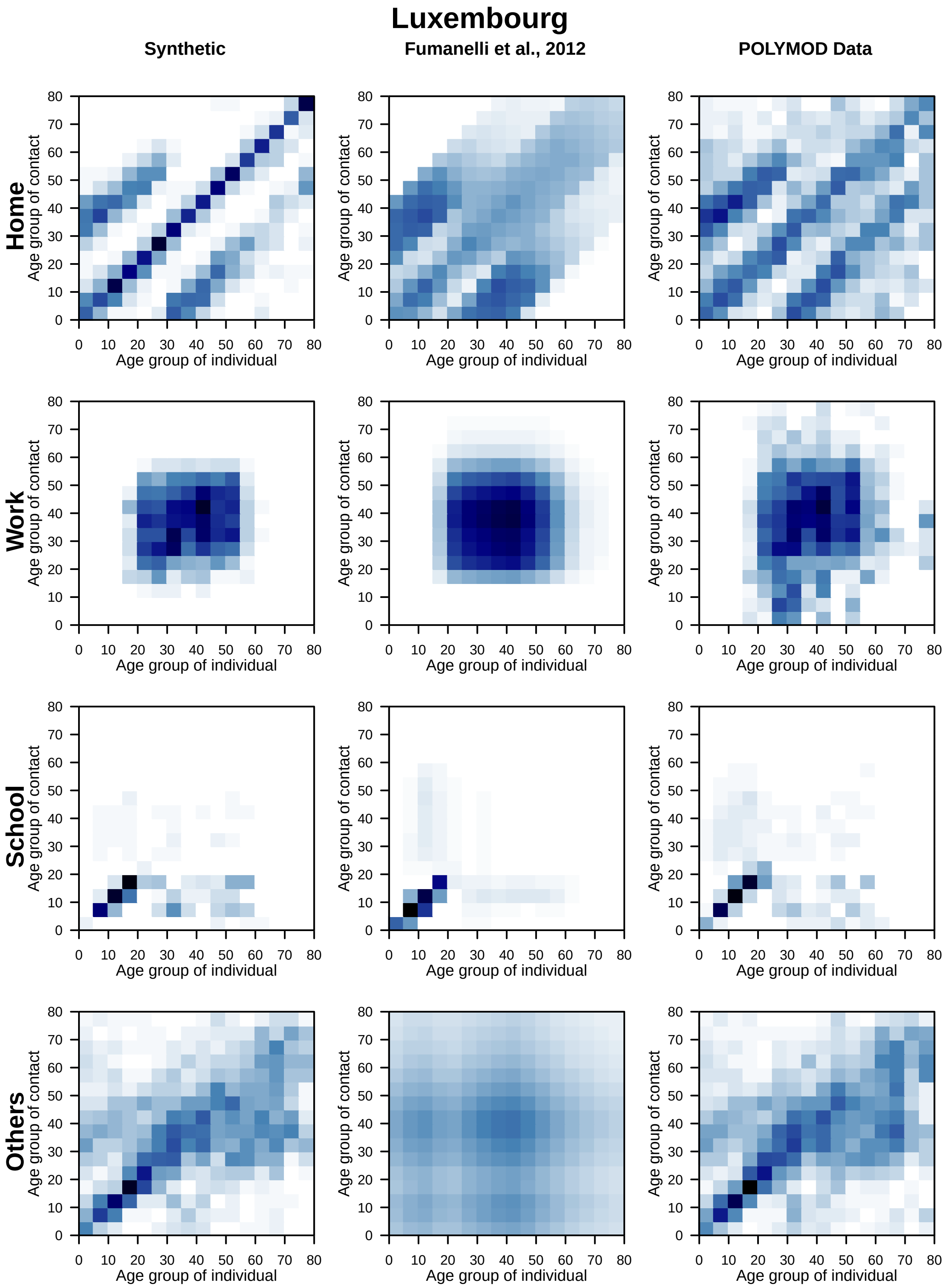
Luxembourg
Synthetic
Fumanelli et al., 2012
POLYMOD Data
Home
Work
School
Others
Age group of contact
Age group of individual
0
10
20
30
40
50
60
70
80

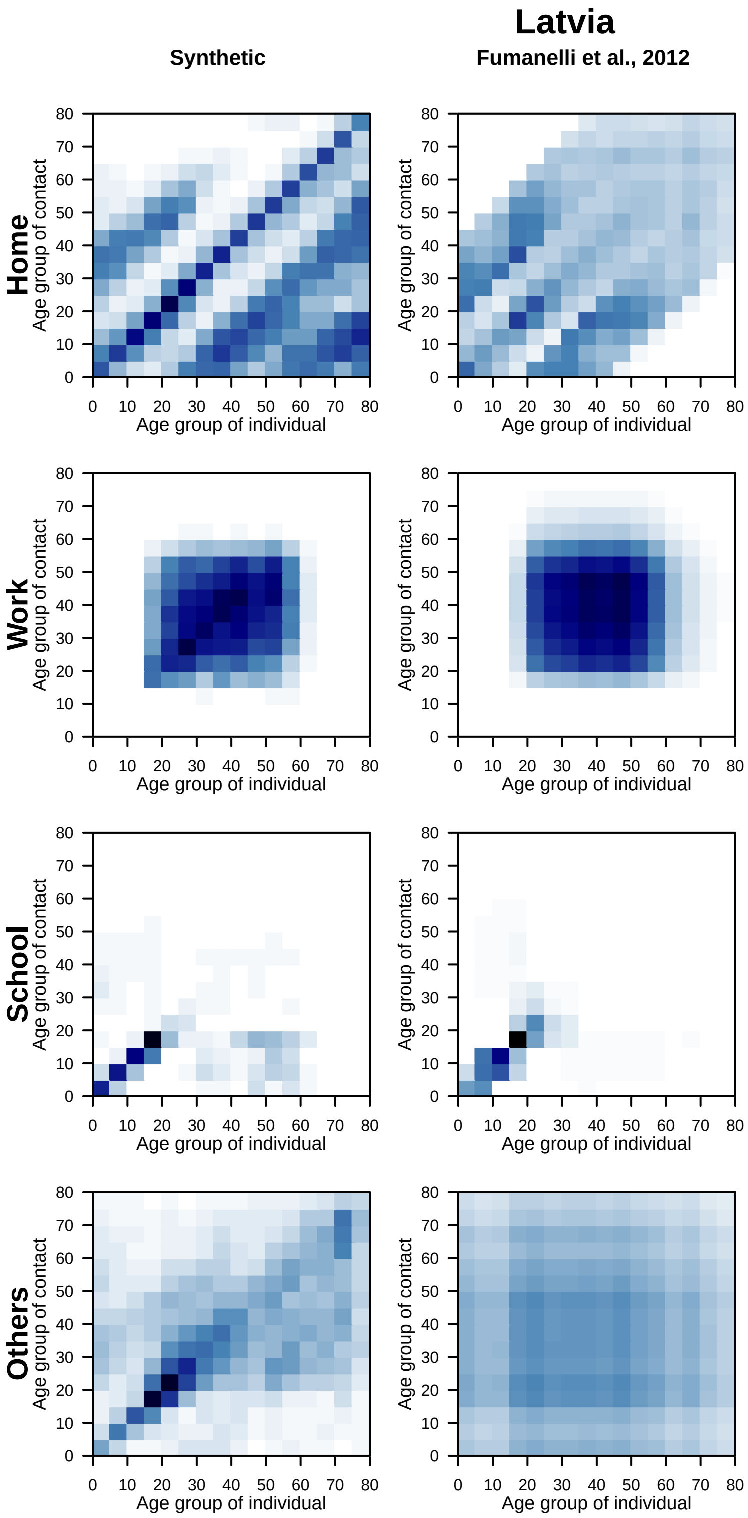
Latvia
Synthetic
Fumanelli et al., 2012
Home
Work
School
Others
Age group of contact
Age group of individual
0 10 20 30 40 50 60 70 80

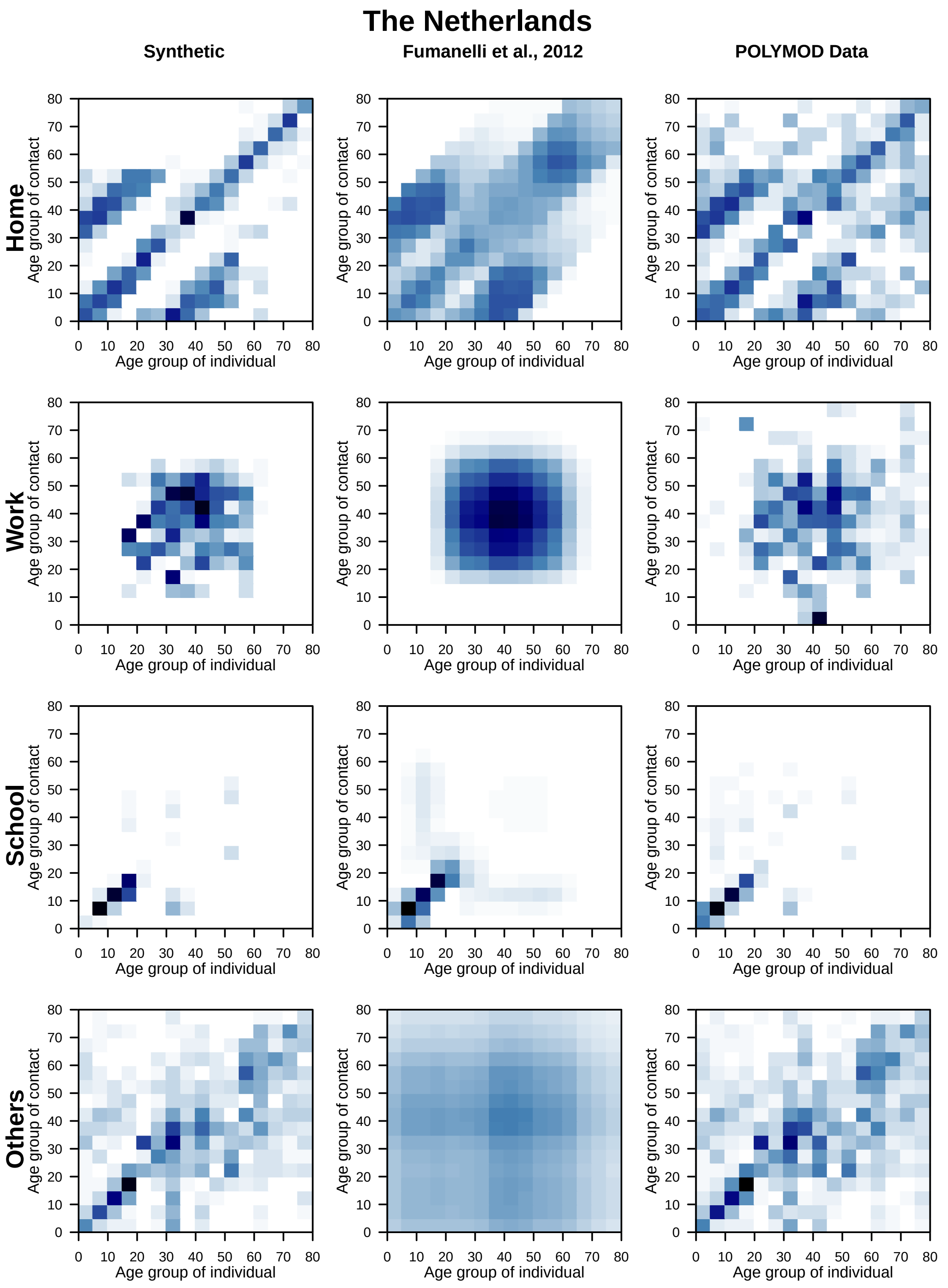
The Netherlands
Synthetic
Fumanelli et al., 2012
POLYMOD Data
Home
Work
School
Others
Age group of contact
Age group of individual
0
10
20
30
40
50
60
70
80

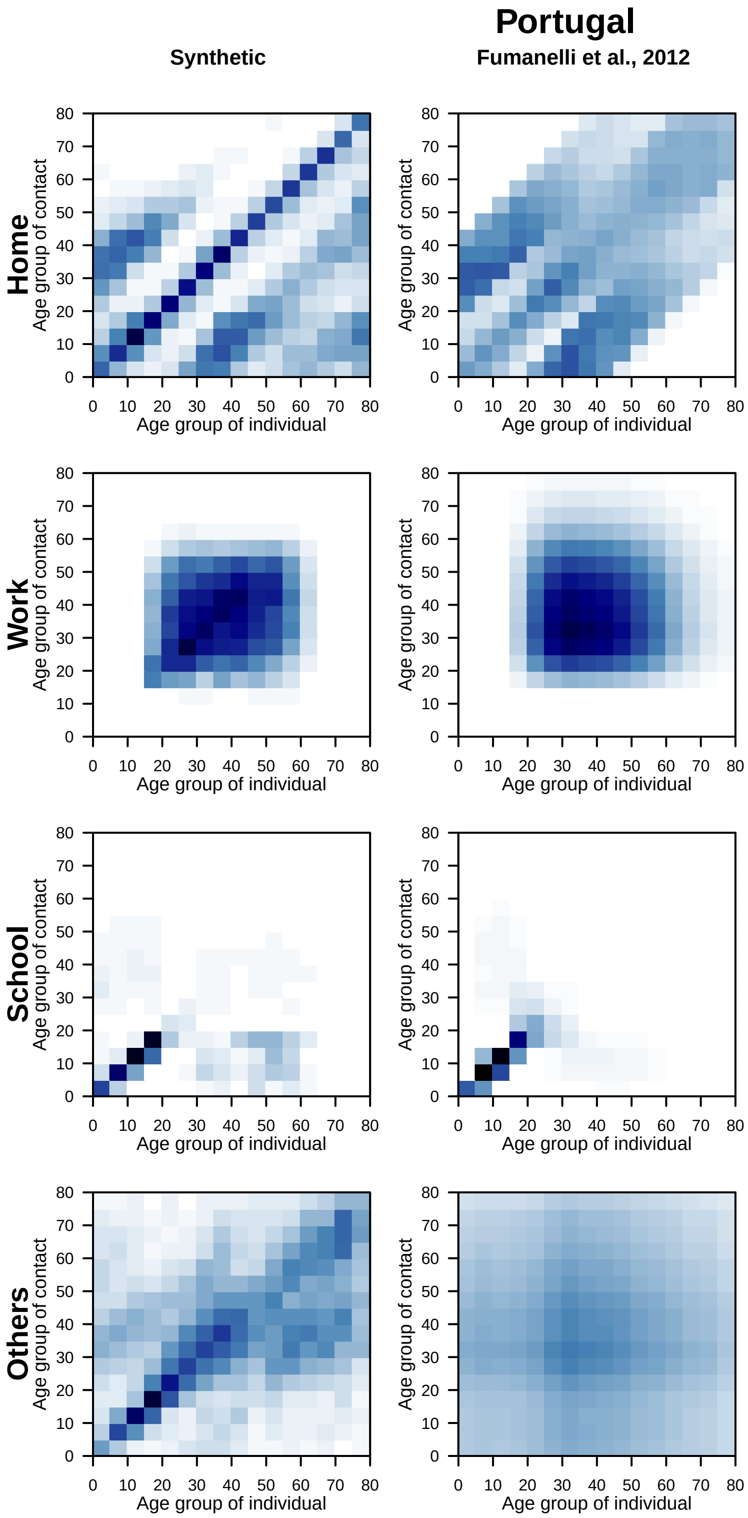
Portugal
Synthetic
Fumanelli et al., 2012
Home
Work
School
Others
Age group of contact
Age group of individual
0
10
20
30
40
50
60
70
80

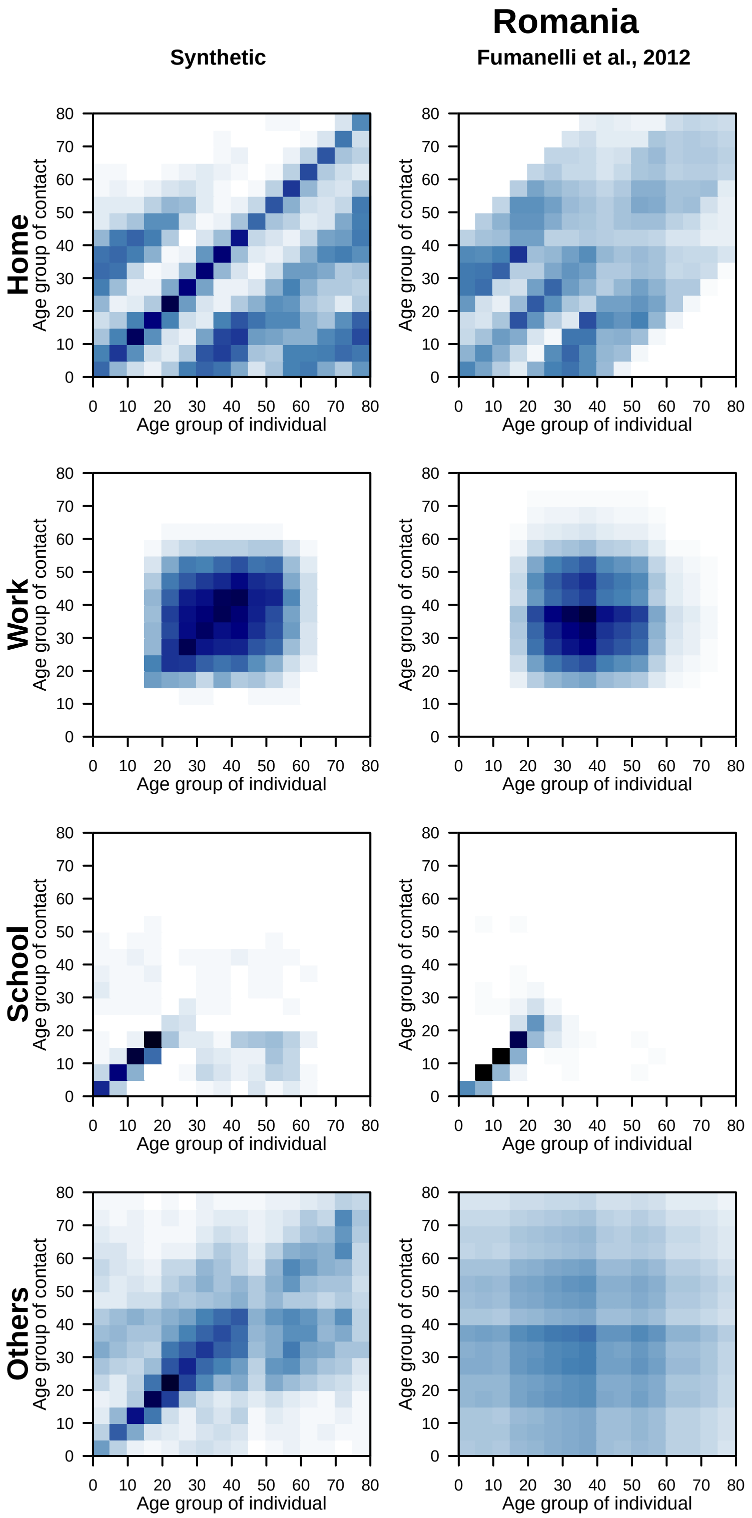
Romania
Synthetic
Fumanelli et al., 2012
Home
Work
School
Others
Age group of contact
Age group of individual
0
10
20
30
40
50
60
70
80

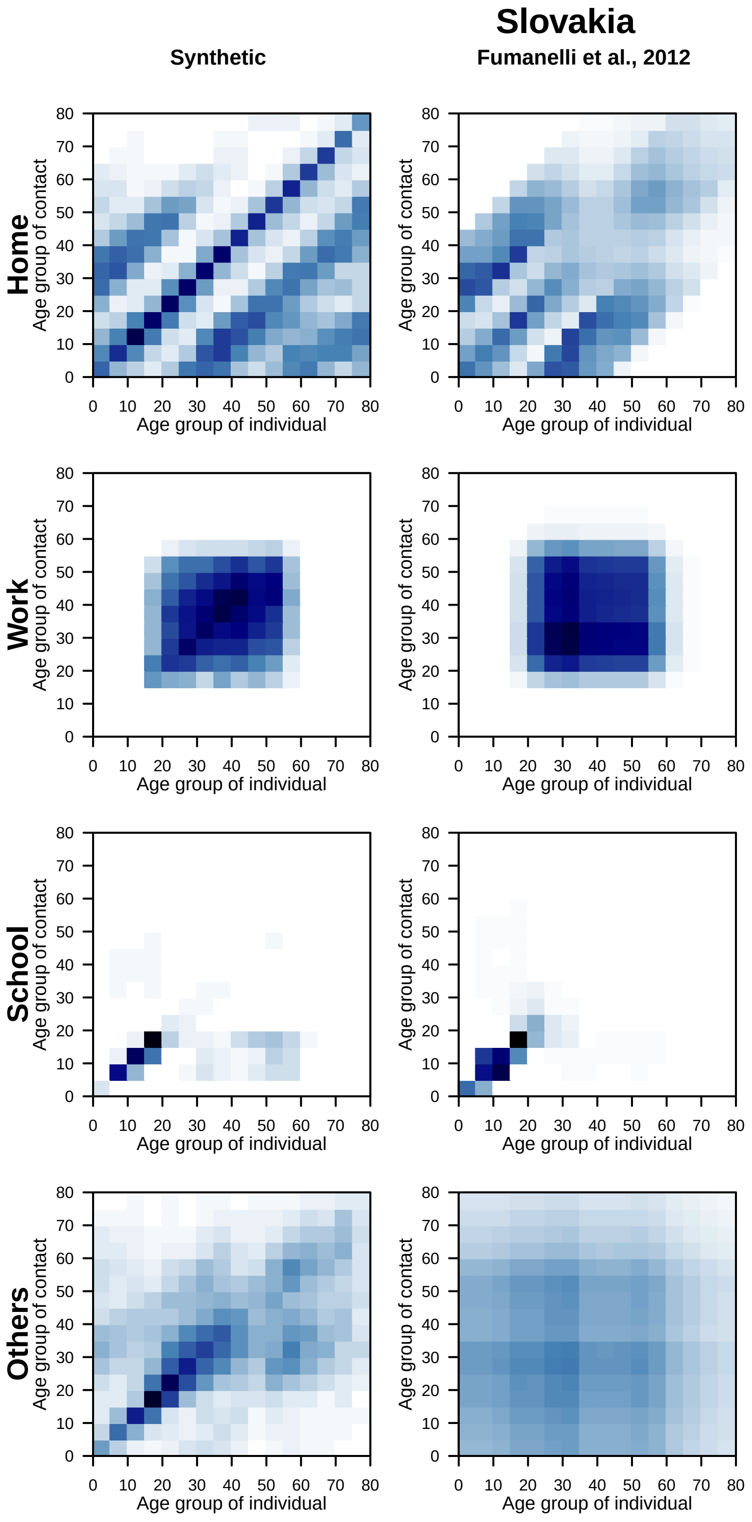
Slovakia
Synthetic
Fumanelli et al., 2012
Home
Work
School
Others
Age group of contact
Age group of individual
0
10
20
30
40
50
60
70
80

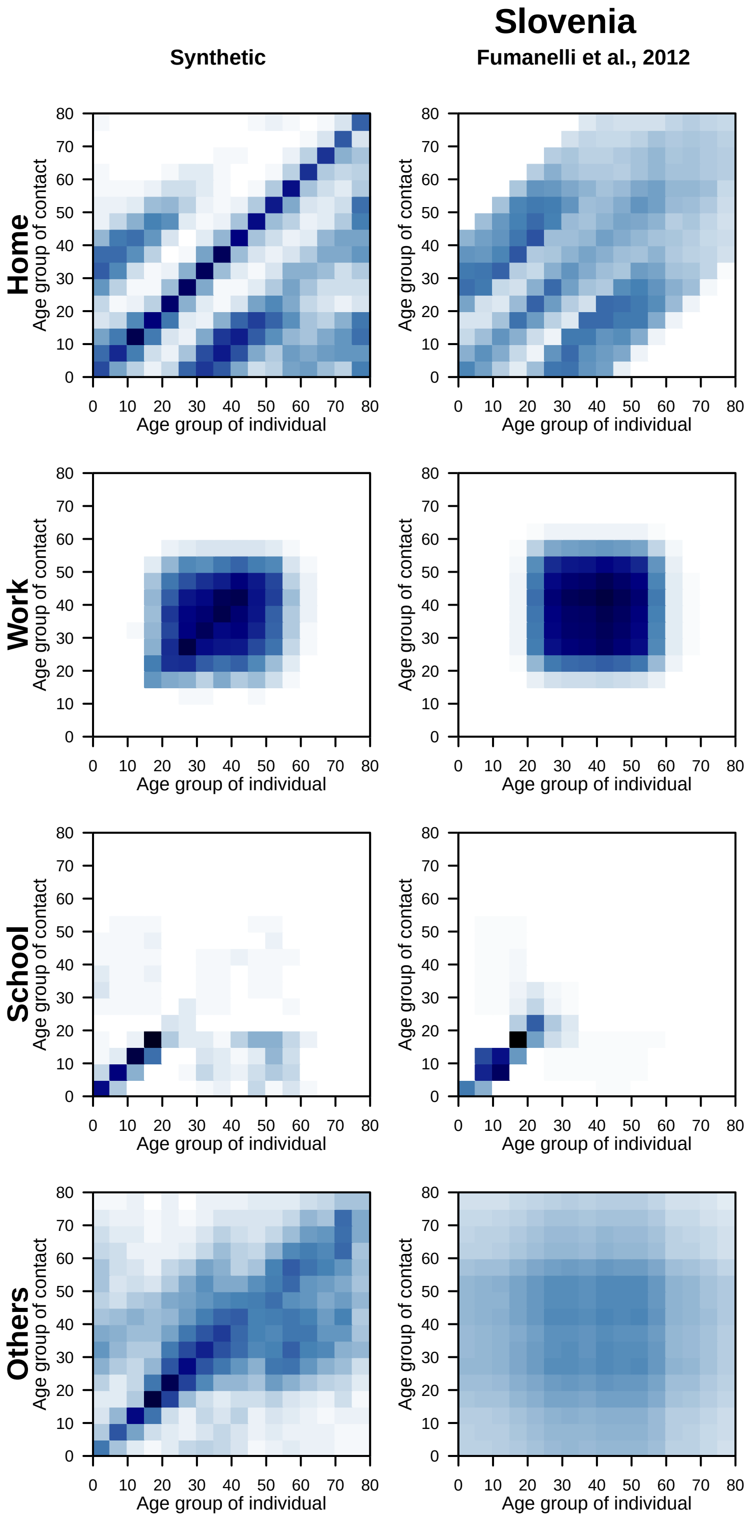
Slovenia
Synthetic
Fumanelli et al., 2012
Home
Work
School
Others
Age group of contact
Age group of individual
0
10
20
30
40
50
60
70
80

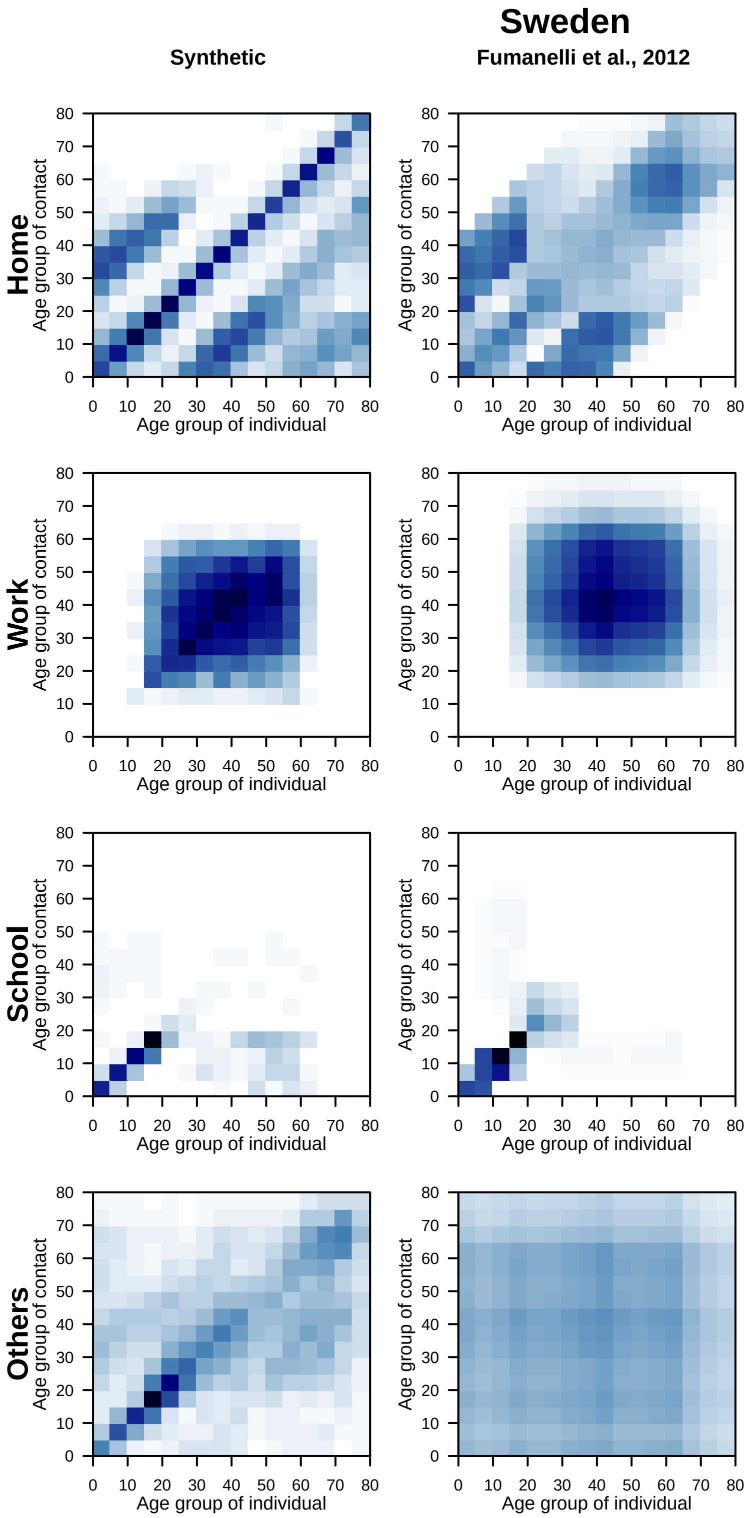

Sweden
Synthetic
Fumanelli et al., 2012
Home
Work
School
Others
Age group of contact
Age group of individual
0
10
20
30
40
50
60
70
80

Cyprus

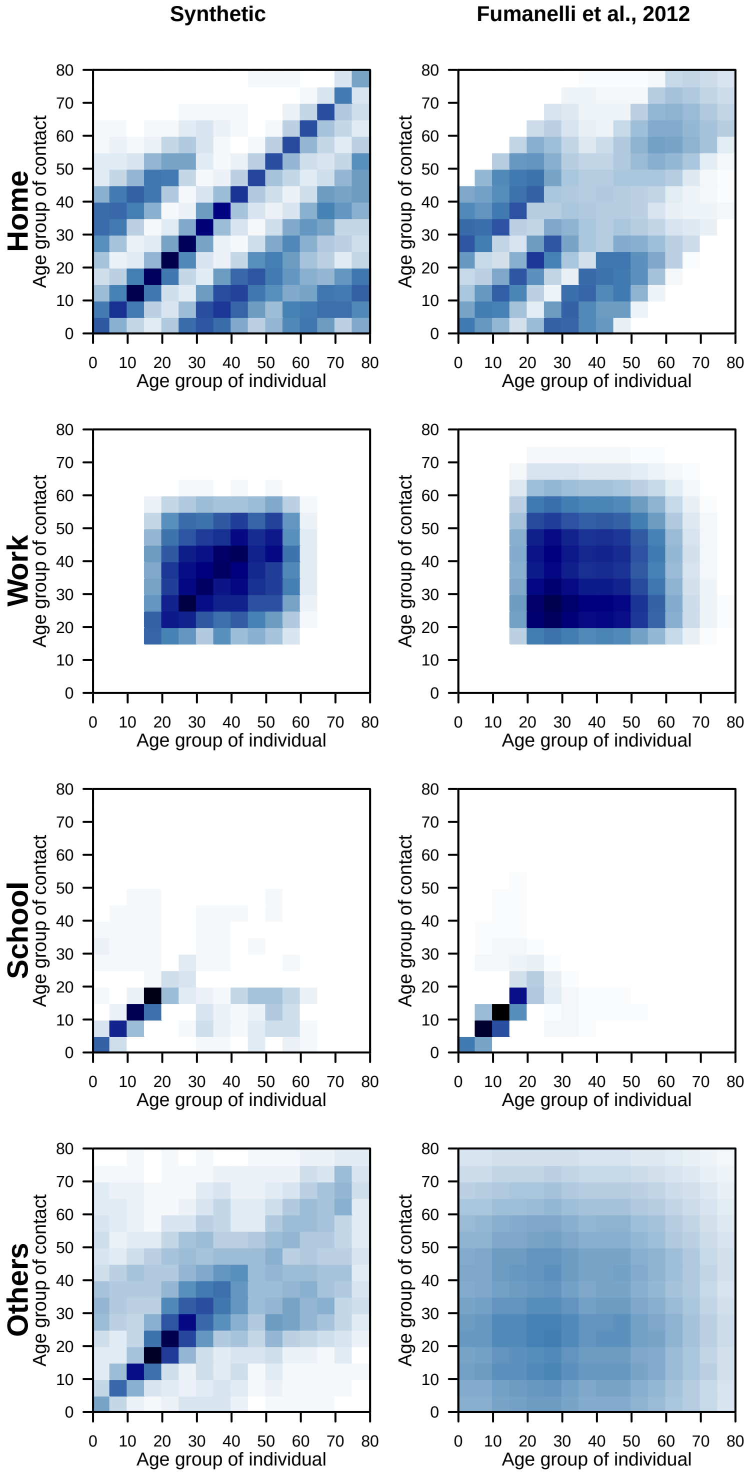
Synthetic
Fumanelli et al., 2012
Home
Work
School
Others
Age group of contact
Age group of individual
0
10
20
30
40
50
60
70
80

Albania

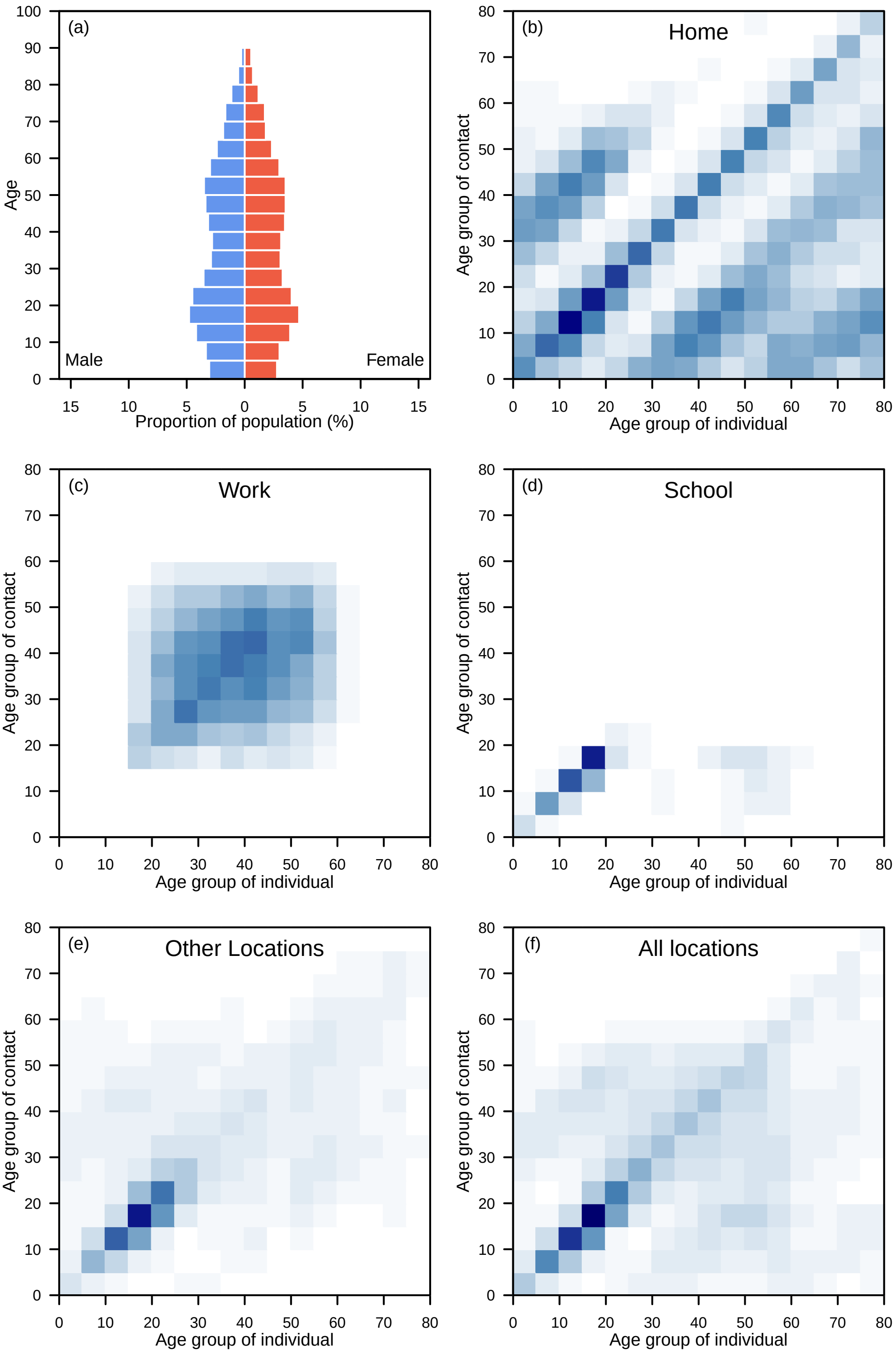

(a)
Male
Female
Age
Proportion of population (%)
(b)
Home
(c)
Work
(d)
School
(e)
Other Locations
(f)
All locations
Age group of contact
Age group of individual

Algeria

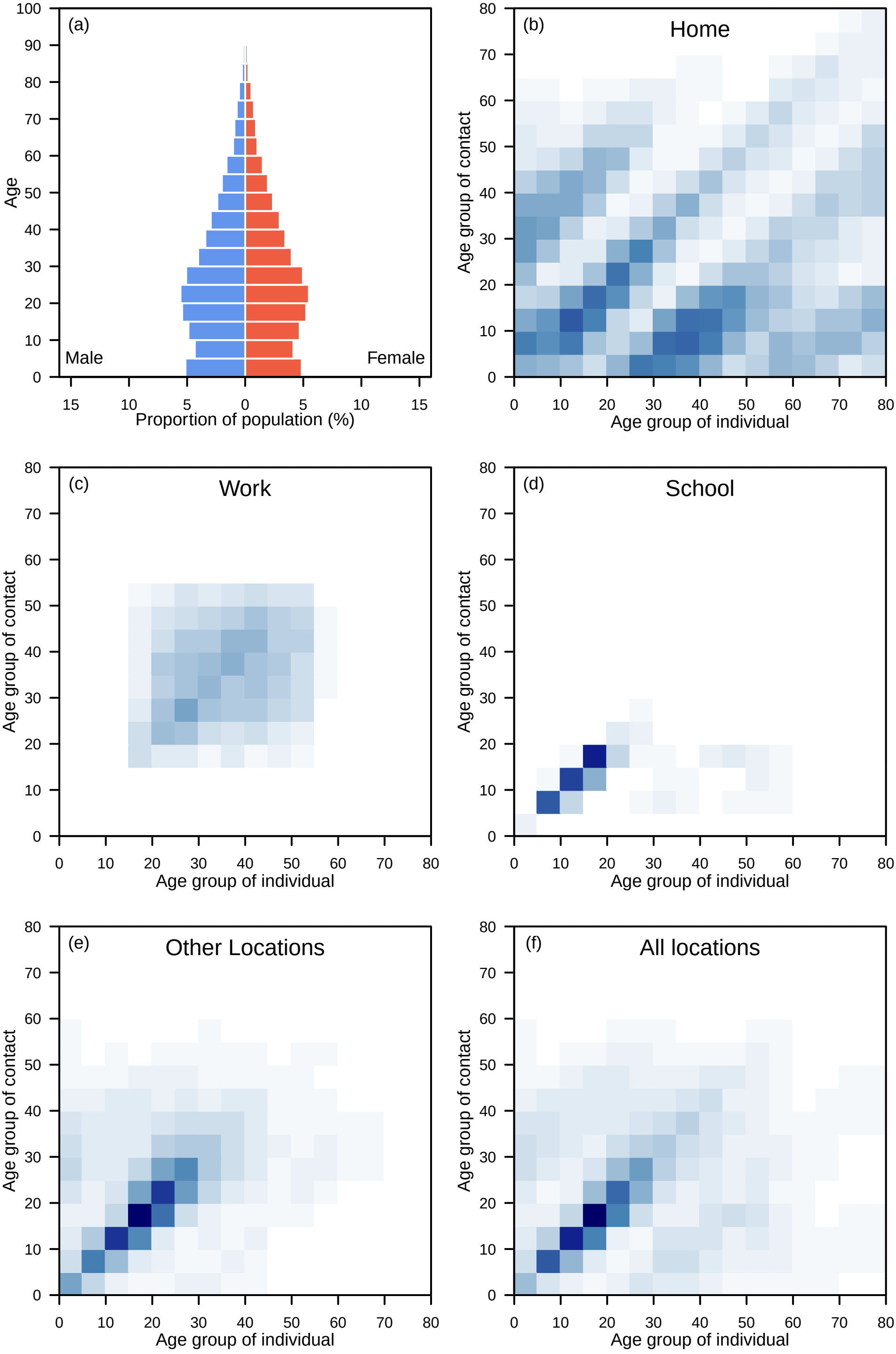

(a)
Age
Male
Female
Proportion of population (%)
(b)
Home
(c)
Work
(d)
School
(e)
Other Locations
(f)
All locations
Age group of contact
Age group of individual

Andorra

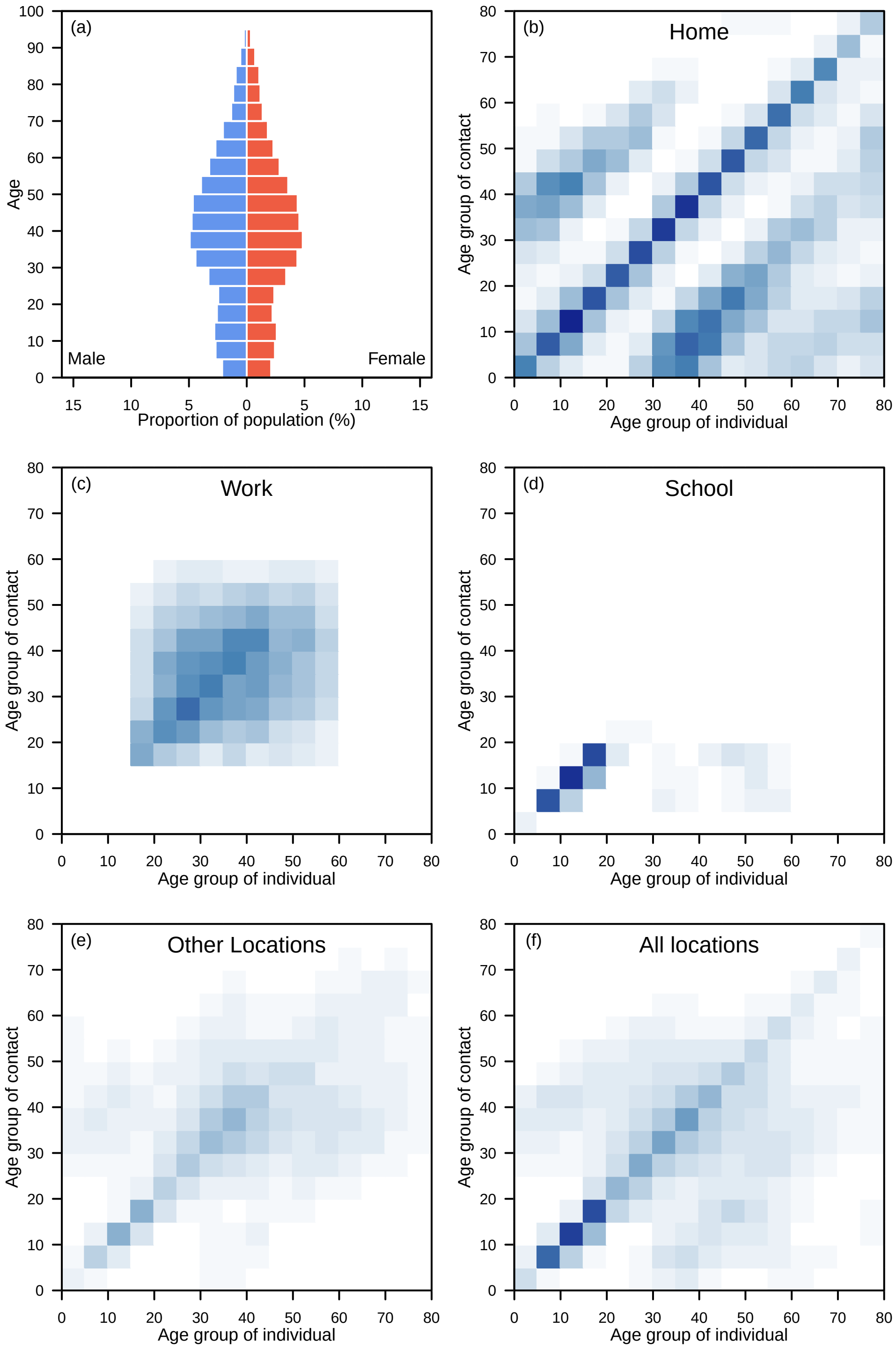

(a)
Male
Female
Age
Proportion of population (%)
(b)
Home
Age group of contact
Age group of individual
(c)
Work
(d)
School
(e)
Other Locations
(f)
All locations

Antigua and Barbuda

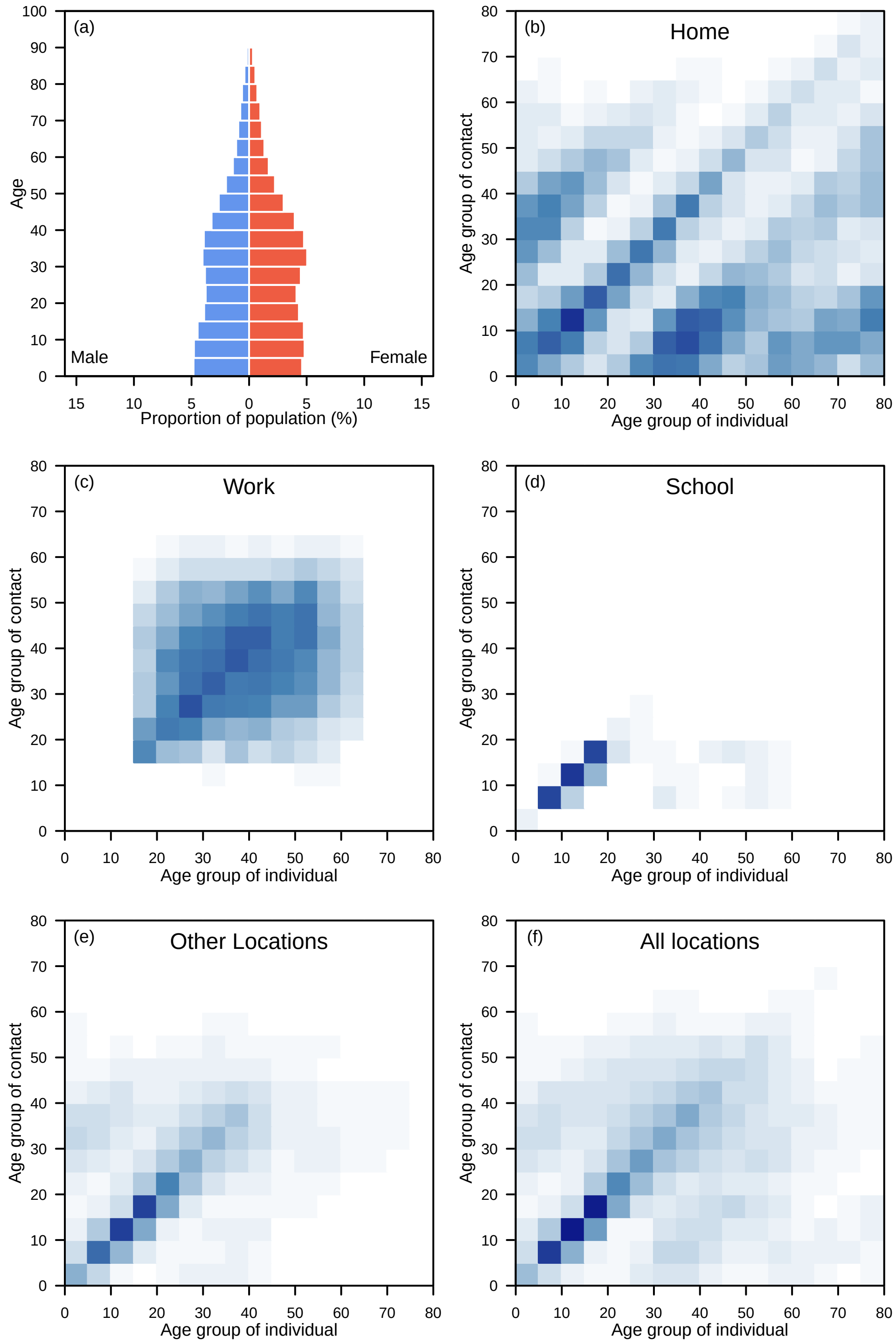

Argentina

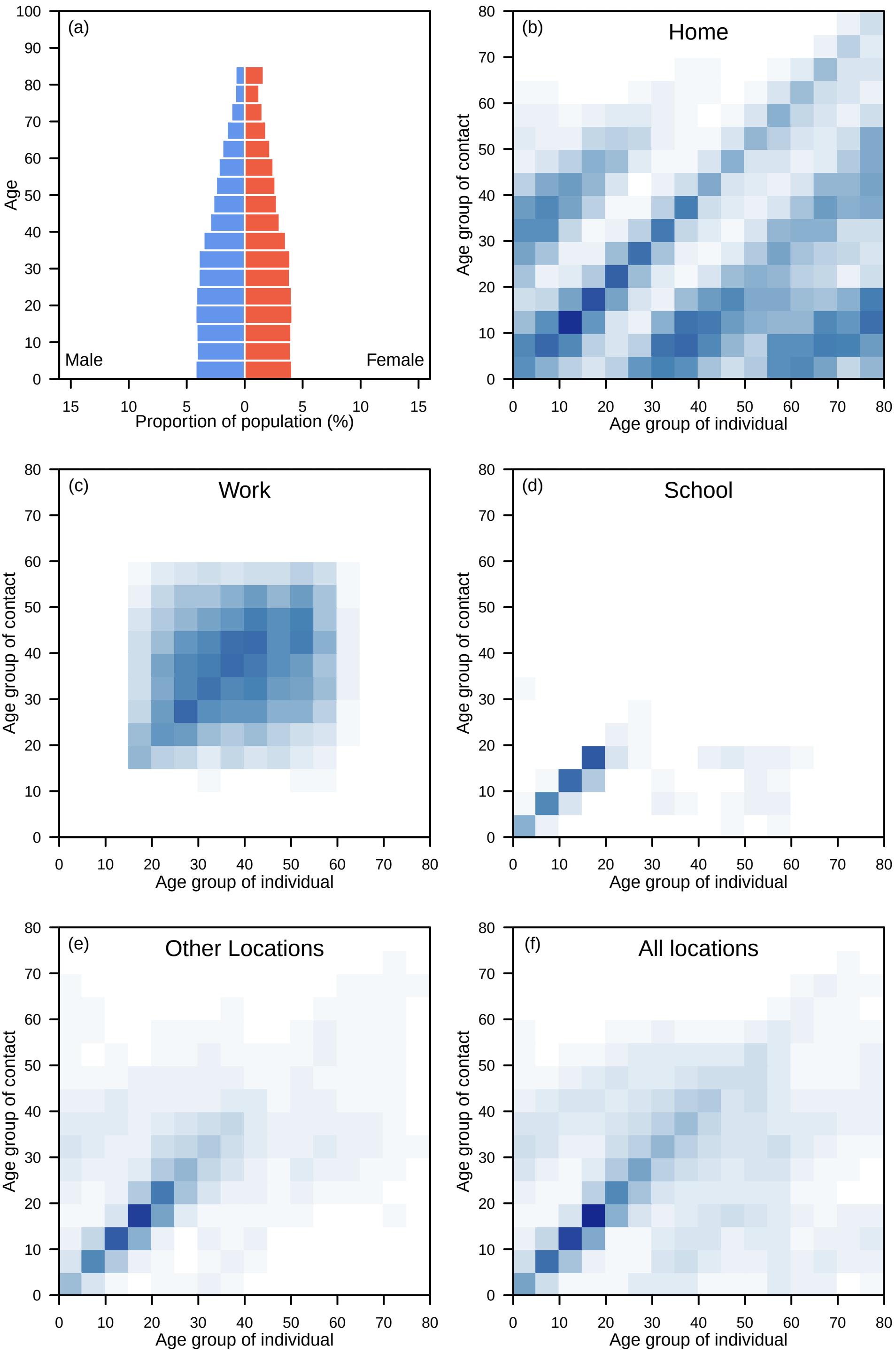

(a)
Age
Male
Female
Proportion of population (%)
(b)
Home
Age group of contact
Age group of individual
(c)
Work
Age group of contact
Age group of individual
(d)
School
Age group of contact
Age group of individual
(e)
Other Locations
Age group of contact
Age group of individual
(f)
All locations
Age group of contact
Age group of individual

Armenia

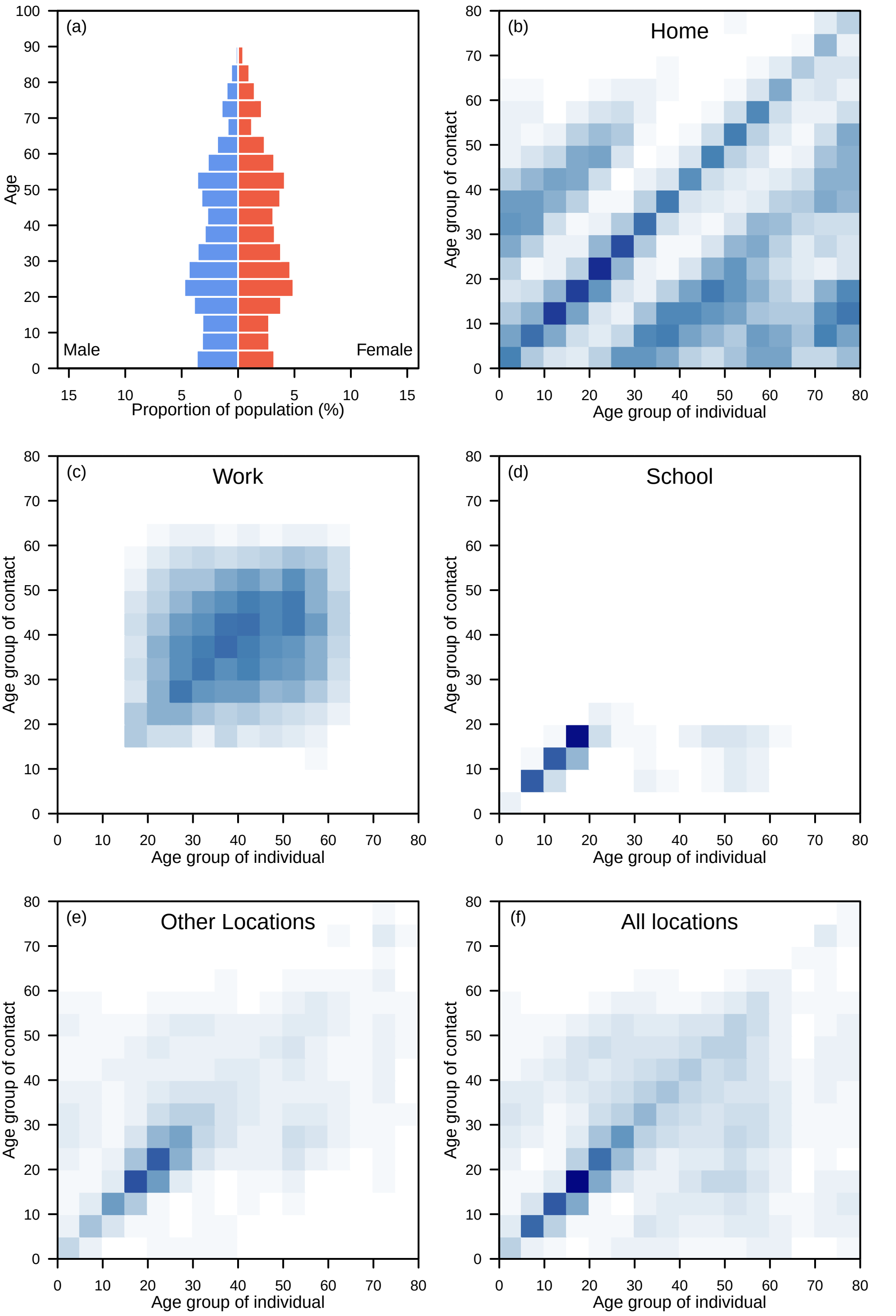

(a)
Age
Male
Female
Proportion of population (%)
(b)
Home
Age group of contact
Age group of individual
(c)
Work
Age group of contact
Age group of individual
(d)
School
Age group of contact
Age group of individual
(e)
Other Locations
Age group of contact
Age group of individual
(f)
All locations
Age group of contact
Age group of individual

Australia

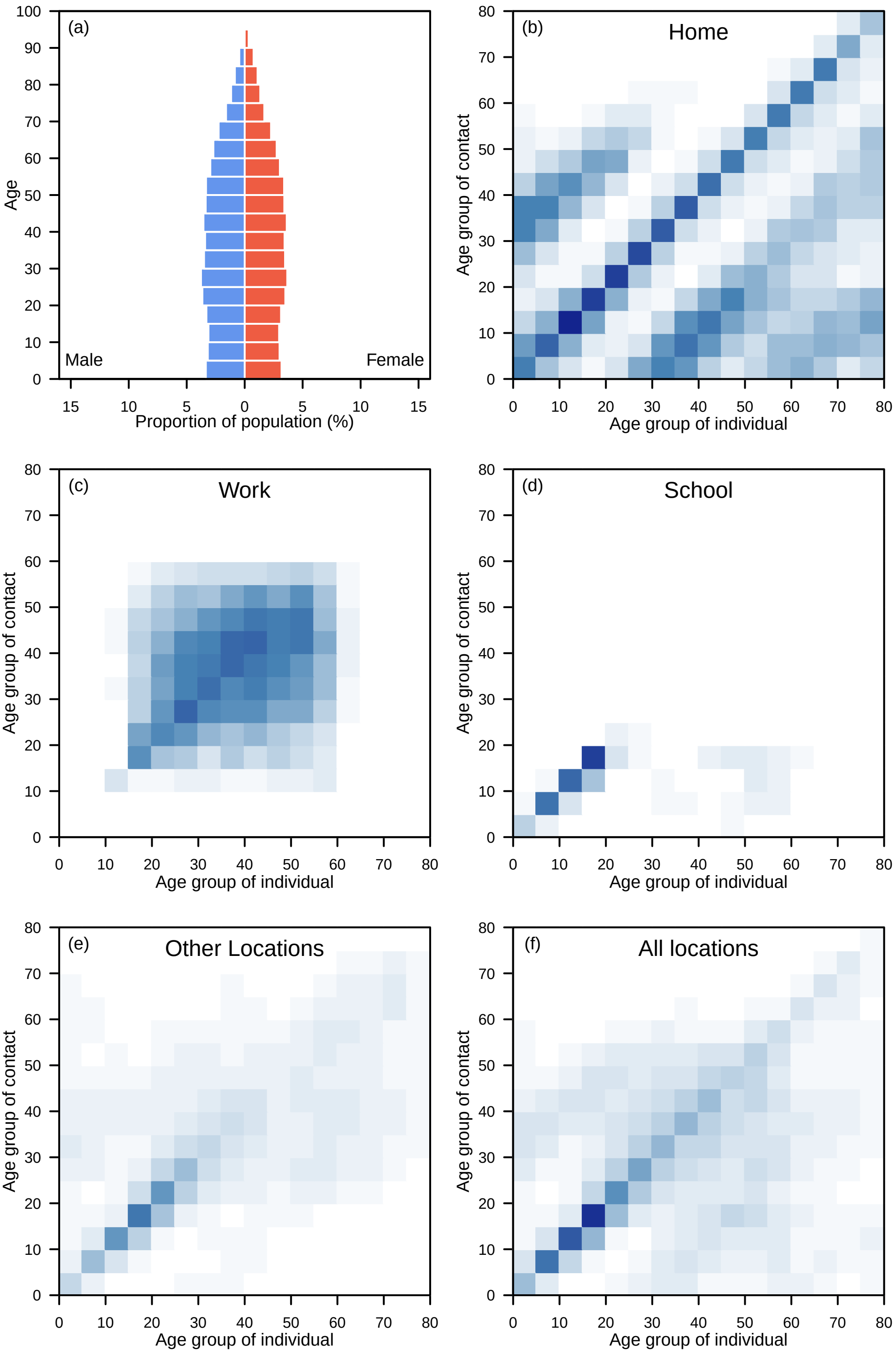

(a)
Age
Male
Female
Proportion of population (%)
(b)
Home
Age group of contact
Age group of individual
(c)
Work
Age group of contact
Age group of individual
(d)
School
Age group of contact
Age group of individual
(e)
Other Locations
Age group of contact
Age group of individual
(f)
All locations
Age group of contact
Age group of individual

Austria

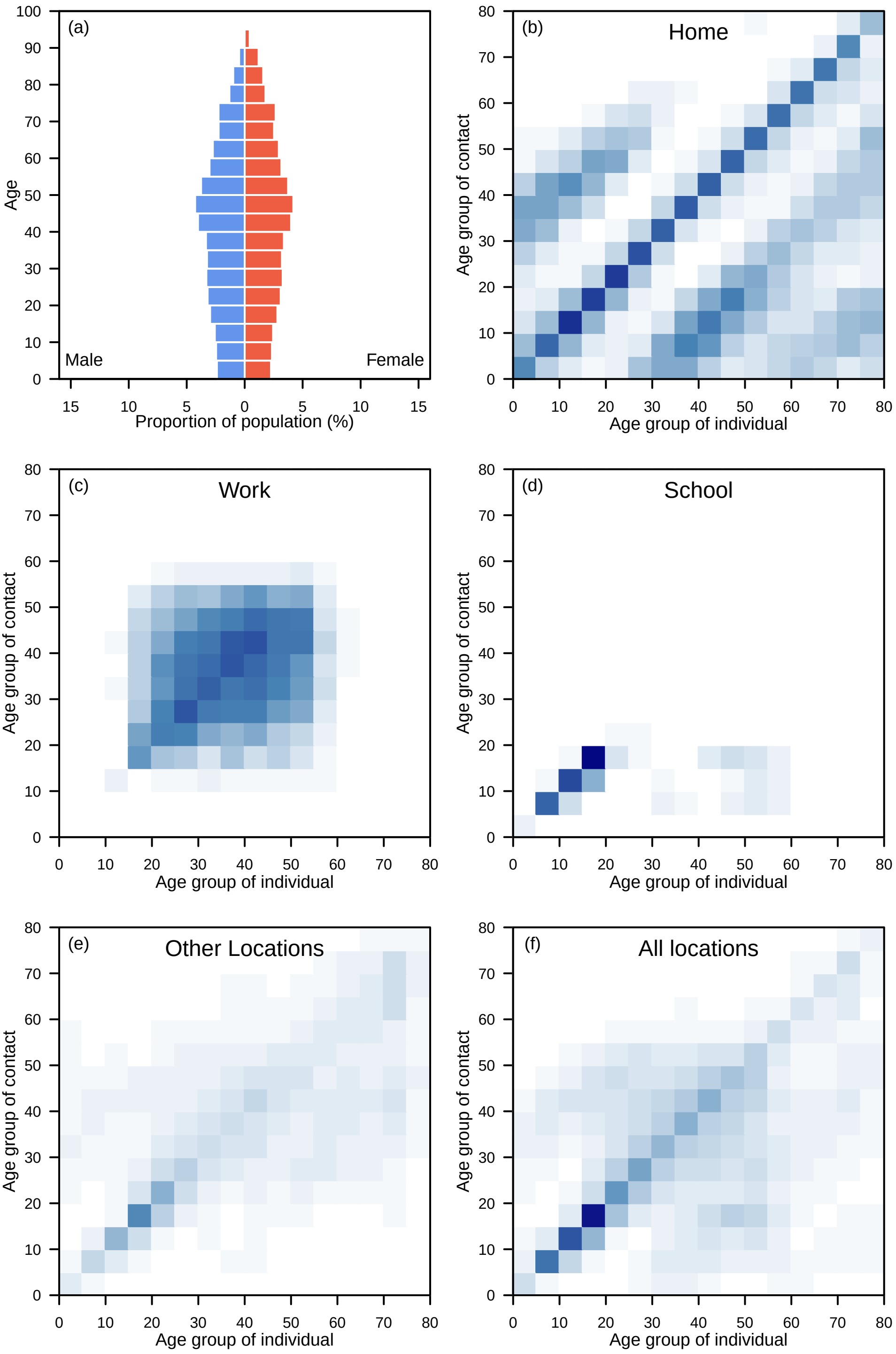

(a)
Age
Male
Female
Proportion of population (%)
(b)
Home
Age group of contact
Age group of individual
(c)
Work
Age group of contact
Age group of individual
(d)
School
Age group of contact
Age group of individual
(e)
Other Locations
Age group of contact
Age group of individual
(f)
All locations
Age group of contact
Age group of individual

Azerbaijan

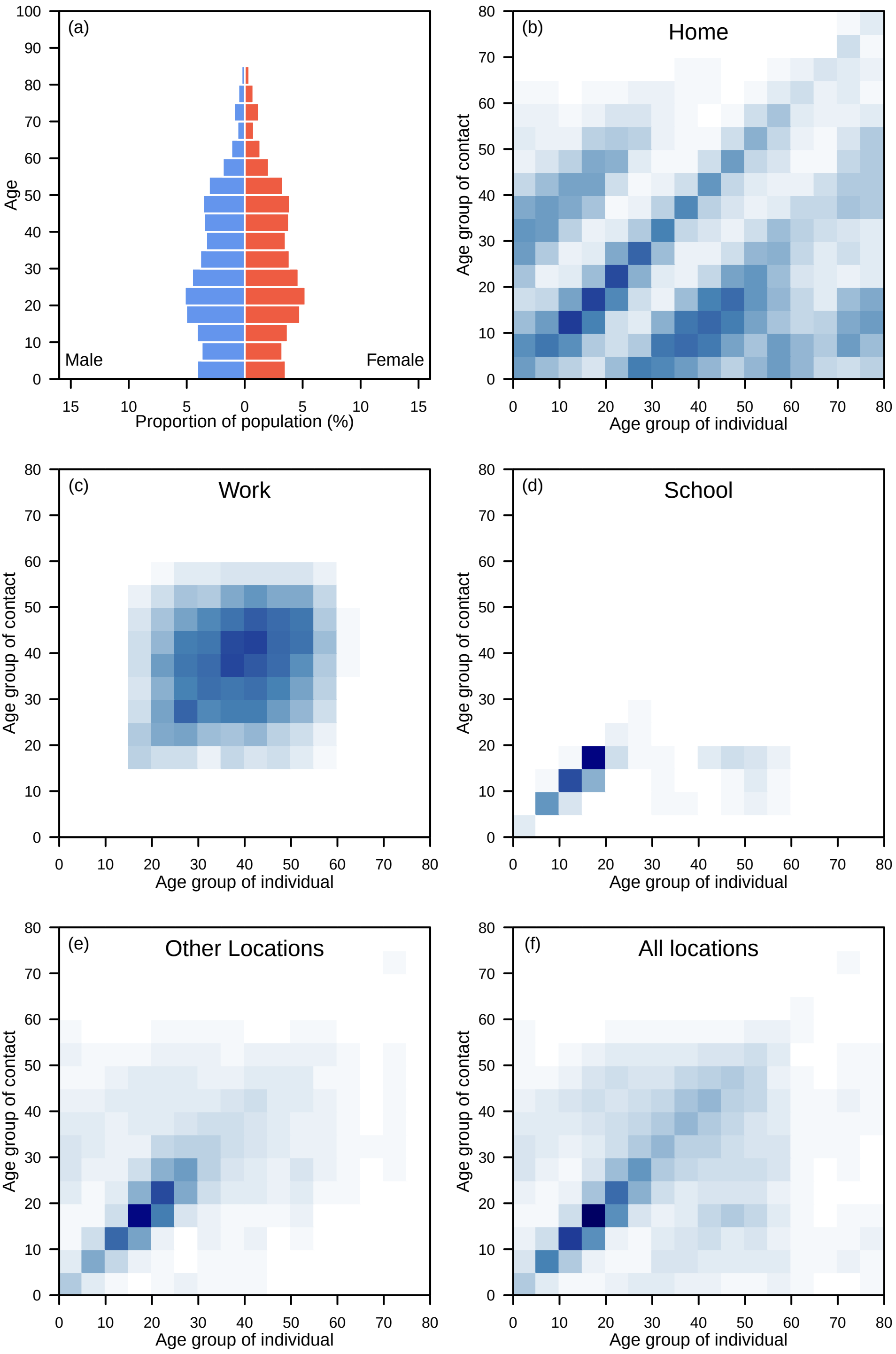

(a)
Age
Male
Female
Proportion of population (%)
(b)
Home
Age group of contact
Age group of individual
(c)
Work
Age group of contact
Age group of individual
(d)
School
Age group of contact
Age group of individual
(e)
Other Locations
Age group of contact
Age group of individual
(f)
All locations
Age group of contact
Age group of individual

Bahamas

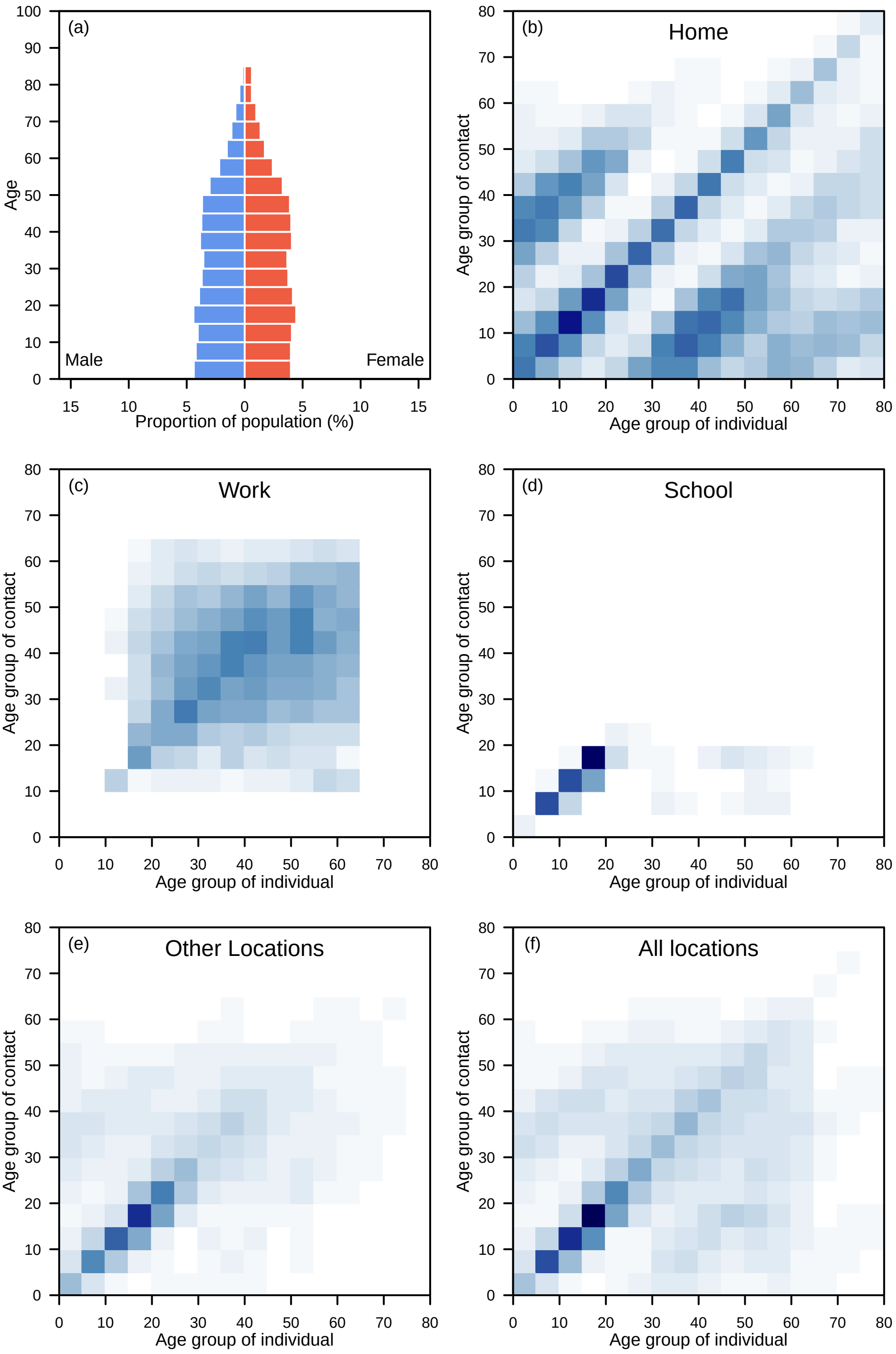

(a)
Age
Male
Female
Proportion of population (%)
(b)
Home
Age group of contact
Age group of individual
(c)
Work
Age group of contact
Age group of individual
(d)
School
Age group of contact
Age group of individual
(e)
Other Locations
Age group of contact
Age group of individual
(f)
All locations
Age group of contact
Age group of individual

Bahrain

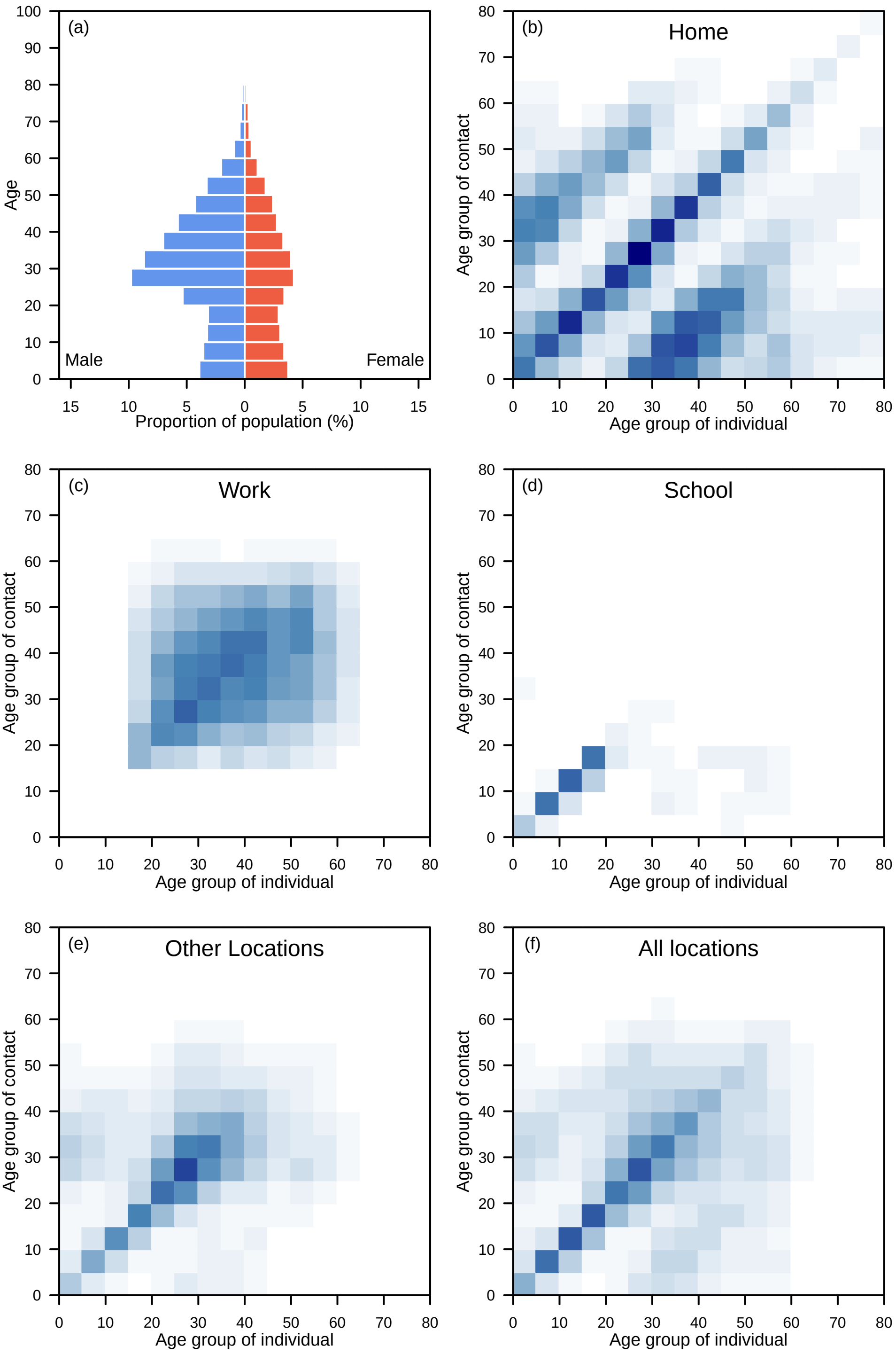

(a)
Age
Male
Female
Proportion of population (%)
(b)
Home
Age group of contact
Age group of individual
(c)
Work
(d)
School
(e)
Other Locations
(f)
All locations

Bangladesh

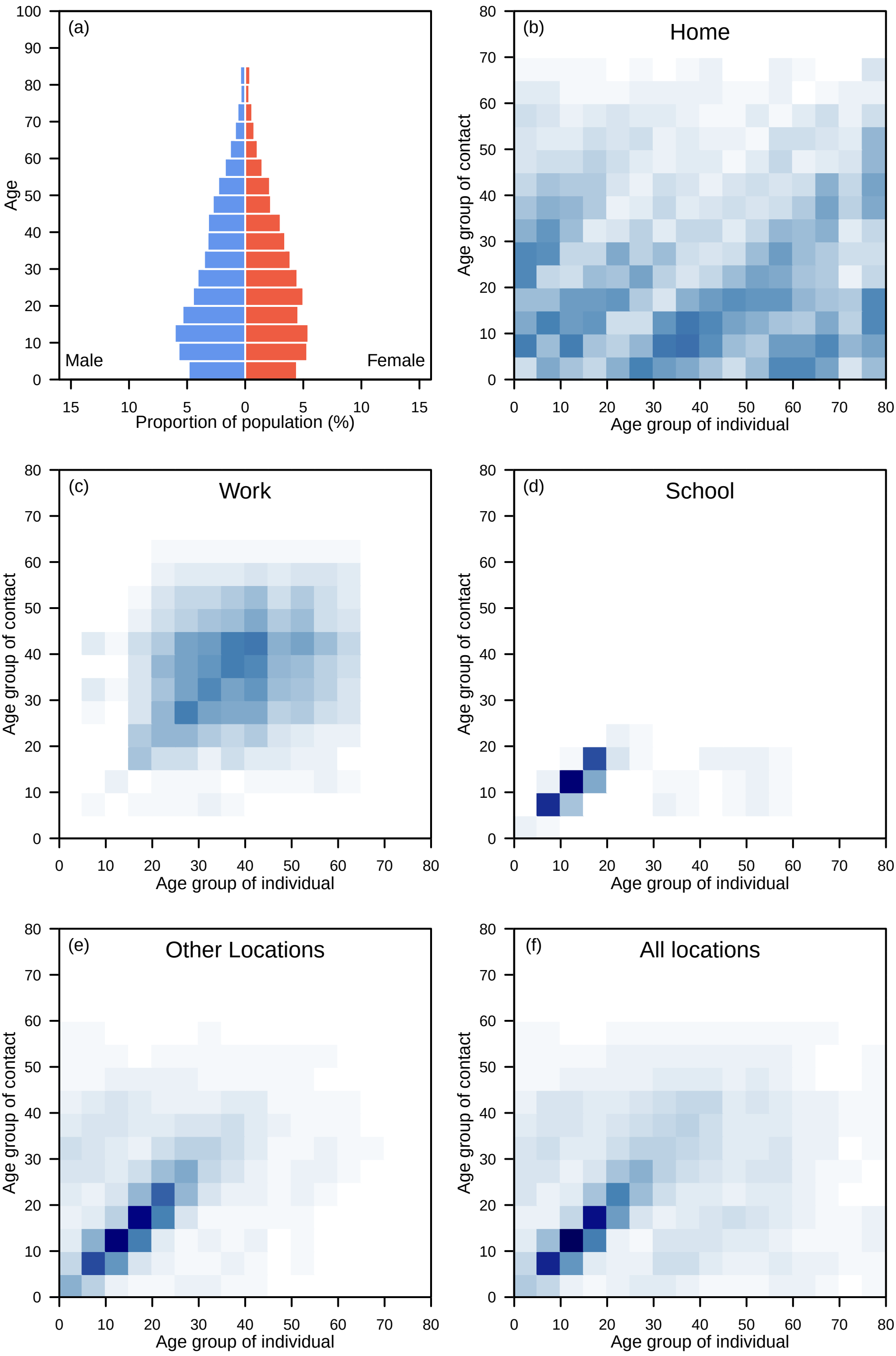
(a)
Age
Male
Female
Proportion of population (%)
(b)
Home
Age group of contact
Age group of individual
(c)
Work
Age group of contact
Age group of individual
(d)
School
Age group of contact
Age group of individual
(e)
Other Locations
Age group of contact
Age group of individual
(f)
All locations
Age group of contact
Age group of individual

Belarus

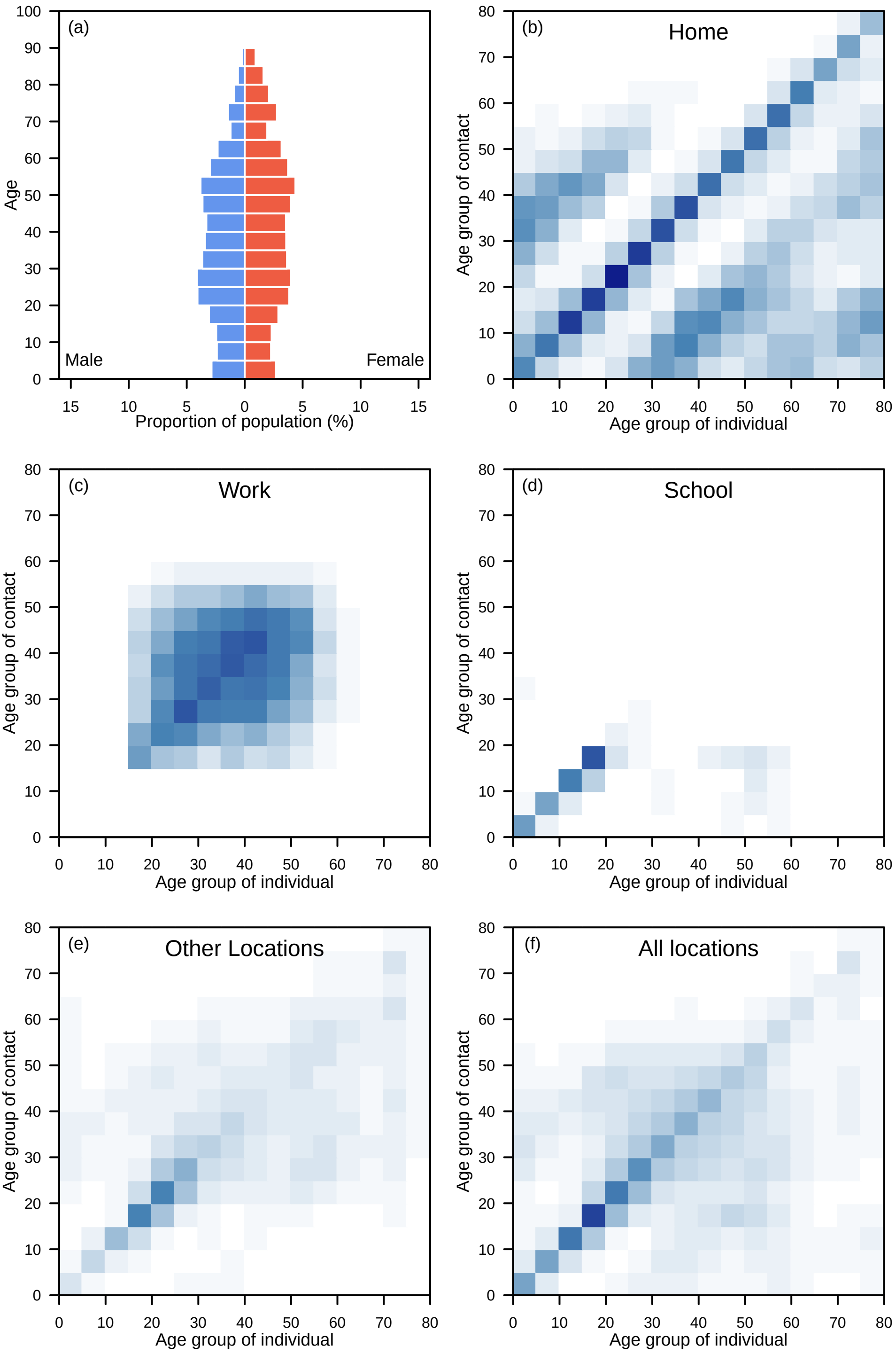

(a)
Male
Female
Age
Proportion of population (%)
(b)
Home
Age group of contact
Age group of individual
(c)
Work
Age group of contact
Age group of individual
(d)
School
Age group of contact
Age group of individual
(e)
Other Locations
Age group of contact
Age group of individual
(f)
All locations
Age group of contact
Age group of individual

Belgium

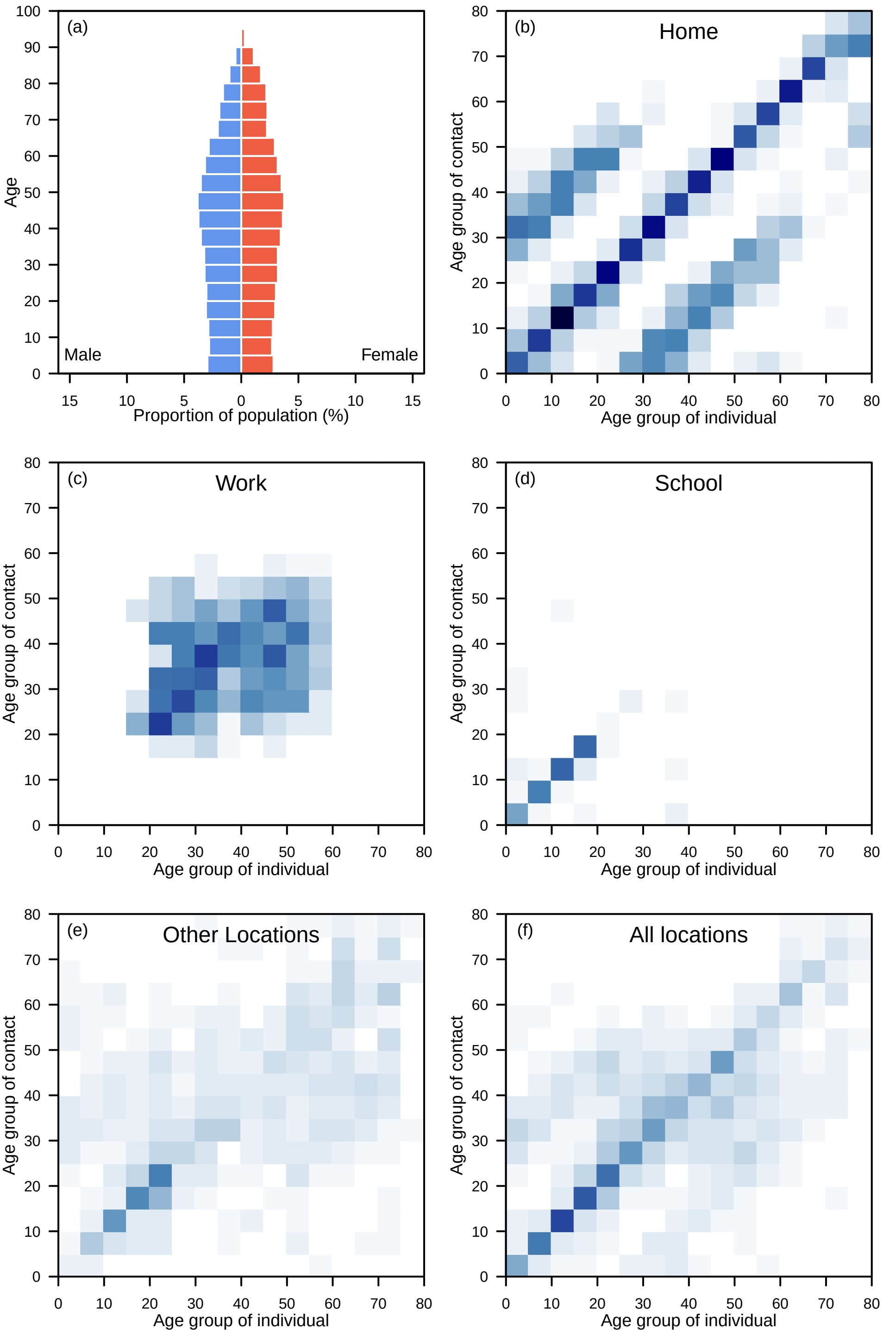

(a)
Age
Male
Female
Proportion of population (%)
(b)
Home
Age group of contact
Age group of individual
(c)
Work
Age group of contact
Age group of individual
(d)
School
Age group of contact
Age group of individual
(e)
Other Locations
Age group of contact
Age group of individual
(f)
All locations
Age group of contact
Age group of individual

Belize

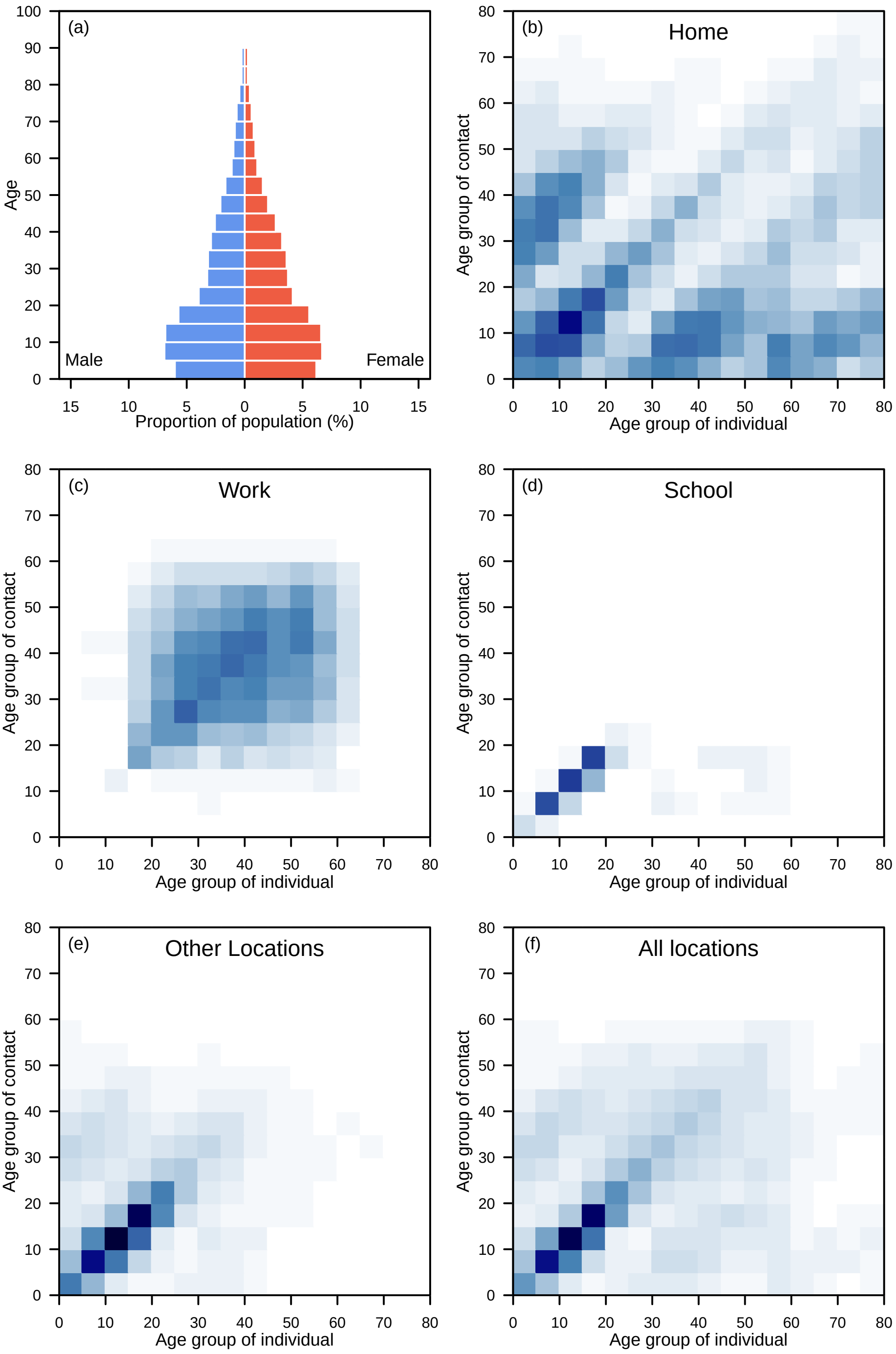

(a)
Age
Male
Female
Proportion of population (%)
(b)
Home
Age group of contact
Age group of individual
(c)
Work
(d)
School
(e)
Other Locations
(f)
All locations

Benin

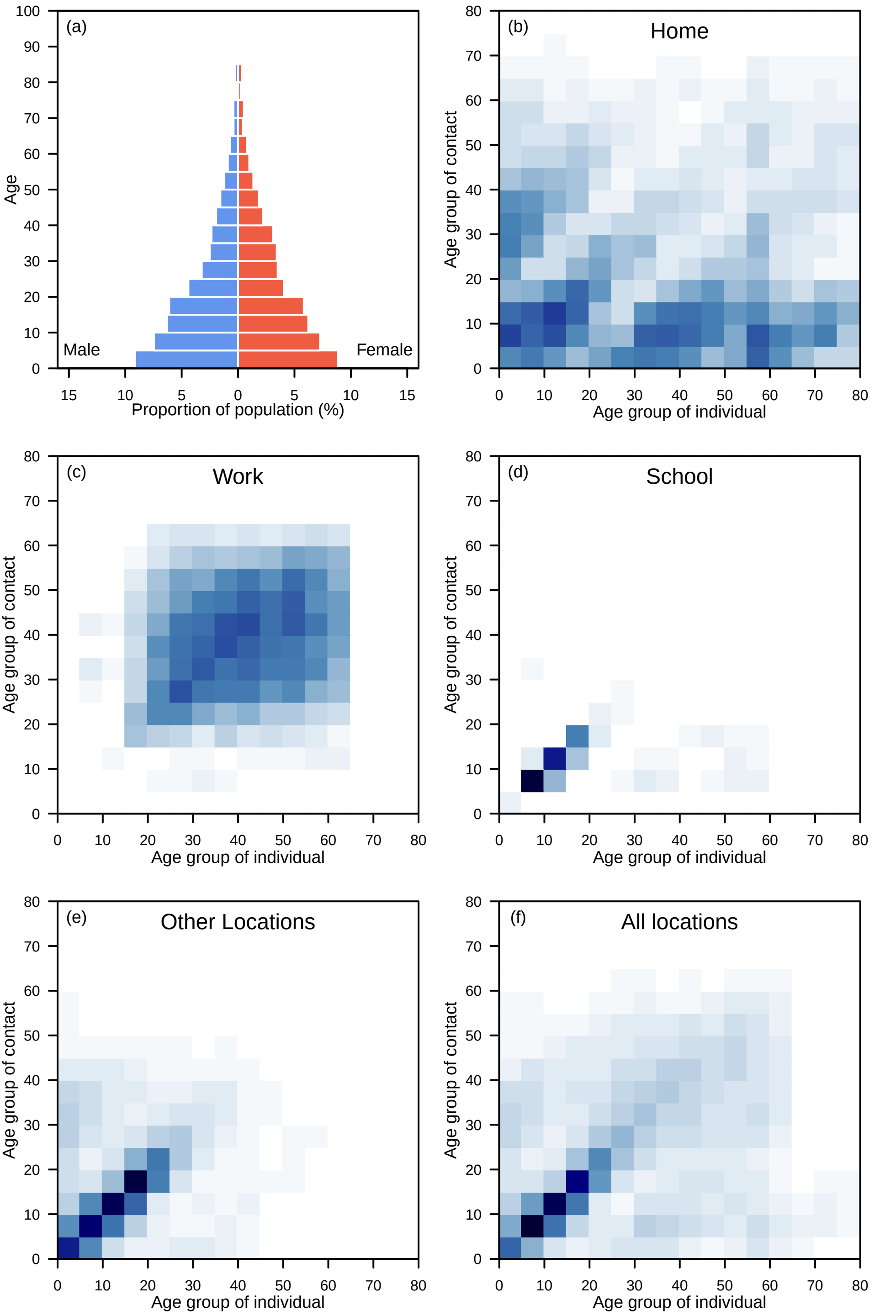

(a)
Age
Male
Female
Proportion of population (%)
(b)
Home
Age group of contact
Age group of individual
(c)
Work
Age group of contact
Age group of individual
(d)
School
Age group of contact
Age group of individual
(e)
Other Locations
Age group of contact
Age group of individual
(f)
All locations
Age group of contact
Age group of individual

Bhutan

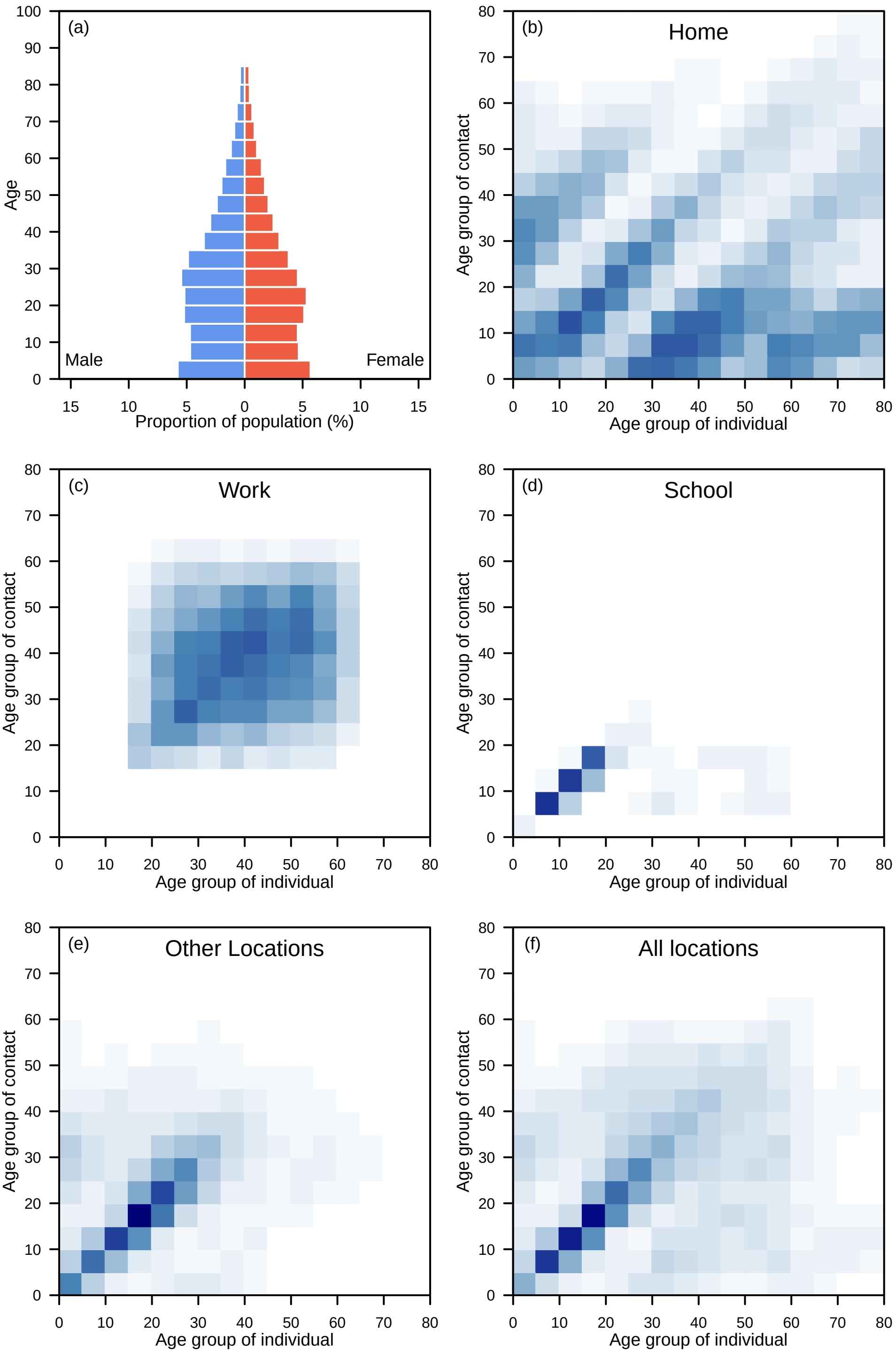

(a)
Age
Male
Female
Proportion of population (%)
(b)
Home
Age group of contact
Age group of individual
(c)
Work
(d)
School
(e)
Other Locations
(f)
All locations

Bolivia (Plurinational State of)

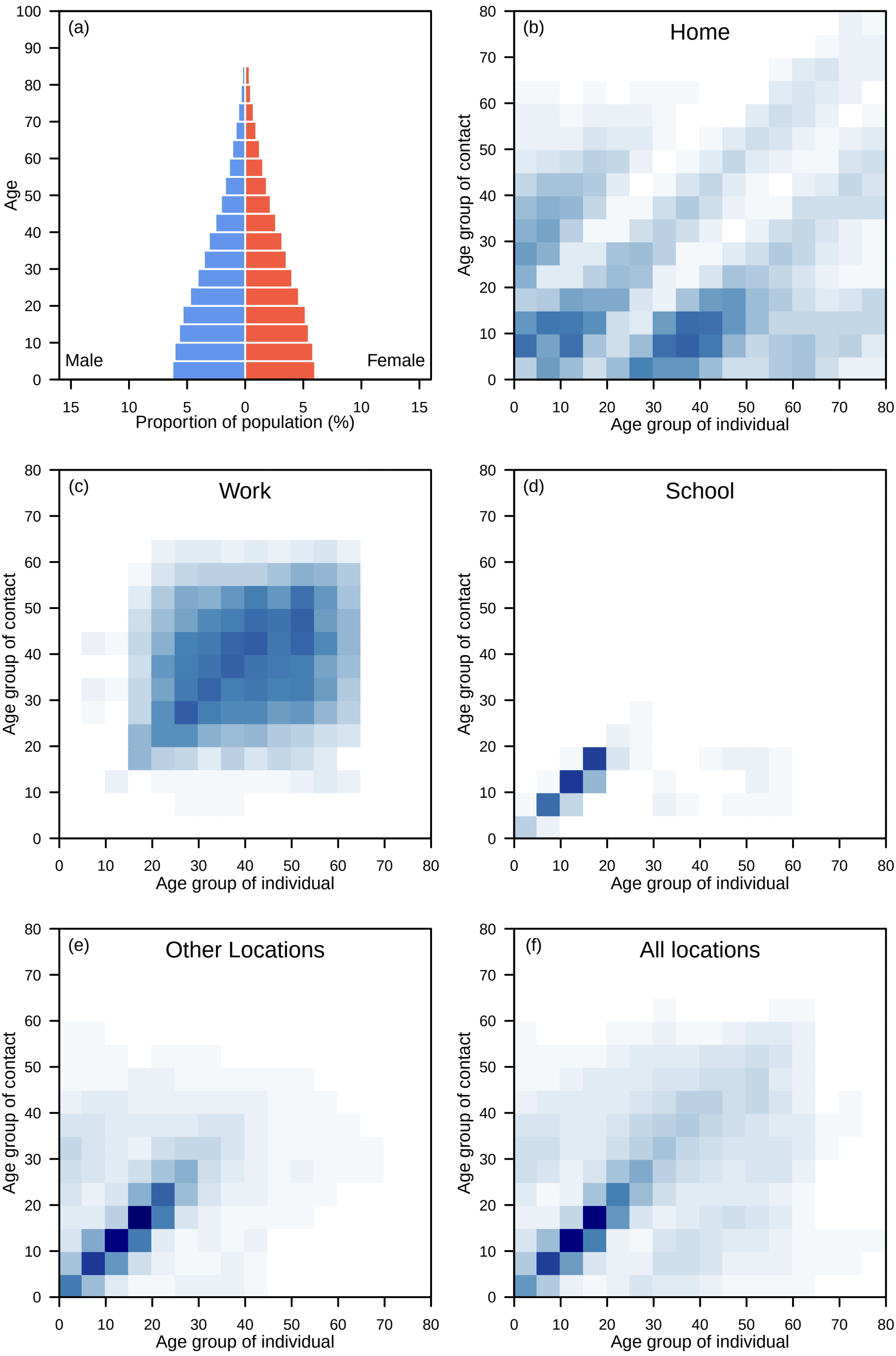

Bosnia and Herzegovina

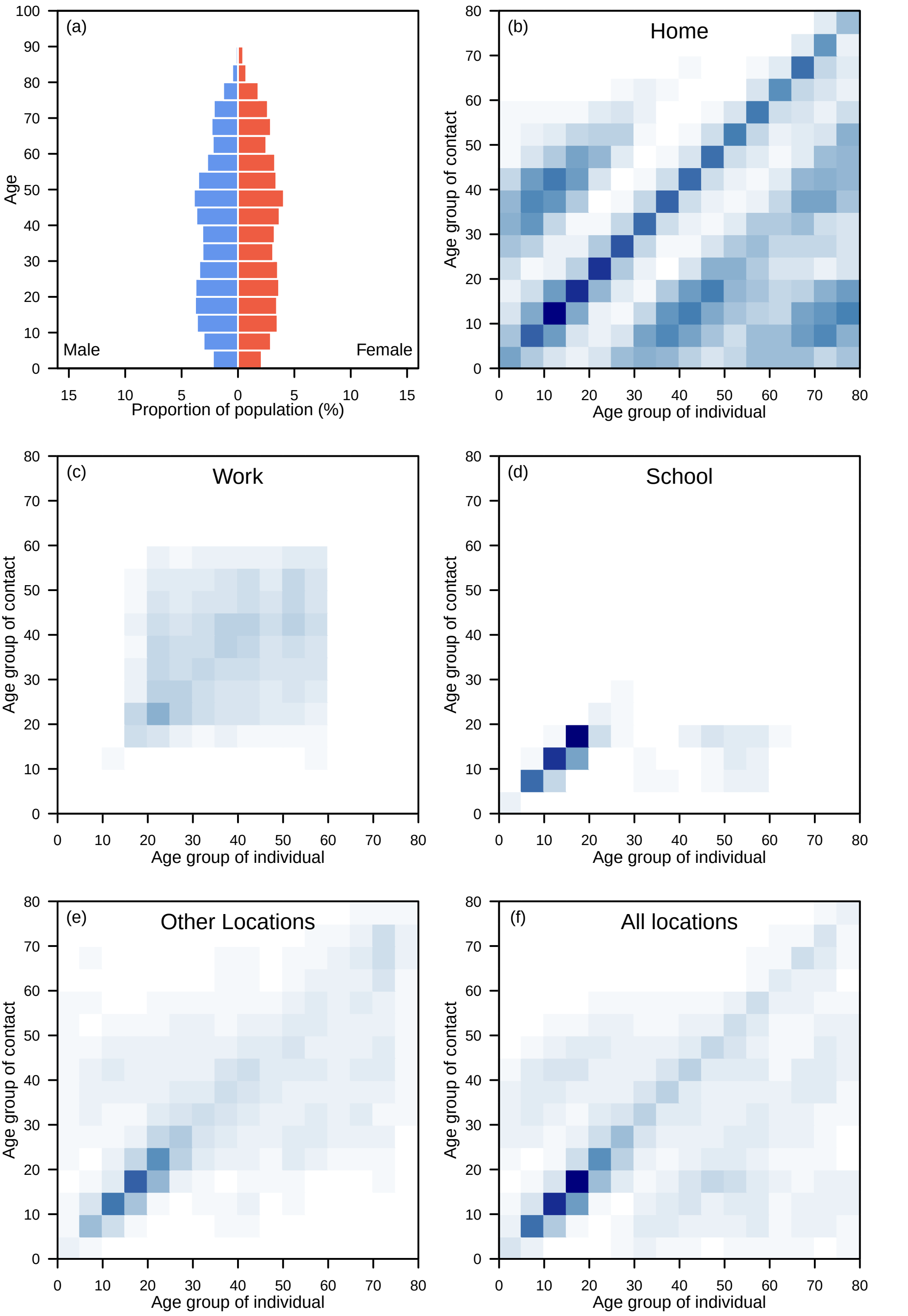

Botswana

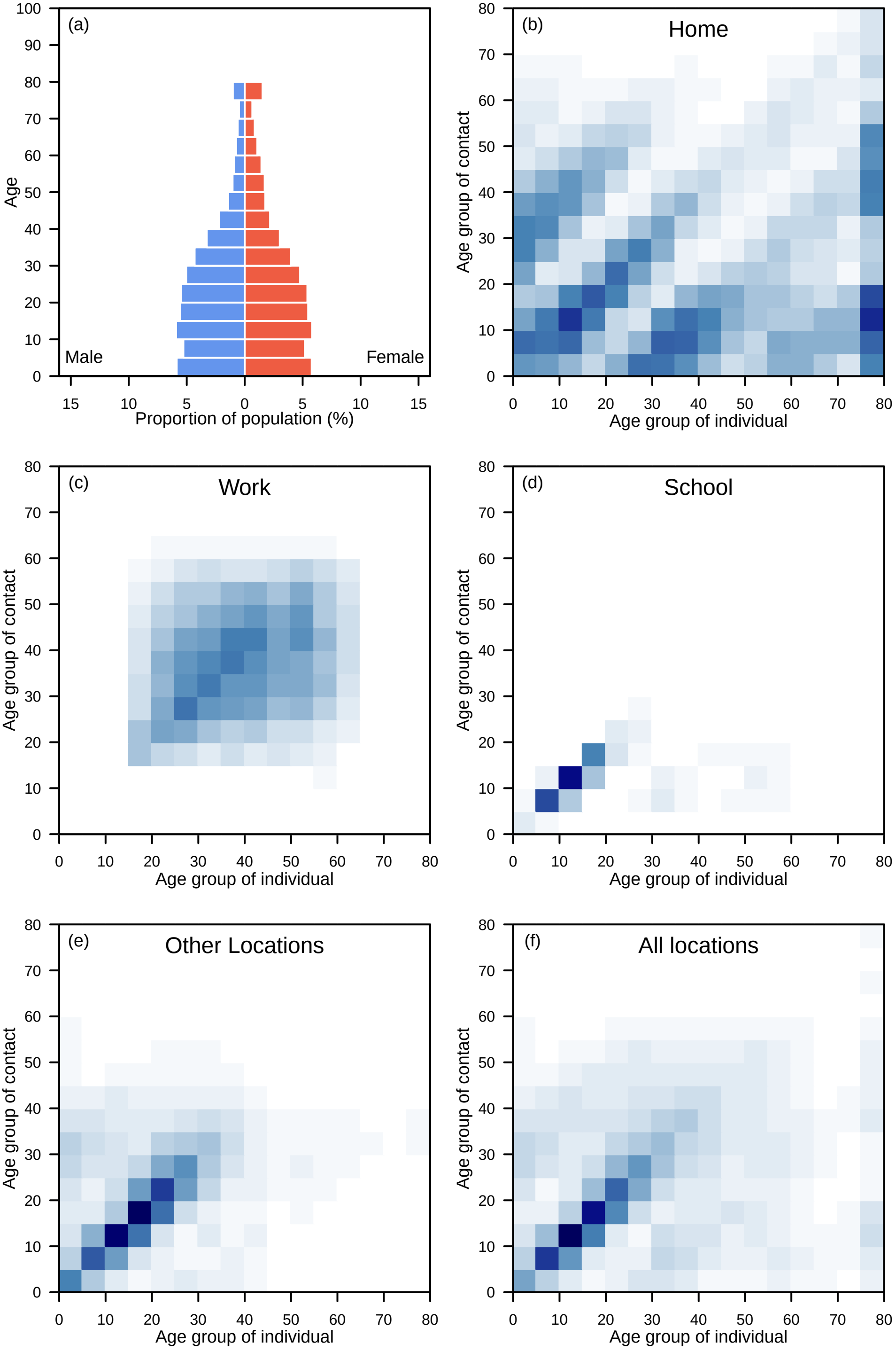

(a)
Age
Male
Female
Proportion of population (%)
(b)
Home
(c)
Work
(d)
School
(e)
Other Locations
(f)
All locations
Age group of contact
Age group of individual

Brazil

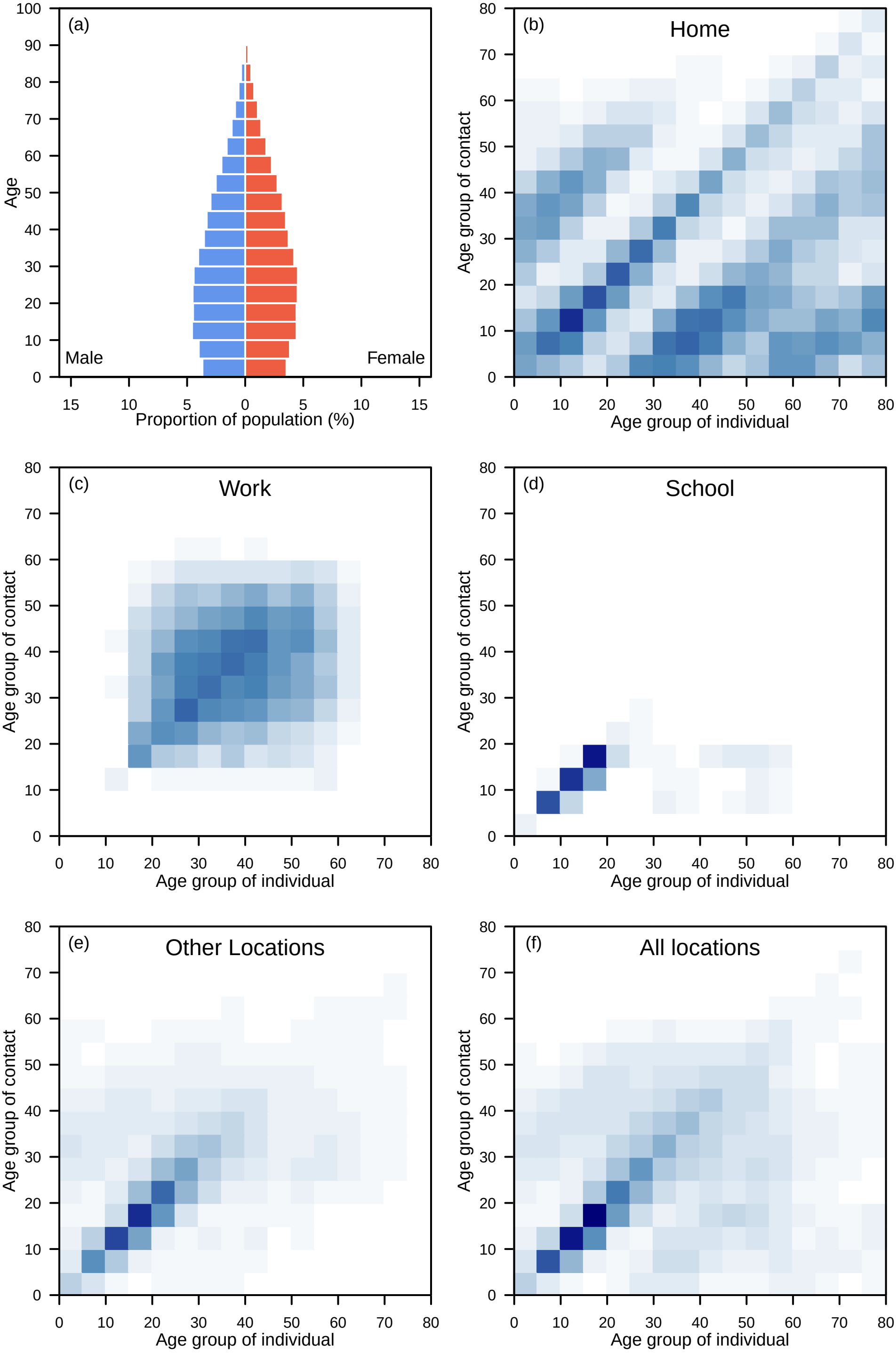

(a)
Age
Male
Female
Proportion of population (%)
(b)
Home
Age group of contact
Age group of individual
(c)
Work
Age group of contact
Age group of individual
(d)
School
Age group of contact
Age group of individual
(e)
Other Locations
Age group of contact
Age group of individual
(f)
All locations
Age group of contact
Age group of individual

Brunei Darussalam

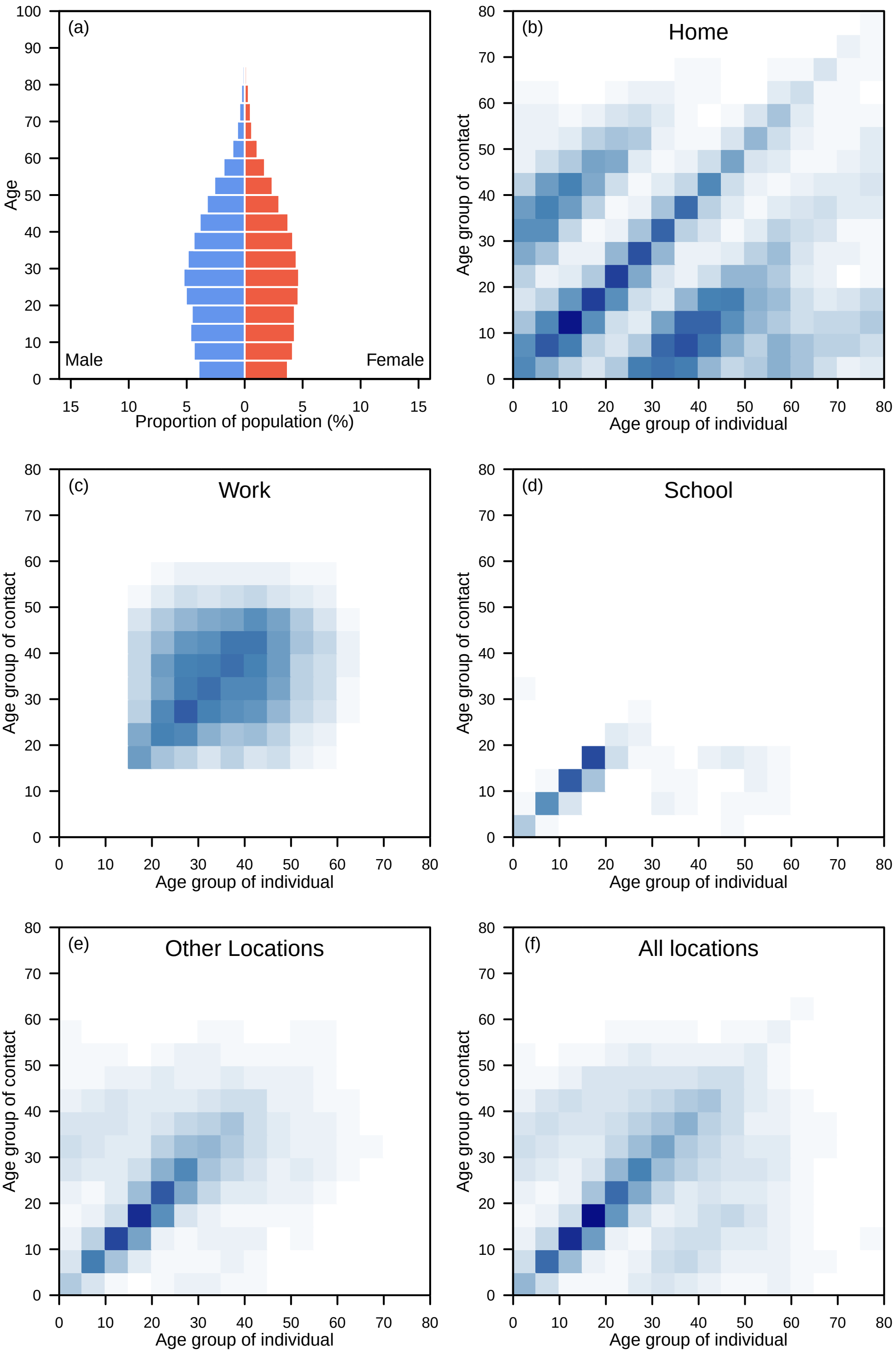

Bulgaria

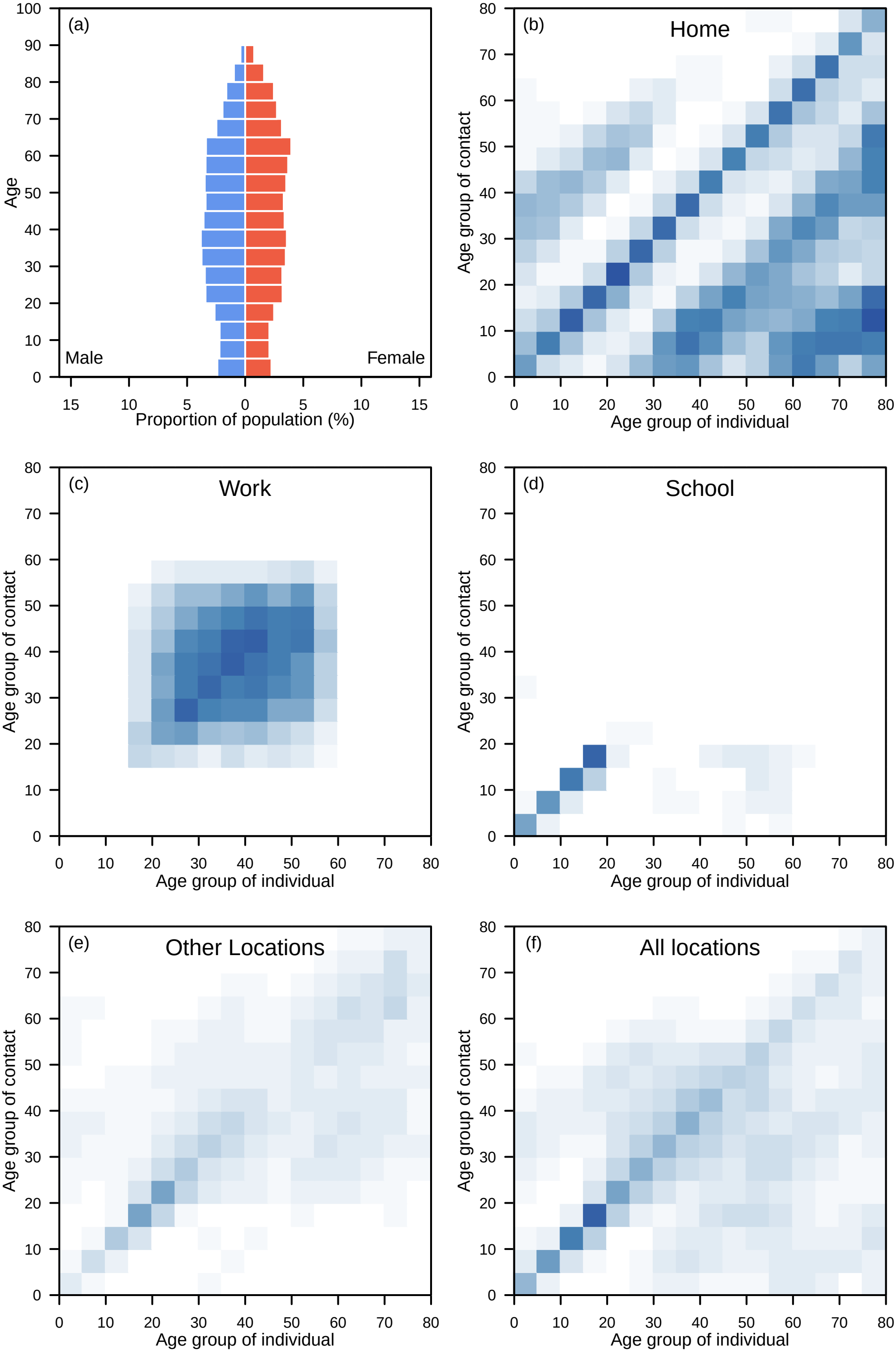

(a)
Age
100
90
80
70
60
50
40
30
20
10
0
Male
Female
15
10
5
0
5
10
15
Proportion of population (%)
(b)
Home
Age group of contact
Age group of individual
(c)
Work
Age group of contact
Age group of individual
(d)
School
Age group of contact
Age group of individual
(e)
Other Locations
Age group of contact
Age group of individual
(f)
All locations
Age group of contact
Age group of individual
0
10
20
30
40
50
60
70
80

Burkina Faso

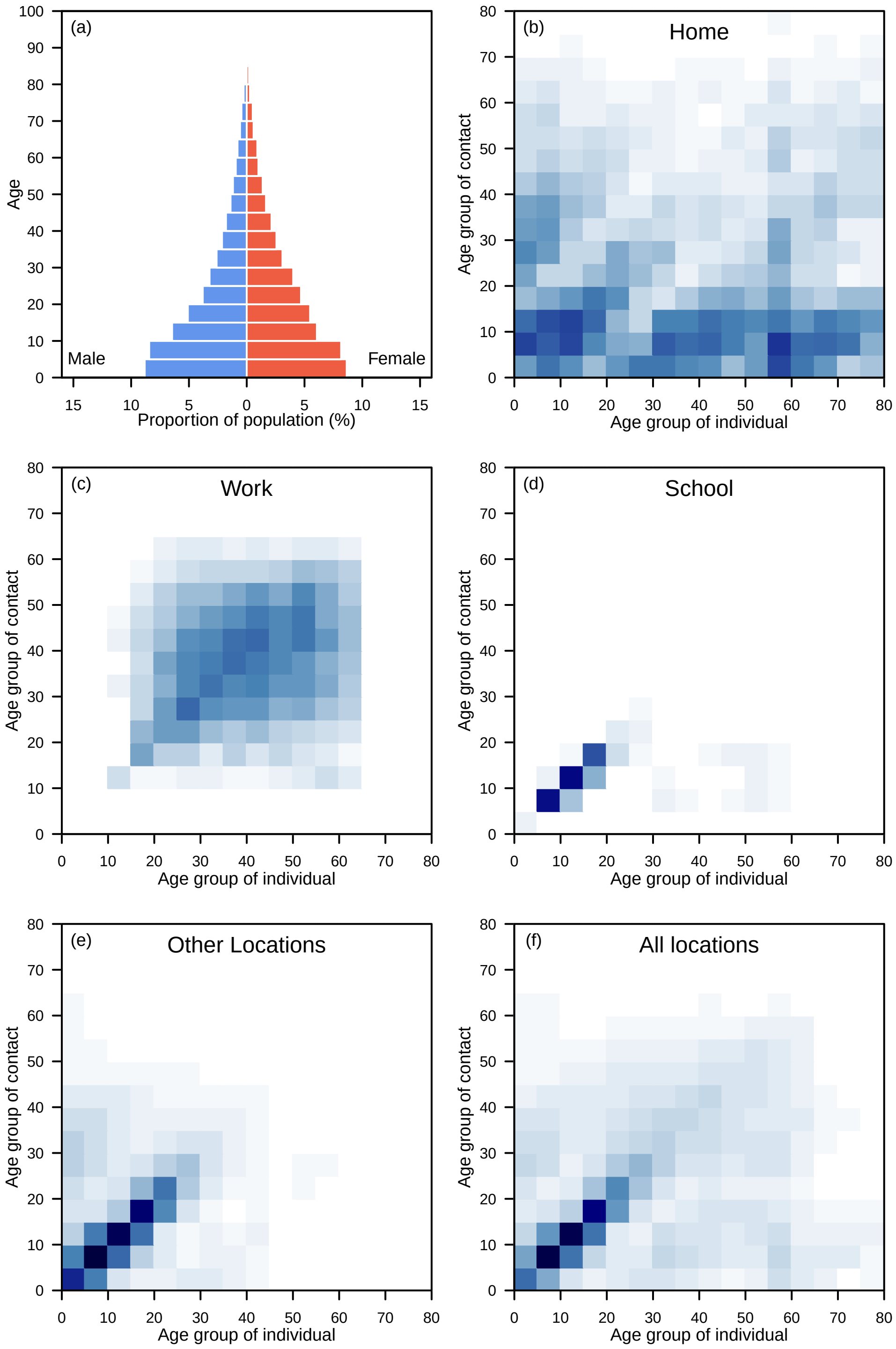

Cabo Verde

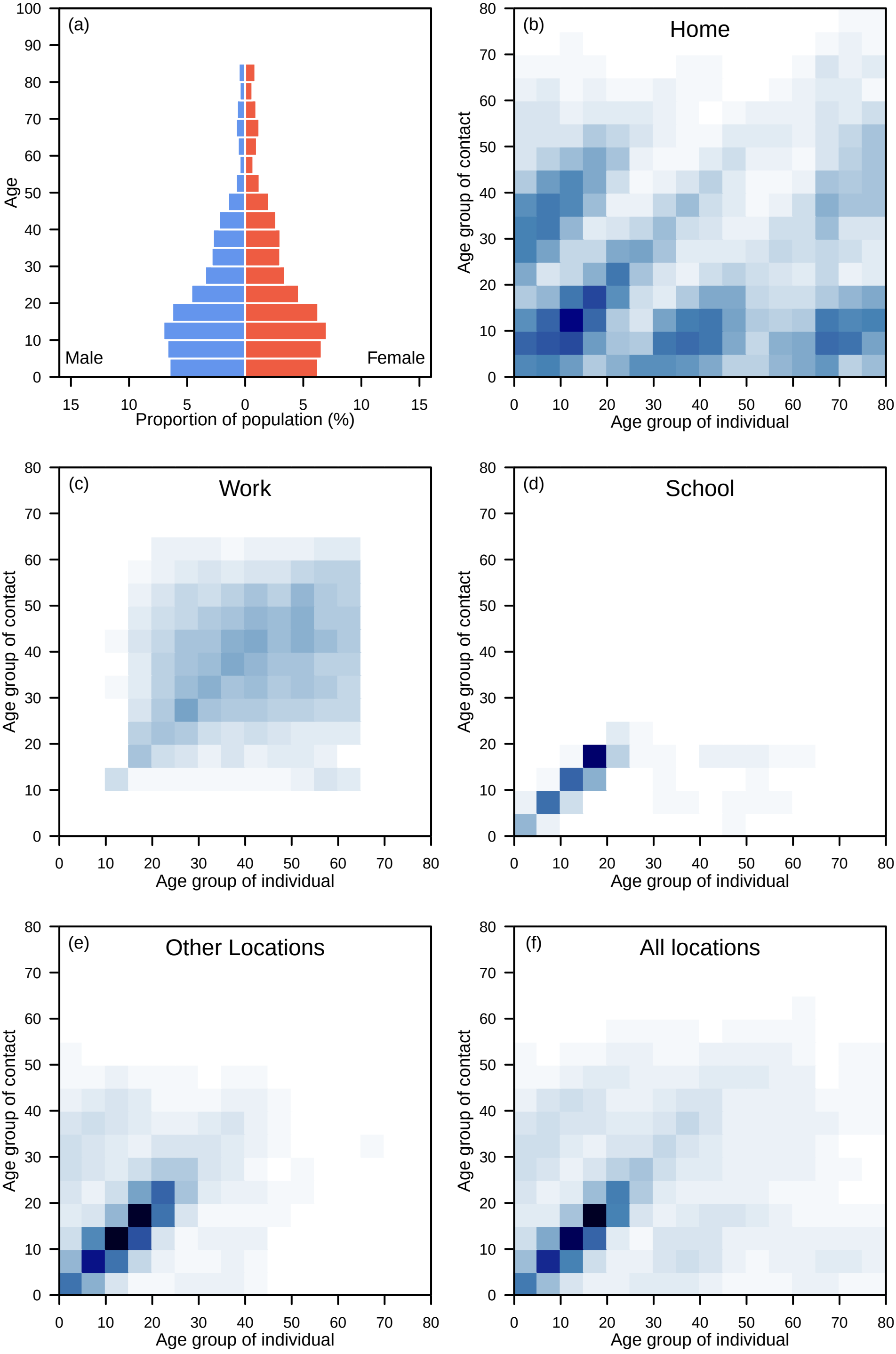

(a)
Age
Male
Female
Proportion of population (%)
(b)
Home
Age group of contact
Age group of individual
(c)
Work
Age group of contact
Age group of individual
(d)
School
Age group of contact
Age group of individual
(e)
Other Locations
Age group of contact
Age group of individual
(f)
All locations
Age group of contact
Age group of individual

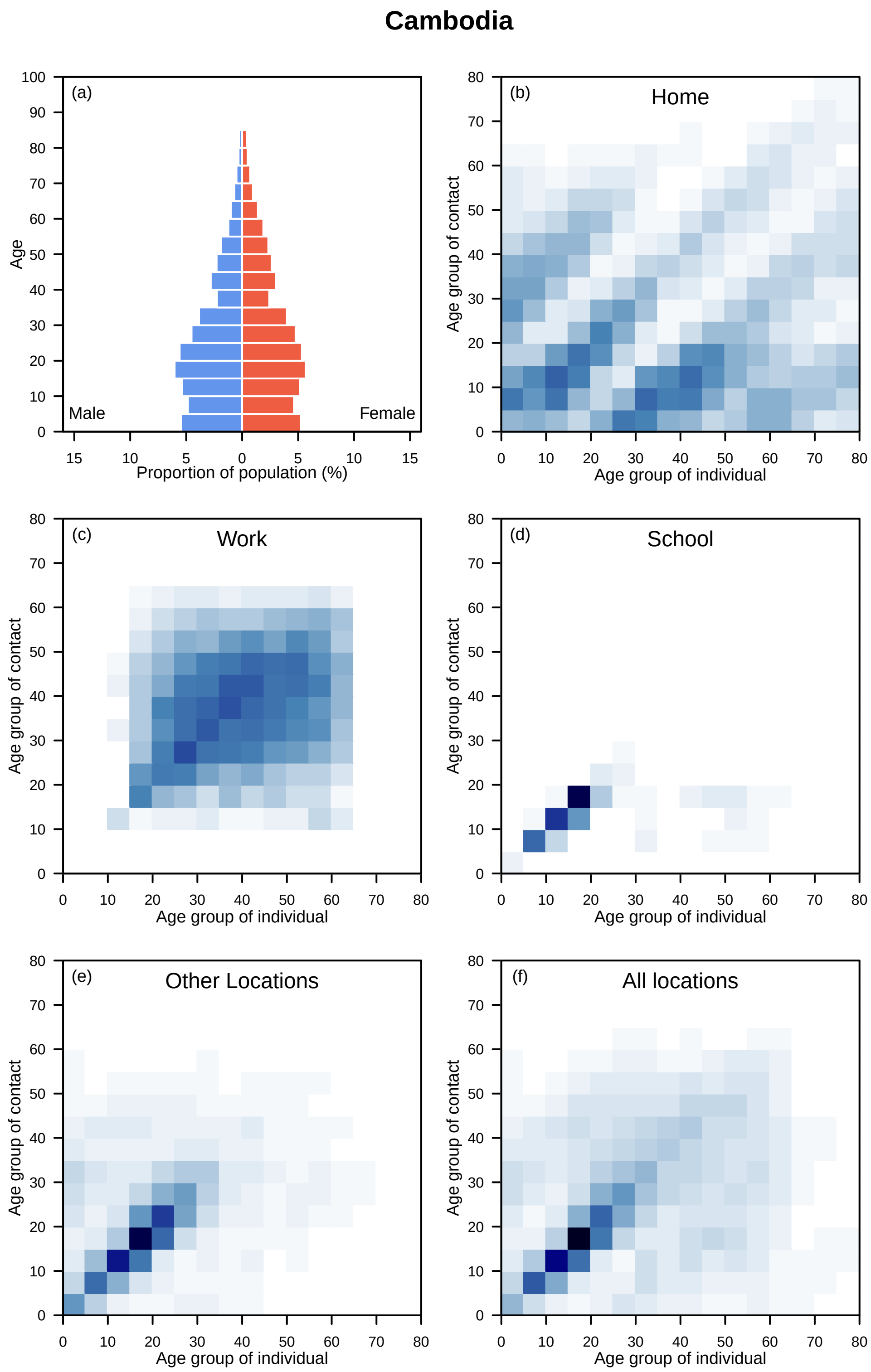
Cambodia
(a)
Age
Male
Female
Proportion of population (%)
(b) Home
(c) Work
(d) School
(e) Other Locations
(f) All locations
Age group of contact
Age group of individual

Cameroon

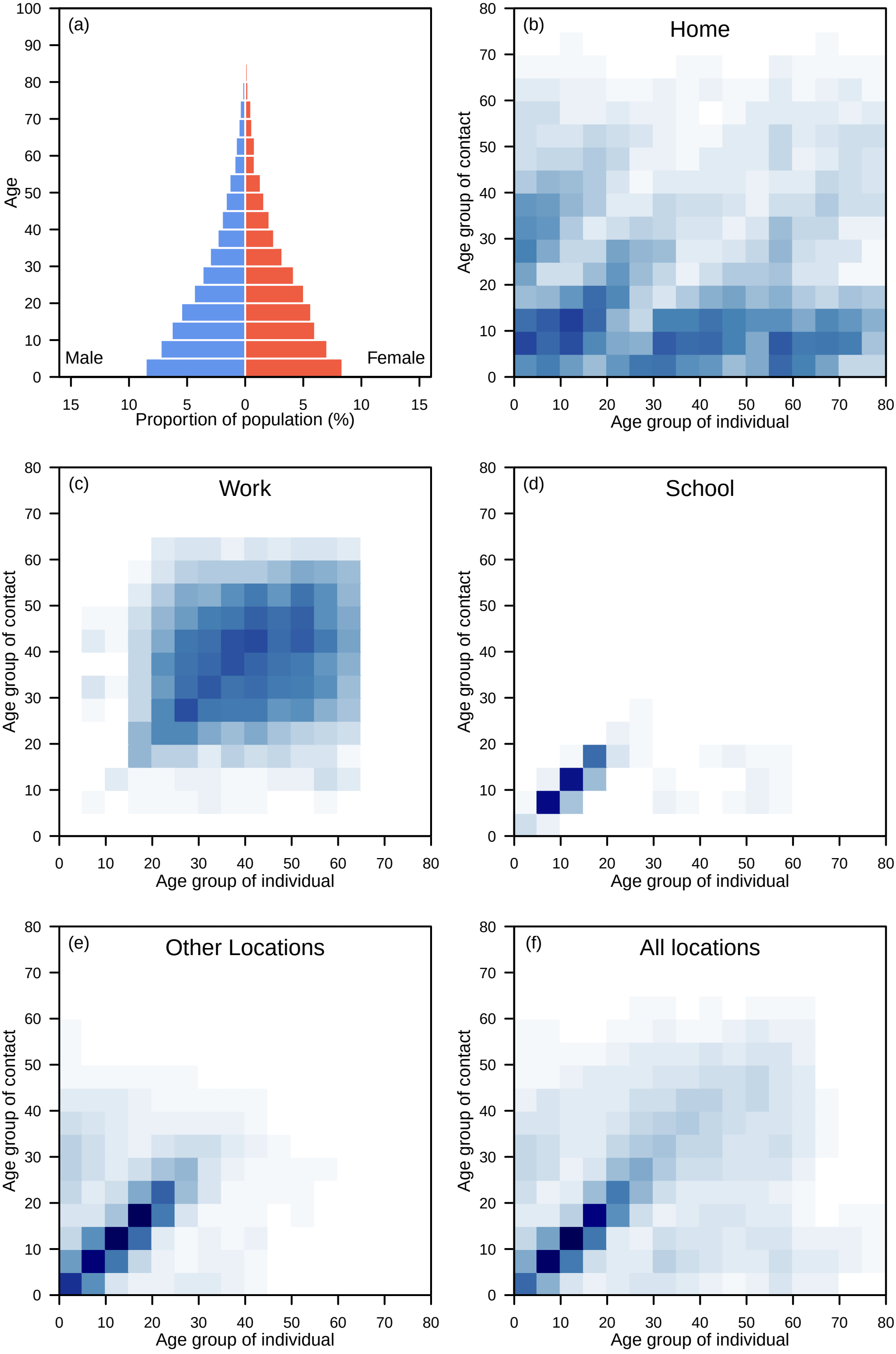

(a)
Age
Male
Female
Proportion of population (%)
(b)
Home
Age group of contact
Age group of individual
(c)
Work
(d)
School
(e)
Other Locations
(f)
All locations

Canada

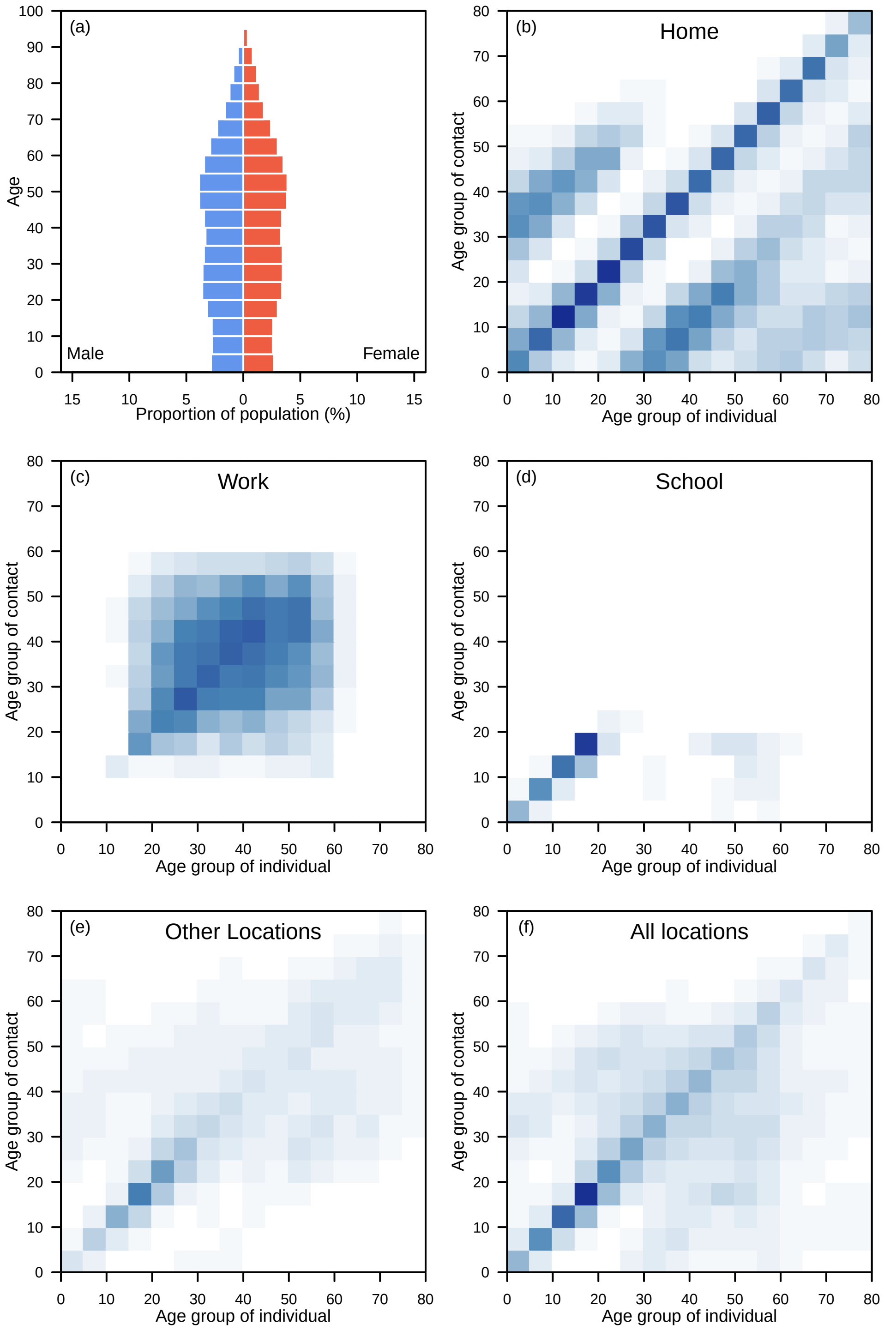

(a)
Age
Male
Female
Proportion of population (%)
(b)
Home
Age group of contact
Age group of individual
(c)
Work
Age group of contact
Age group of individual
(d)
School
Age group of contact
Age group of individual
(e)
Other Locations
Age group of contact
Age group of individual
(f)
All locations
Age group of contact
Age group of individual

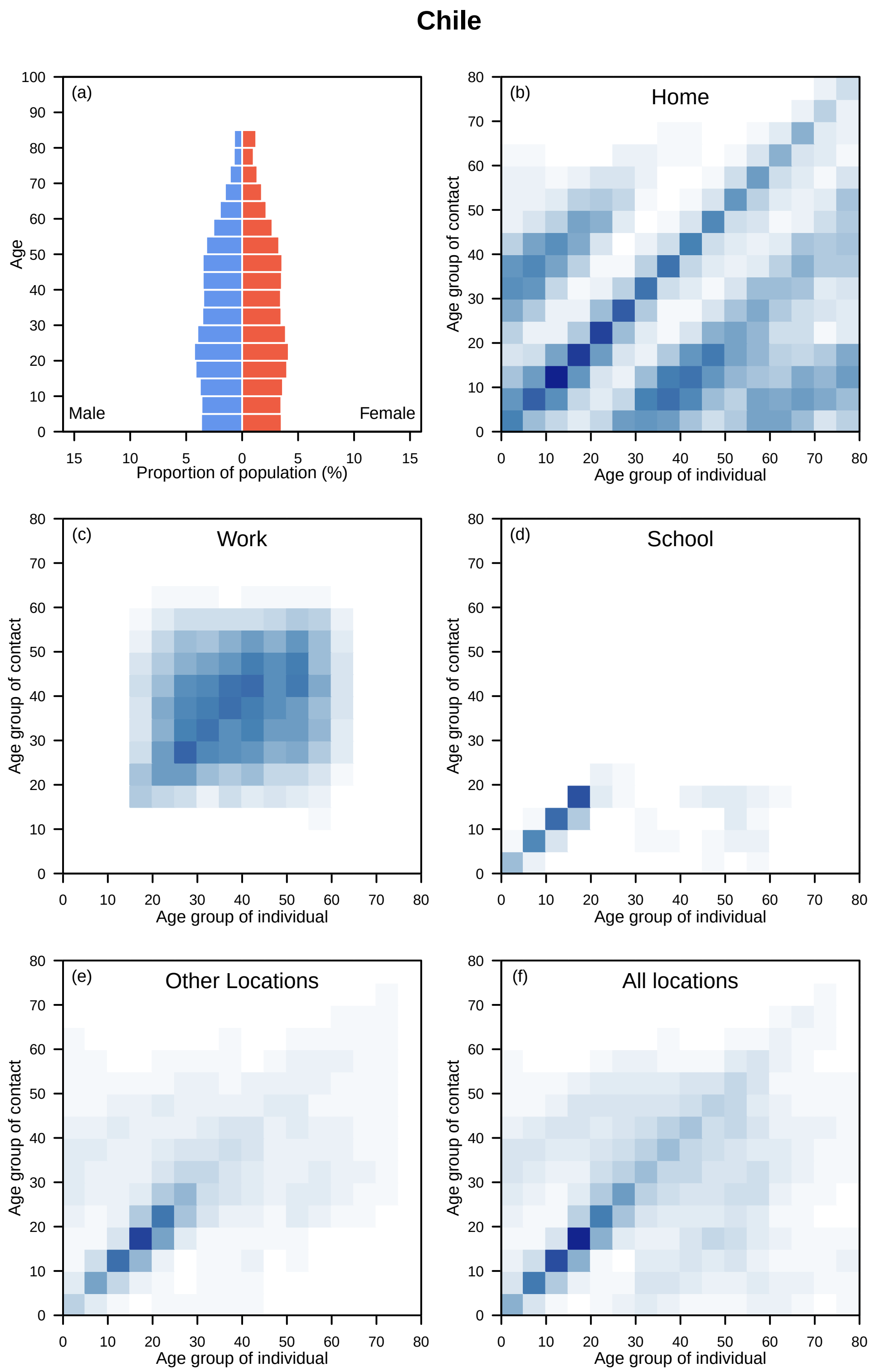
Chile
(a)
Male
Female
Age
Proportion of population (%)
(b)
Home
(c)
Work
(d)
School
(e)
Other Locations
(f)
All locations
Age group of contact
Age group of individual

China

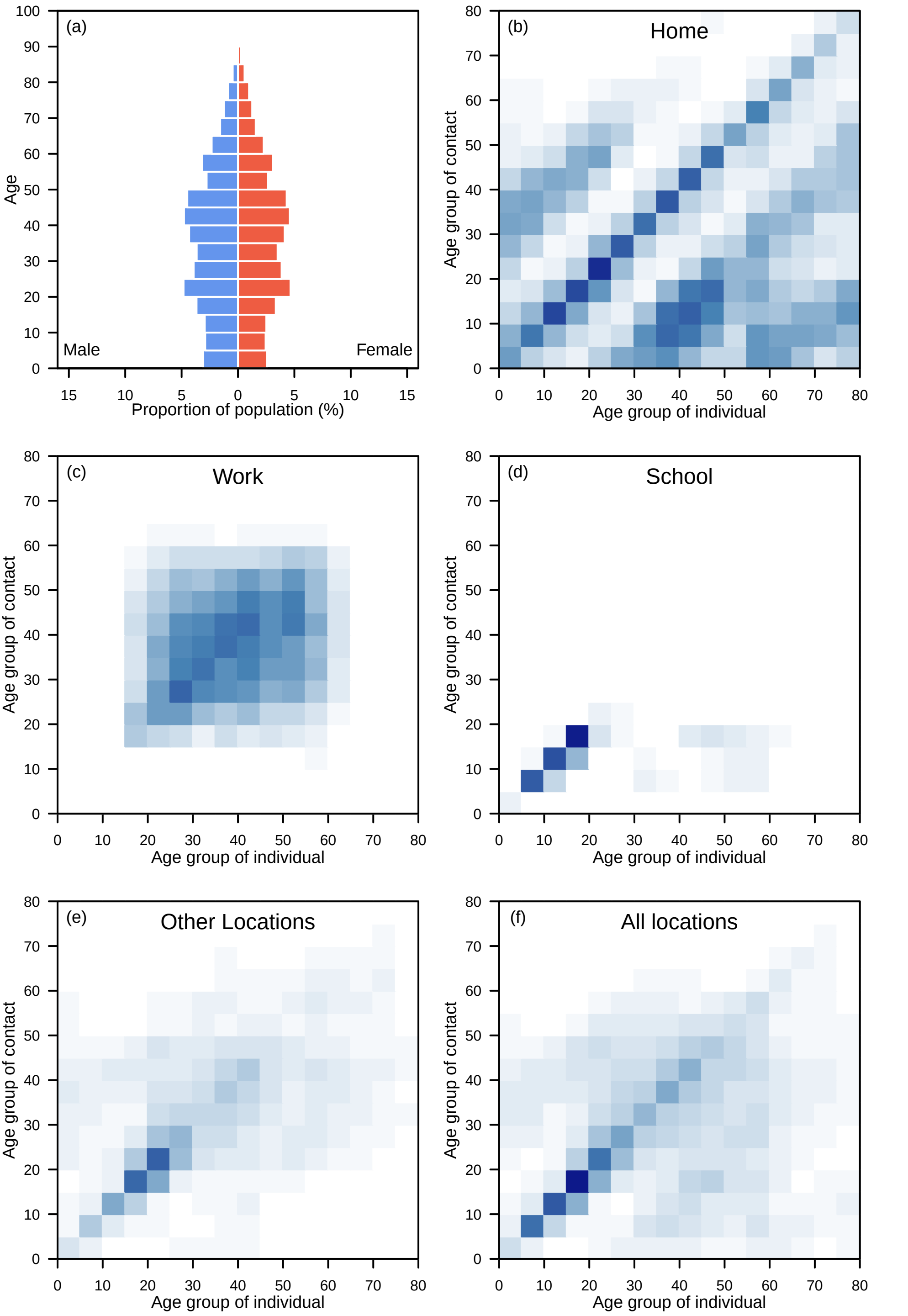

(a)
Age
Male
Female
Proportion of population (%)
(b)
Home
Age group of contact
Age group of individual
(c)
Work
Age group of contact
Age group of individual
(d)
School
Age group of contact
Age group of individual
(e)
Other Locations
Age group of contact
Age group of individual
(f)
All locations
Age group of contact
Age group of individual

Colombia

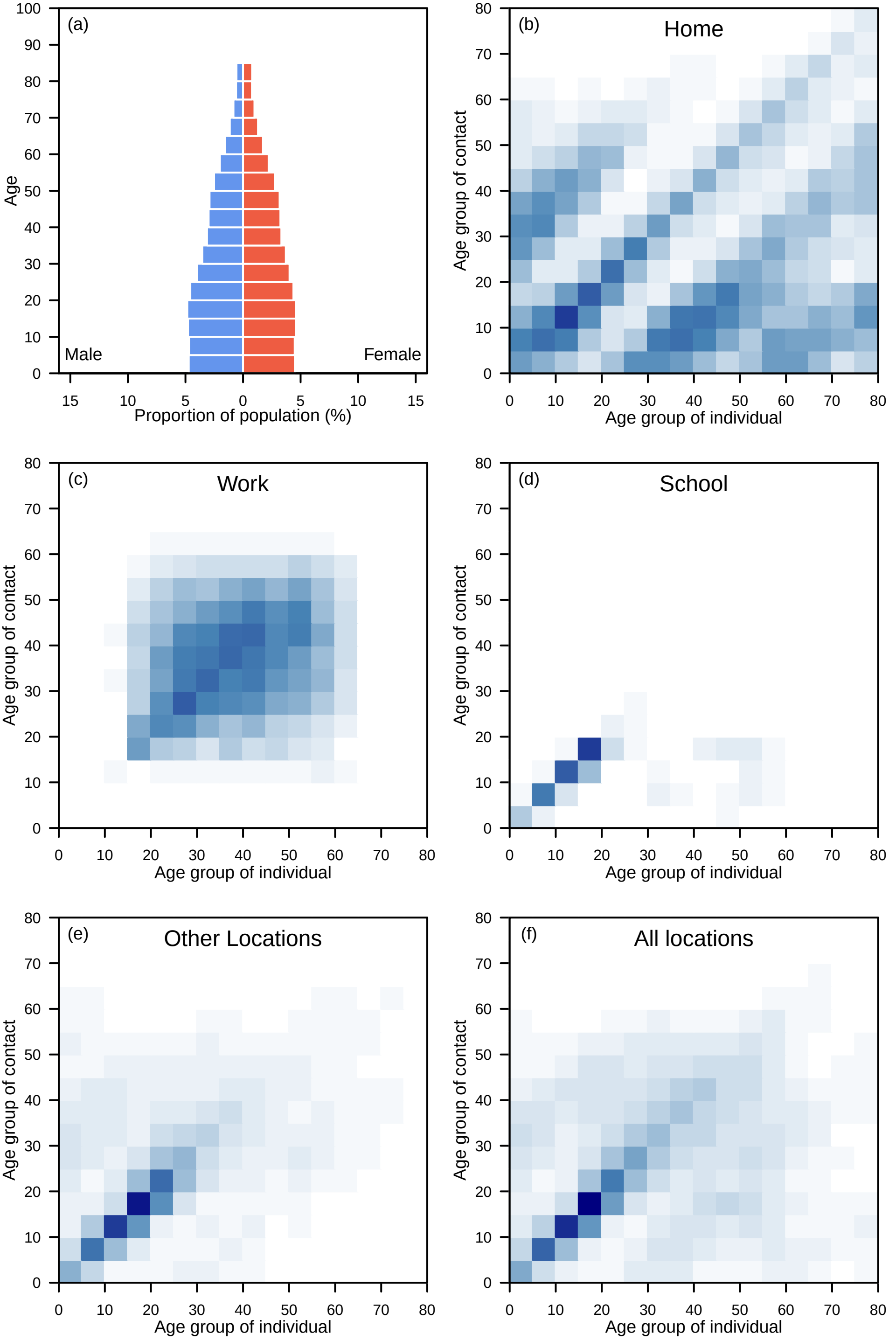
(a)
Age
Male
Female
Proportion of population (%)
(b)
Home
Age group of contact
Age group of individual
(c)
Work
(d)
School
(e)
Other Locations
(f)
All locations

Congo

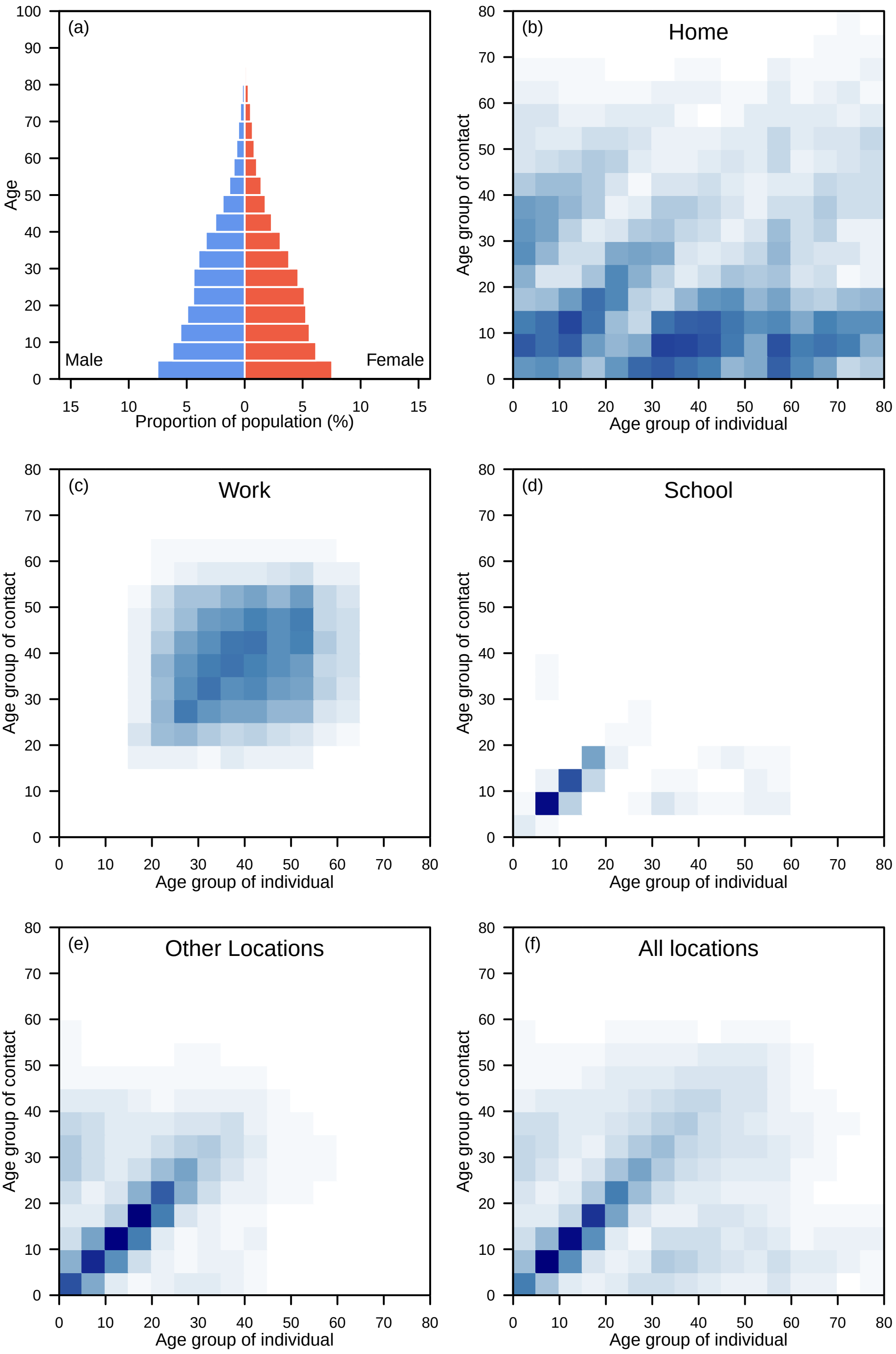

(a)
Age
Male
Female
Proportion of population (%)
(b)
Home
Age group of contact
Age group of individual
(c)
Work
Age group of contact
Age group of individual
(d)
School
Age group of contact
Age group of individual
(e)
Other Locations
Age group of contact
Age group of individual
(f)
All locations
Age group of contact
Age group of individual

Costa Rica

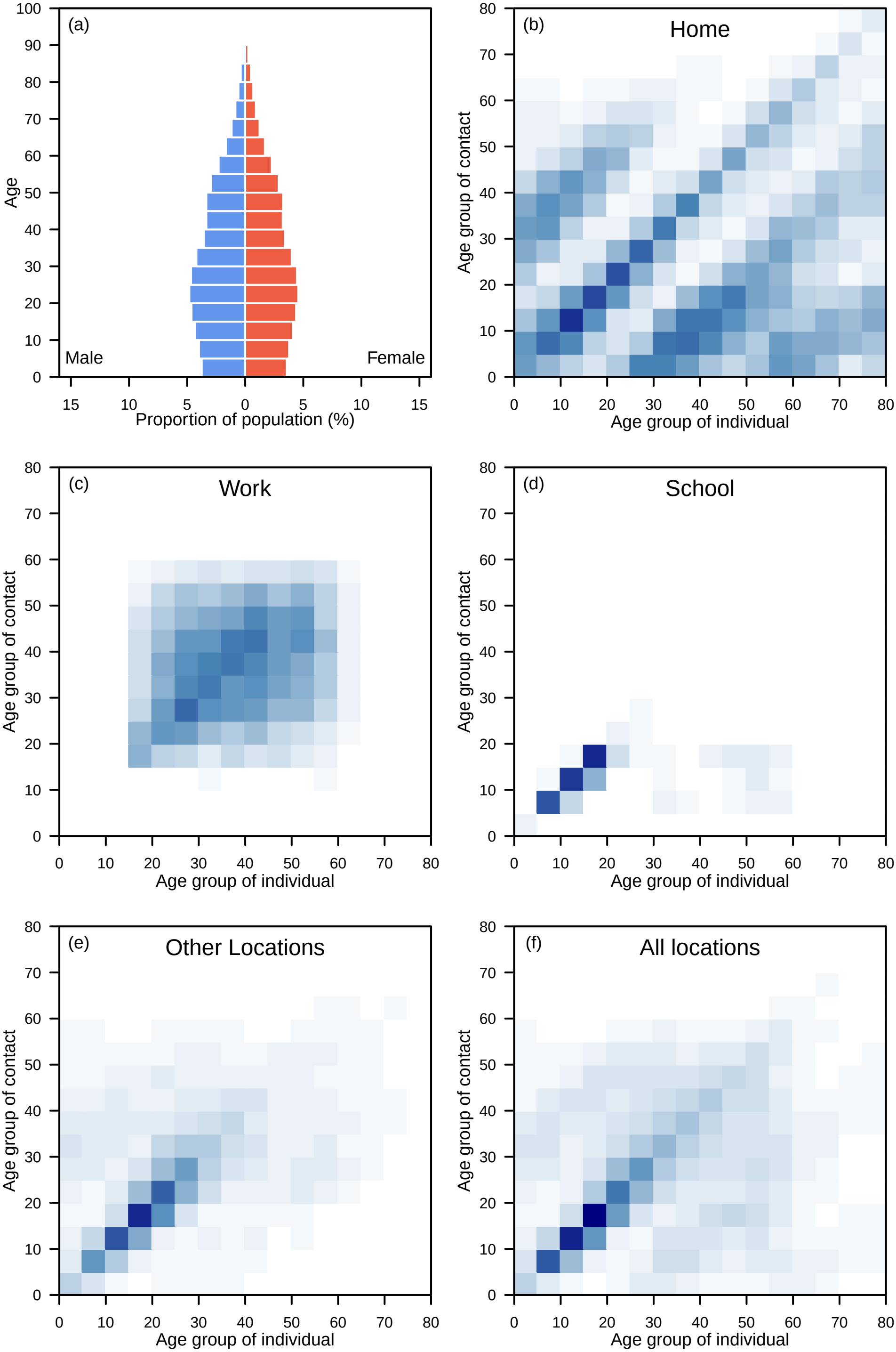

(a)
Male
Female
Age
Proportion of population (%)
(b)
Home
(c)
Work
(d)
School
(e)
Other Locations
(f)
All locations
Age group of contact
Age group of individual

Croatia

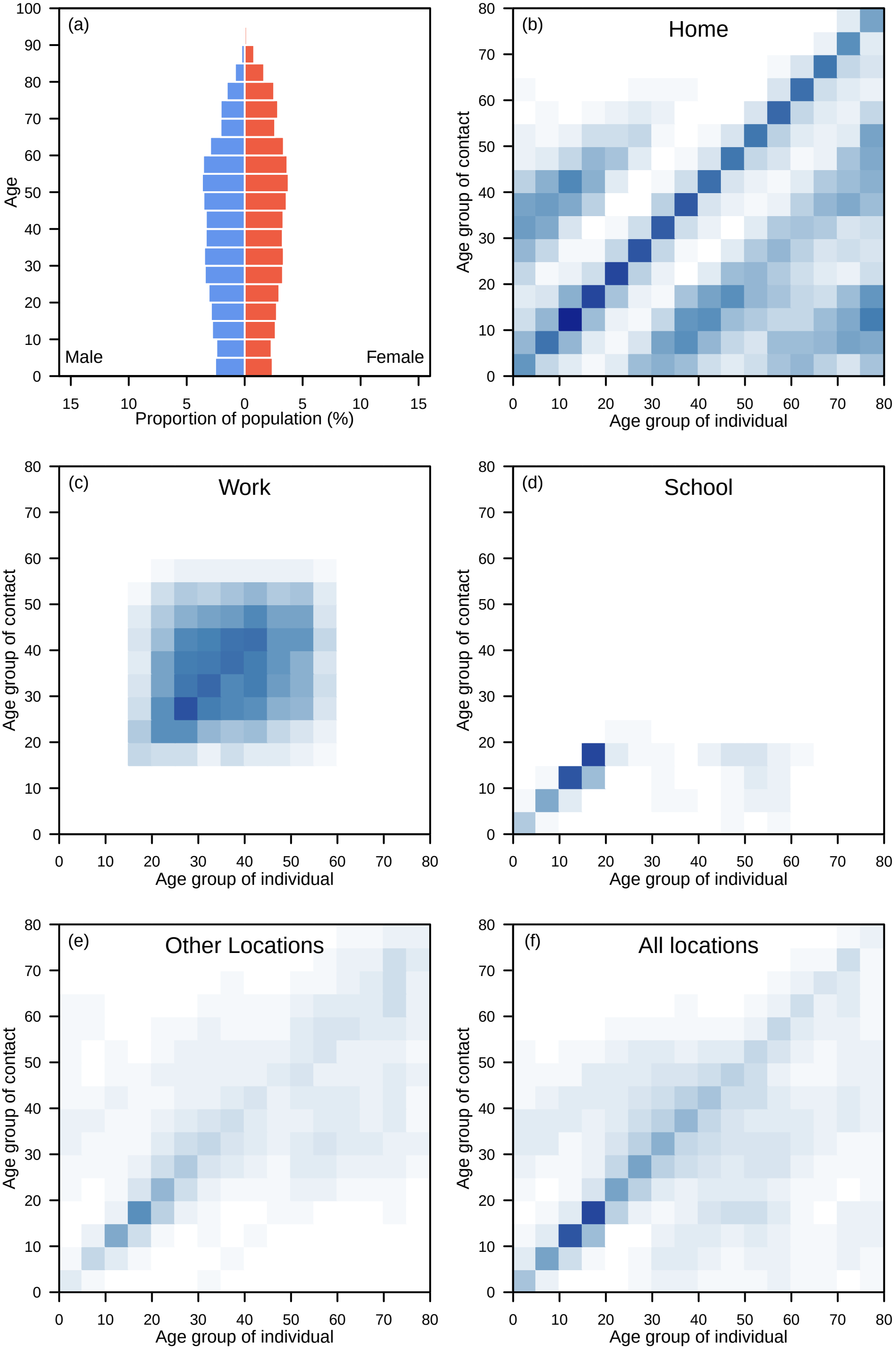

(a)
Male
Female
Age
Proportion of population (%)
(b)
Home
Age group of contact
Age group of individual
(c)
Work
Age group of contact
Age group of individual
(d)
School
Age group of contact
Age group of individual
(e)
Other Locations
Age group of contact
Age group of individual
(f)
All locations
Age group of contact
Age group of individual

Cyprus

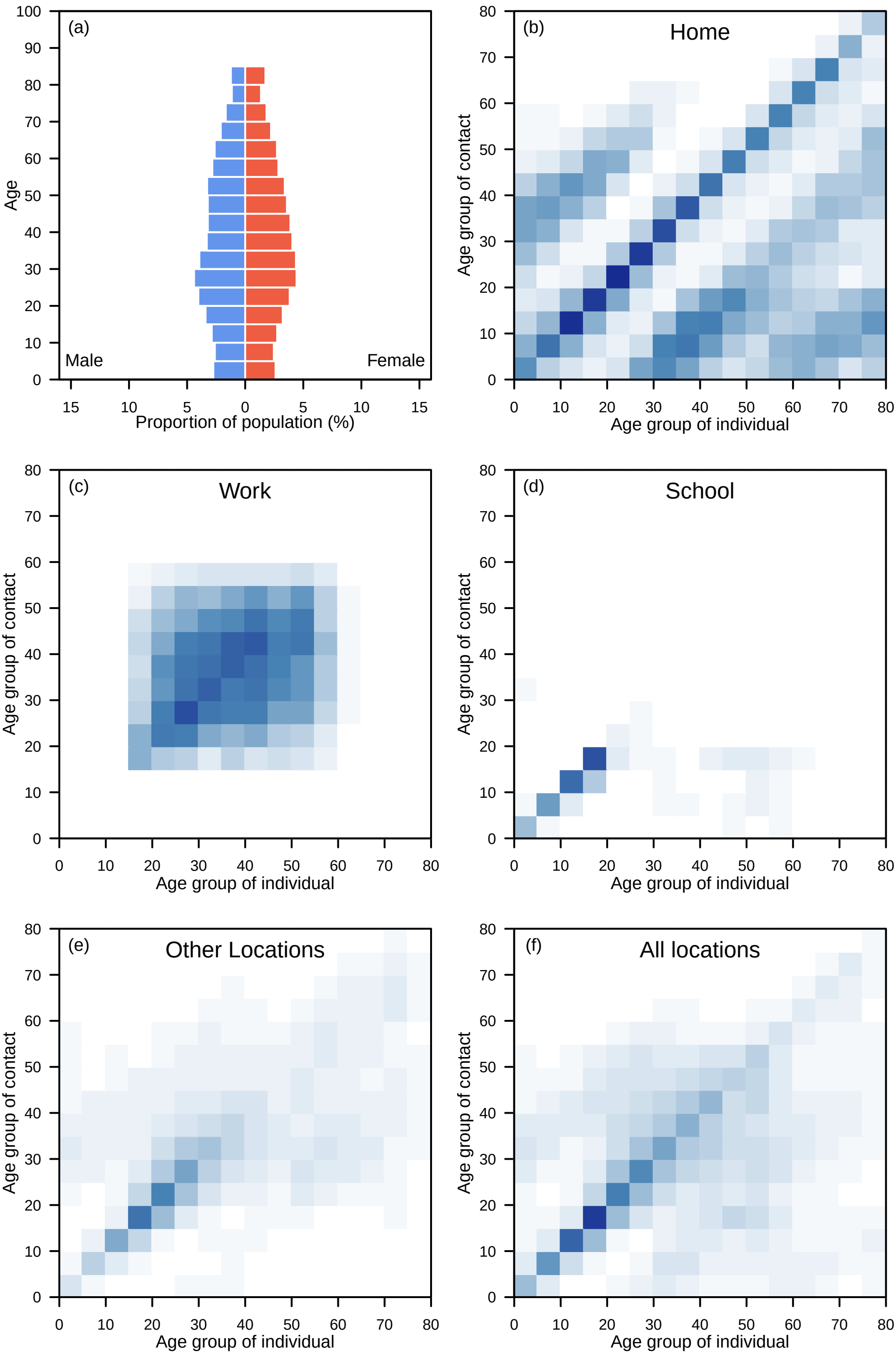

(a)
Age
Male
Female
Proportion of population (%)
(b)
Home
Age group of contact
Age group of individual
(c)
Work
Age group of contact
Age group of individual
(d)
School
Age group of contact
Age group of individual
(e)
Other Locations
Age group of contact
Age group of individual
(f)
All locations
Age group of contact
Age group of individual

Czech Republic

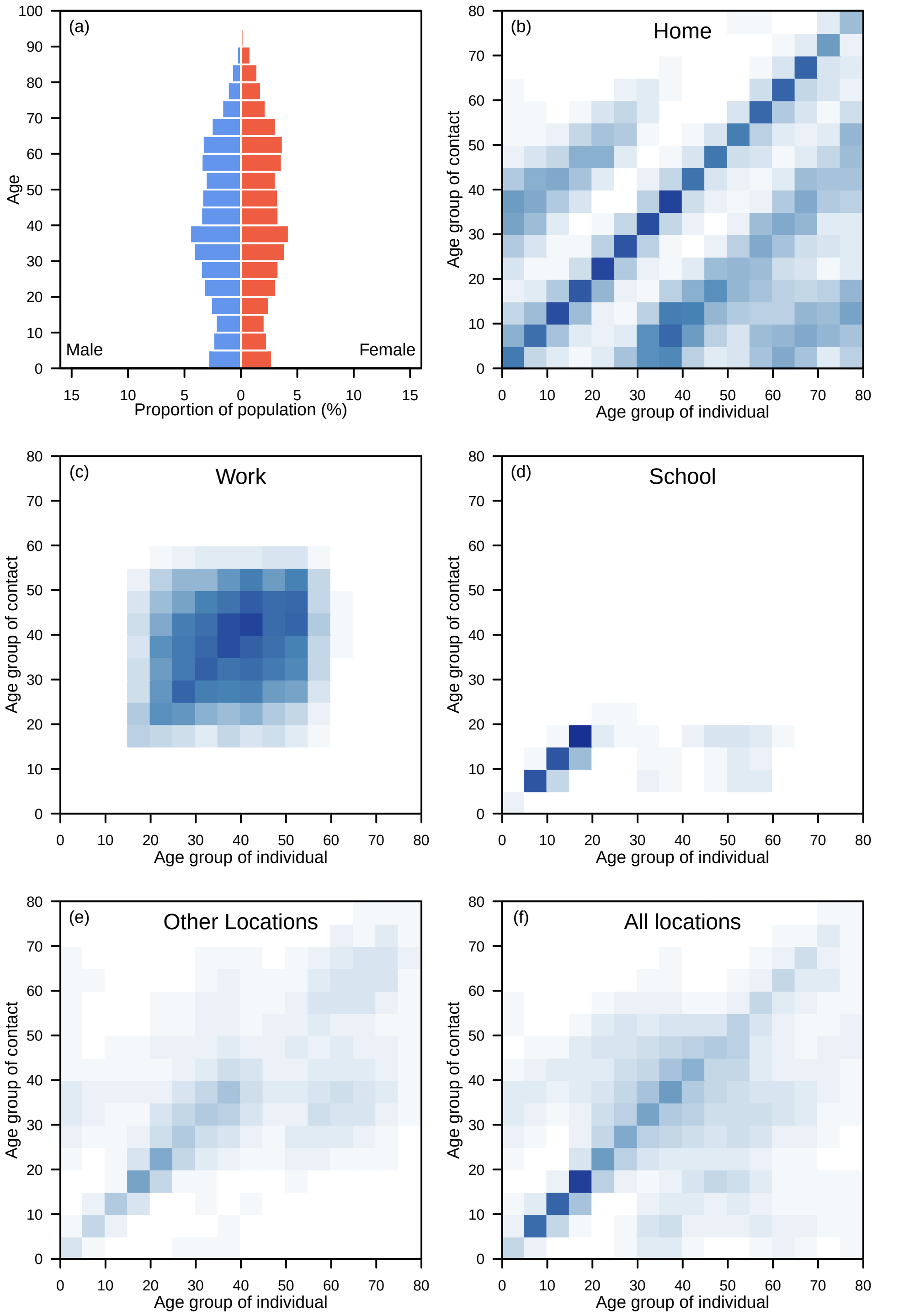

Denmark

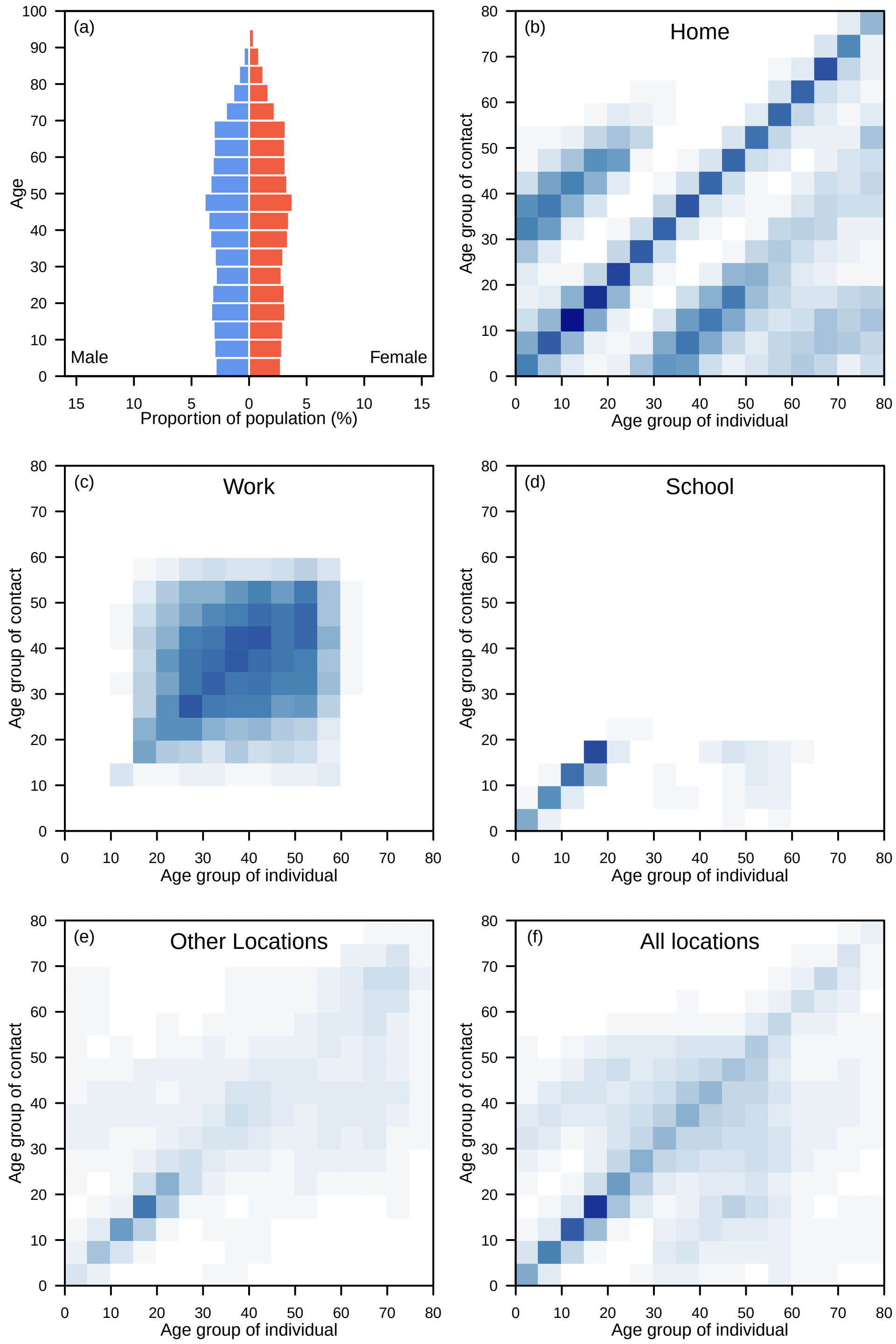

(a)
Age
Male
Female
Proportion of population (%)
(b) Home
(c) Work
(d) School
(e) Other Locations
(f) All locations
Age group of contact
Age group of individual

Dominican Republic

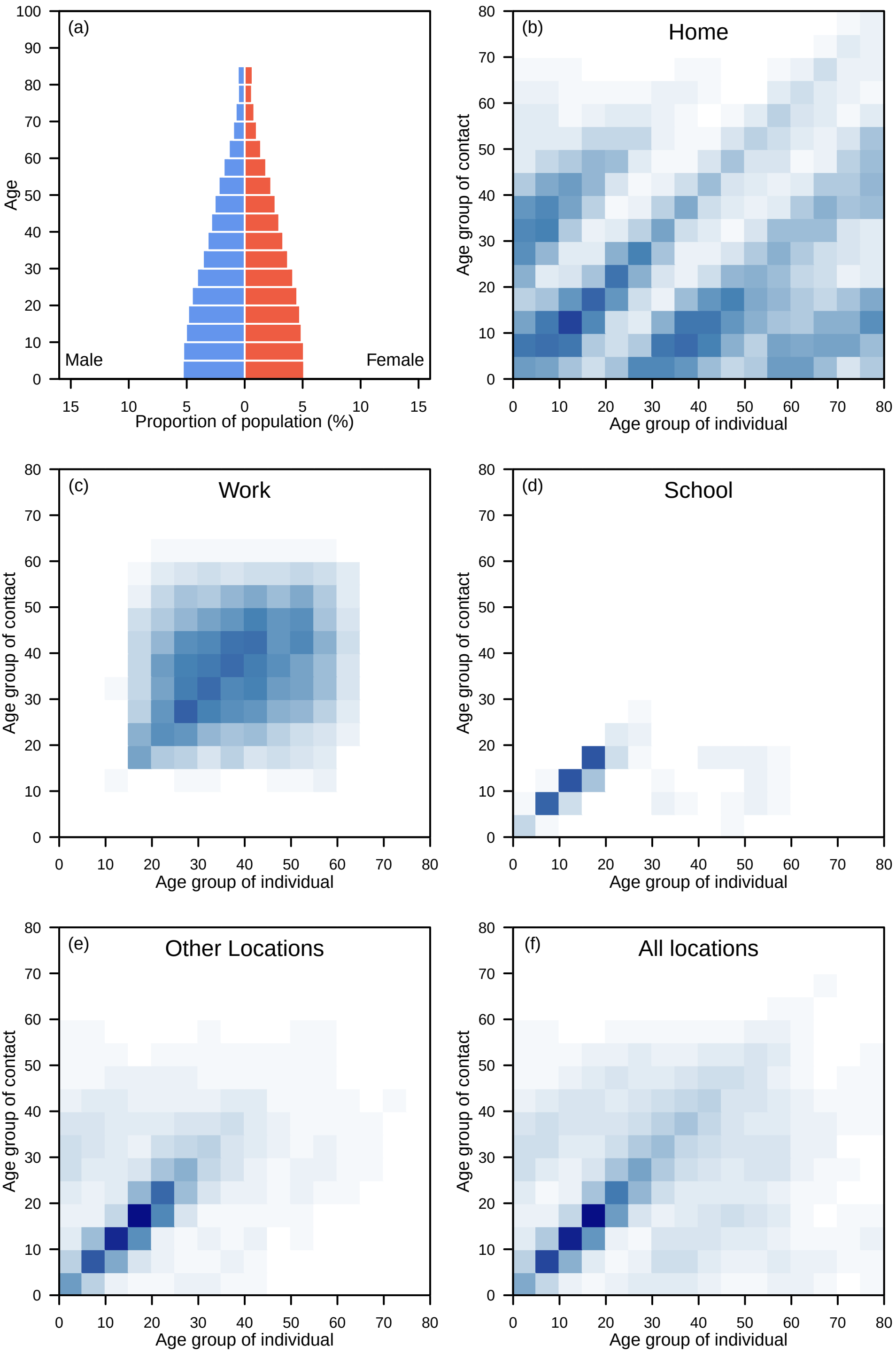

Ecuador

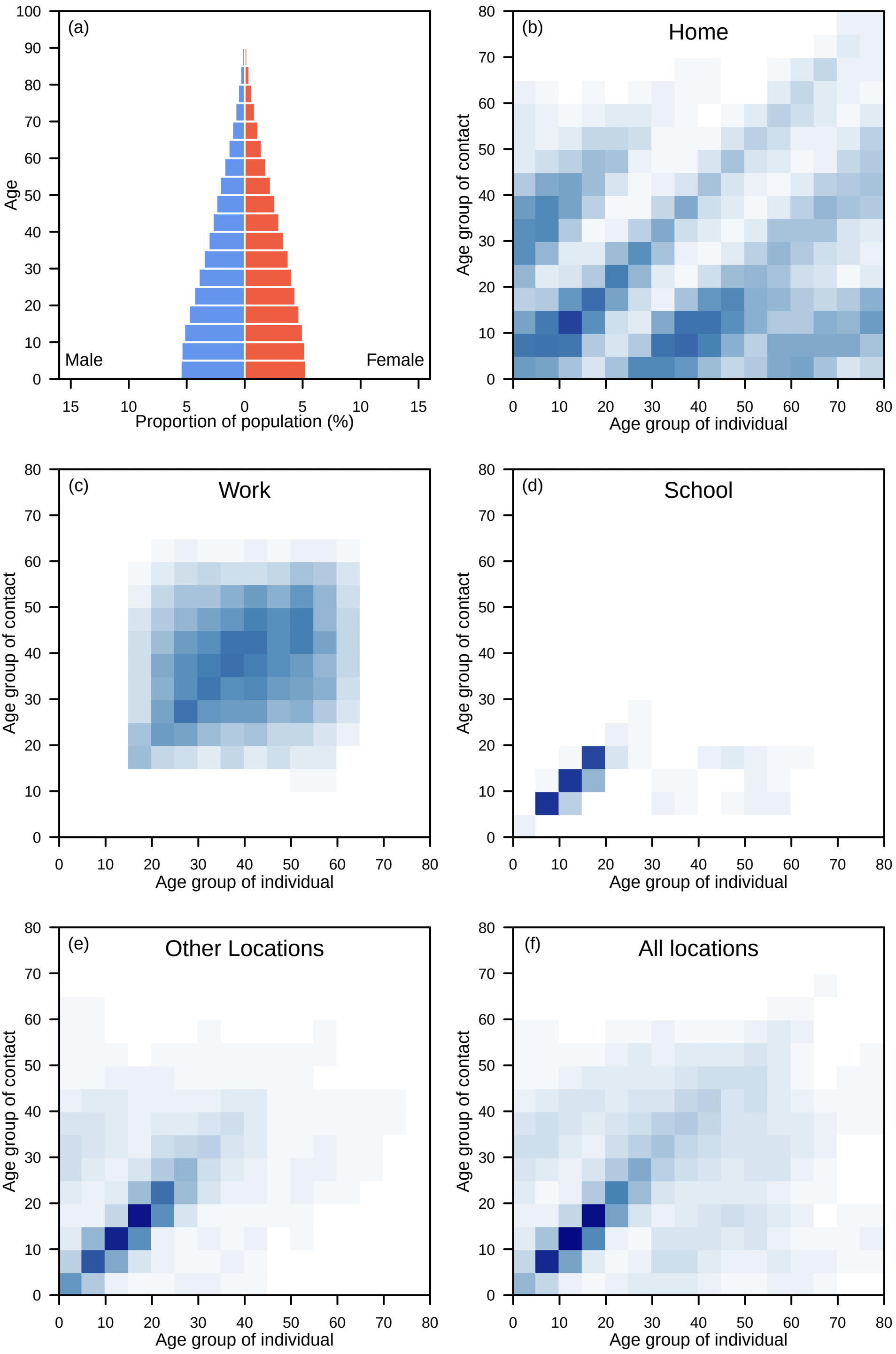
(a)
Male
Female
Age
Proportion of population (%)
(b)
Home
(c)
Work
(d)
School
(e)
Other Locations
(f)
All locations
Age group of contact
Age group of individual

Egypt

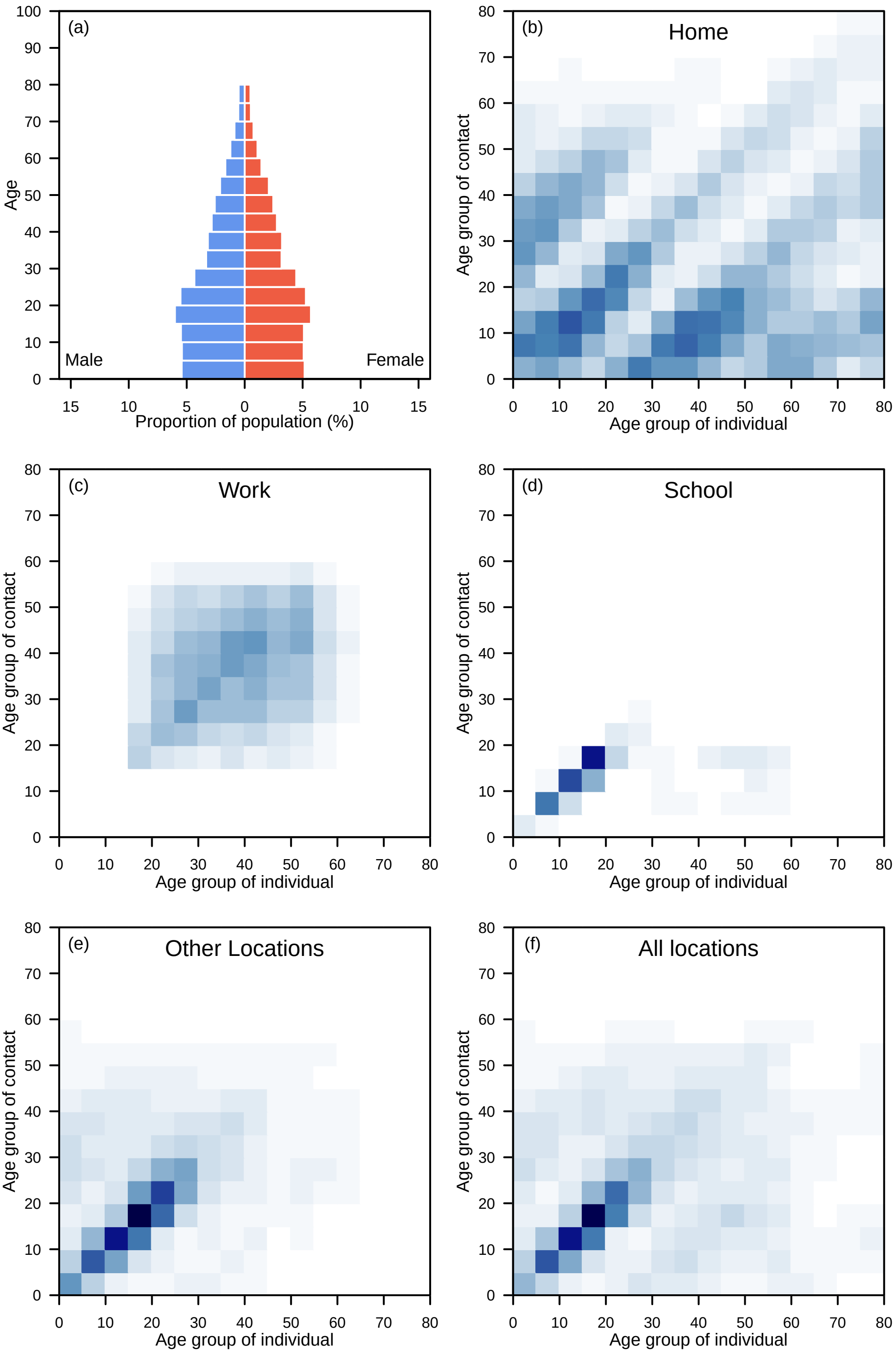
(a)
Age
Male
Female
Proportion of population (%)
(b) Home
(c) Work
(d) School
(e) Other Locations
(f) All locations
Age group of contact
Age group of individual

El Salvador

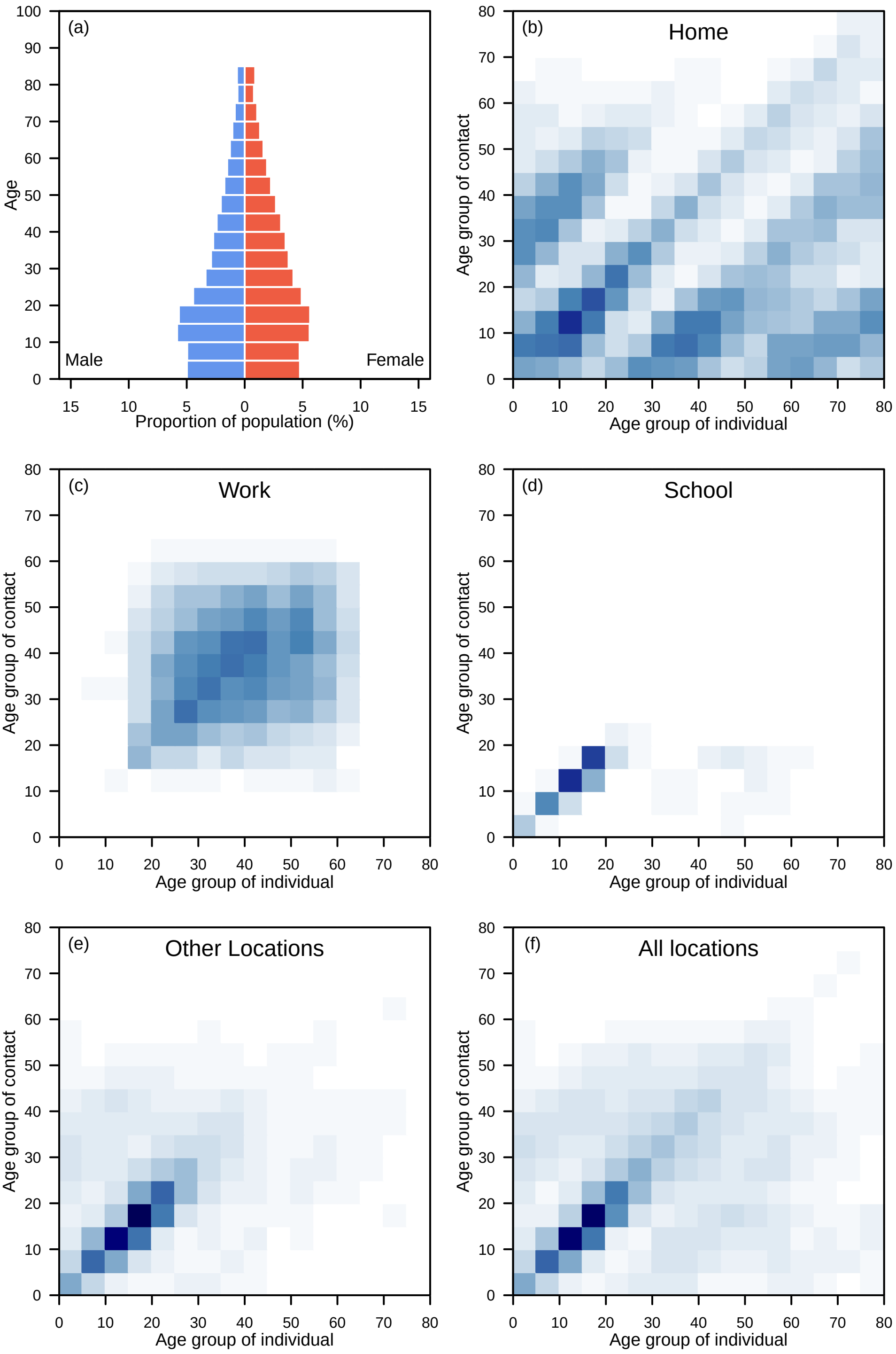

(a)
Age
Male
Female
Proportion of population (%)
(b)
Home
Age group of contact
Age group of individual
(c)
Work
Age group of contact
Age group of individual
(d)
School
Age group of contact
Age group of individual
(e)
Other Locations
Age group of contact
Age group of individual
(f)
All locations
Age group of contact
Age group of individual

Estonia

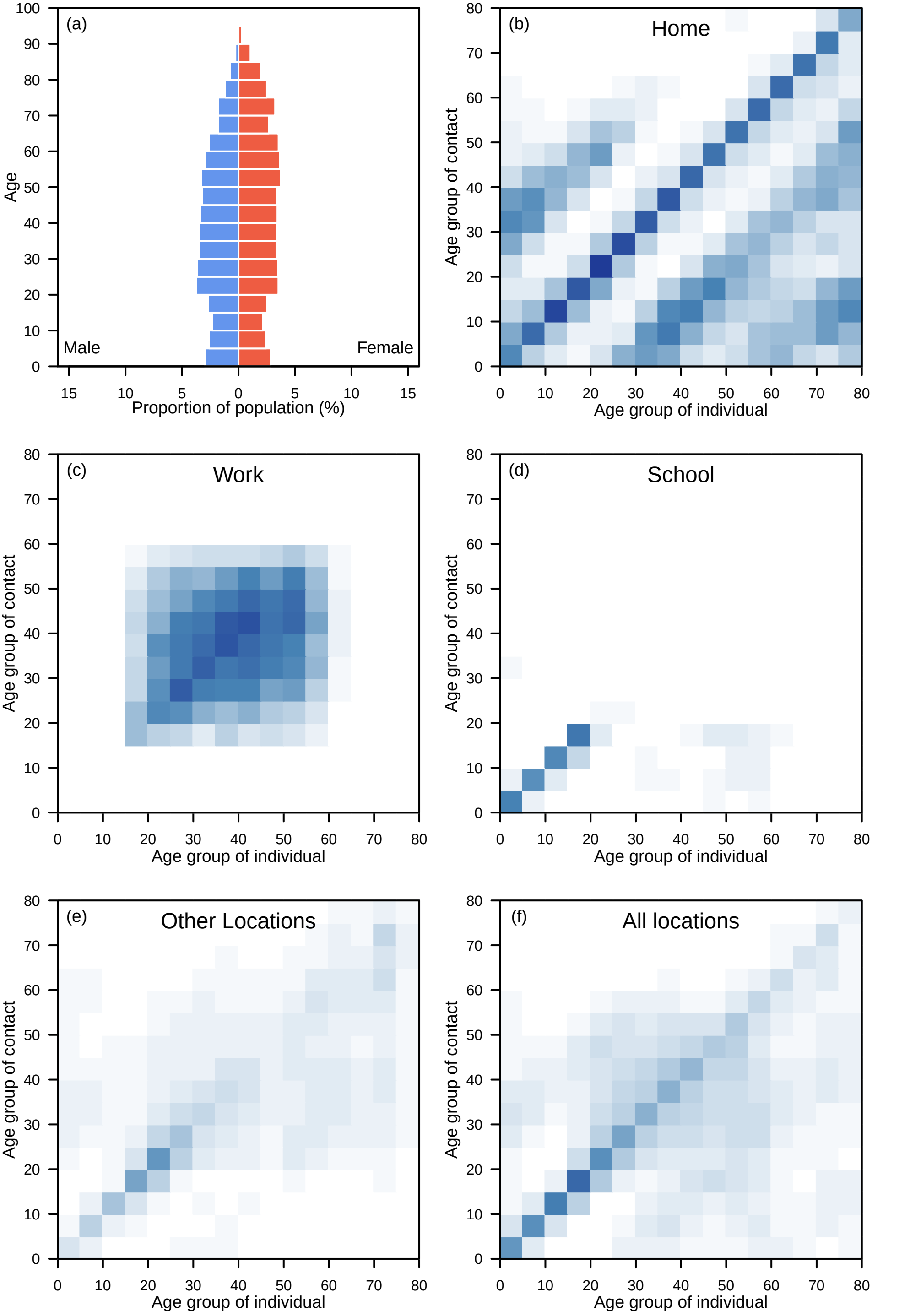

(a)
Age
Male
Female
Proportion of population (%)
(b)
Home
Age group of contact
Age group of individual
(c)
Work
Age group of contact
Age group of individual
(d)
School
Age group of contact
Age group of individual
(e)
Other Locations
Age group of contact
Age group of individual
(f)
All locations
Age group of contact
Age group of individual

Ethiopia

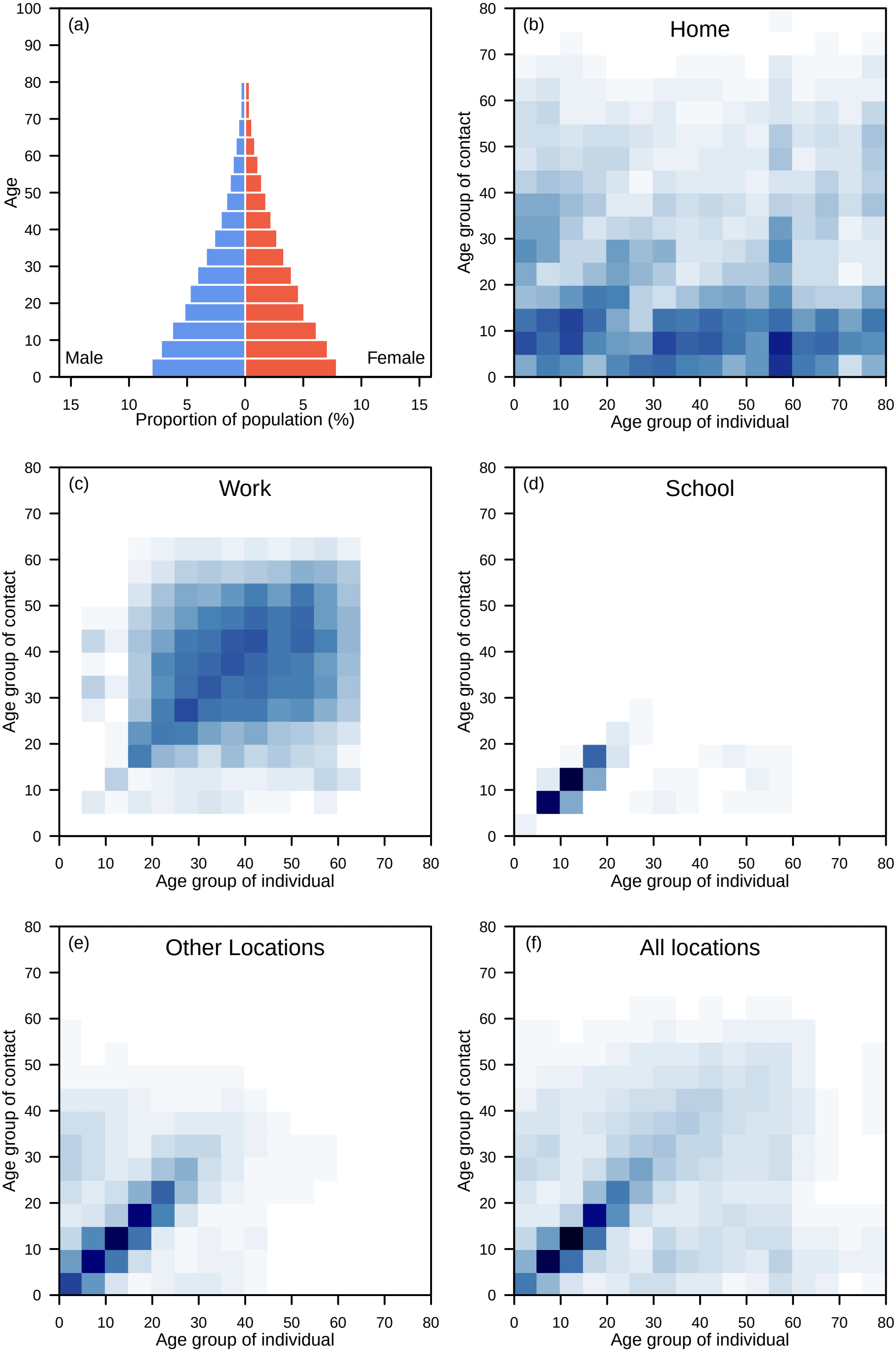

(a)
Age
Male
Female
Proportion of population (%)
(b)
Home
(c)
Work
(d)
School
(e)
Other Locations
(f)
All locations
Age group of contact
Age group of individual

Fiji

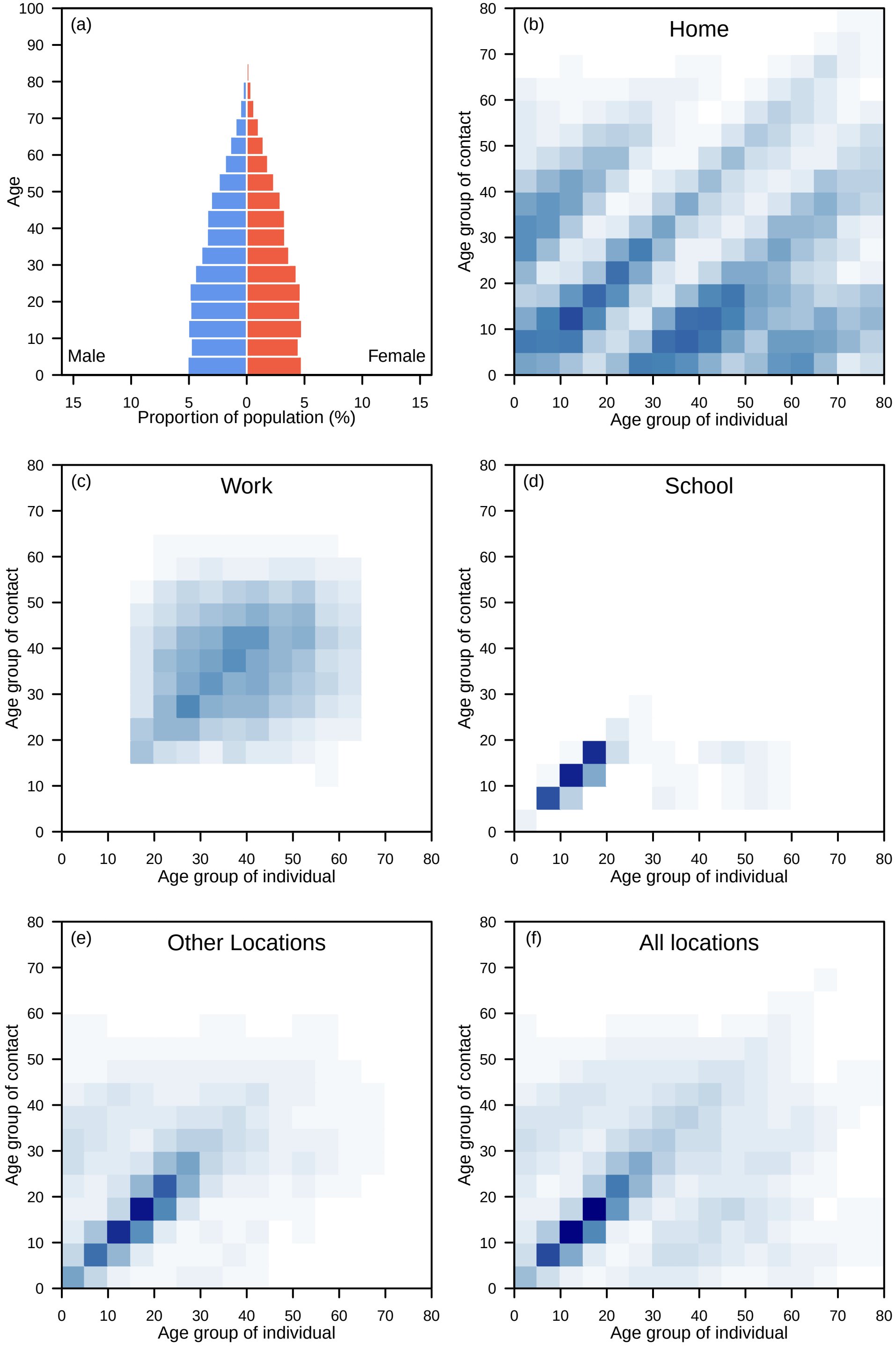

(a)
Age
Male
Female
Proportion of population (%)
(b)
Home
Age group of contact
Age group of individual
(c)
Work
Age group of contact
Age group of individual
(d)
School
Age group of contact
Age group of individual
(e)
Other Locations
Age group of contact
Age group of individual
(f)
All locations
Age group of contact
Age group of individual

Finland

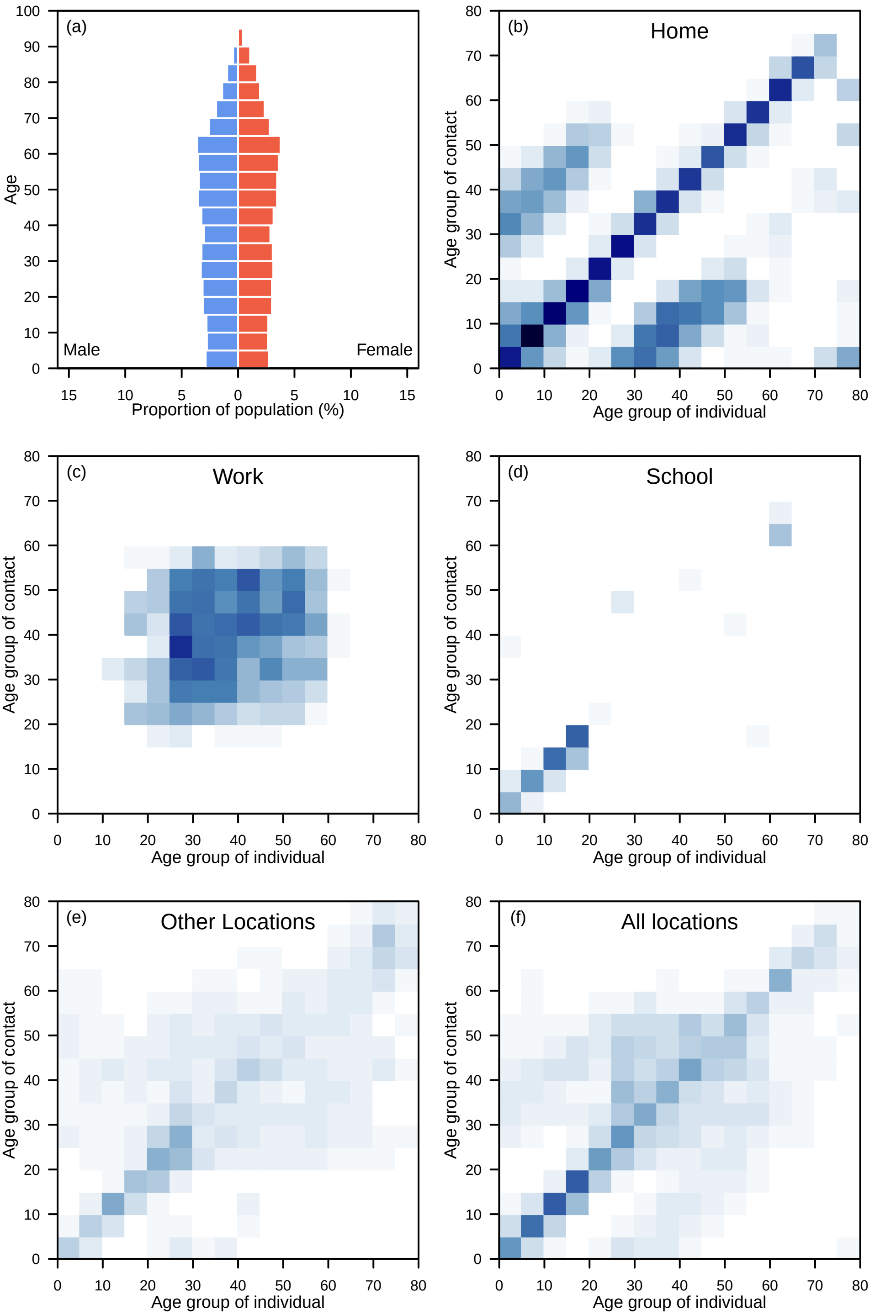

(a)
Age
Male
Female
Proportion of population (%)
(b)
Home
Age group of contact
Age group of individual
(c)
Work
Age group of contact
Age group of individual
(d)
School
Age group of contact
Age group of individual
(e)
Other Locations
Age group of contact
Age group of individual
(f)
All locations
Age group of contact
Age group of individual

France

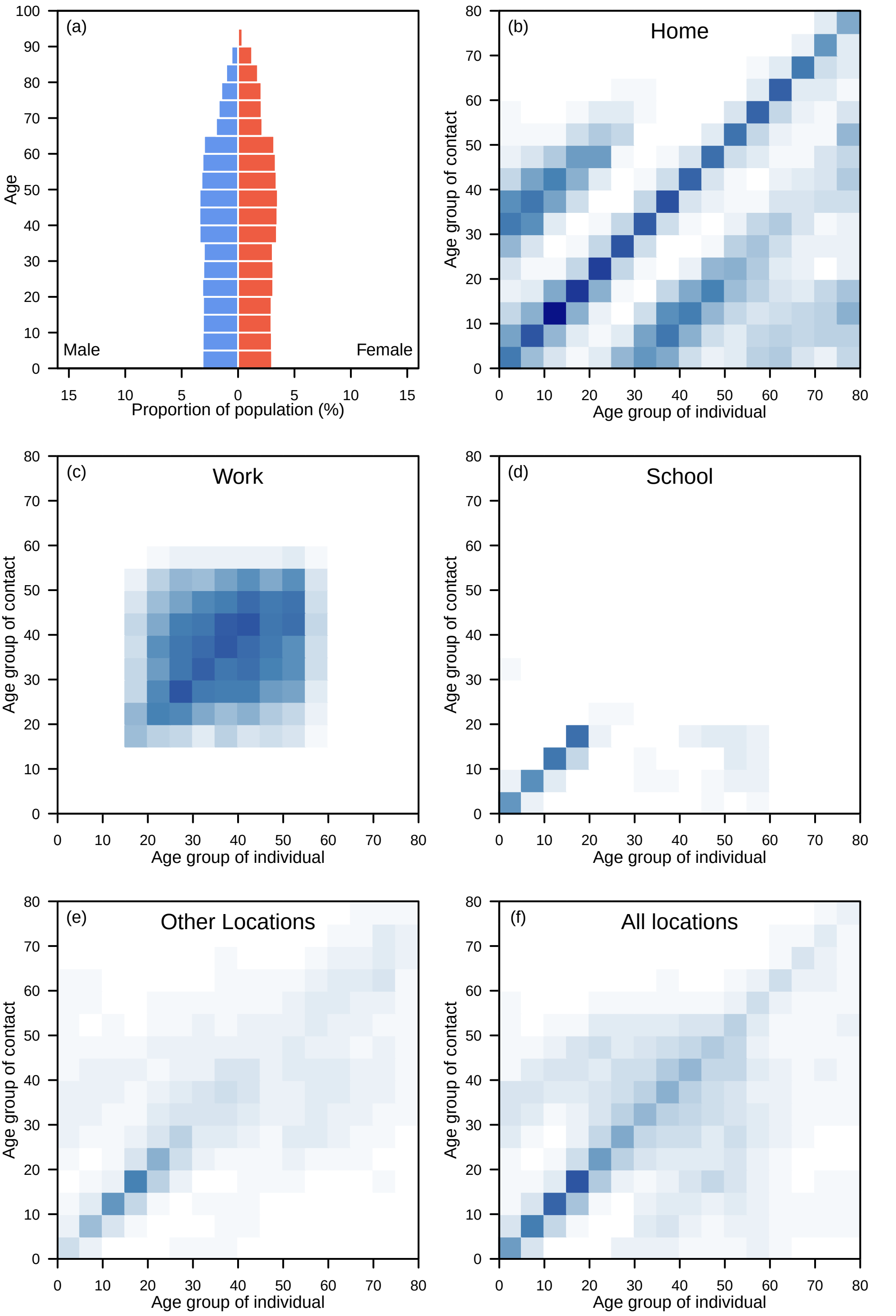

(a)
Age
Male
Female
Proportion of population (%)
(b)
Home
Age group of contact
Age group of individual
(c)
Work
Age group of contact
Age group of individual
(d)
School
Age group of contact
Age group of individual
(e)
Other Locations
Age group of contact
Age group of individual
(f)
All locations
Age group of contact
Age group of individual

Georgia

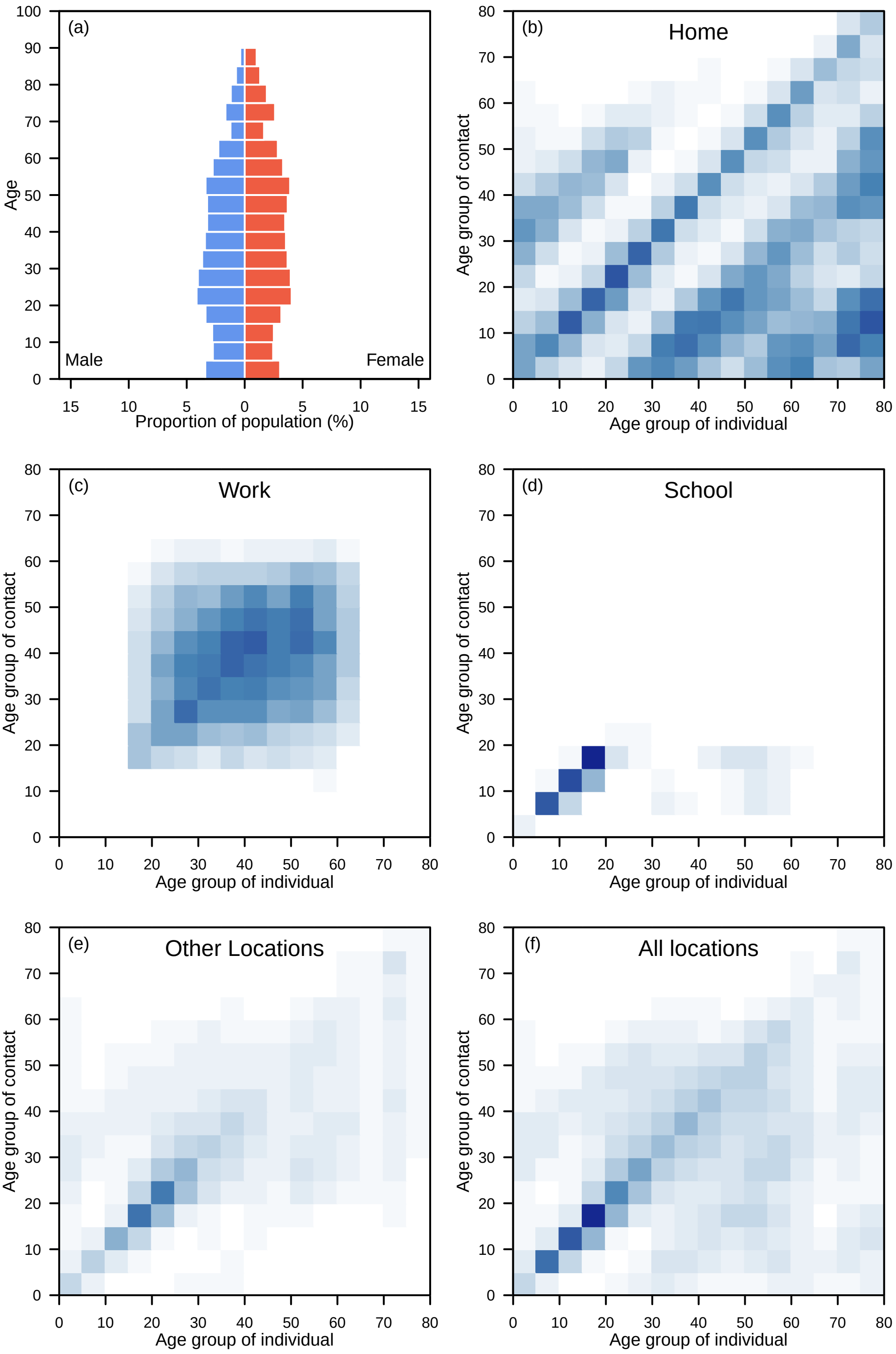

(a)
Age
Male
Female
Proportion of population (%)
(b)
Home
Age group of contact
Age group of individual
(c)
Work
Age group of contact
Age group of individual
(d)
School
Age group of contact
Age group of individual
(e)
Other Locations
Age group of contact
Age group of individual
(f)
All locations
Age group of contact
Age group of individual

Germany

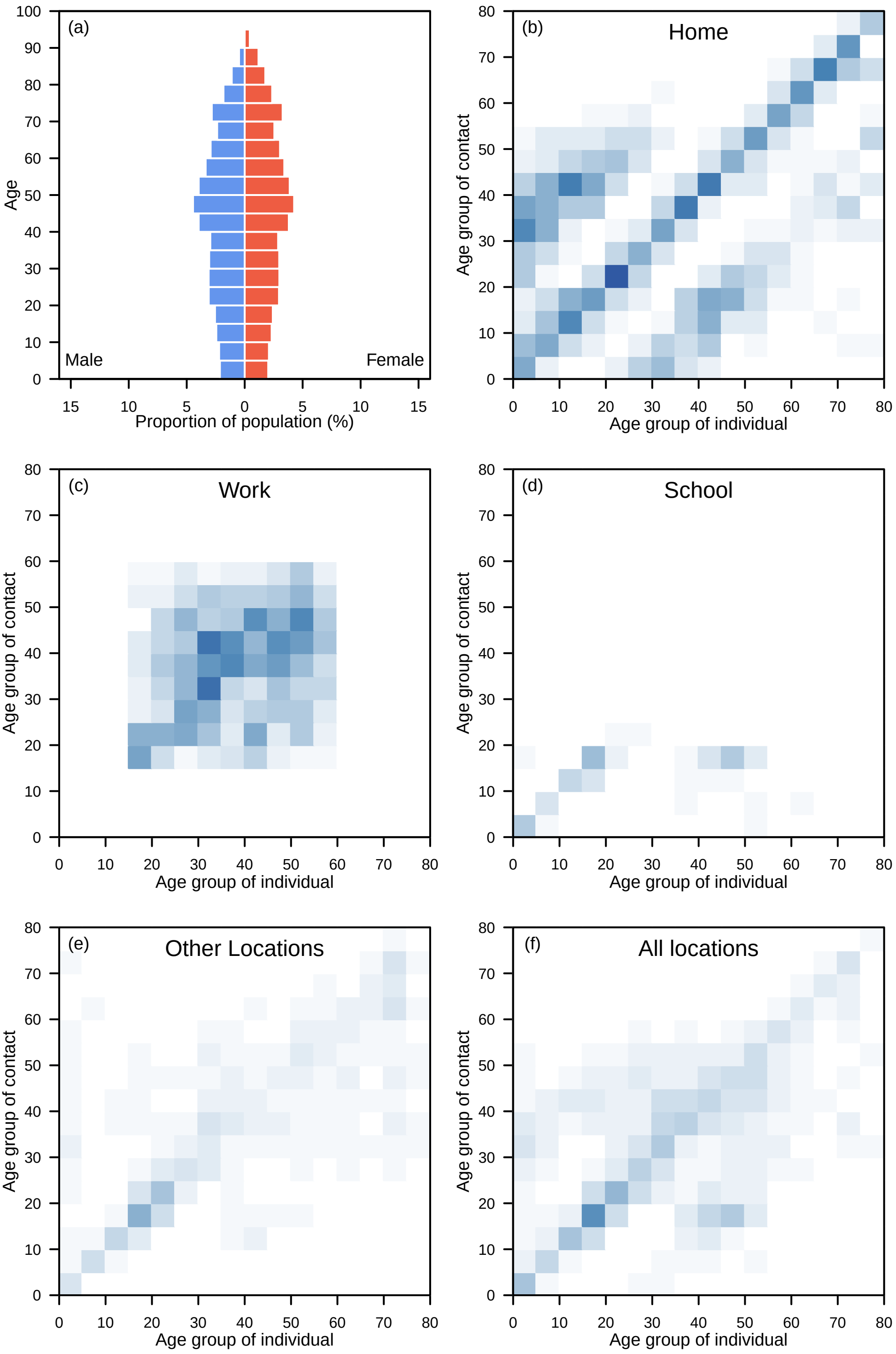

(a)
Age
Male
Female
Proportion of population (%)
(b) Home
Age group of contact
Age group of individual
(c) Work
(d) School
(e) Other Locations
(f) All locations

Ghana

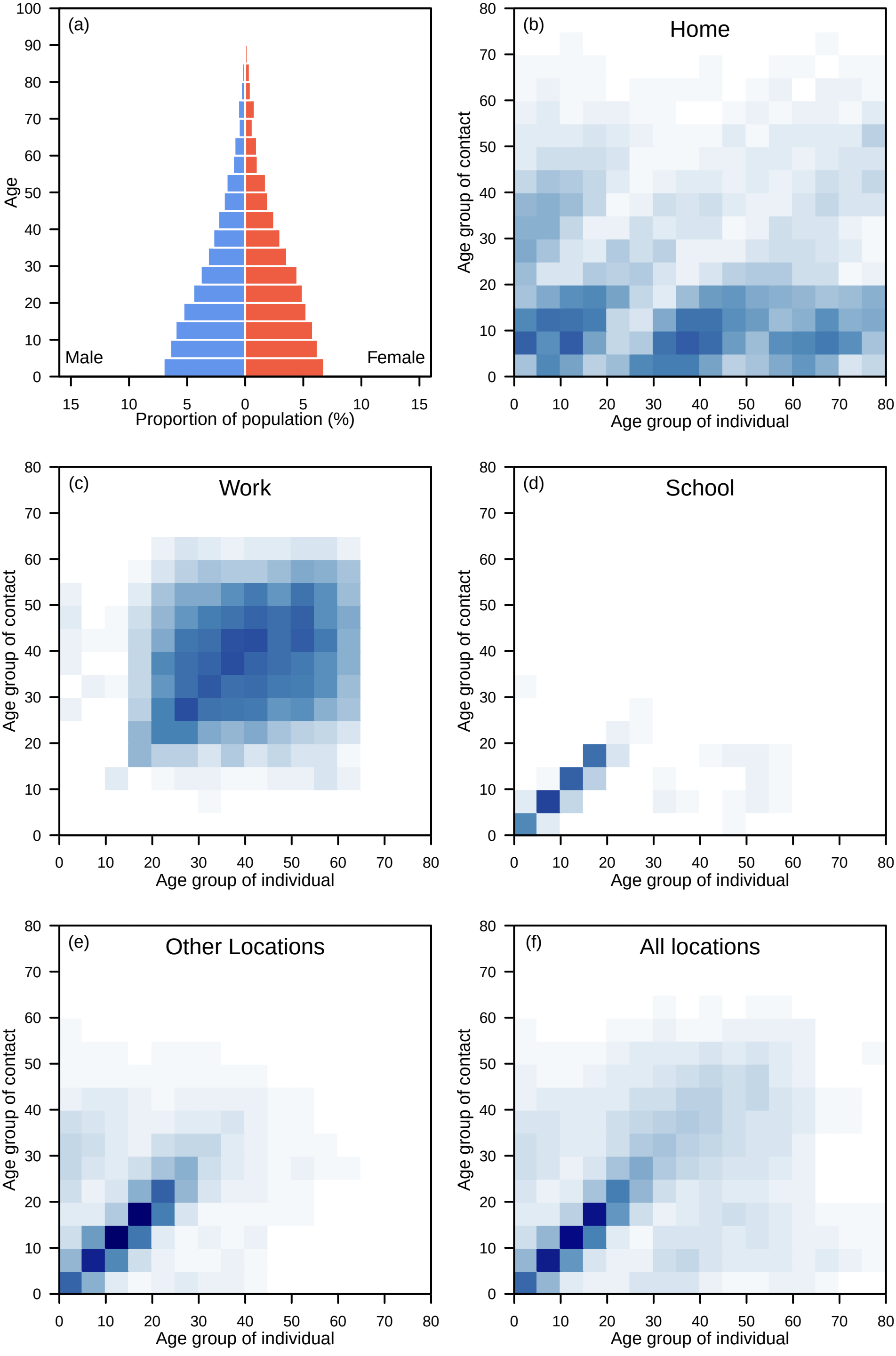

(a)
Male
Female
Age
Proportion of population (%)
(b)
Home
Age group of contact
Age group of individual
(c)
Work
Age group of contact
Age group of individual
(d)
School
Age group of contact
Age group of individual
(e)
Other Locations
Age group of contact
Age group of individual
(f)
All locations
Age group of contact
Age group of individual

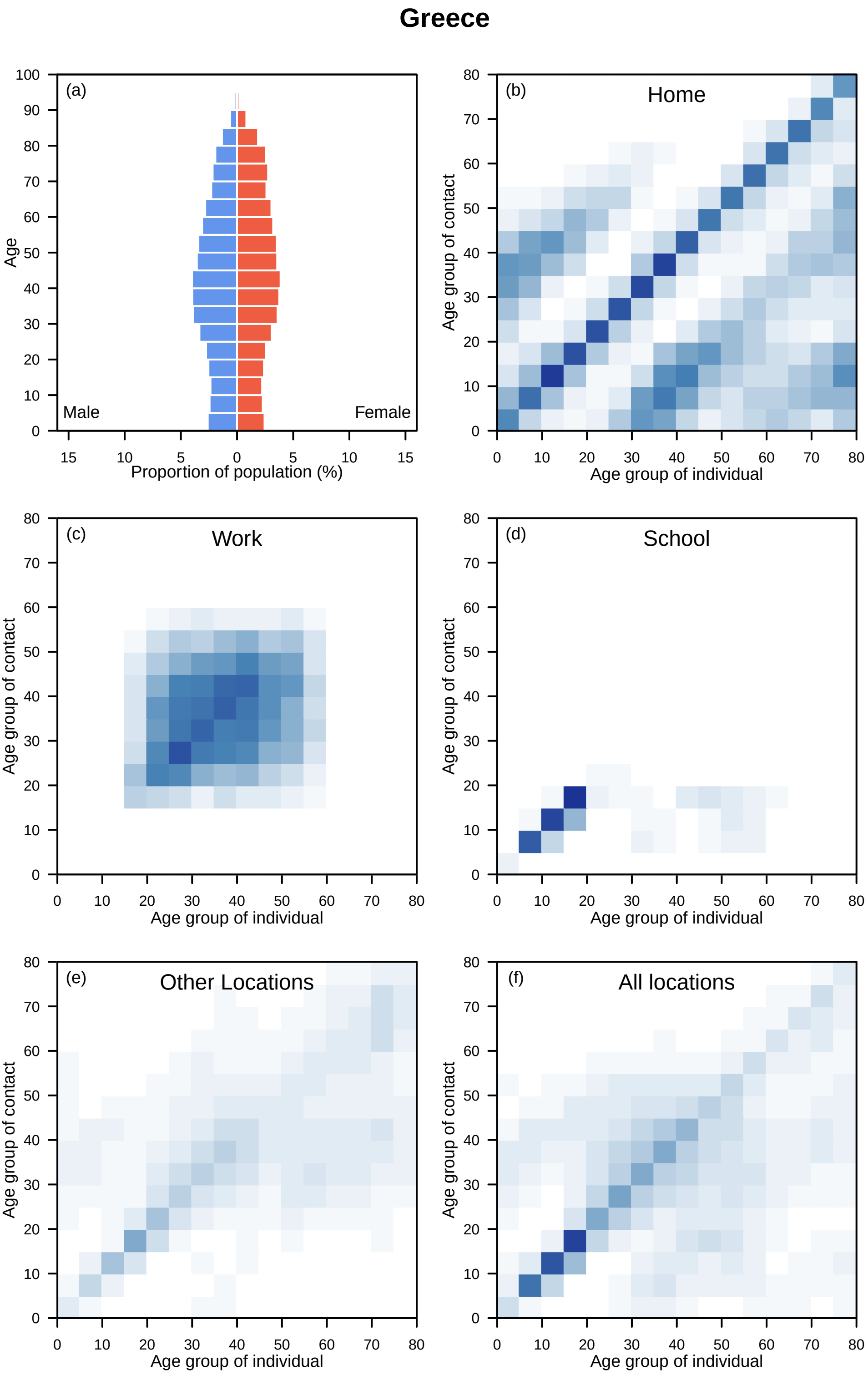
Greece
(a)
Male
Female
Age
Proportion of population (%)
(b)
Home
(c)
Work
(d)
School
(e)
Other Locations
(f)
All locations
Age group of contact
Age group of individual

Guatemala

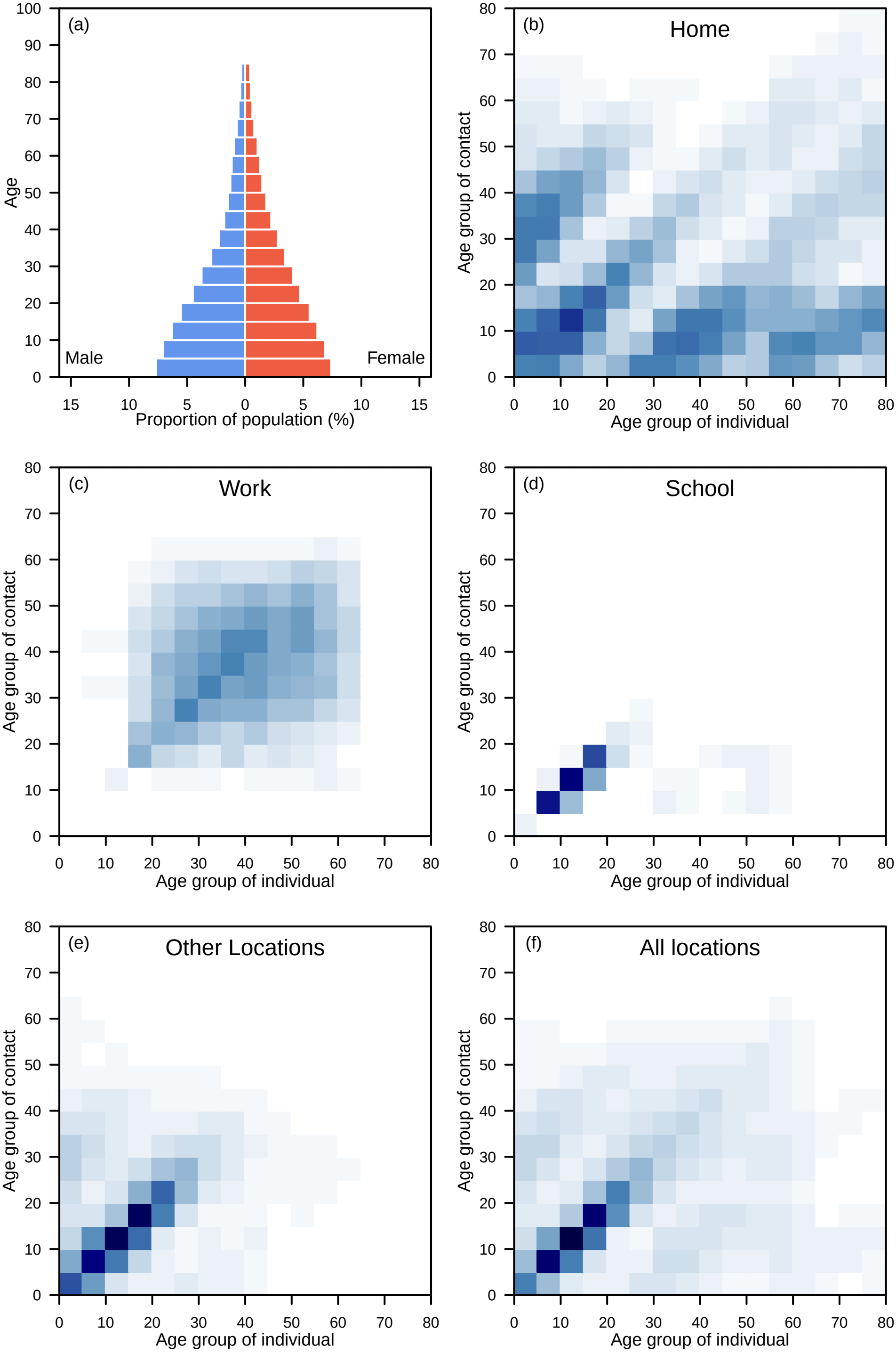

(a)
Age
Male
Female
Proportion of population (%)
(b)
Home
Age group of contact
Age group of individual
(c)
Work
Age group of contact
Age group of individual
(d)
School
Age group of contact
Age group of individual
(e)
Other Locations
Age group of contact
Age group of individual
(f)
All locations
Age group of contact
Age group of individual

Guinea

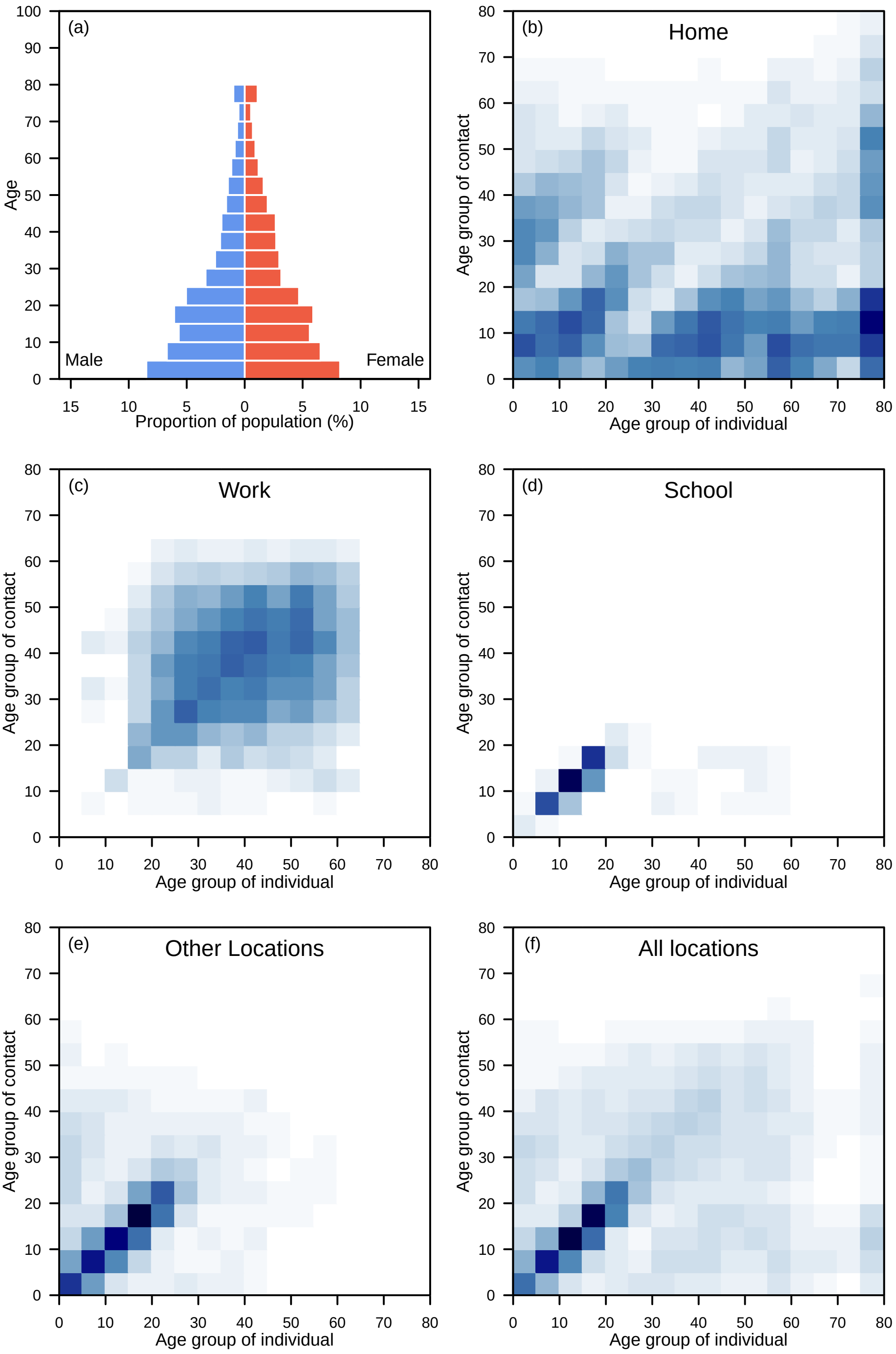

(a)
Age
Male
Female
Proportion of population (%)
(b)
Home
(c)
Work
(d)
School
(e)
Other Locations
(f)
All locations
Age group of contact
Age group of individual

Guyana

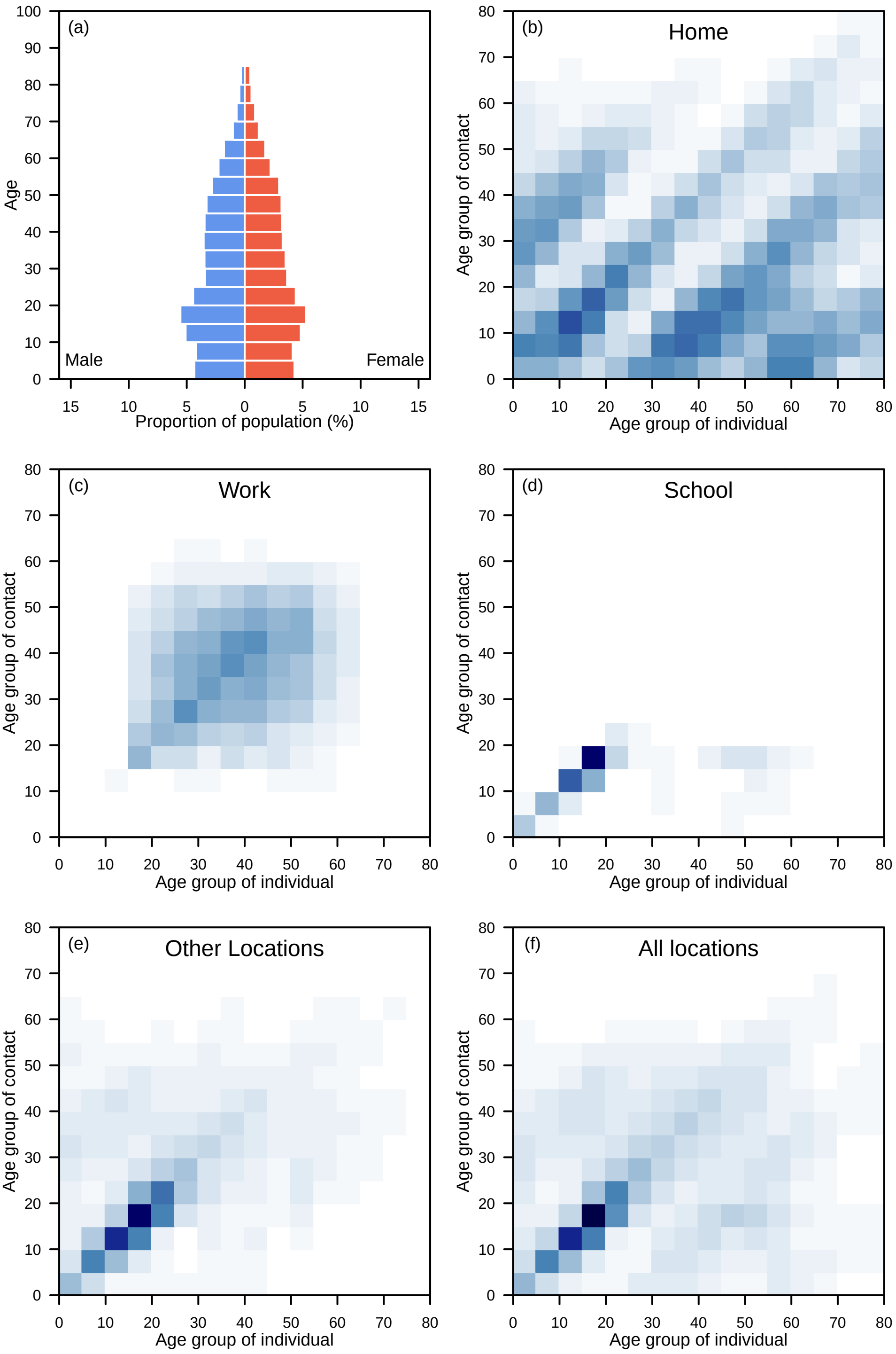
(a)
Age
Male
Female
Proportion of population (%)
(b)
Home
Age group of contact
Age group of individual
(c)
Work
Age group of contact
Age group of individual
(d)
School
Age group of contact
Age group of individual
(e)
Other Locations
Age group of contact
Age group of individual
(f)
All locations
Age group of contact
Age group of individual

Haiti

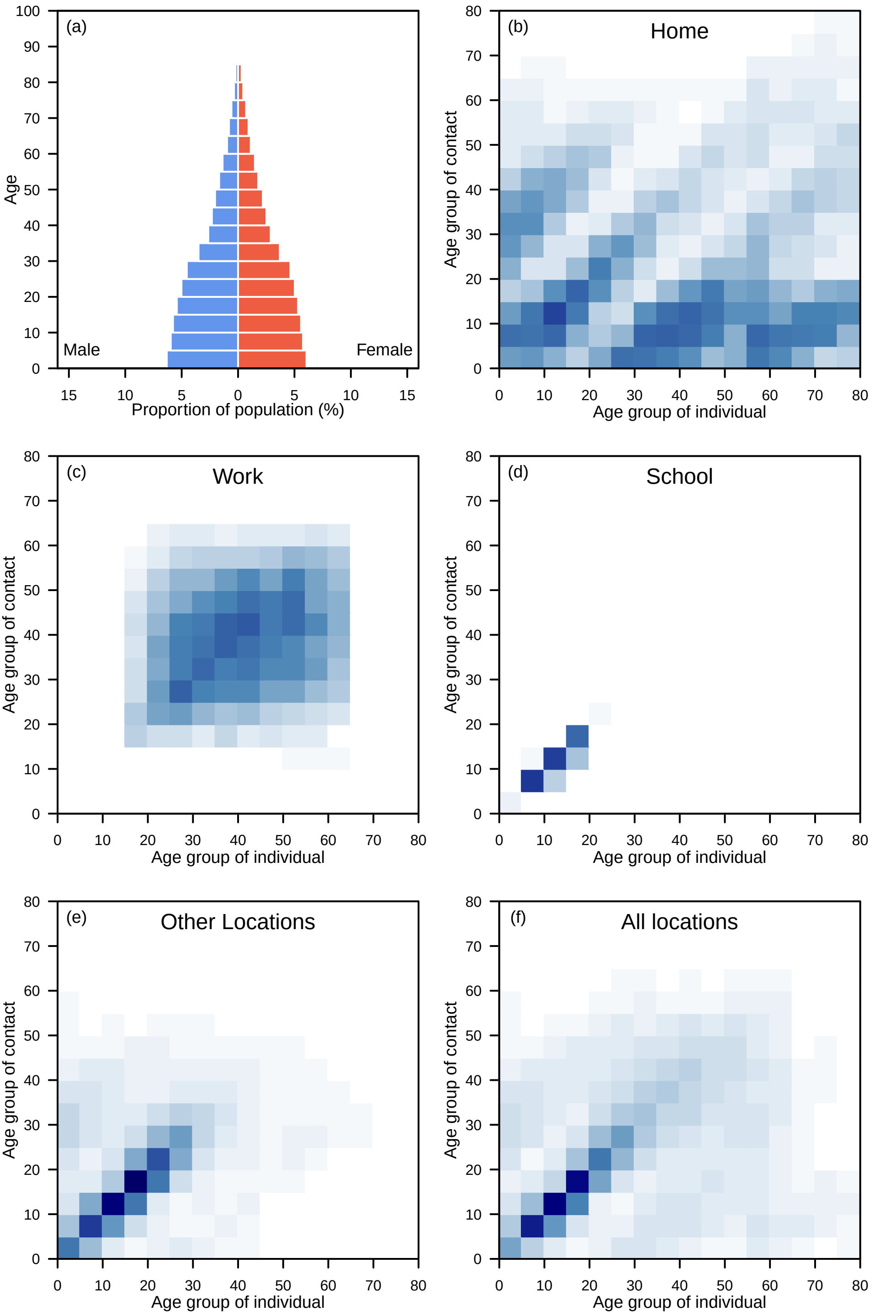

(a)
Age
Male
Female
Proportion of population (%)
(b)
Home
Age group of contact
Age group of individual
(c)
Work
Age group of contact
Age group of individual
(d)
School
Age group of contact
Age group of individual
(e)
Other Locations
Age group of contact
Age group of individual
(f)
All locations
Age group of contact
Age group of individual

Honduras

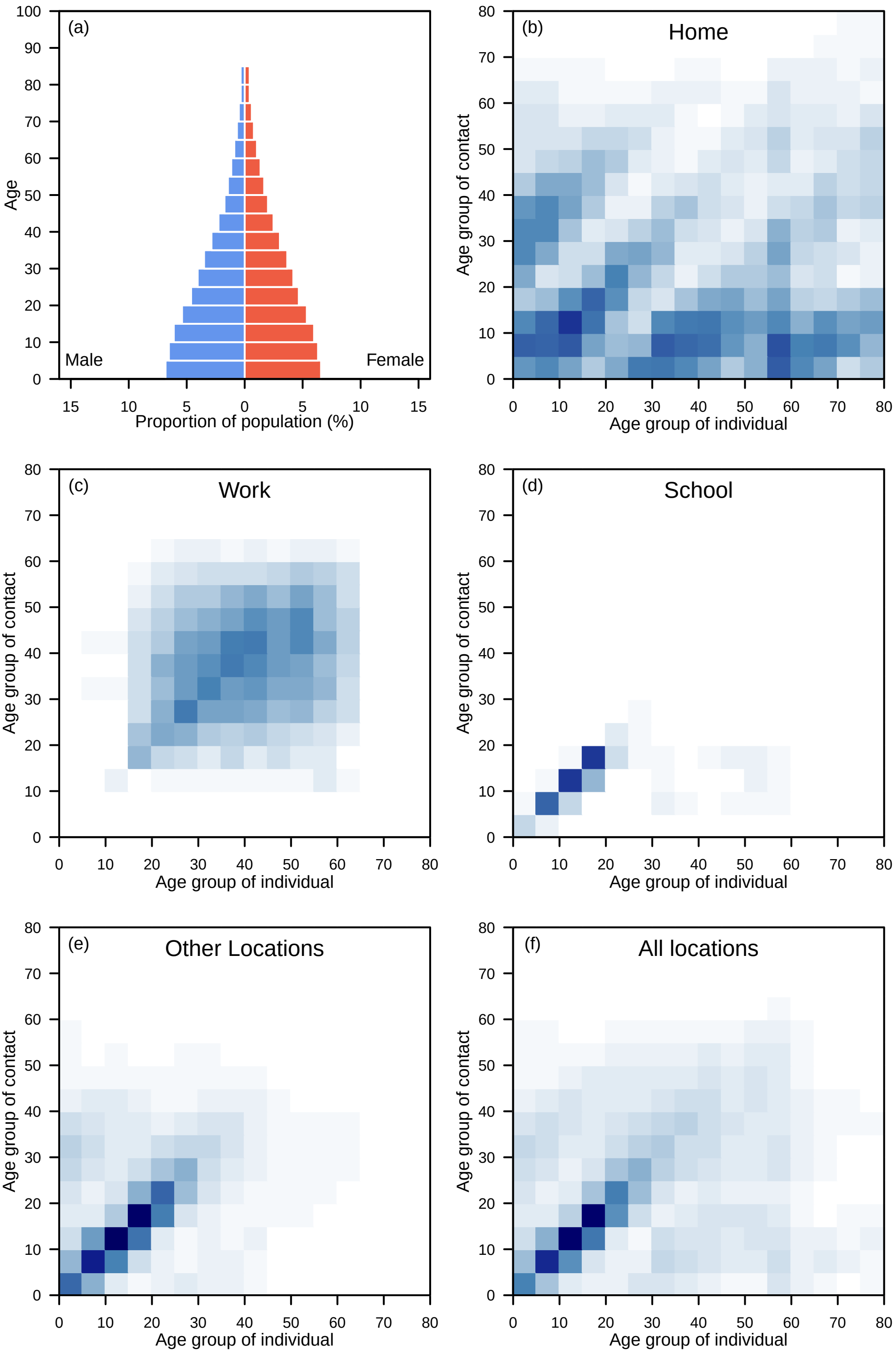

(a)
Male
Female
Age
Proportion of population (%)
(b)
Home
(c)
Work
(d)
School
(e)
Other Locations
(f)
All locations
Age group of contact
Age group of individual

Hong Kong SAR, China

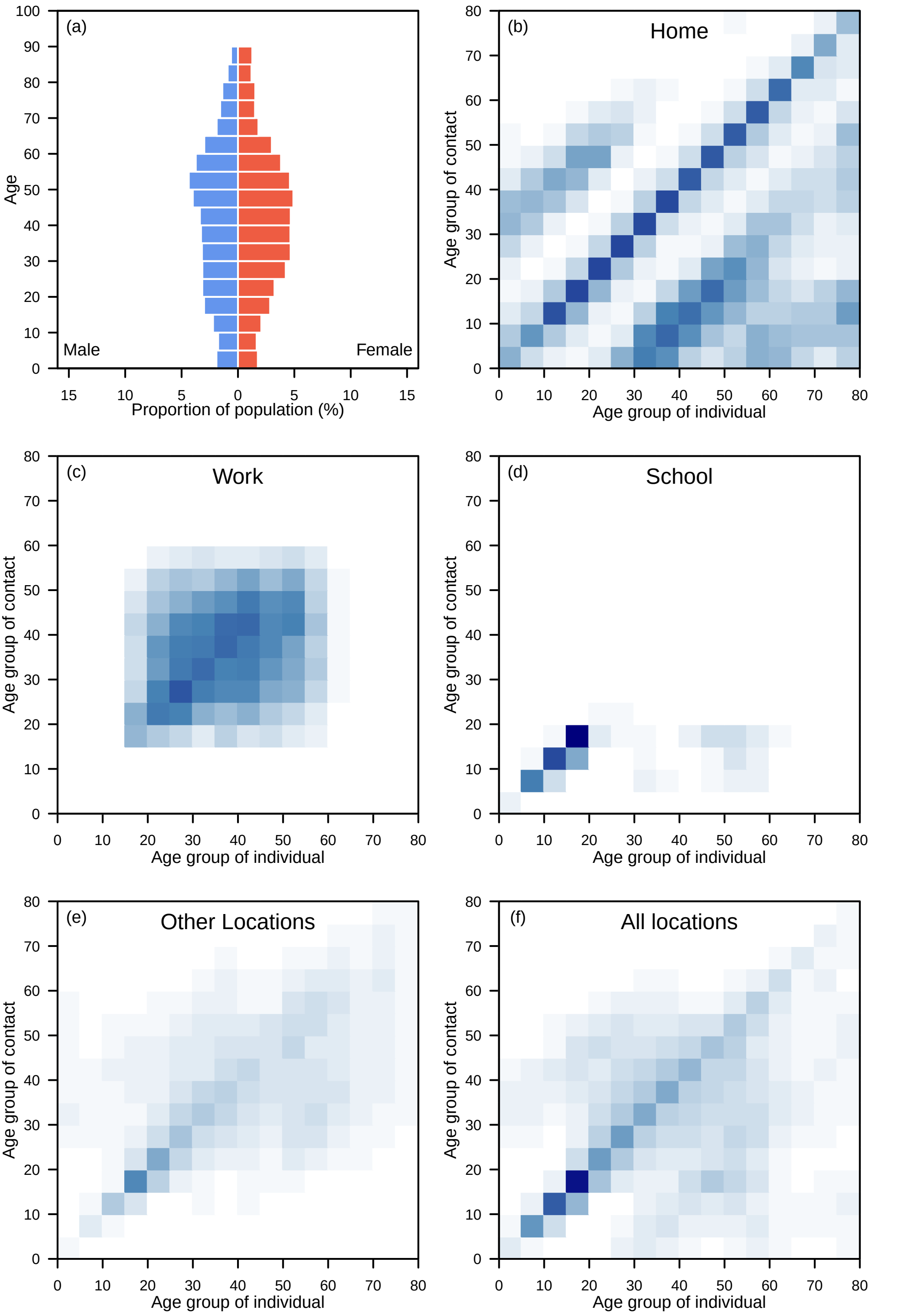

Hungary

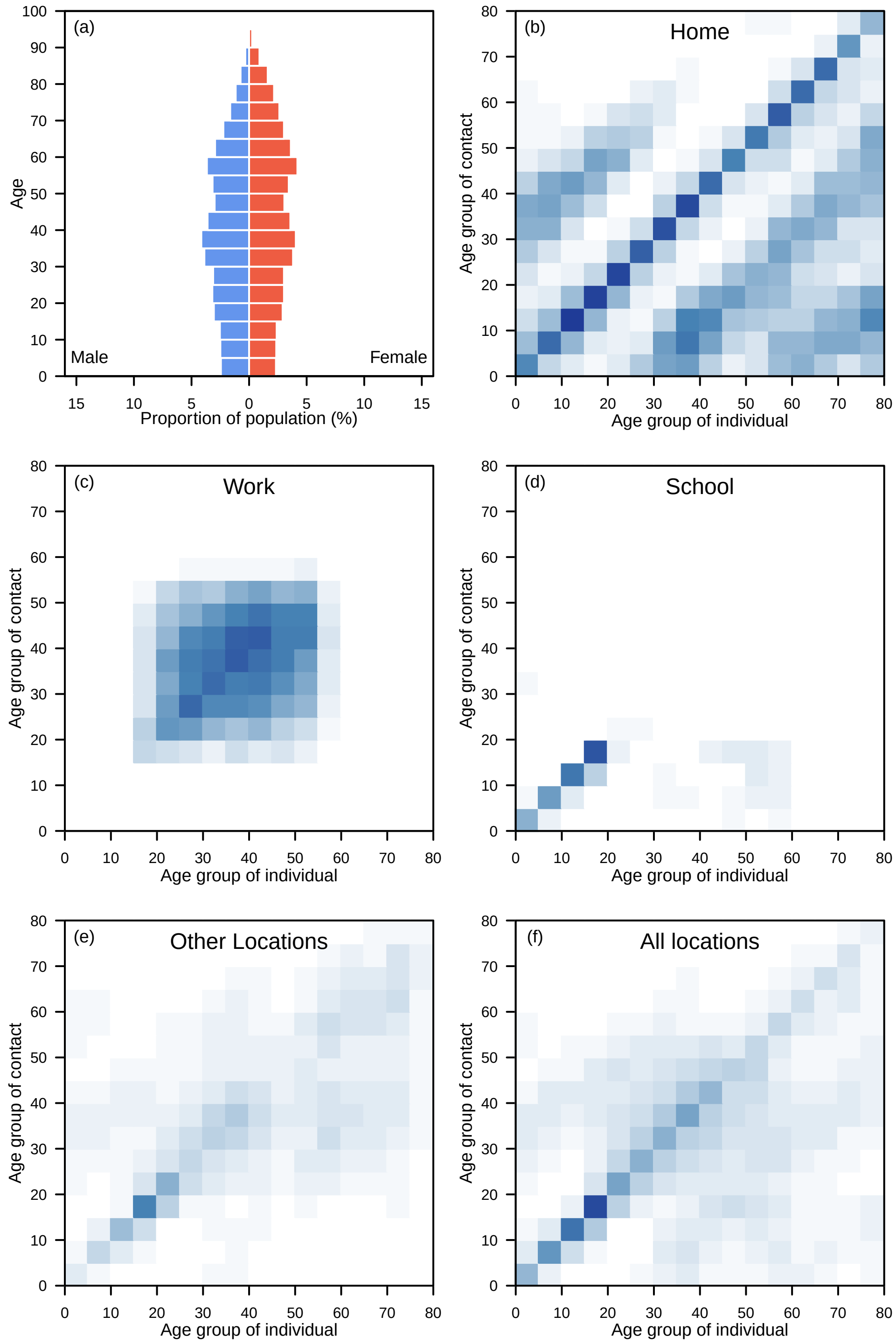

(a)
Age
Male
Female
Proportion of population (%)
(b)
Home
Age group of contact
Age group of individual
(c)
Work
Age group of contact
Age group of individual
(d)
School
Age group of contact
Age group of individual
(e)
Other Locations
Age group of contact
Age group of individual
(f)
All locations
Age group of contact
Age group of individual

Iceland

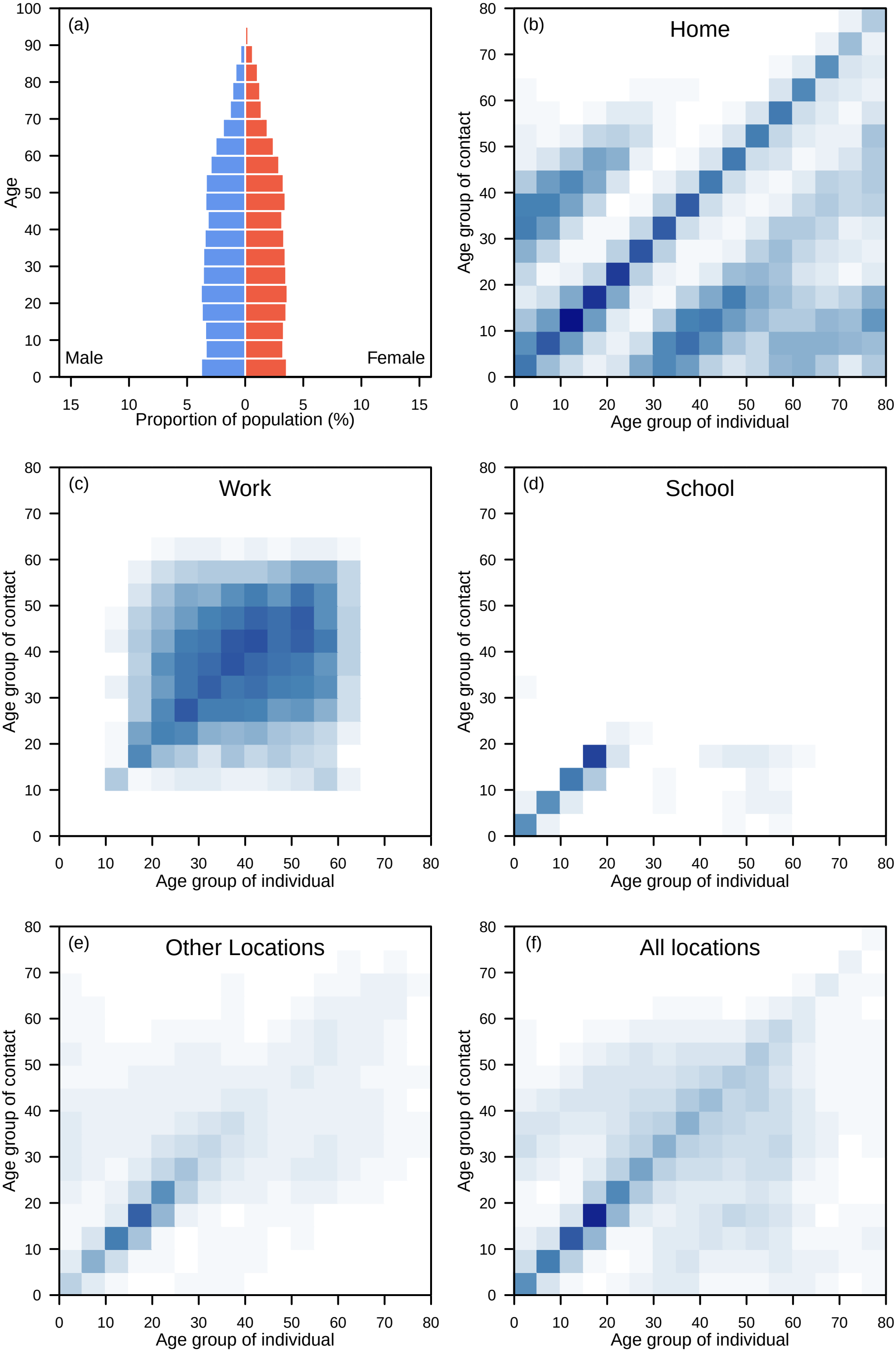

(a)
Age
Male
Female
Proportion of population (%)
(b)
Home
Age group of contact
Age group of individual
(c)
Work
Age group of contact
Age group of individual
(d)
School
Age group of contact
Age group of individual
(e)
Other Locations
Age group of contact
Age group of individual
(f)
All locations
Age group of contact
Age group of individual

India

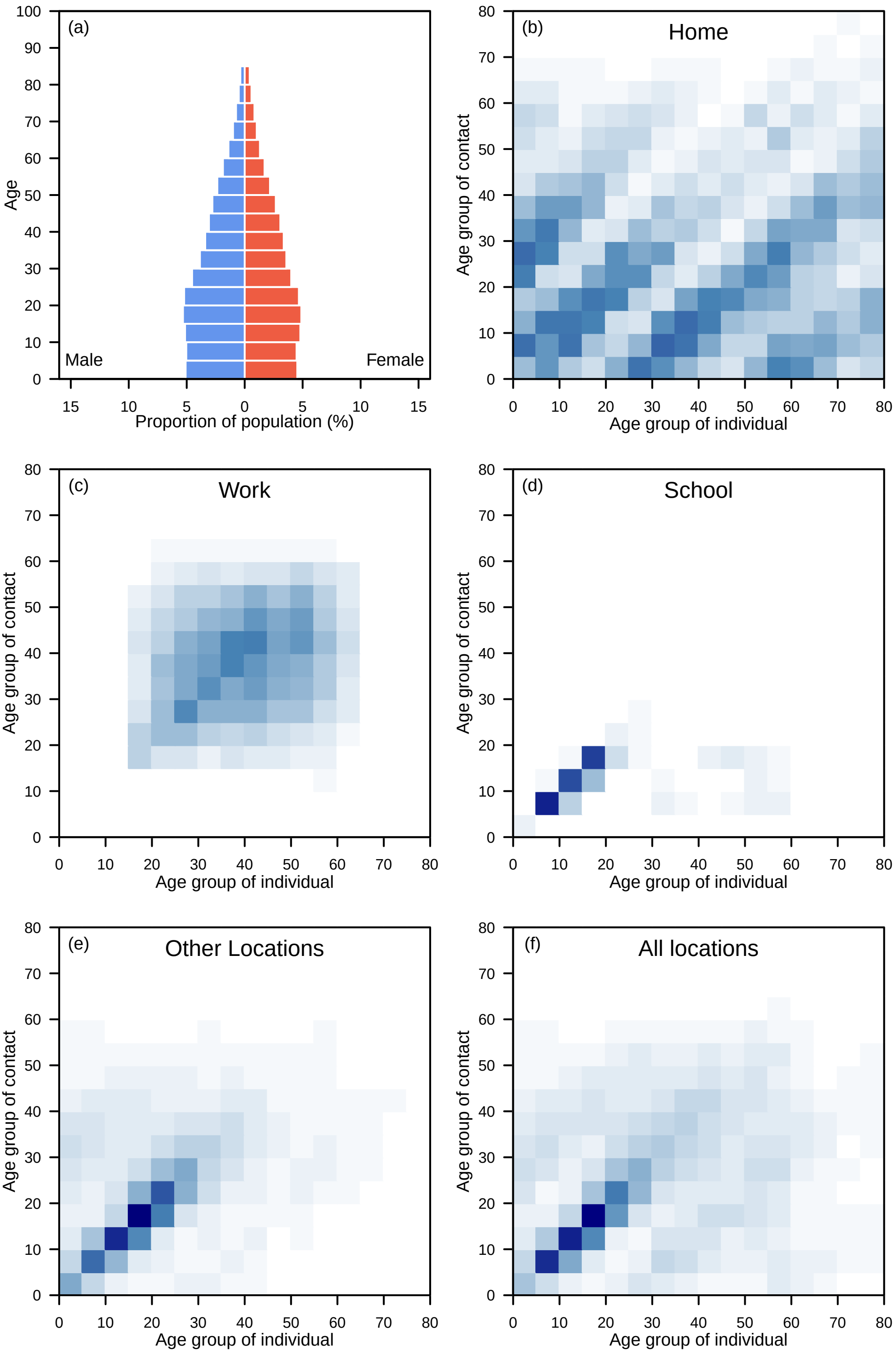

(a)
Age
Male
Female
Proportion of population (%)
(b)
Home
Age group of contact
Age group of individual
(c)
Work
Age group of contact
Age group of individual
(d)
School
Age group of contact
Age group of individual
(e)
Other Locations
Age group of contact
Age group of individual
(f)
All locations
Age group of contact
Age group of individual

Indonesia

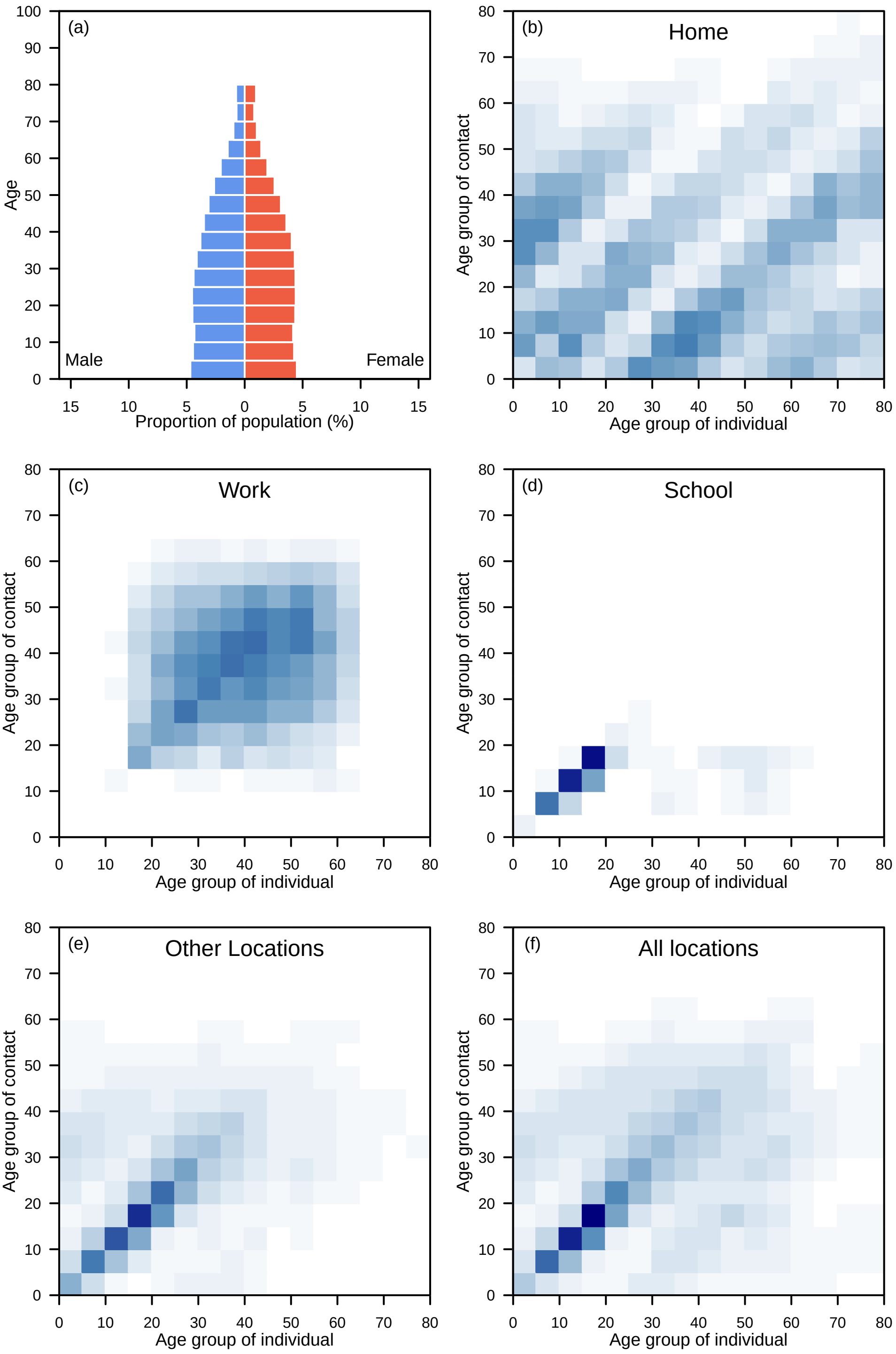

(a)
Age
Male
Female
Proportion of population (%)
(b)
Home
Age group of contact
Age group of individual
(c)
Work
Age group of contact
Age group of individual
(d)
School
Age group of contact
Age group of individual
(e)
Other Locations
Age group of contact
Age group of individual
(f)
All locations
Age group of contact
Age group of individual

Iran (Islamic Republic of)

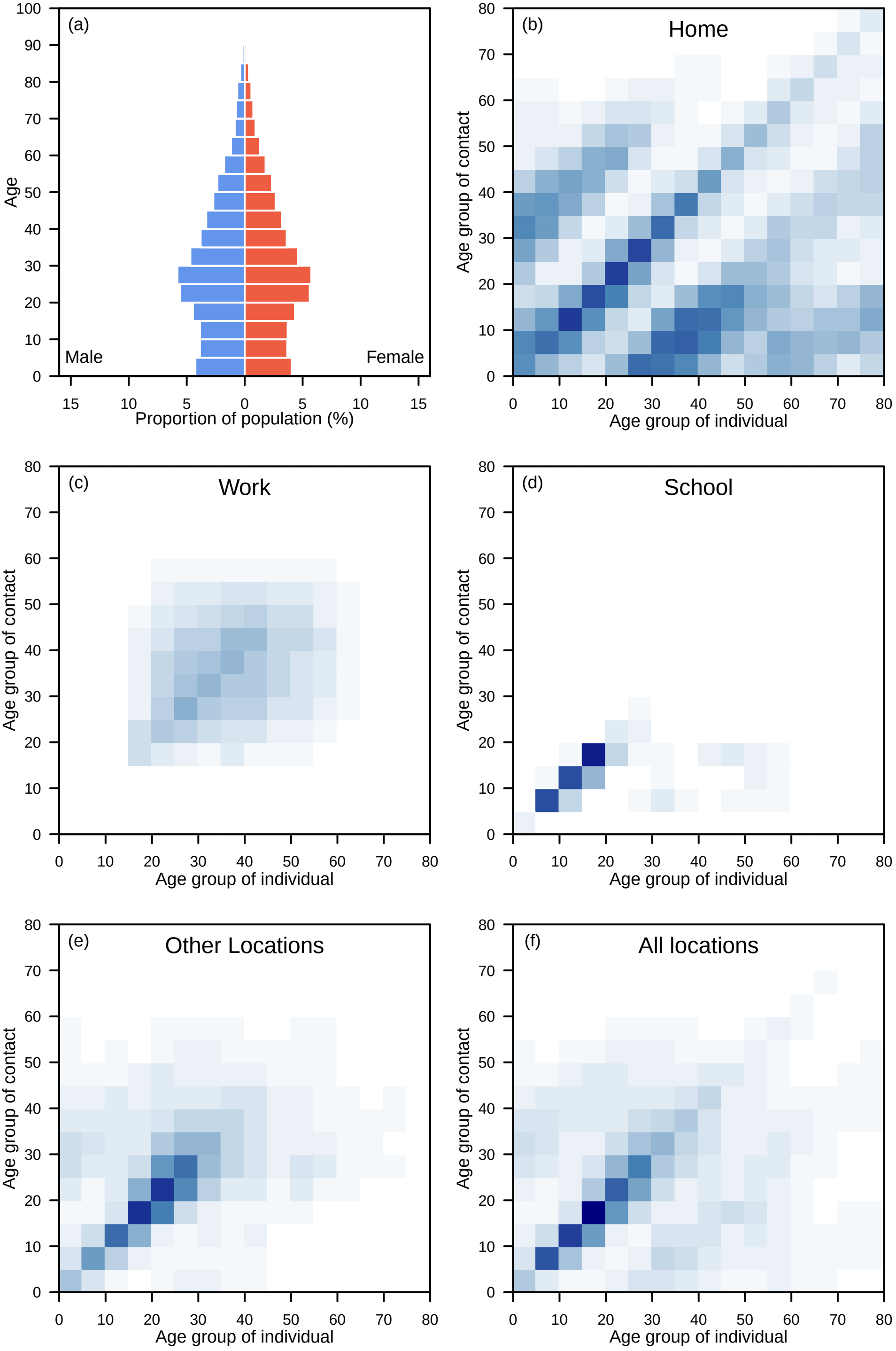

Iraq

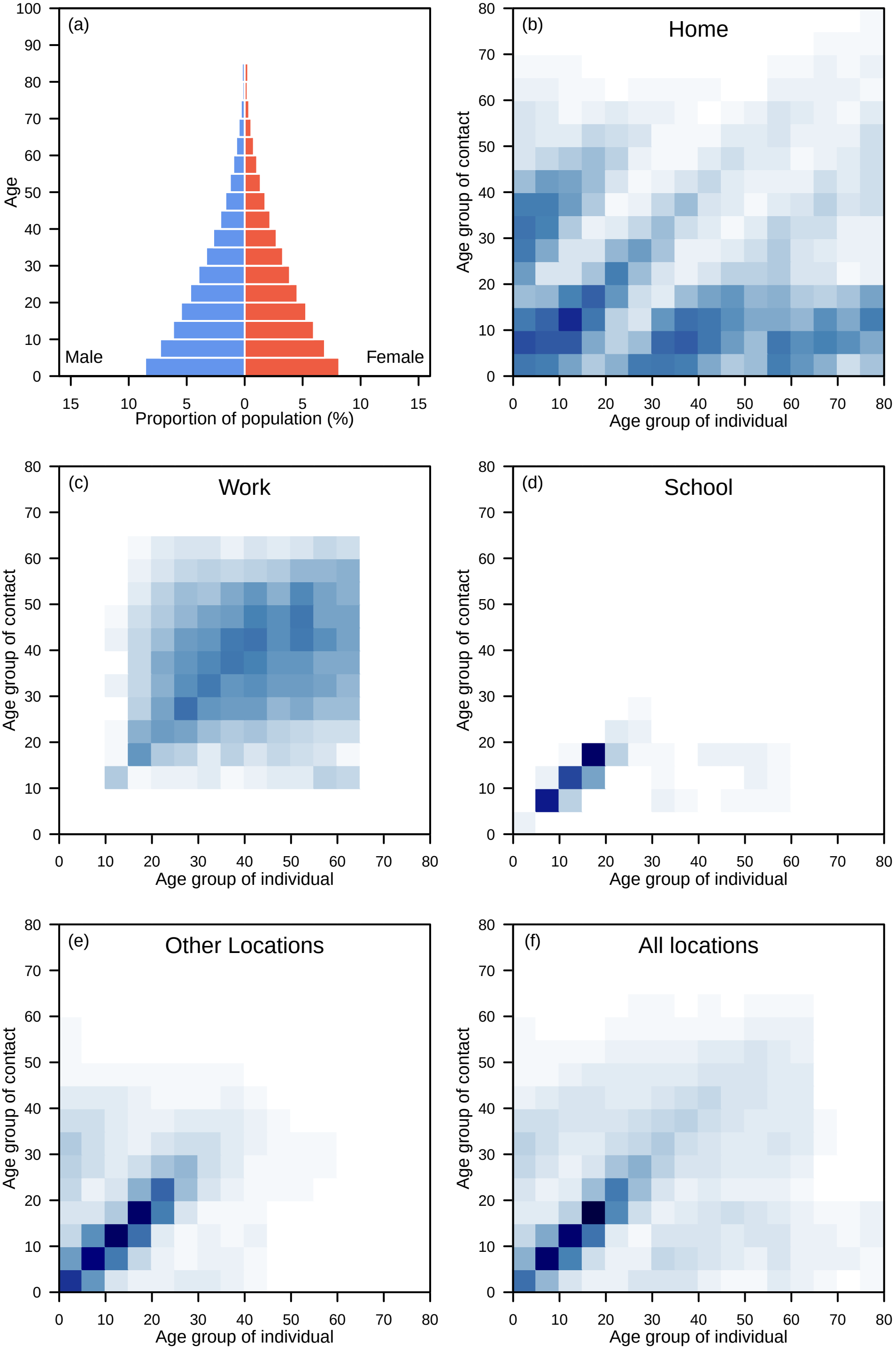
(a)
Age
Male
Female
Proportion of population (%)
(b)
Home
Age group of contact
Age group of individual
(c)
Work
Age group of contact
Age group of individual
(d)
School
Age group of contact
Age group of individual
(e)
Other Locations
Age group of contact
Age group of individual
(f)
All locations
Age group of contact
Age group of individual

Ireland

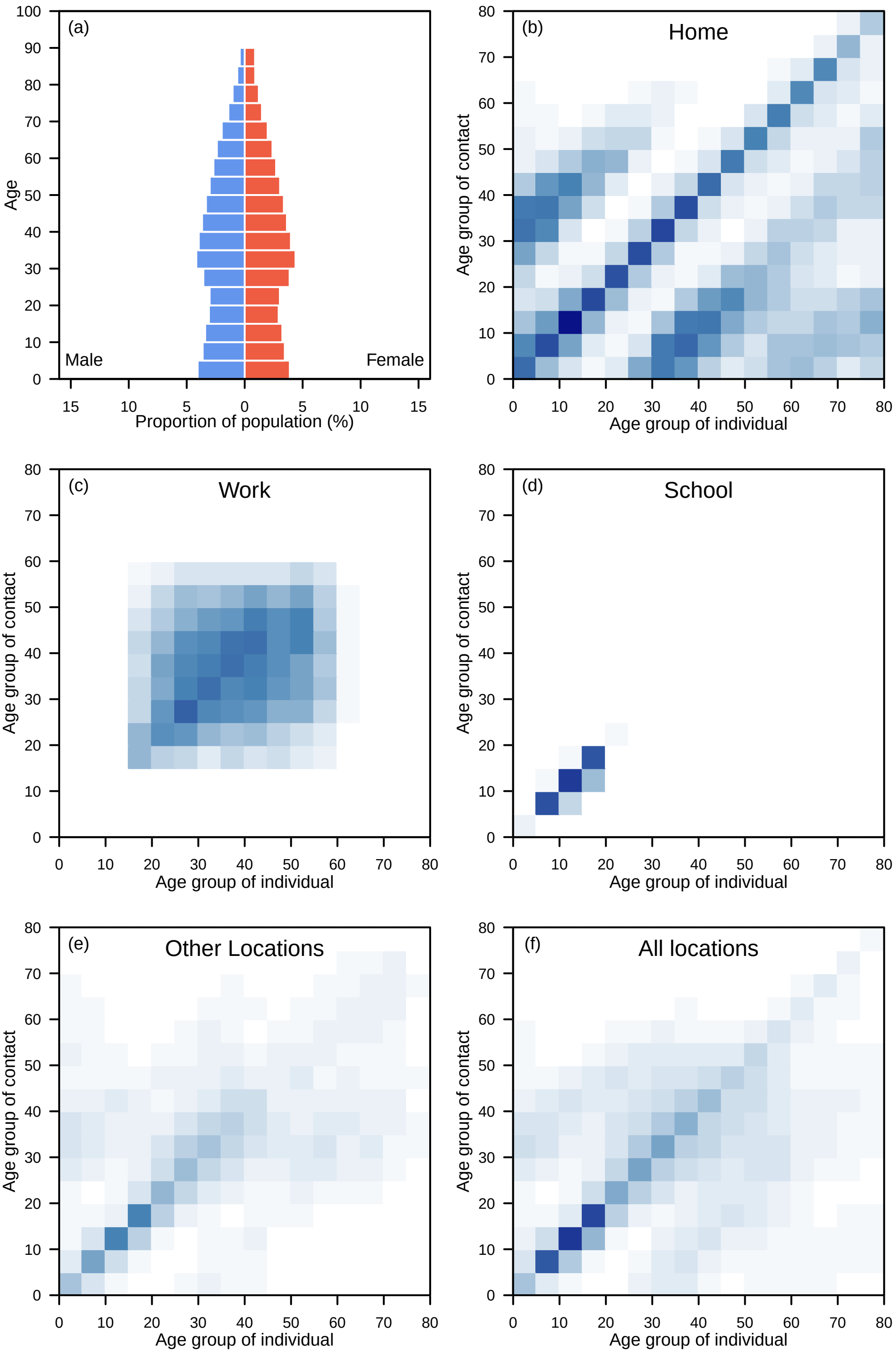

(a)
Age
Male
Female
Proportion of population (%)
(b)
Home
Age group of contact
Age group of individual
(c)
Work
Age group of contact
Age group of individual
(d)
School
Age group of contact
Age group of individual
(e)
Other Locations
Age group of contact
Age group of individual
(f)
All locations
Age group of contact
Age group of individual

Israel

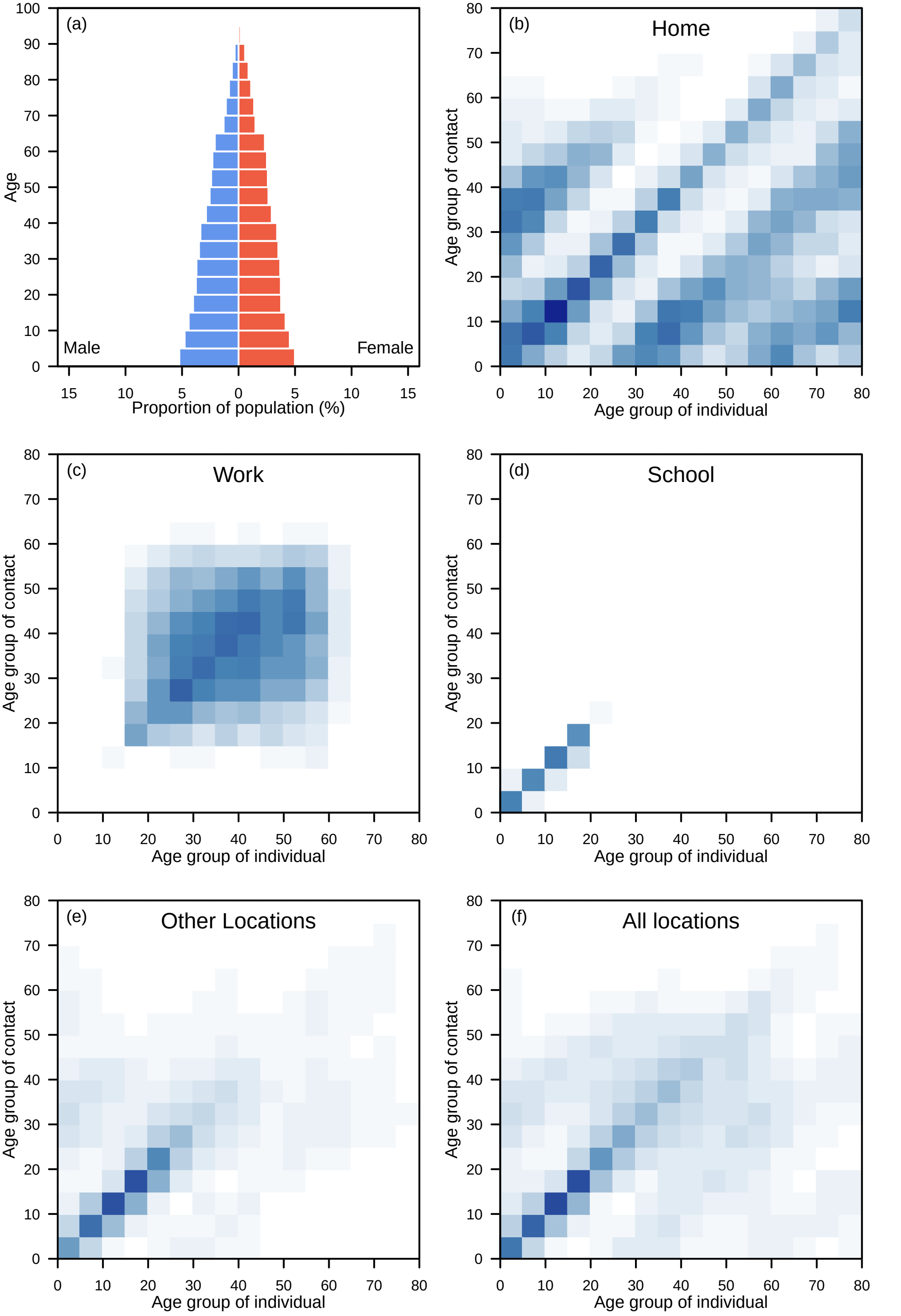

(a)
Male
Female
Age
Proportion of population (%)
100
90
80
70
60
50
40
30
20
10
0
15
10
5
0
5
10
15
(b)
Home
Age group of contact
Age group of individual
(c)
Work
(d)
School
(e)
Other Locations
(f)
All locations
0
10
20
30
40
50
60
70
80

Italy

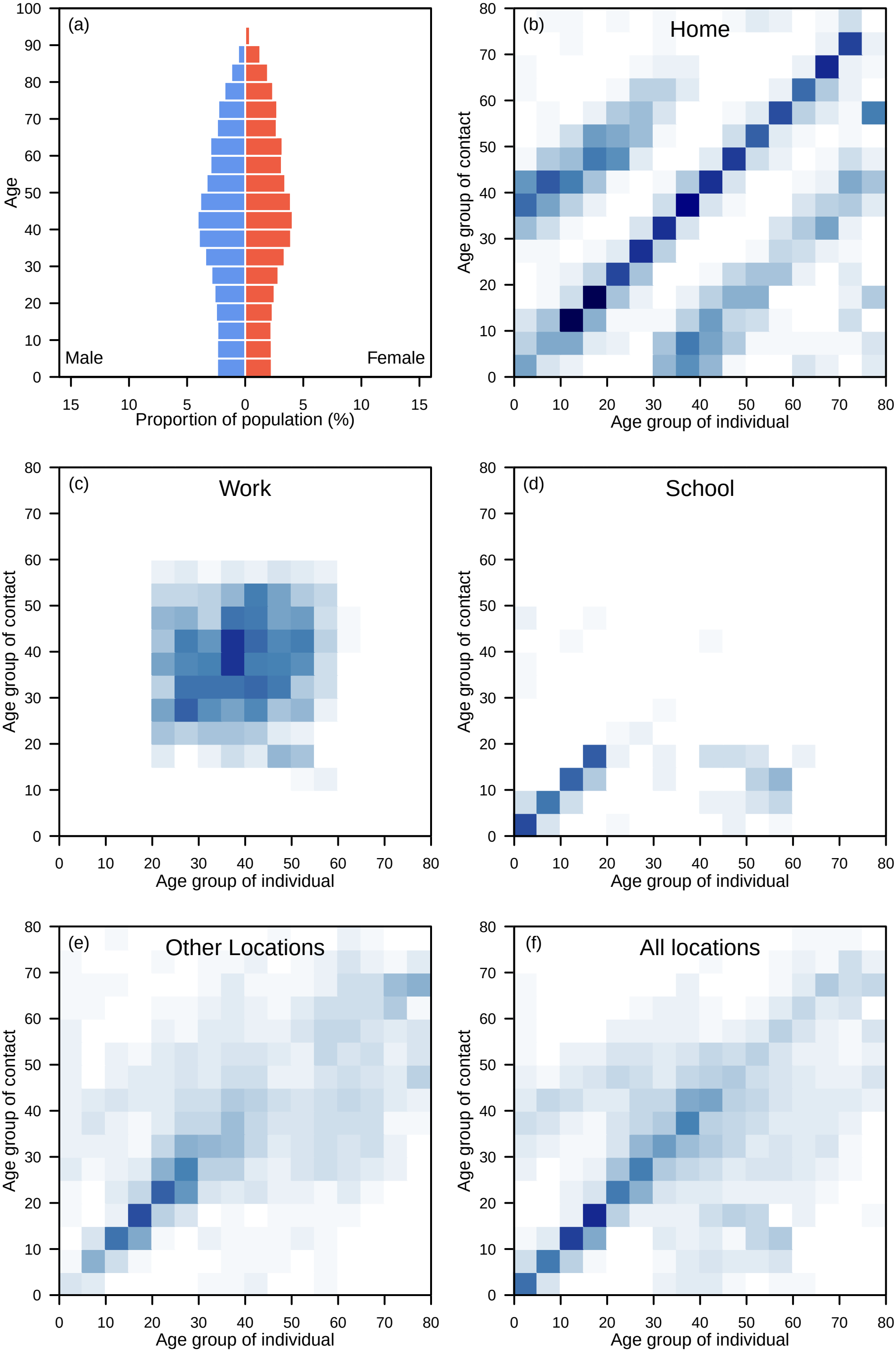

(a)
Age
Male
Female
Proportion of population (%)
(b)
Home
Age group of contact
Age group of individual
(c)
Work
(d)
School
(e)
Other Locations
(f)
All locations

Jamaica

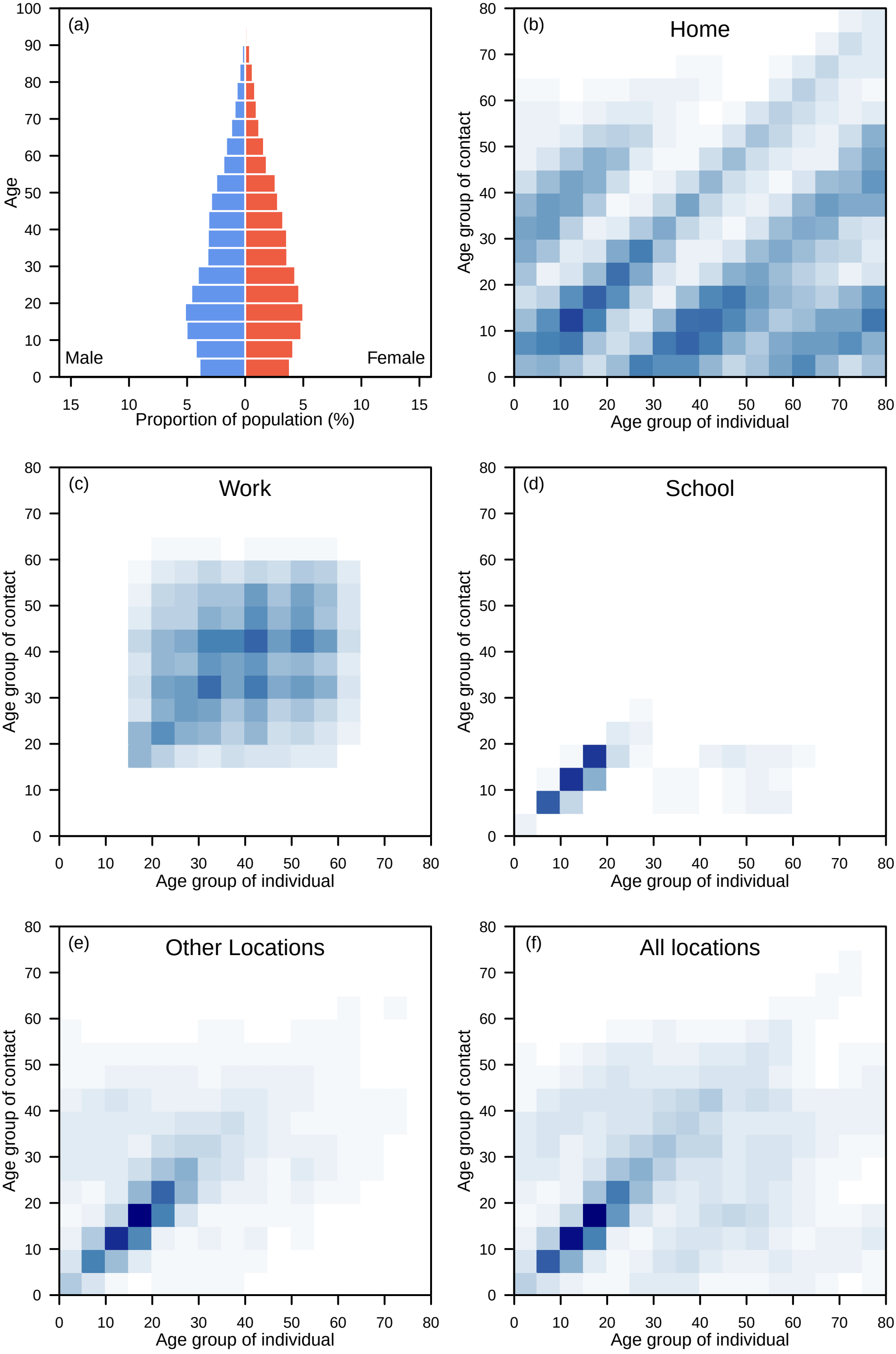

(a)
Age
Male
Female
Proportion of population (%)
(b)
Home
(c)
Work
(d)
School
(e)
Other Locations
(f)
All locations
Age group of contact
Age group of individual

Japan

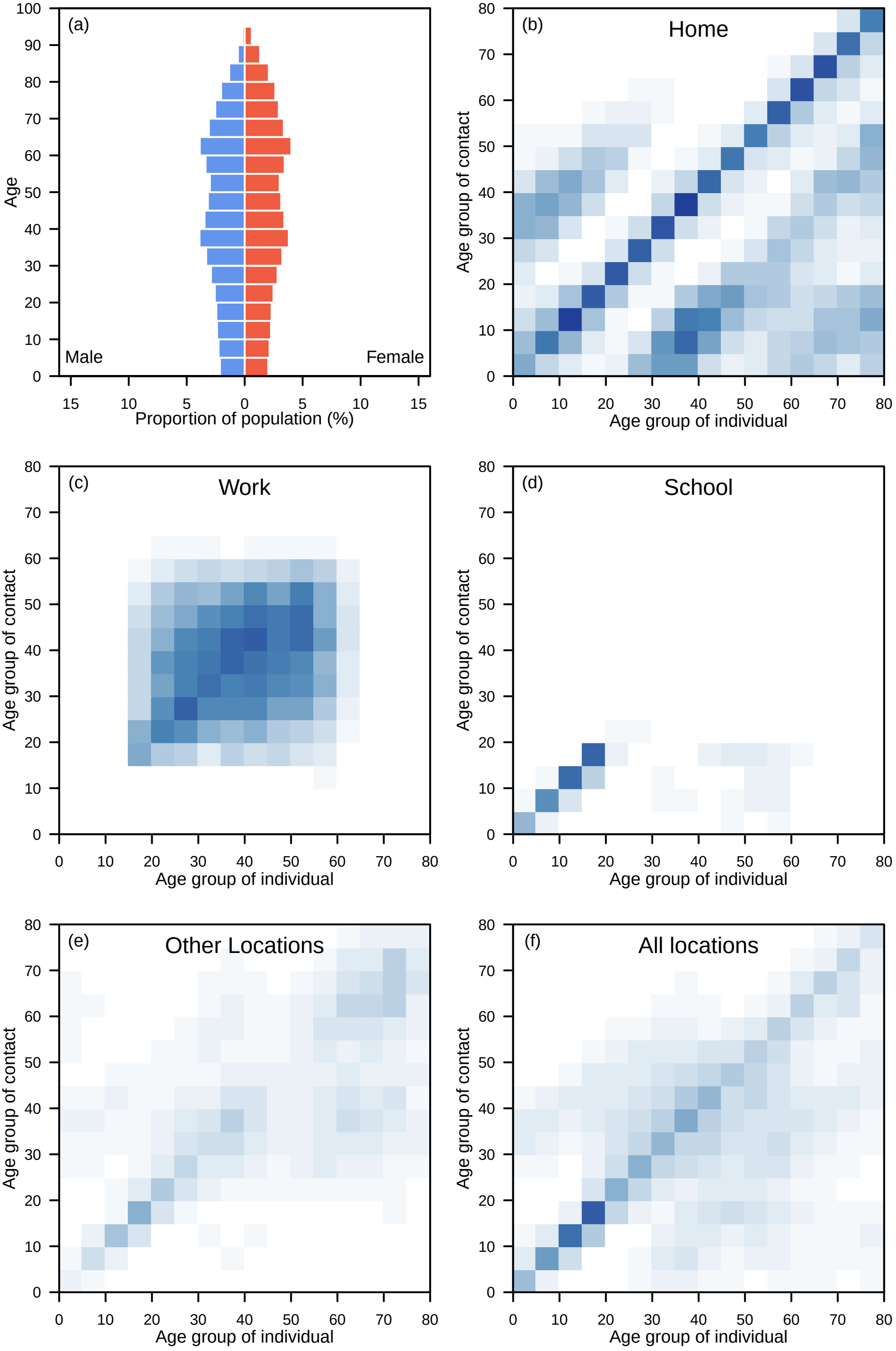
(a)
Age
Male
Female
Proportion of population (%)
(b)
Home
(c)
Work
(d)
School
(e)
Other Locations
(f)
All locations
Age group of contact
Age group of individual

Jordan

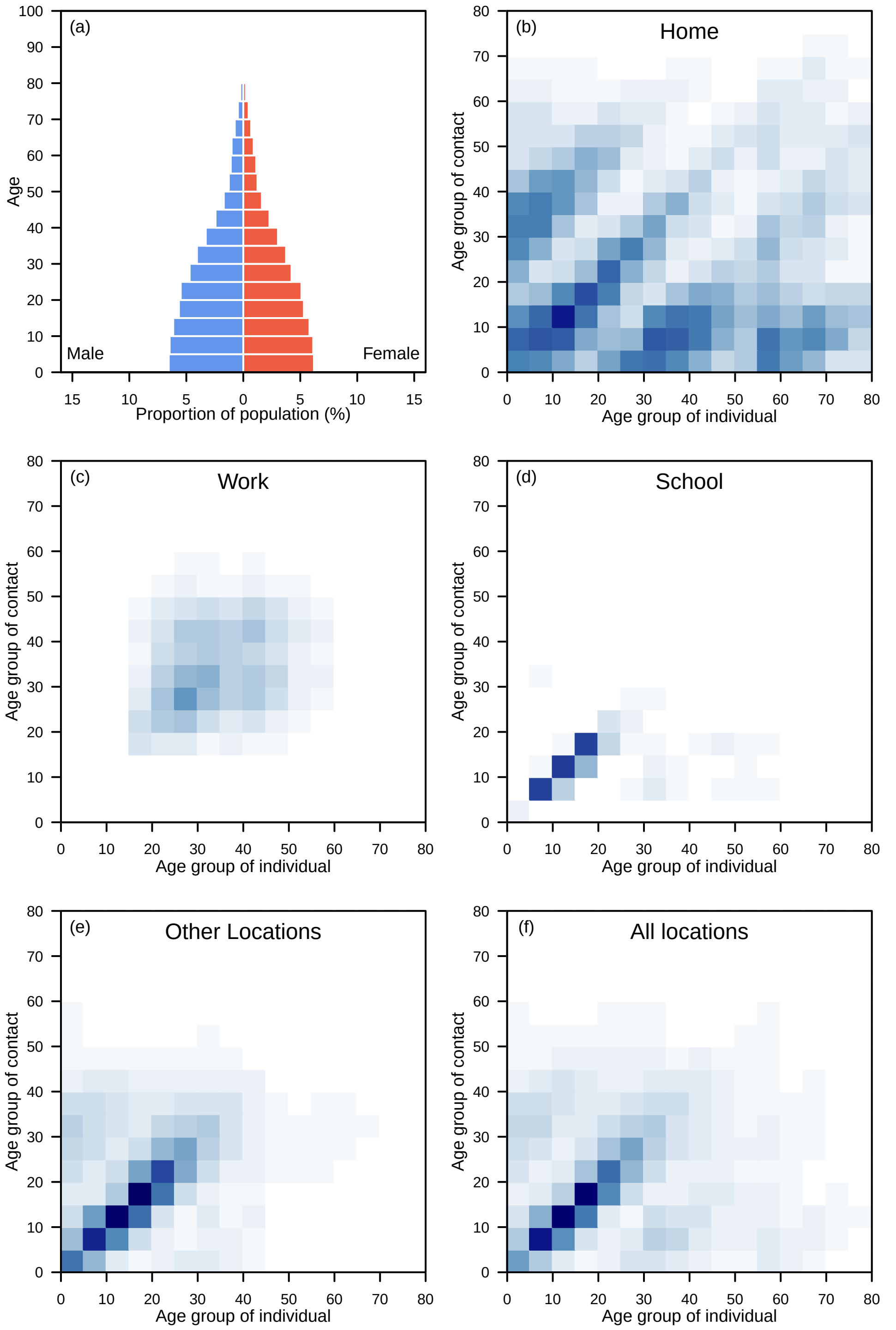

(a)
Age
Male
Female
Proportion of population (%)
(b)
Home
Age group of contact
Age group of individual
(c)
Work
Age group of contact
Age group of individual
(d)
School
Age group of contact
Age group of individual
(e)
Other Locations
Age group of contact
Age group of individual
(f)
All locations
Age group of contact
Age group of individual

Kazakhstan

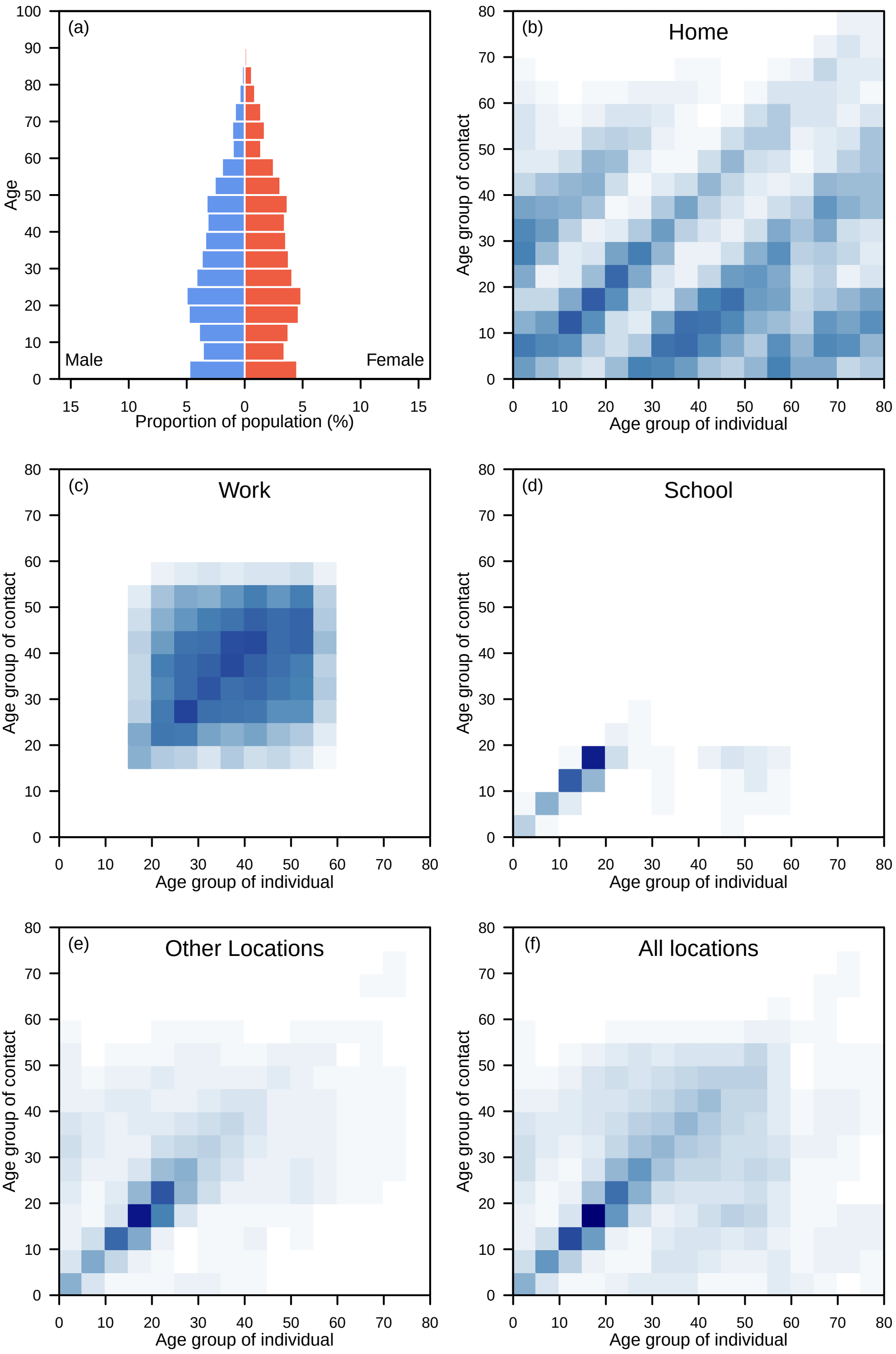

(a)
Age
Male
Female
Proportion of population (%)
(b)
Home
(c)
Work
(d)
School
(e)
Other Locations
(f)
All locations
Age group of contact
Age group of individual

Kenya

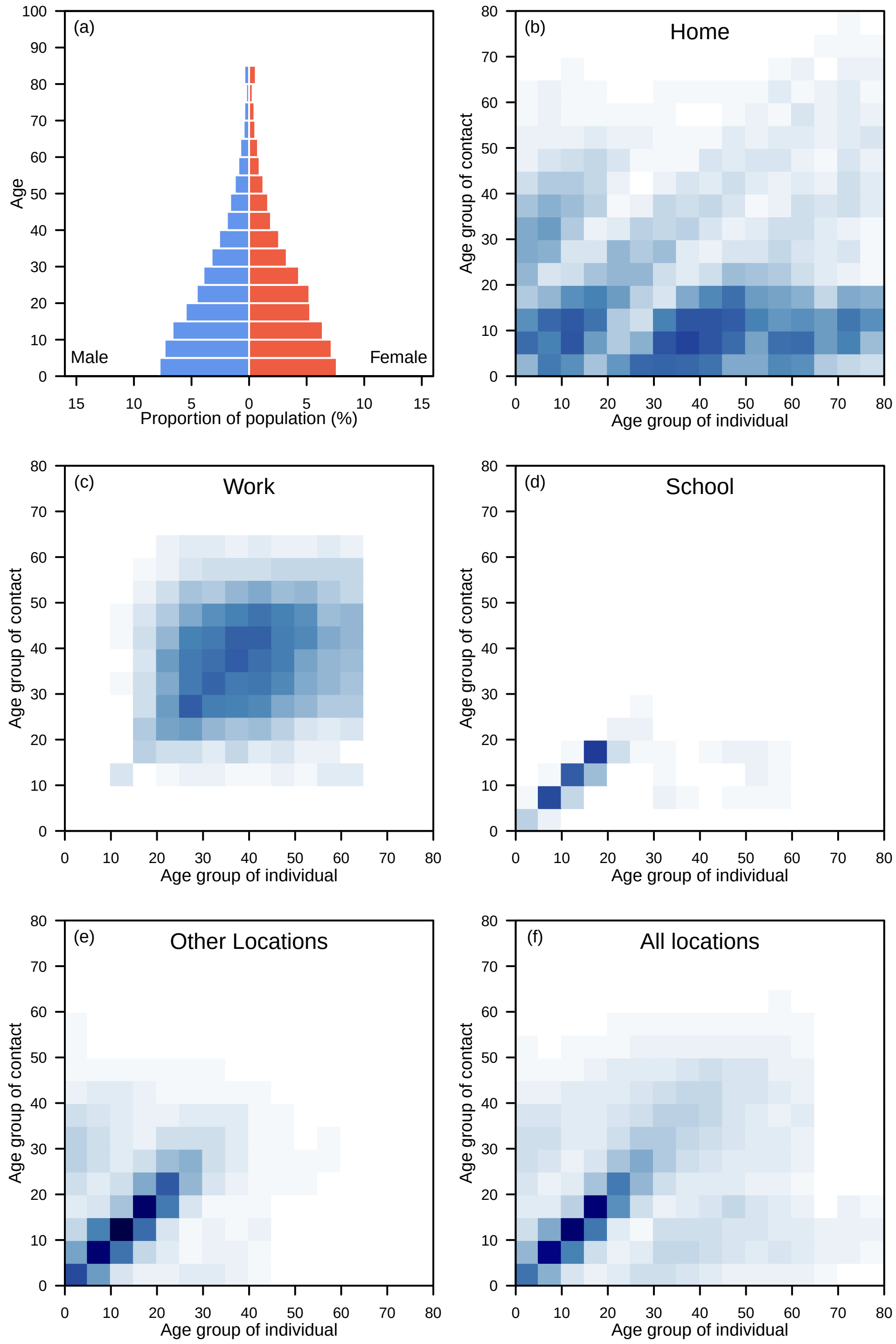

(a)
Age
Male
Female
Proportion of population (%)
(b)
Home
(c)
Work
(d)
School
(e)
Other Locations
(f)
All locations
Age group of contact
Age group of individual

Kiribati

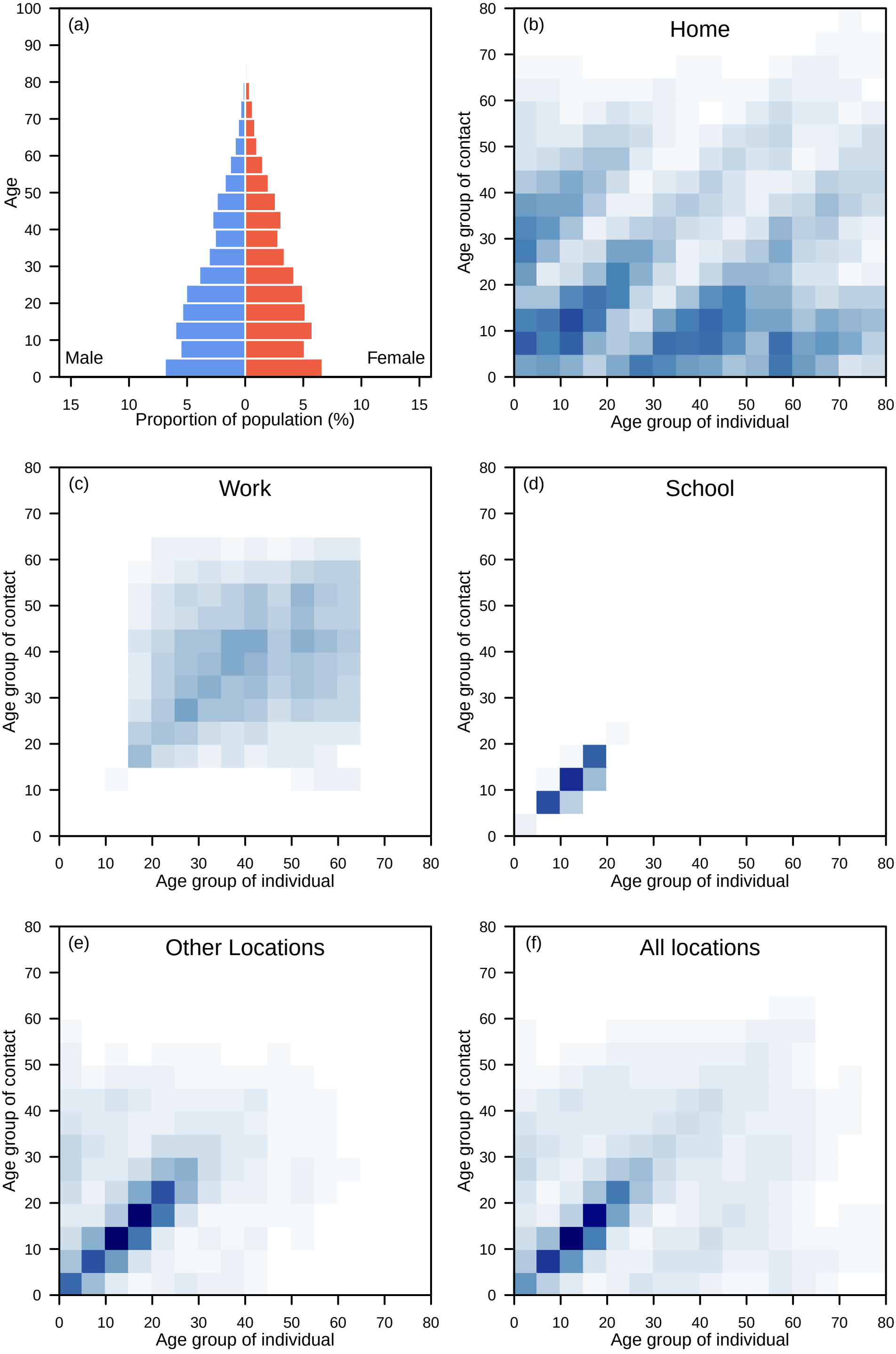

(a)
Age
Male
Female
Proportion of population (%)
(b)
Home
Age group of contact
Age group of individual
(c)
Work
Age group of contact
Age group of individual
(d)
School
Age group of contact
Age group of individual
(e)
Other Locations
Age group of contact
Age group of individual
(f)
All locations
Age group of contact
Age group of individual

Kuwait

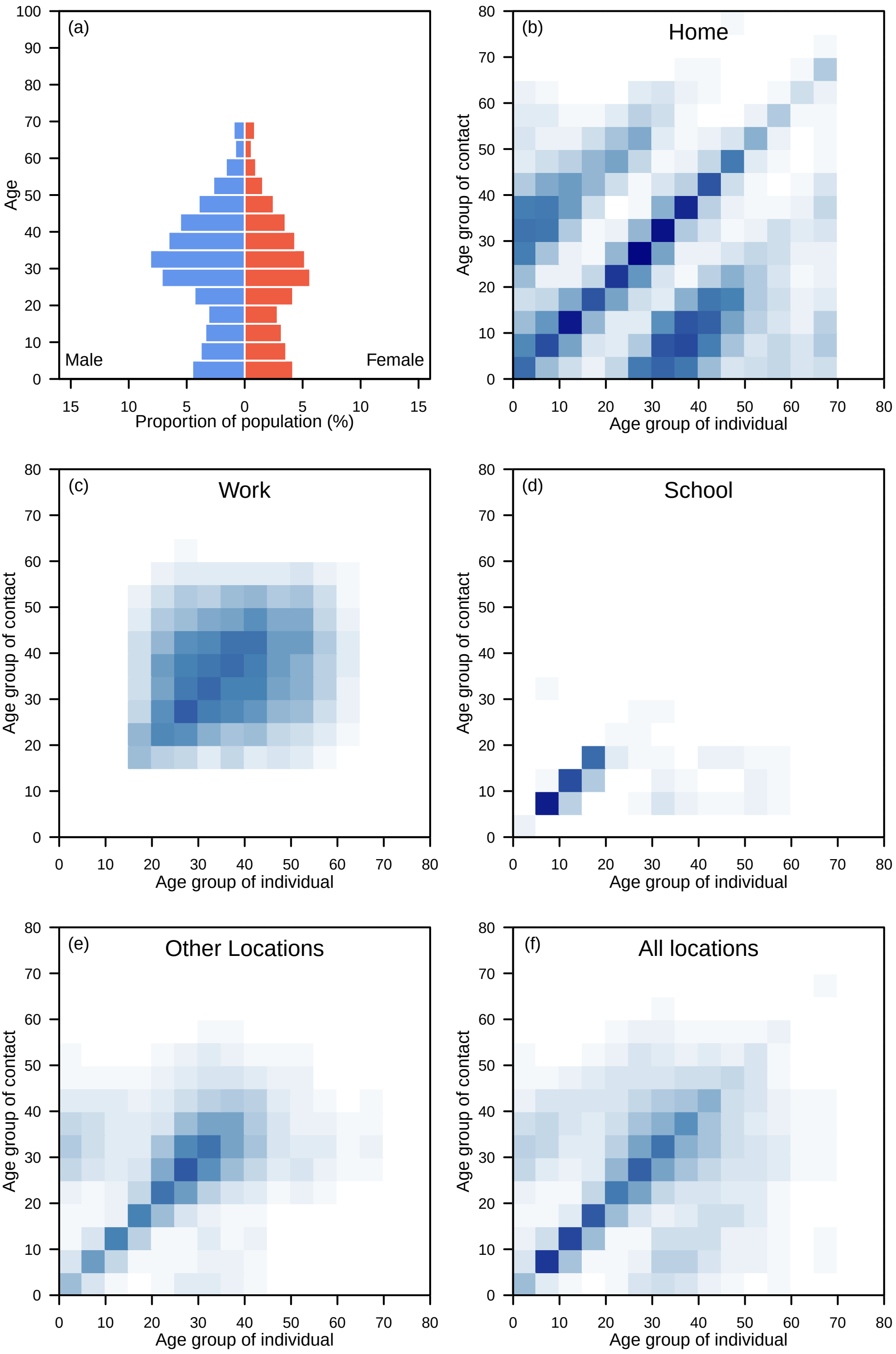
(a)
Age
Male
Female
Proportion of population (%)
(b)
Home
Age group of contact
Age group of individual
(c)
Work
Age group of contact
Age group of individual
(d)
School
Age group of contact
Age group of individual
(e)
Other Locations
Age group of contact
Age group of individual
(f)
All locations
Age group of contact
Age group of individual

Kyrgyzstan

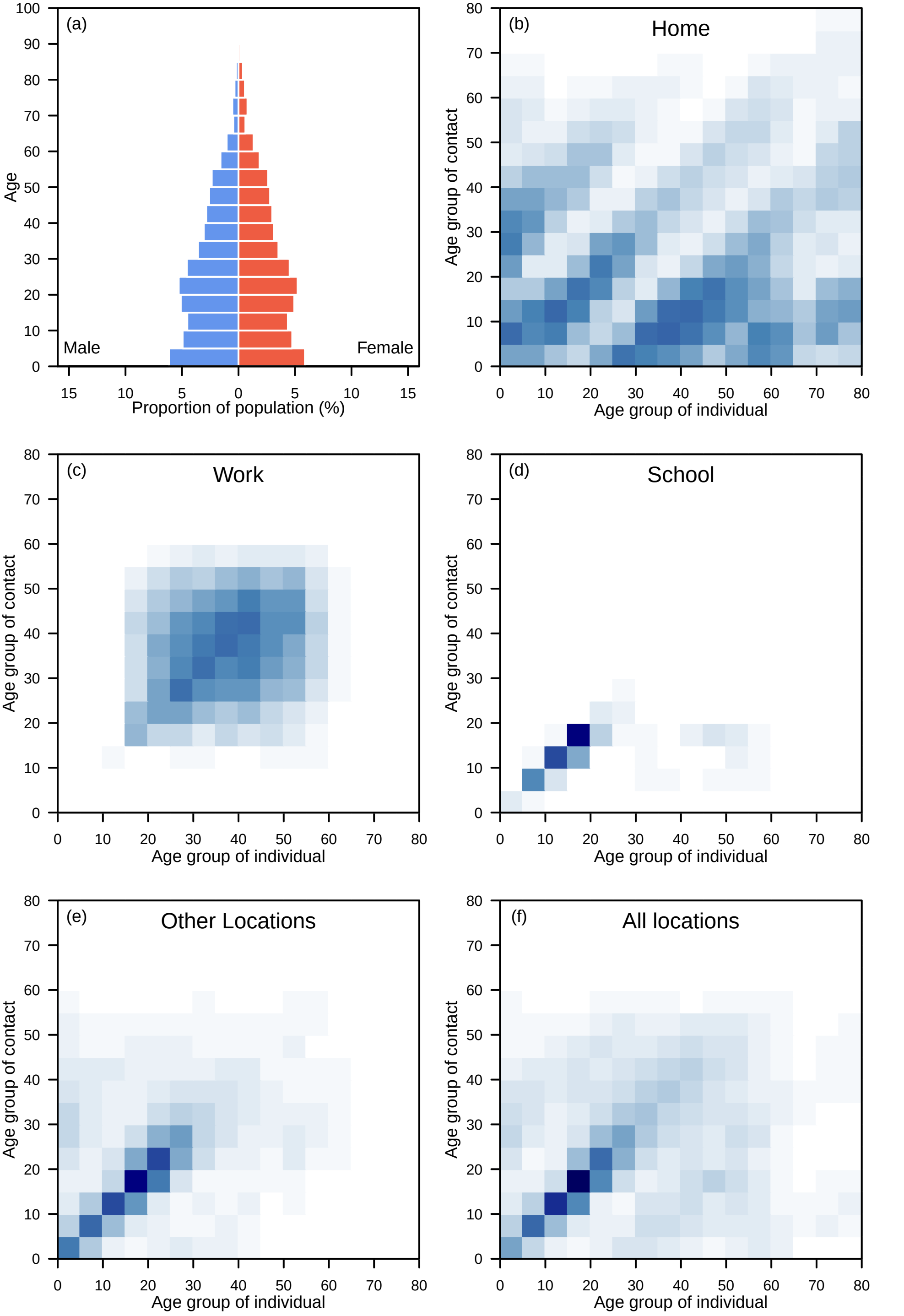

(a)
Age
Male
Female
Proportion of population (%)
(b)
Home
Age group of contact
Age group of individual
(c)
Work
Age group of contact
Age group of individual
(d)
School
Age group of contact
Age group of individual
(e)
Other Locations
Age group of contact
Age group of individual
(f)
All locations
Age group of contact
Age group of individual

Lao People's Democratic Republic

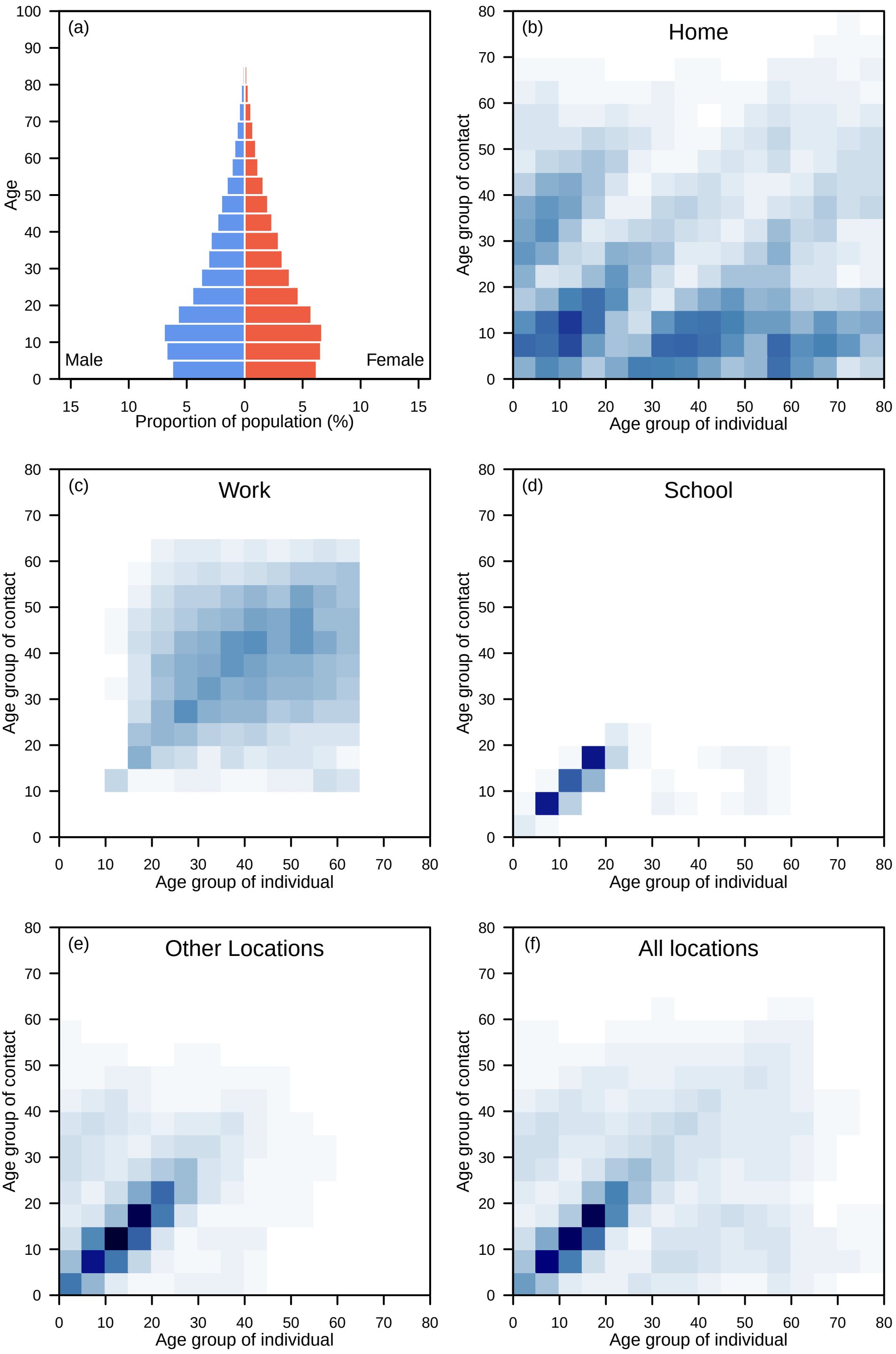

Latvia

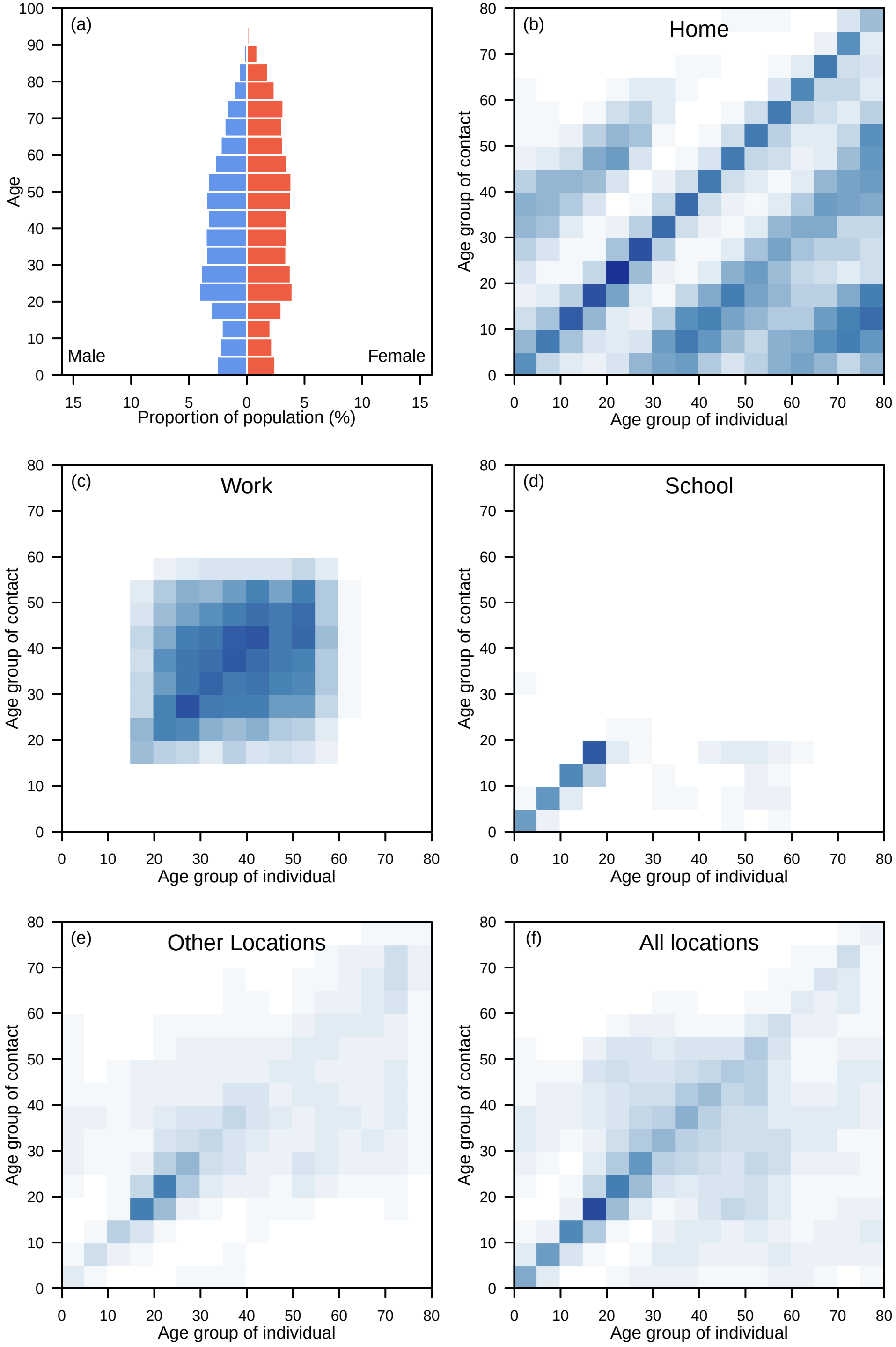

(a)
Age
Male
Female
Proportion of population (%)
(b)
Home
(c)
Work
(d)
School
(e)
Other Locations
(f)
All locations
Age group of contact
Age group of individual

Lebanon

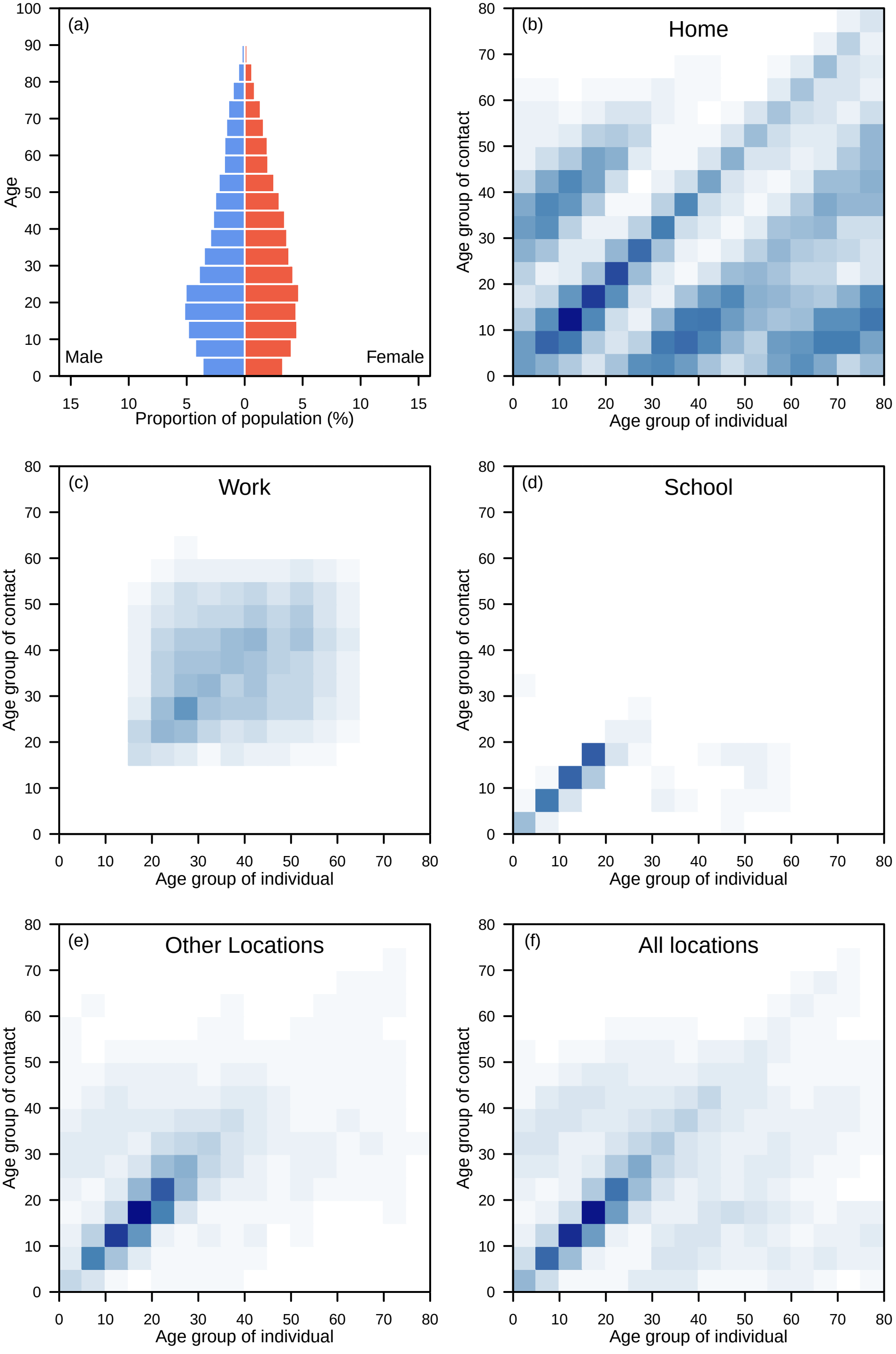
(a)
Age
Male
Female
Proportion of population (%)
(b)
Home
(c)
Work
(d)
School
(e)
Other Locations
(f)
All locations
Age group of contact
Age group of individual

Lesotho

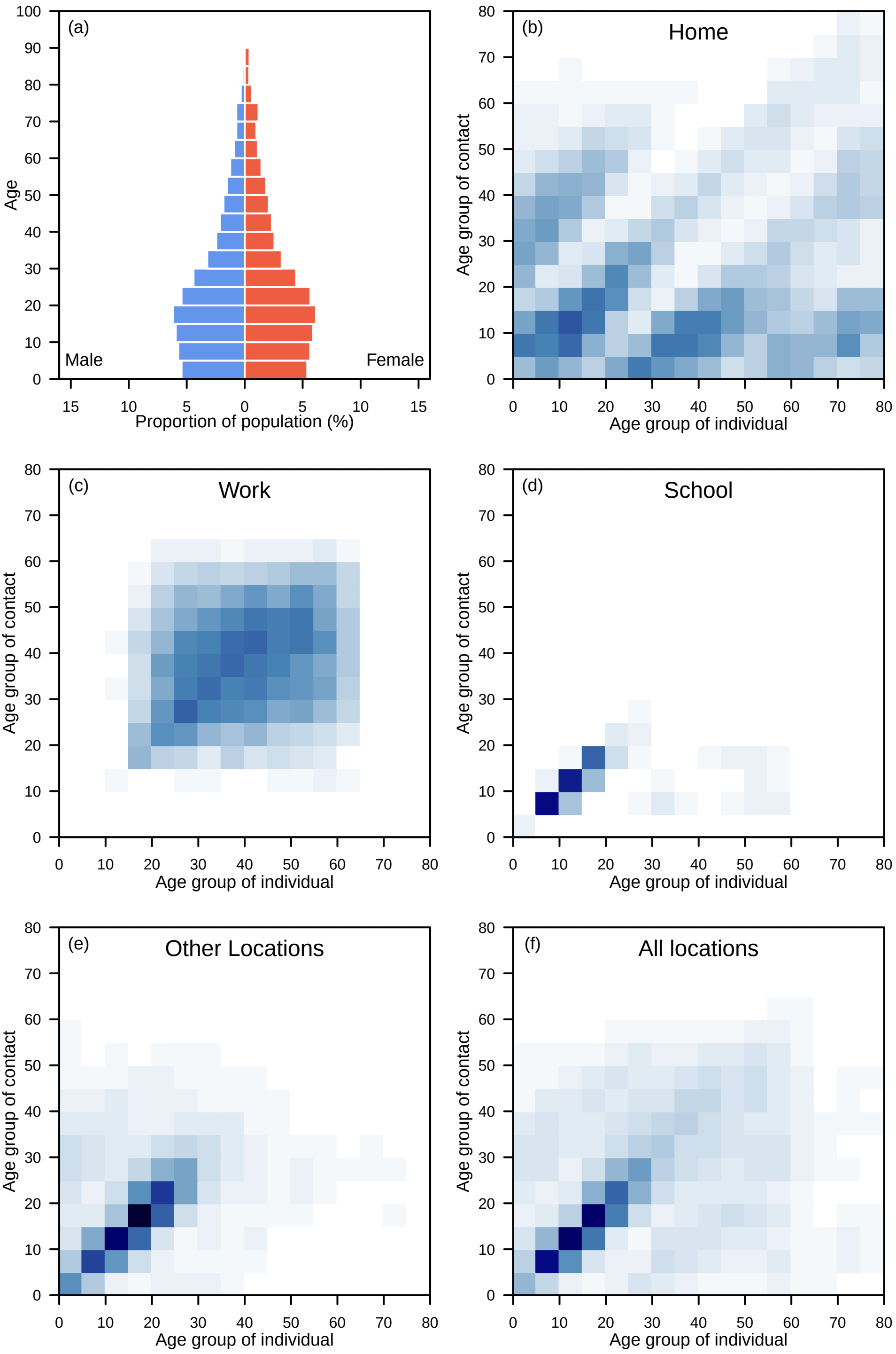

(a)
Age
Male
Female
Proportion of population (%)
(b)
Home
Age group of contact
Age group of individual
(c)
Work
Age group of contact
Age group of individual
(d)
School
Age group of contact
Age group of individual
(e)
Other Locations
Age group of contact
Age group of individual
(f)
All locations
Age group of contact
Age group of individual

Liberia

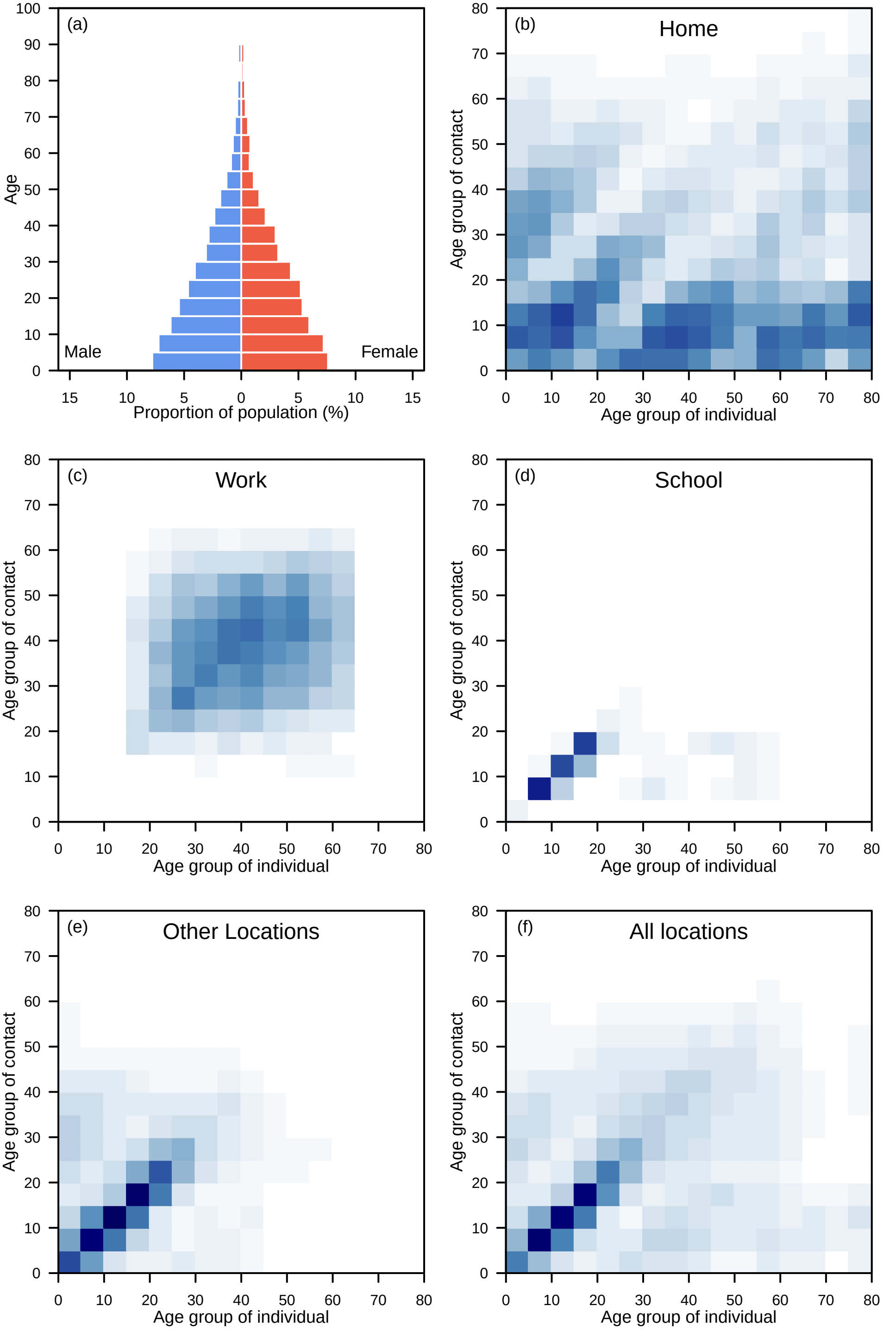

(a)
Age
Male
Female
Proportion of population (%)
(b)
Home
Age group of contact
Age group of individual
(c)
Work
(d)
School
(e)
Other Locations
(f)
All locations

Lithuania

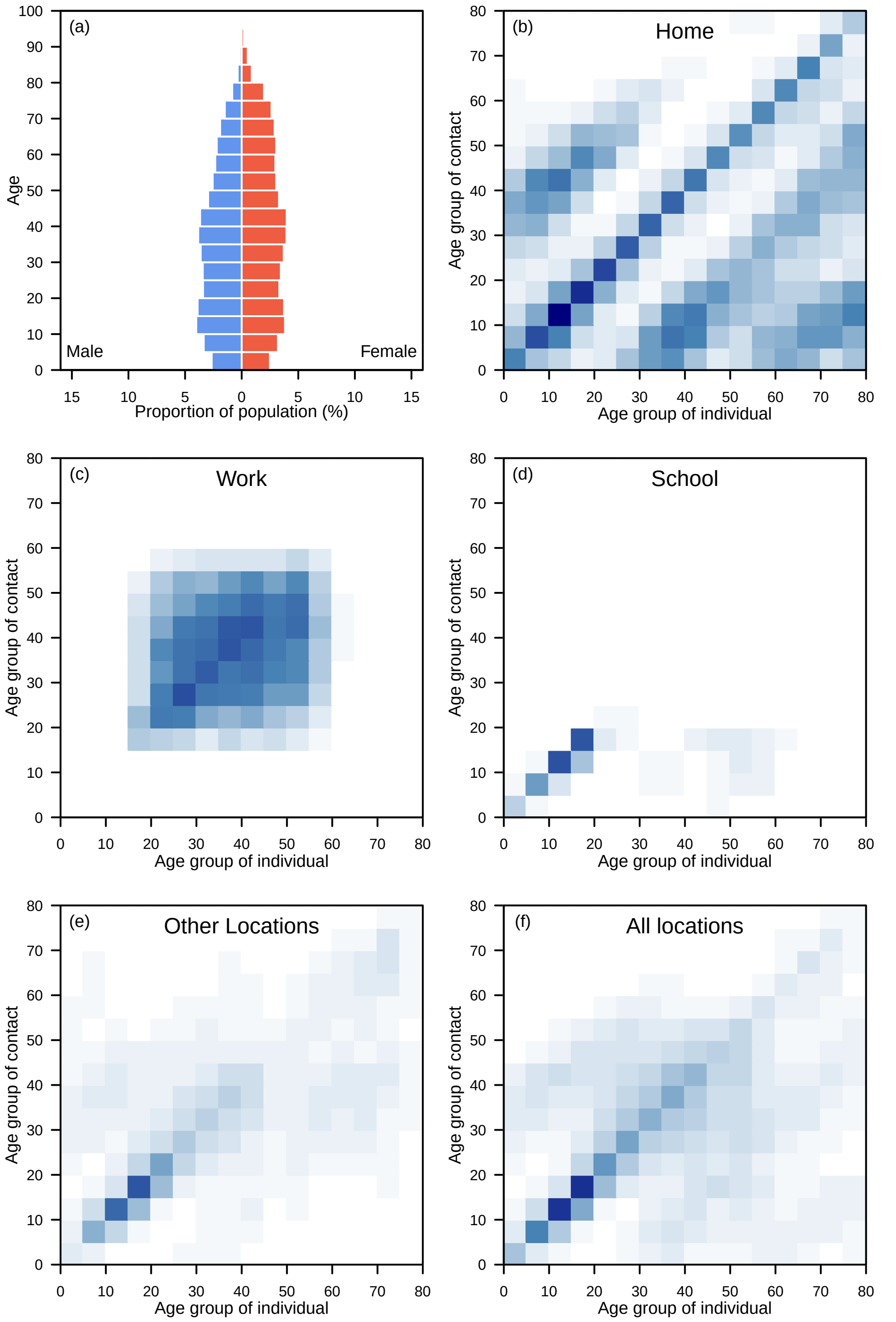

(a)
Male
Female
Age
Proportion of population (%)
(b)
Home
(c)
Work
(d)
School
(e)
Other Locations
(f)
All locations
Age group of contact
Age group of individual

Luxembourg

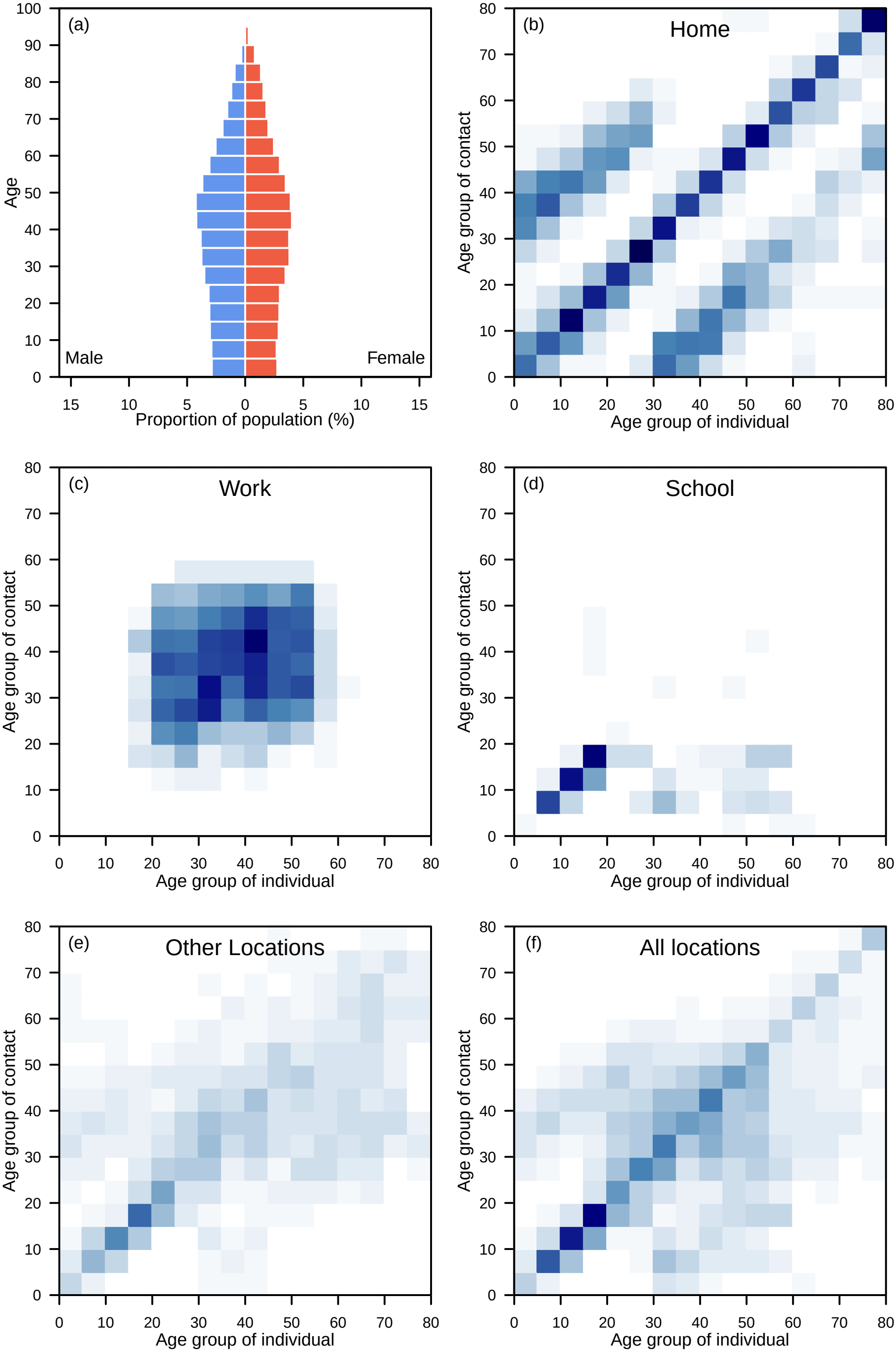
(a)
Age
Male
Female
Proportion of population (%)
(b)
Home
Age group of contact
Age group of individual
(c)
Work
Age group of contact
Age group of individual
(d)
School
Age group of contact
Age group of individual
(e)
Other Locations
Age group of contact
Age group of individual
(f)
All locations
Age group of contact
Age group of individual

Malaysia

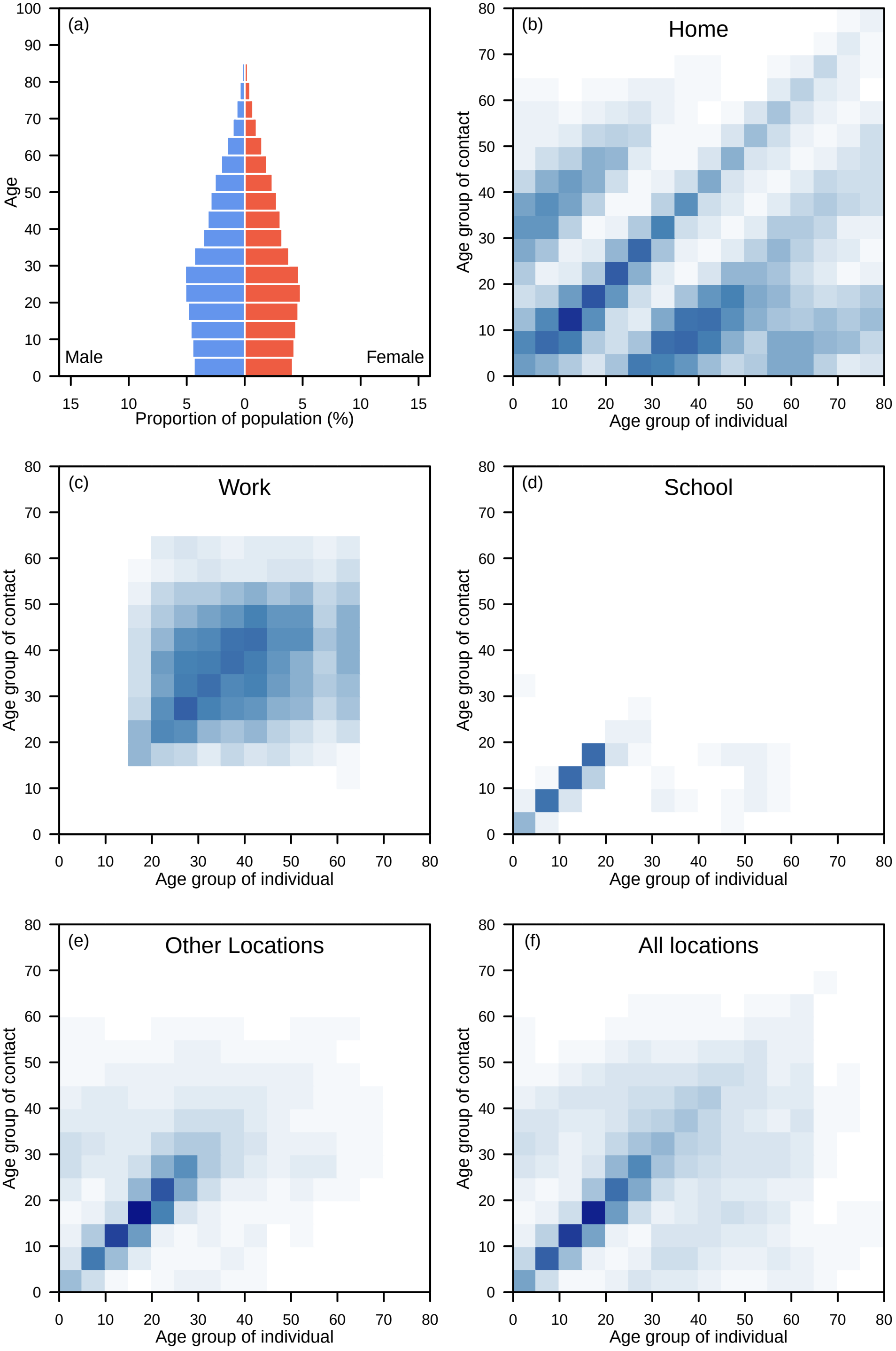

(a)
Age
Male
Female
Proportion of population (%)
(b)
Home
Age group of contact
Age group of individual
(c)
Work
Age group of contact
Age group of individual
(d)
School
Age group of contact
Age group of individual
(e)
Other Locations
Age group of contact
Age group of individual
(f)
All locations
Age group of contact
Age group of individual

Maldives

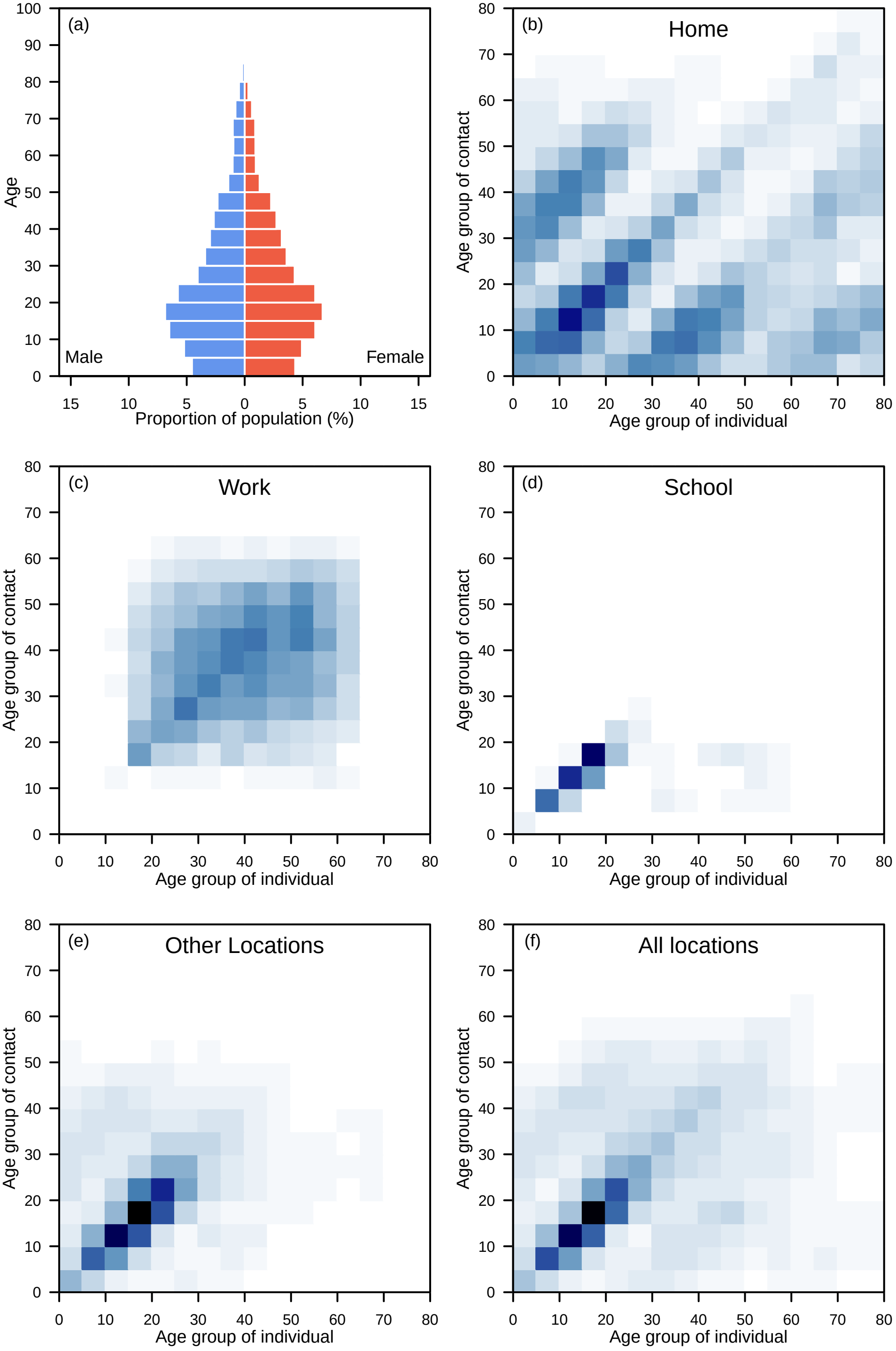

(a)
Age
Male
Female
Proportion of population (%)
(b)
Home
Age group of contact
Age group of individual
(c)
Work
(d)
School
(e)
Other Locations
(f)
All locations

Malta

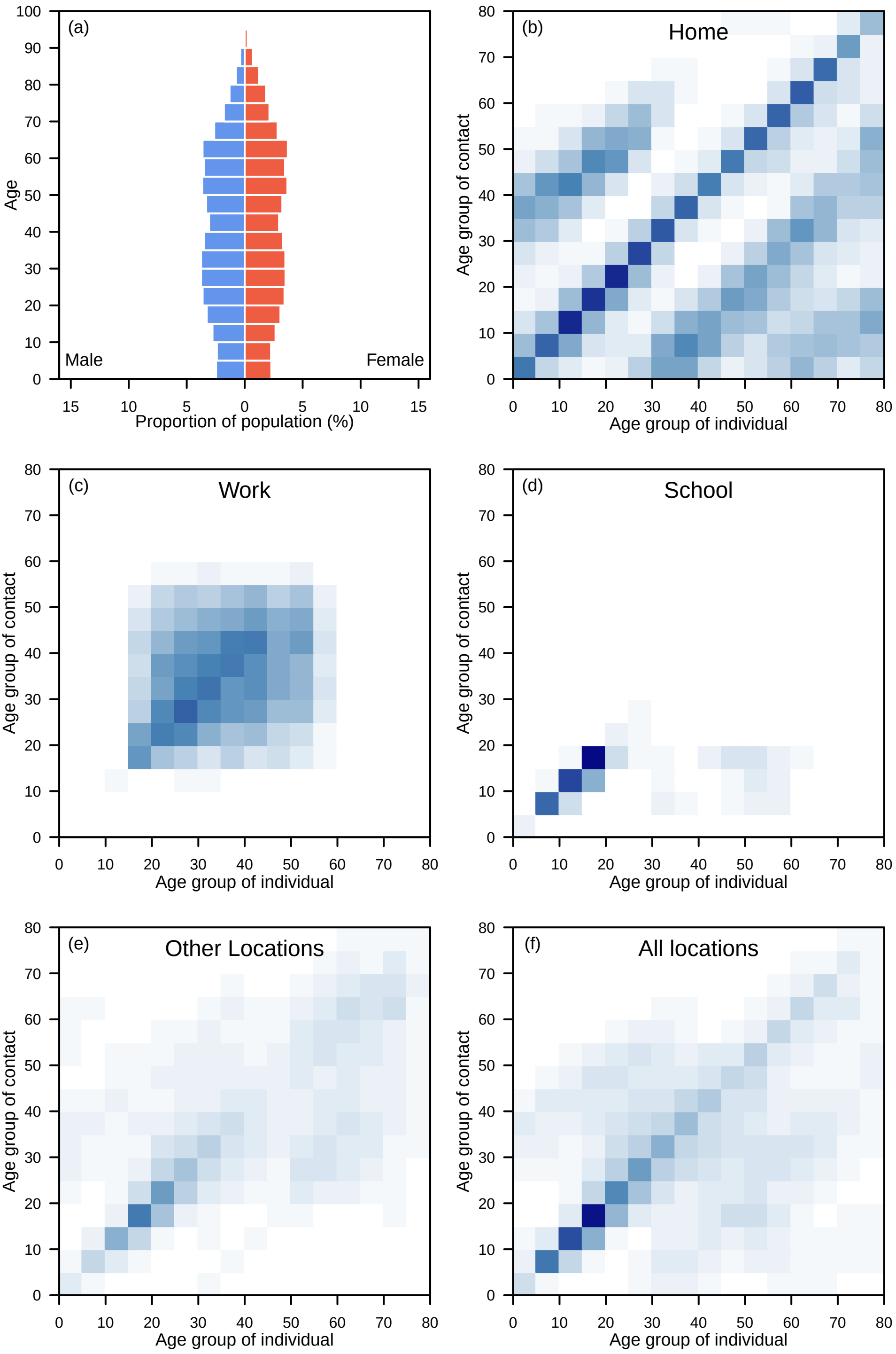

(a)
Male
Female
Age
Proportion of population (%)
(b)
Home
(c)
Work
(d)
School
(e)
Other Locations
(f)
All locations
Age group of contact
Age group of individual

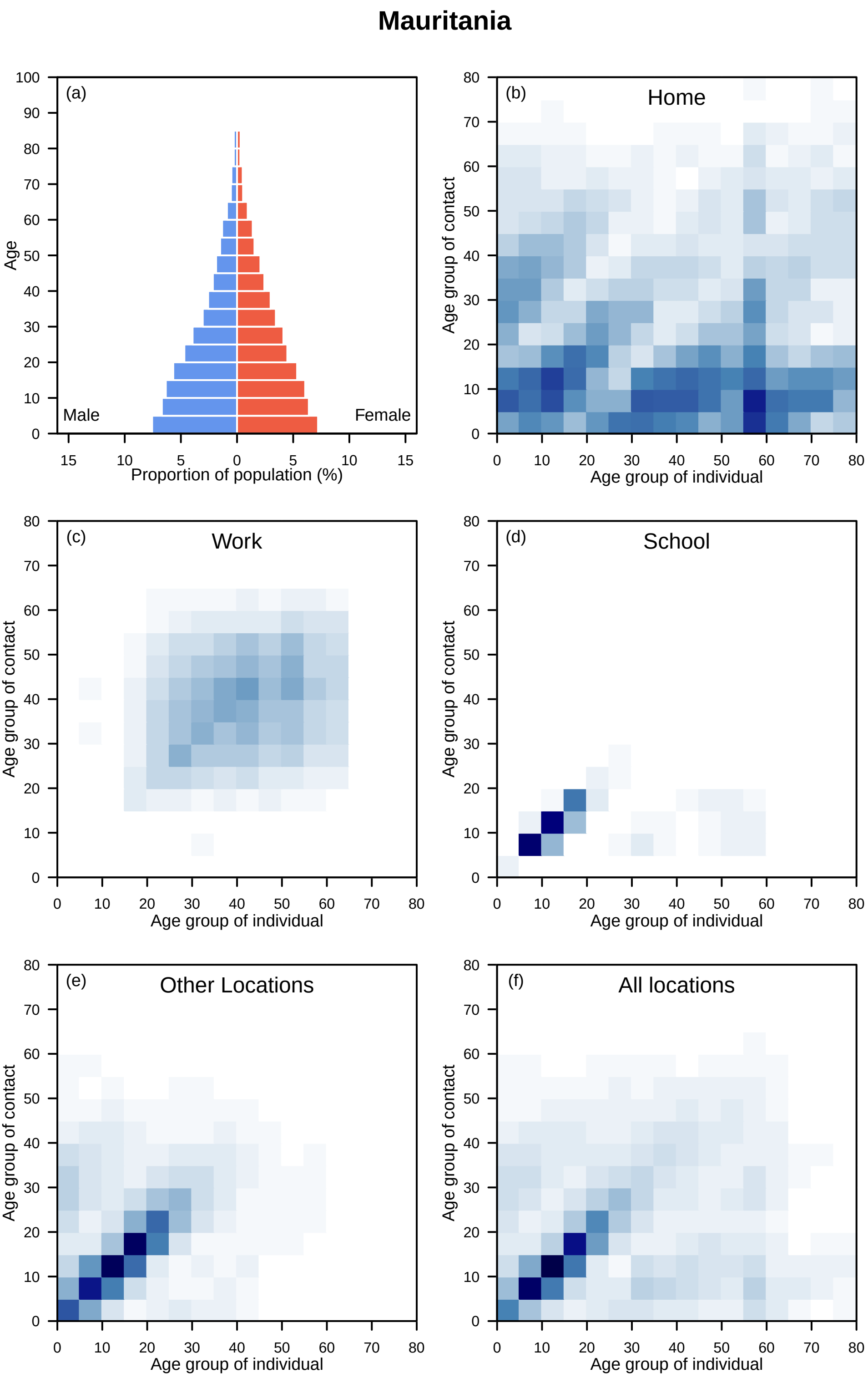
Mauritania
(a)
Age
Male
Female
Proportion of population (%)
(b) Home
Age group of contact
Age group of individual
(c) Work
(d) School
(e) Other Locations
(f) All locations

Mauritius

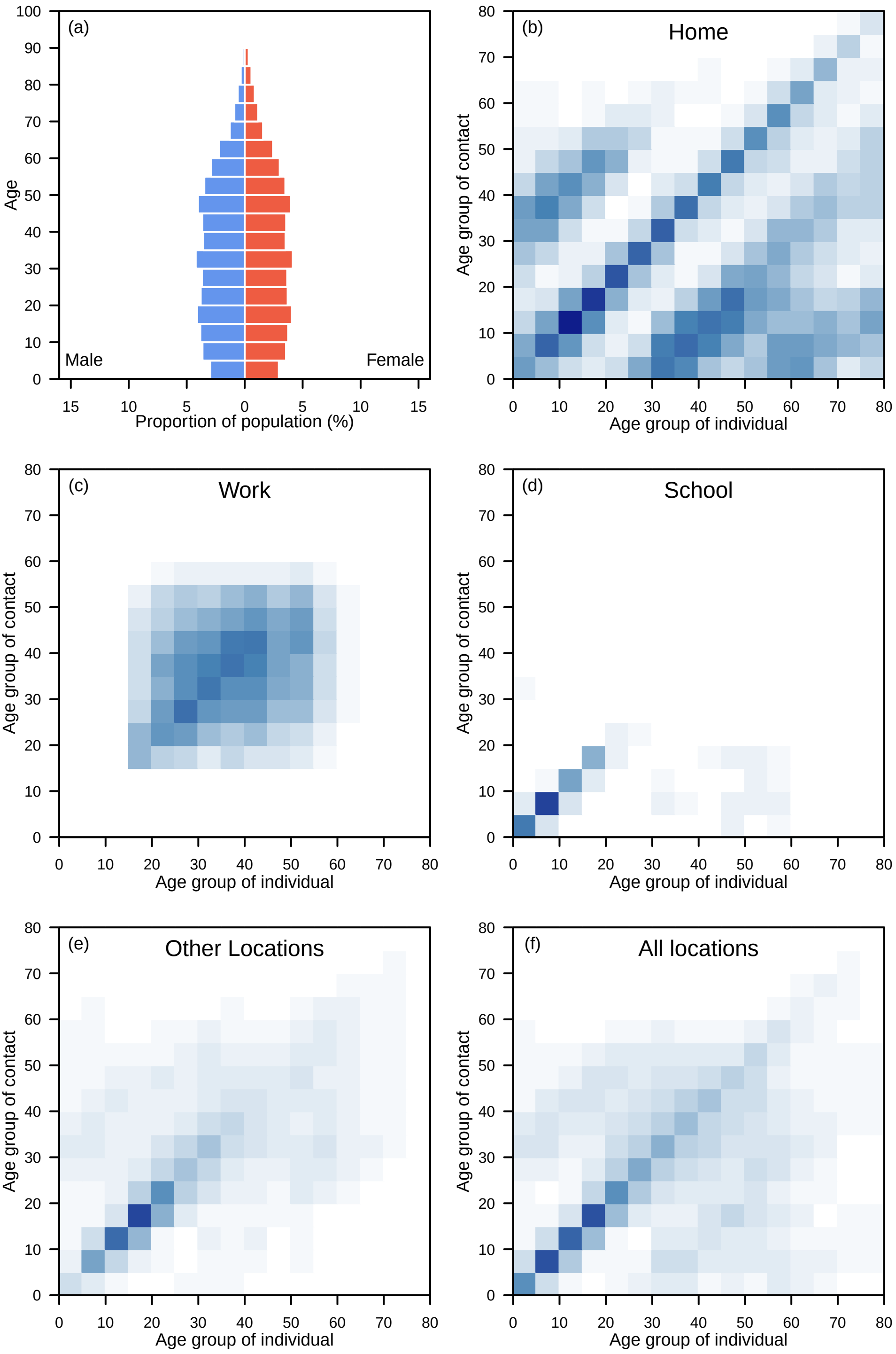

(a)
Age
Male
Female
Proportion of population (%)
(b)
Home
Age group of contact
Age group of individual
(c)
Work
Age group of contact
Age group of individual
(d)
School
Age group of contact
Age group of individual
(e)
Other Locations
Age group of contact
Age group of individual
(f)
All locations
Age group of contact
Age group of individual

Mexico

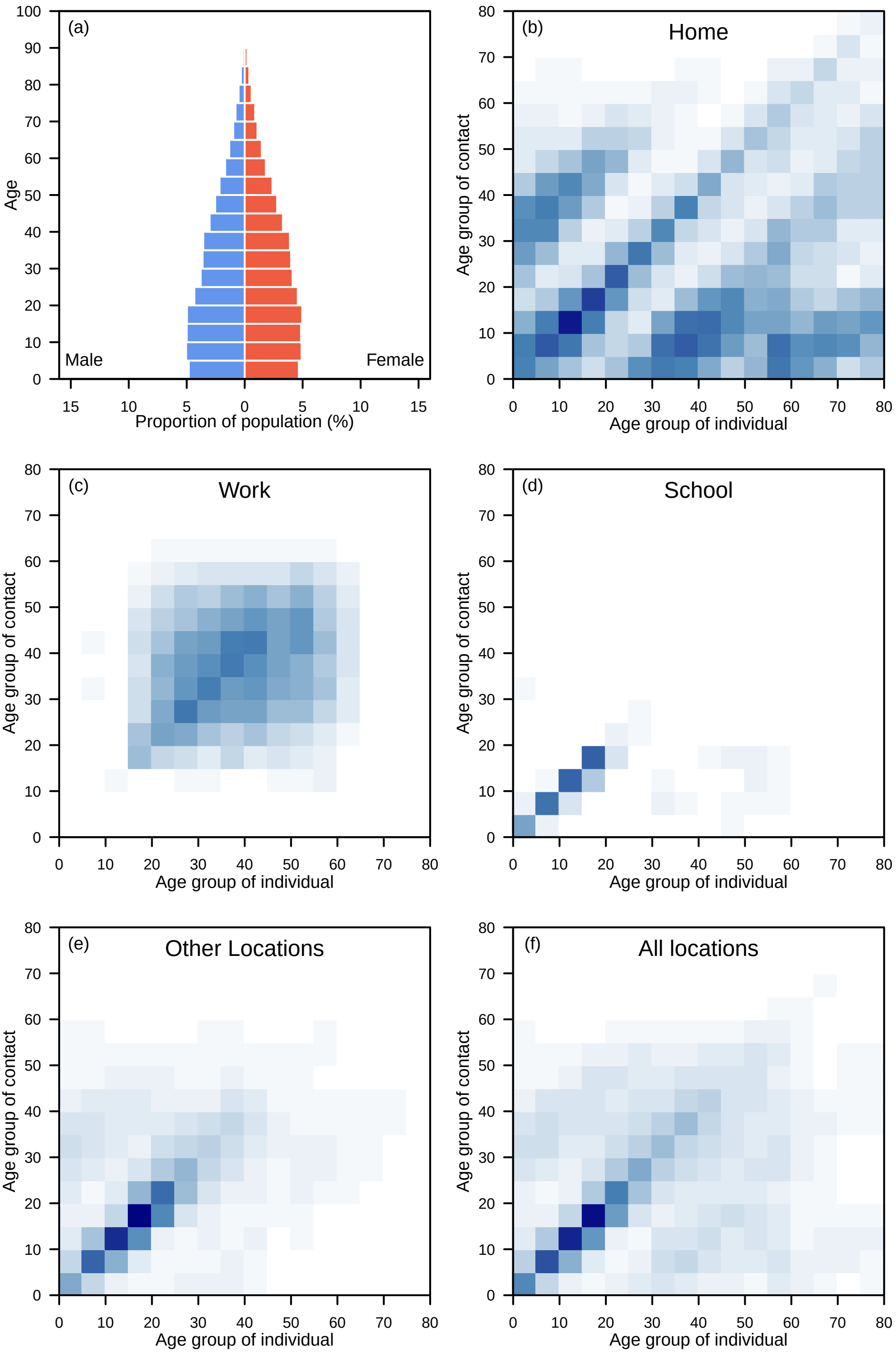
(a)
Age
Male
Female
Proportion of population (%)
(b)
Home
(c)
Work
(d)
School
(e)
Other Locations
(f)
All locations
Age group of contact
Age group of individual

Monaco

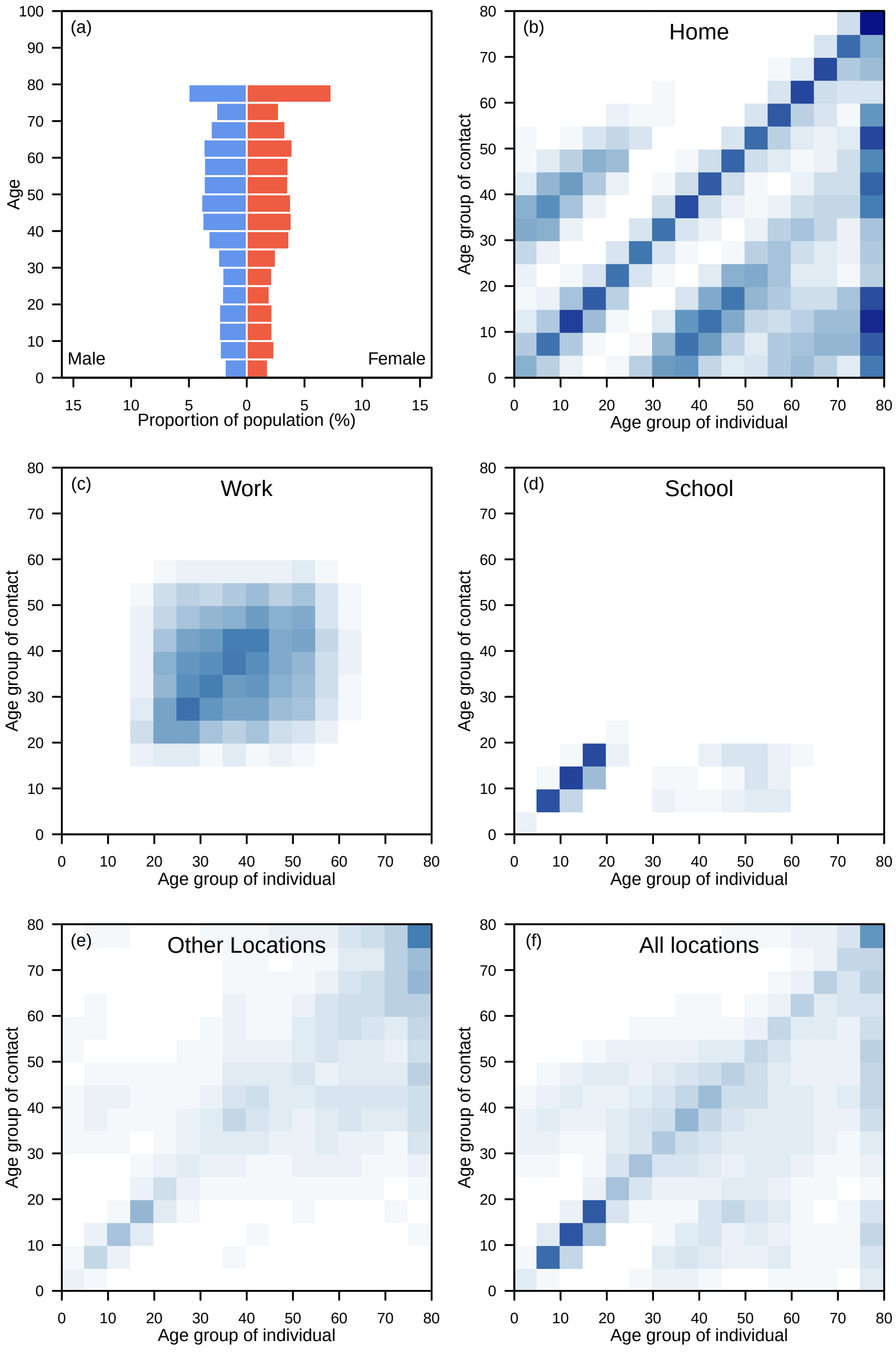

(a)
Age
Male
Female
Proportion of population (%)
(b)
Home
Age group of contact
Age group of individual
(c)
Work
Age group of contact
Age group of individual
(d)
School
Age group of contact
Age group of individual
(e)
Other Locations
Age group of contact
Age group of individual
(f)
All locations
Age group of contact
Age group of individual

Mongolia

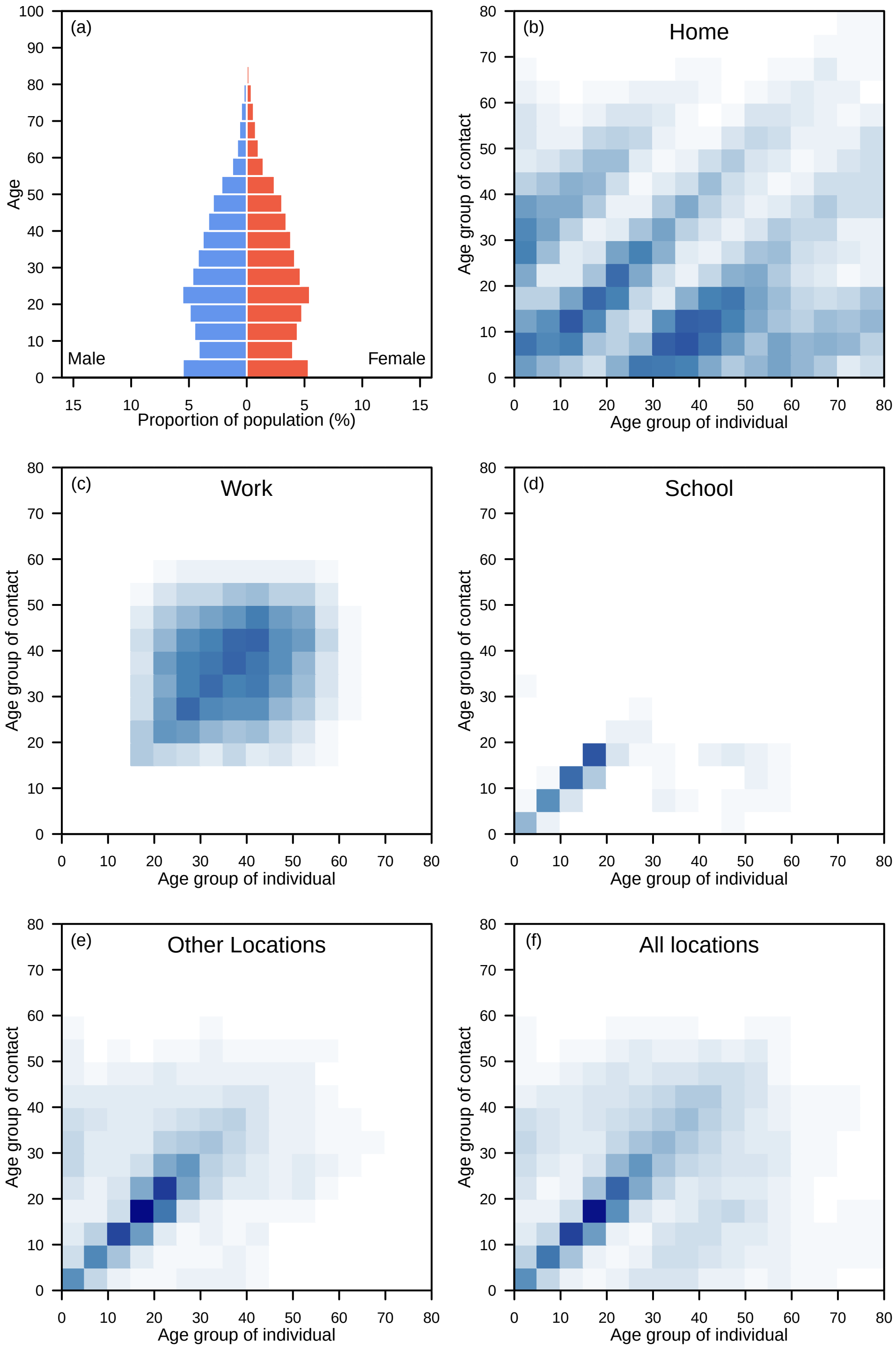

(a)
Age
Male
Female
Proportion of population (%)
(b) Home
(c) Work
(d) School
(e) Other Locations
(f) All locations
Age group of contact
Age group of individual

Montenegro

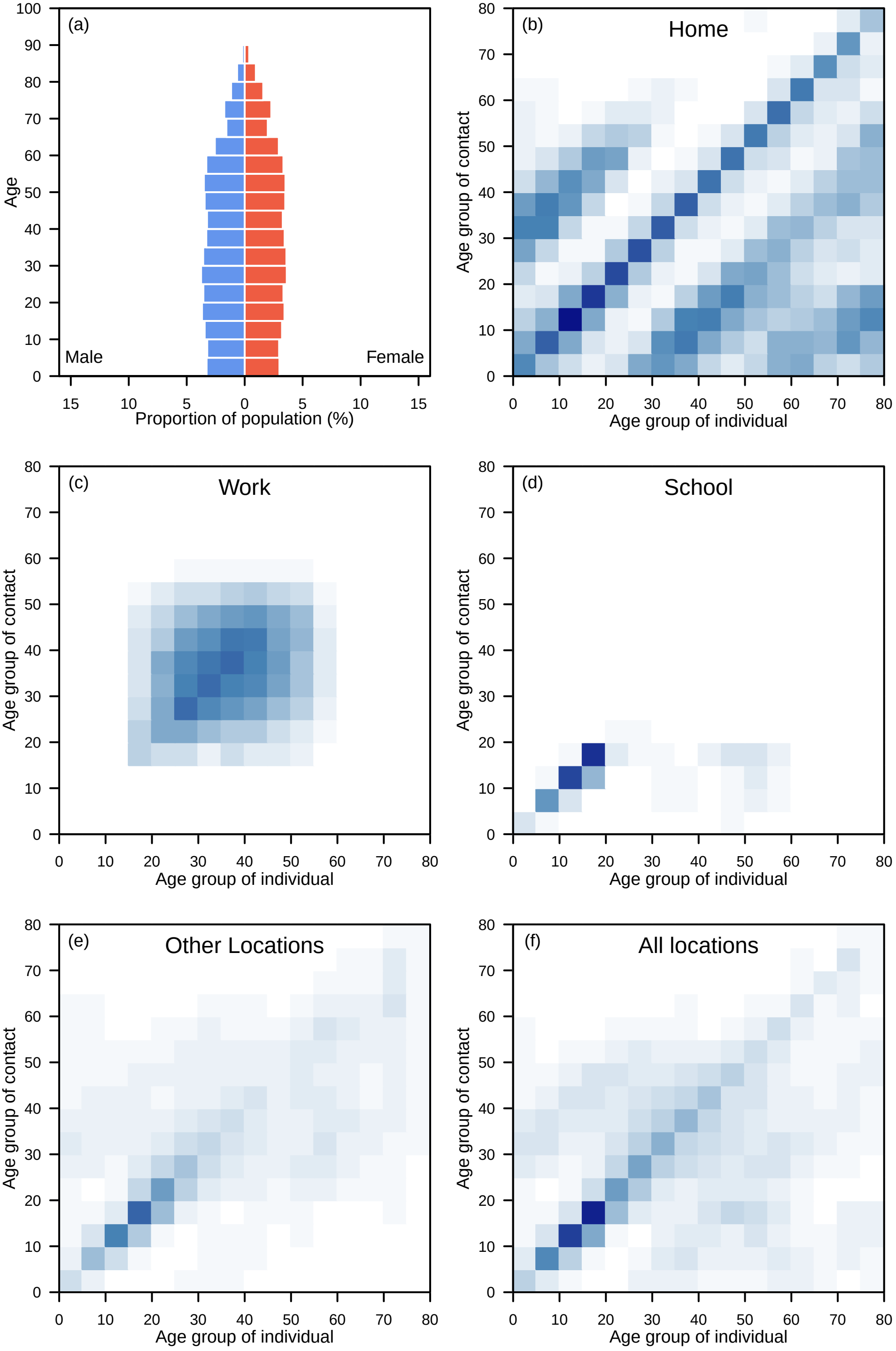

(a)
Age
Male
Female
Proportion of population (%)
(b)
Home
Age group of contact
Age group of individual
(c)
Work
Age group of contact
Age group of individual
(d)
School
Age group of contact
Age group of individual
(e)
Other Locations
Age group of contact
Age group of individual
(f)
All locations
Age group of contact
Age group of individual

Morocco

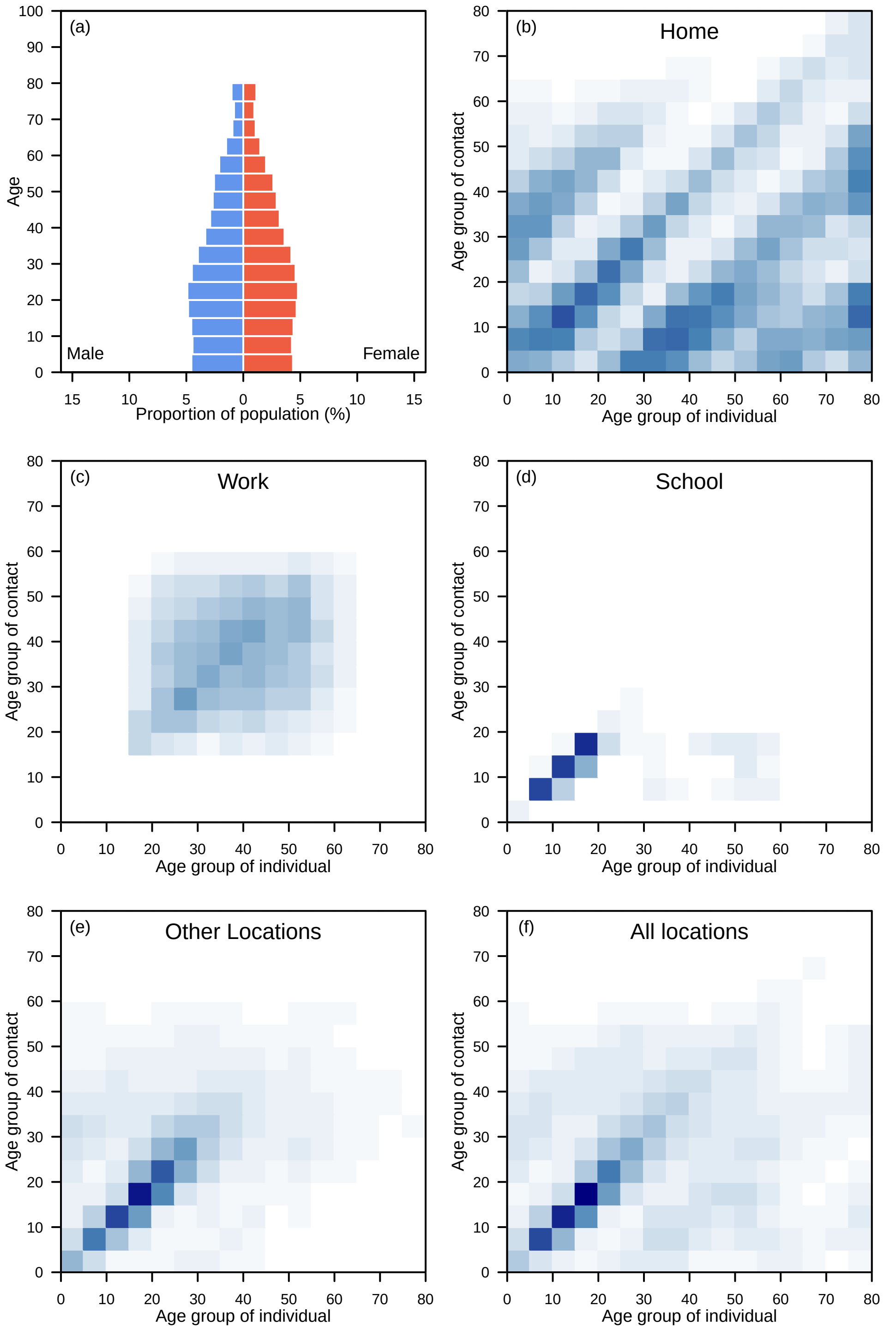
(a)
Age
Male
Female
Proportion of population (%)
(b)
Home
Age group of contact
Age group of individual
(c)
Work
Age group of contact
Age group of individual
(d)
School
Age group of contact
Age group of individual
(e)
Other Locations
Age group of contact
Age group of individual
(f)
All locations
Age group of contact
Age group of individual

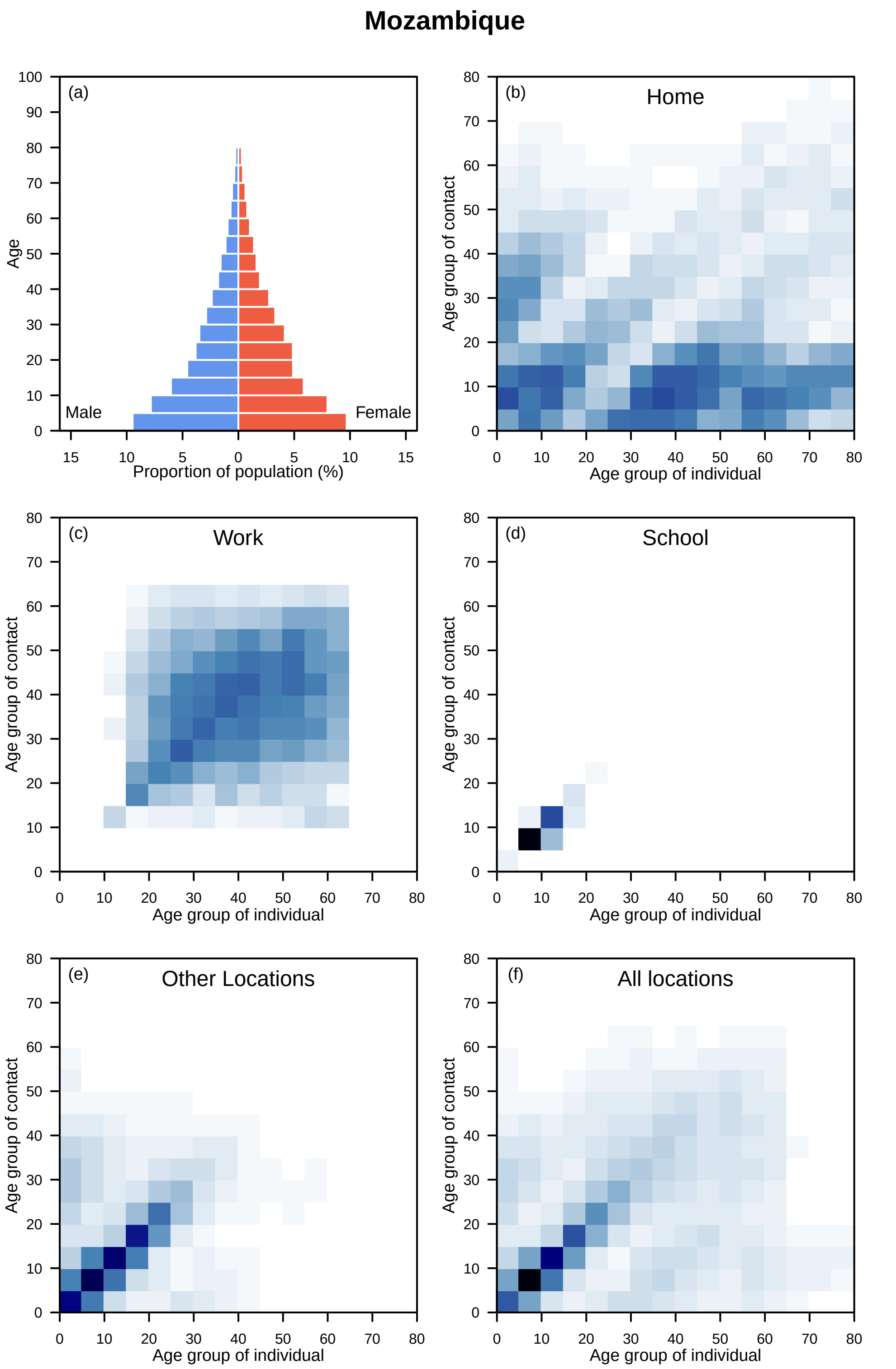

Mozambique
(a)
Male
Female
Age
Proportion of population (%)
(b)
Home
(c)
Work
(d)
School
(e)
Other Locations
(f)
All locations
Age group of contact
Age group of individual

Namibia

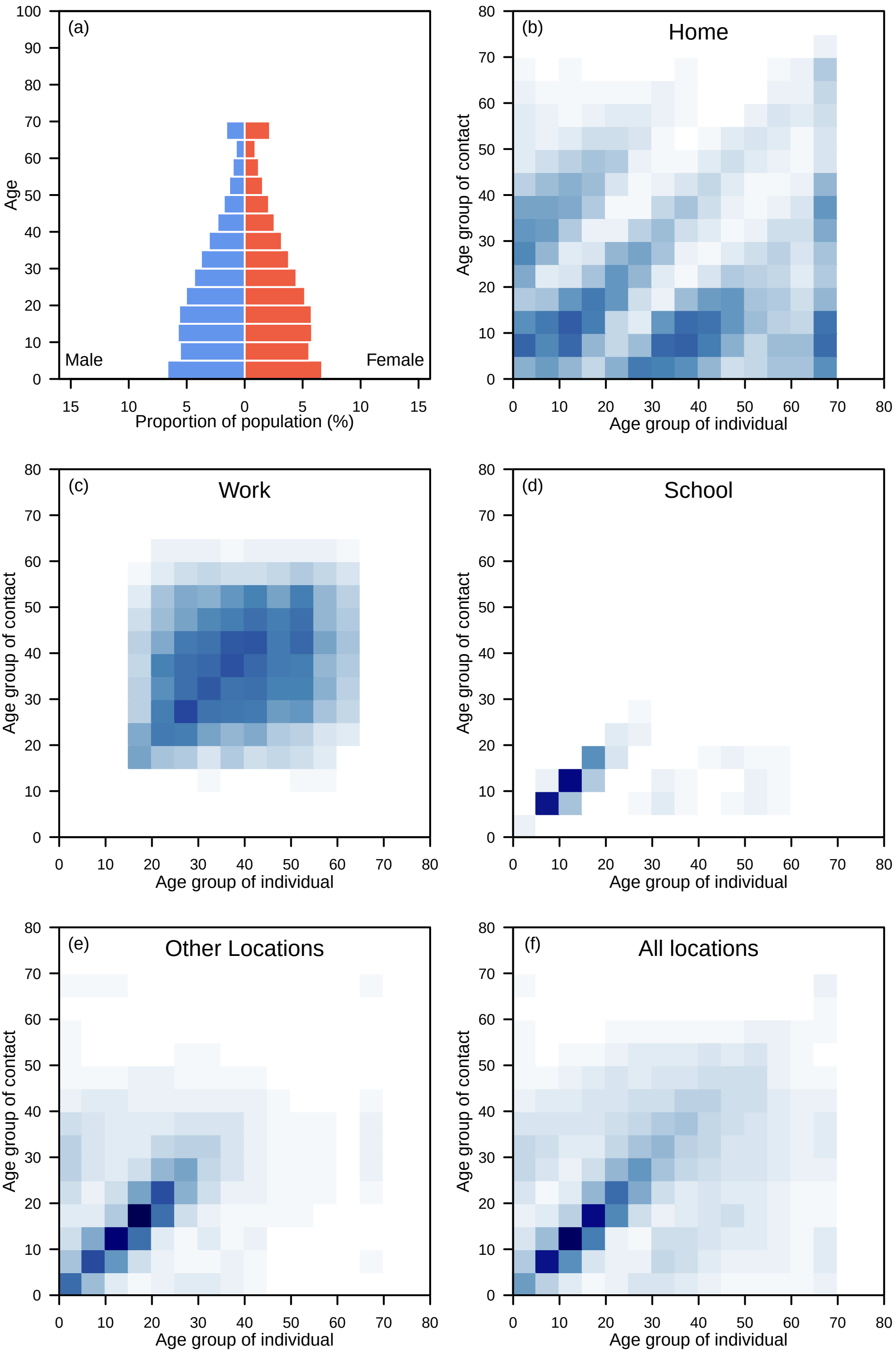

(a)
Age
Male
Female
Proportion of population (%)
(b)
Home
Age group of contact
Age group of individual
(c)
Work
Age group of contact
Age group of individual
(d)
School
Age group of contact
Age group of individual
(e)
Other Locations
Age group of contact
Age group of individual
(f)
All locations
Age group of contact
Age group of individual

Nepal

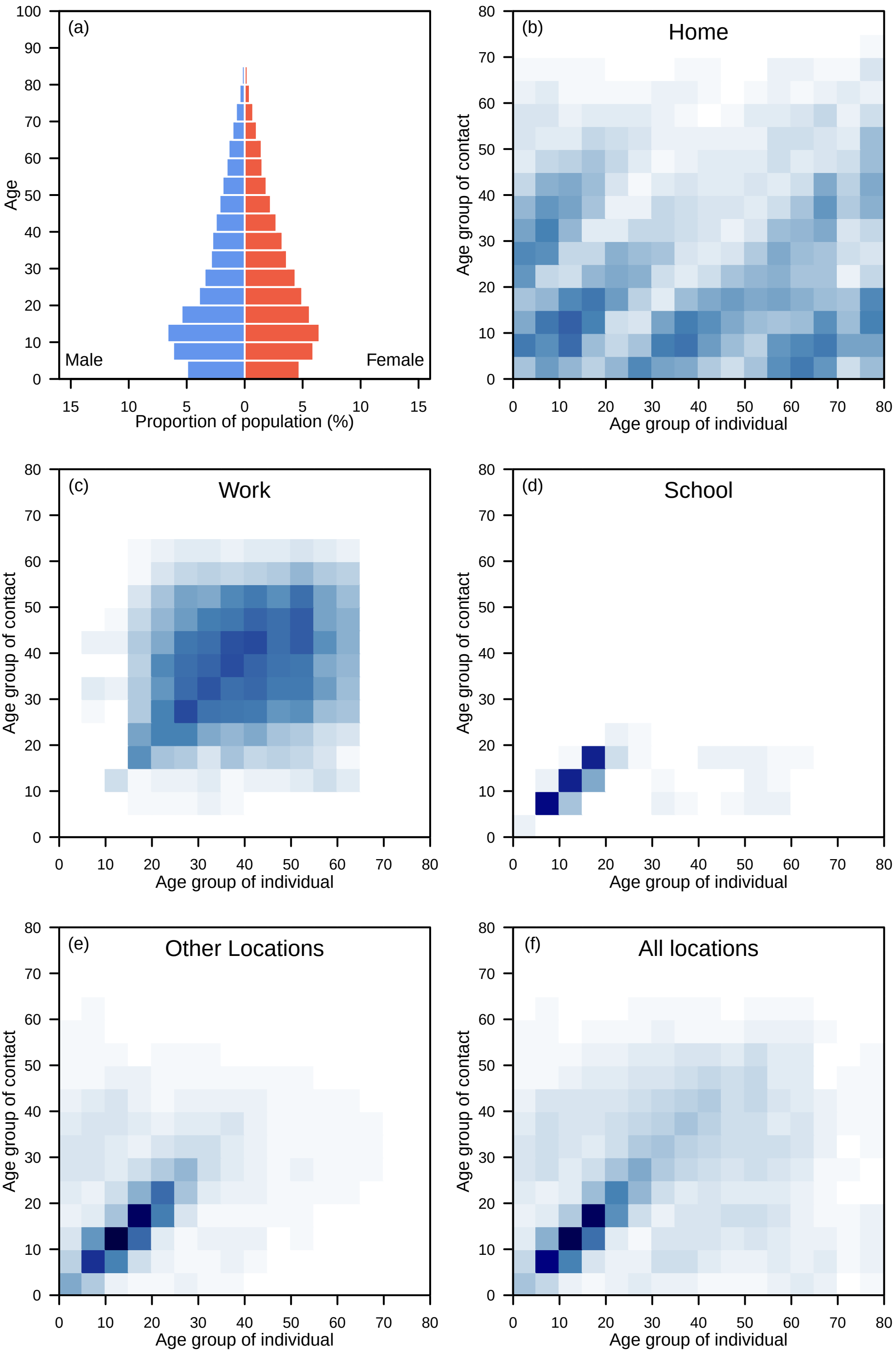

(a)
Age
Male
Female
Proportion of population (%)
(b)
Home
(c)
Work
(d)
School
(e)
Other Locations
(f)
All locations
Age group of contact
Age group of individual

Netherlands

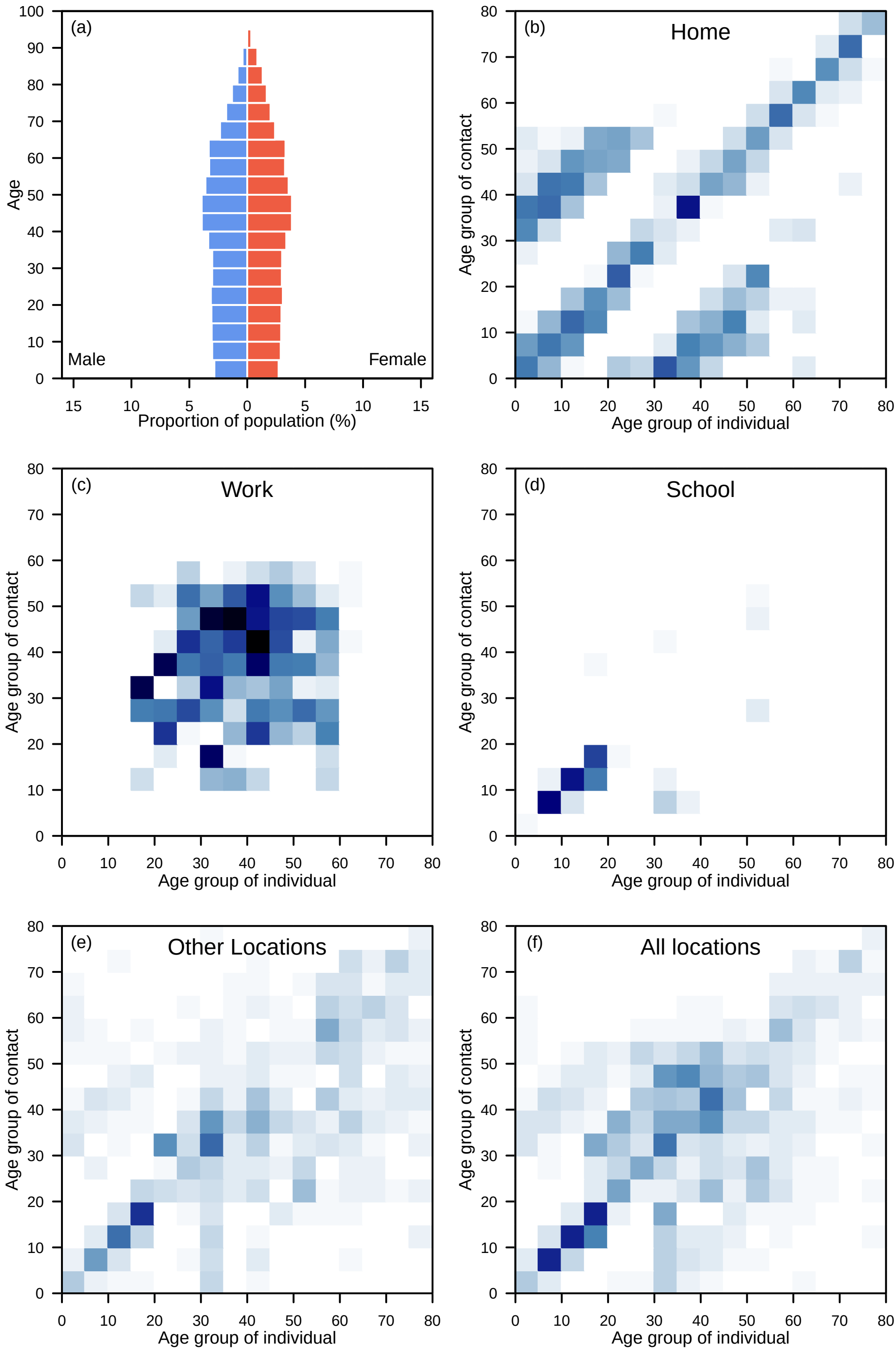

New Zealand

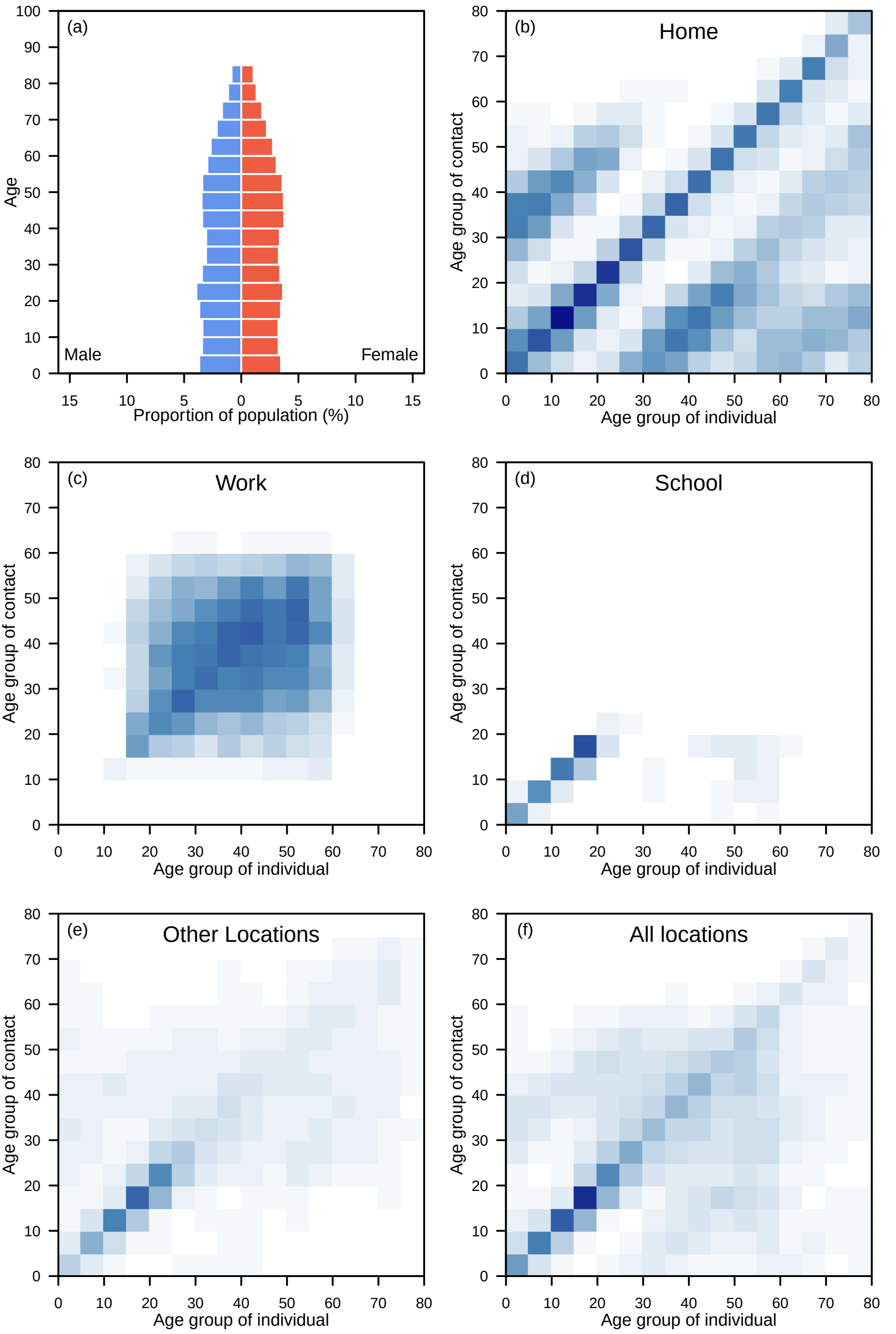
(a)
Male
Female
Age
Proportion of population (%)
(b) Home
(c) Work
(d) School
(e) Other Locations
(f) All locations
Age group of contact
Age group of individual

Nicaragua

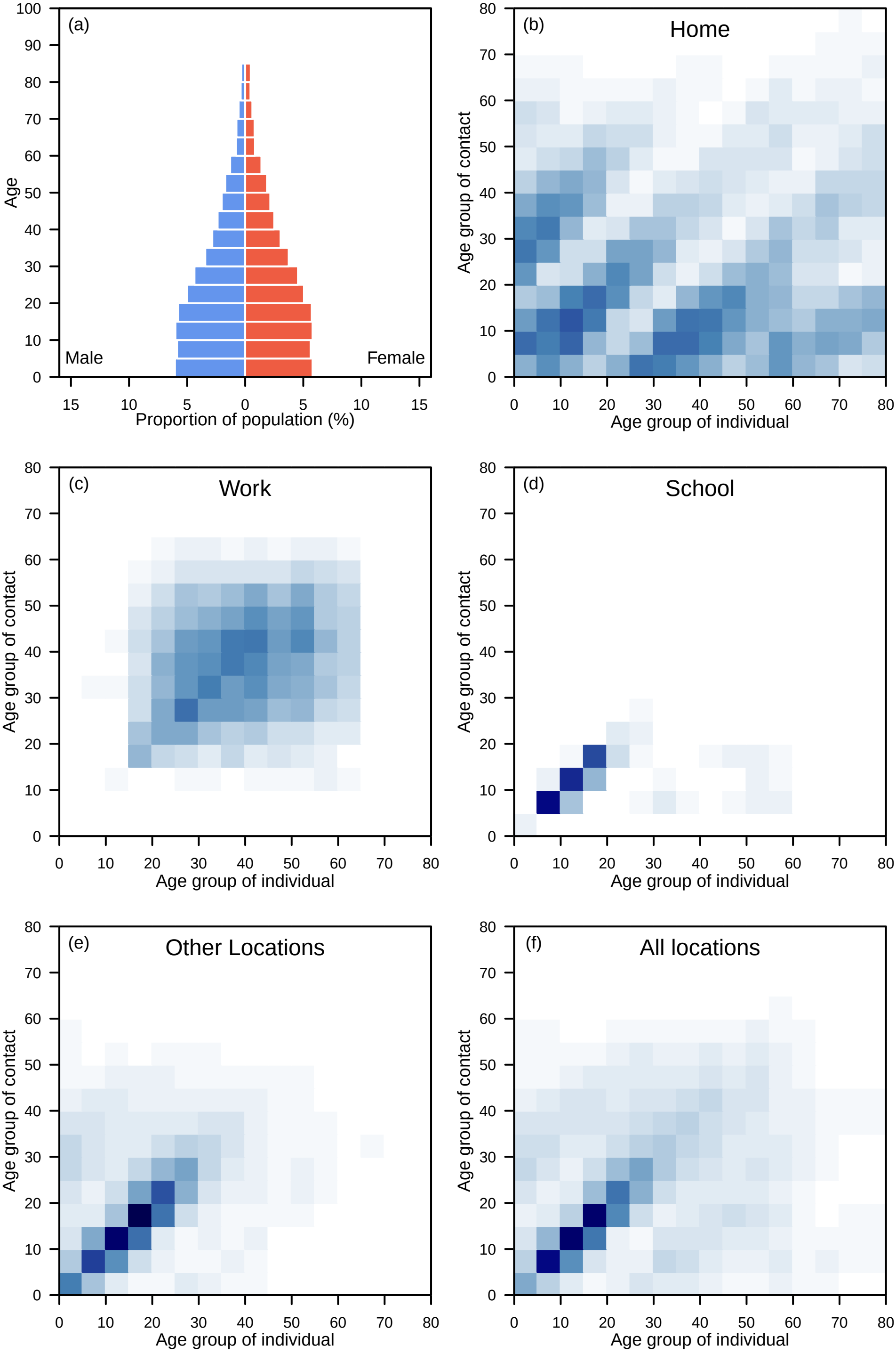

(a)
Age
Male
Female
Proportion of population (%)
(b) Home
(c) Work
(d) School
(e) Other Locations
(f) All locations
Age group of contact
Age group of individual

Niger

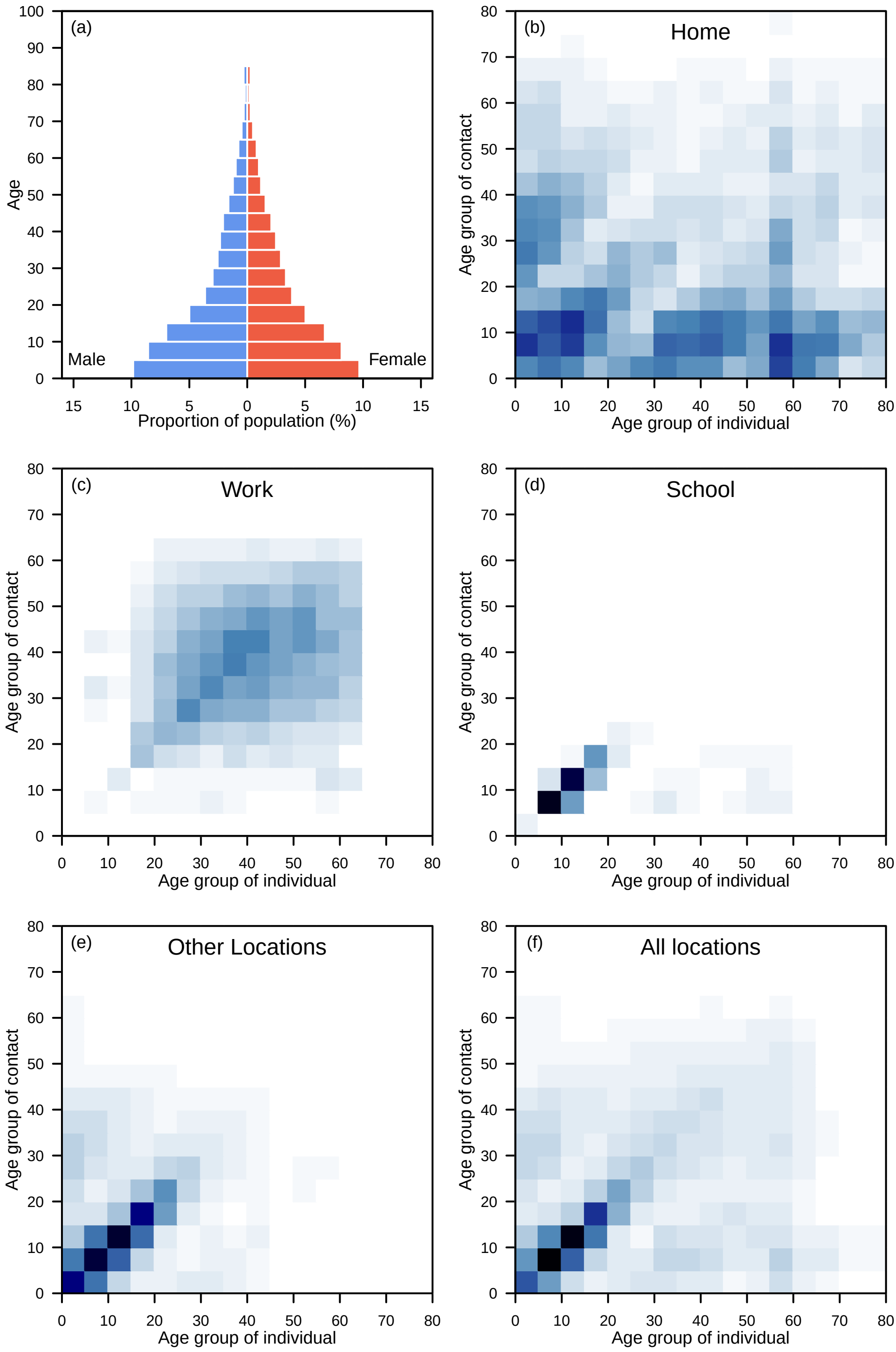

(a)
Male
Female
Age
Proportion of population (%)
(b)
Home
(c)
Work
(d)
School
(e)
Other Locations
(f)
All locations
Age group of contact
Age group of individual

Nigeria

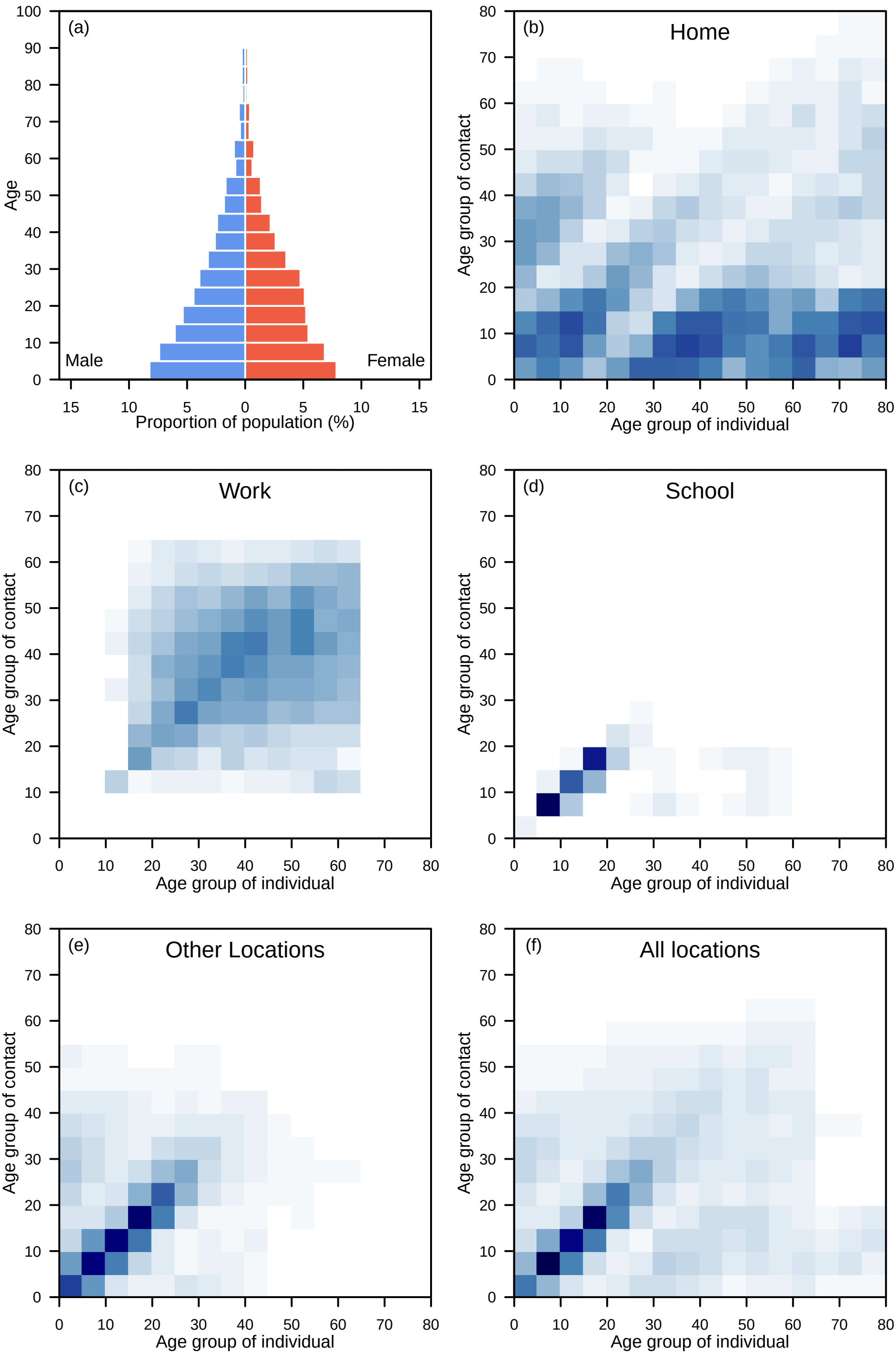
(a)
Age
Male
Female
Proportion of population (%)
(b)
Home
Age group of contact
Age group of individual
(c)
Work
(d)
School
(e)
Other Locations
(f)
All locations

Oman

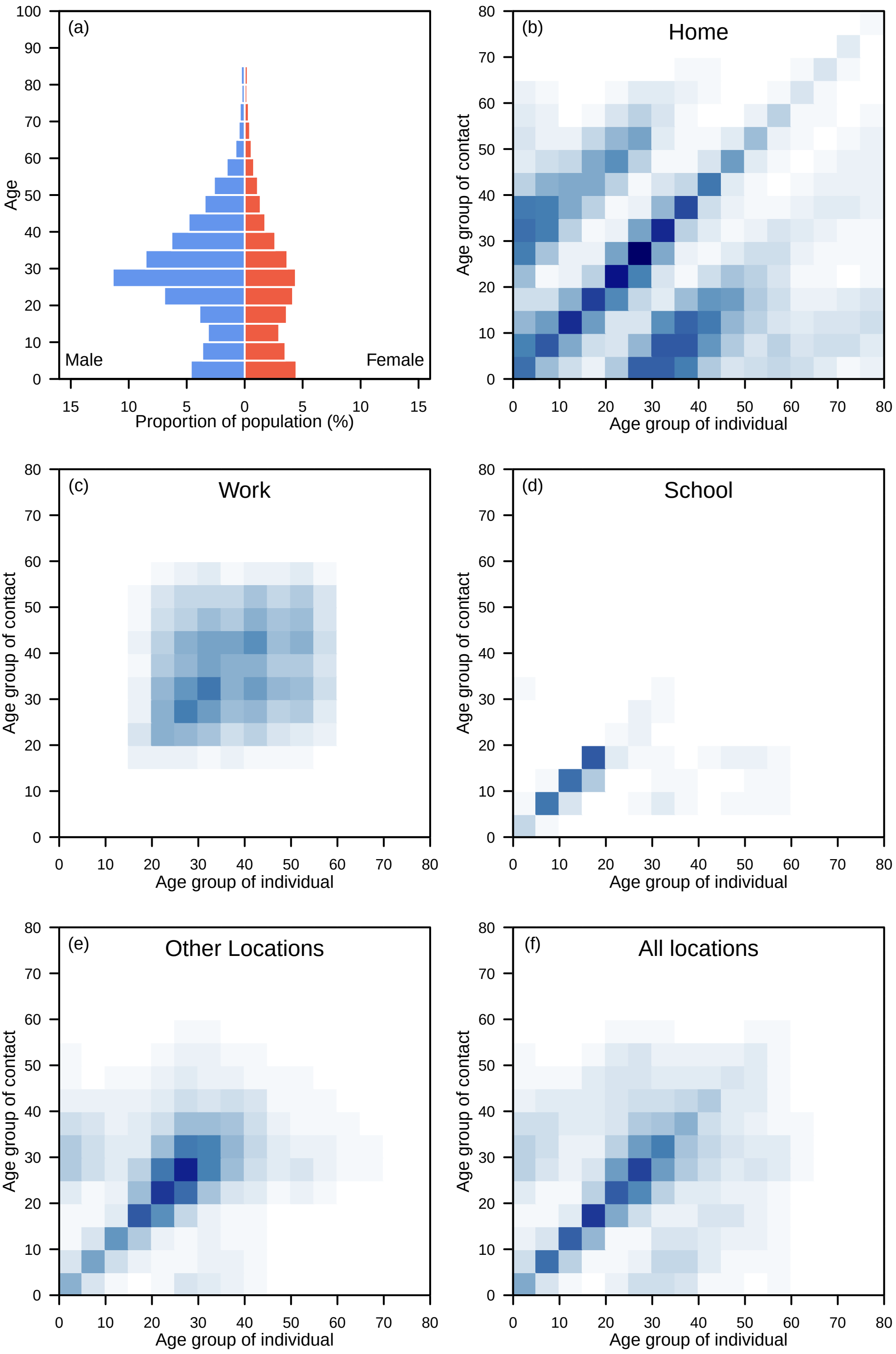

(a)
Age
Male
Female
Proportion of population (%)
(b)
Home
Age group of contact
Age group of individual
(c)
Work
Age group of contact
Age group of individual
(d)
School
Age group of contact
Age group of individual
(e)
Other Locations
Age group of contact
Age group of individual
(f)
All locations
Age group of contact
Age group of individual

Pakistan

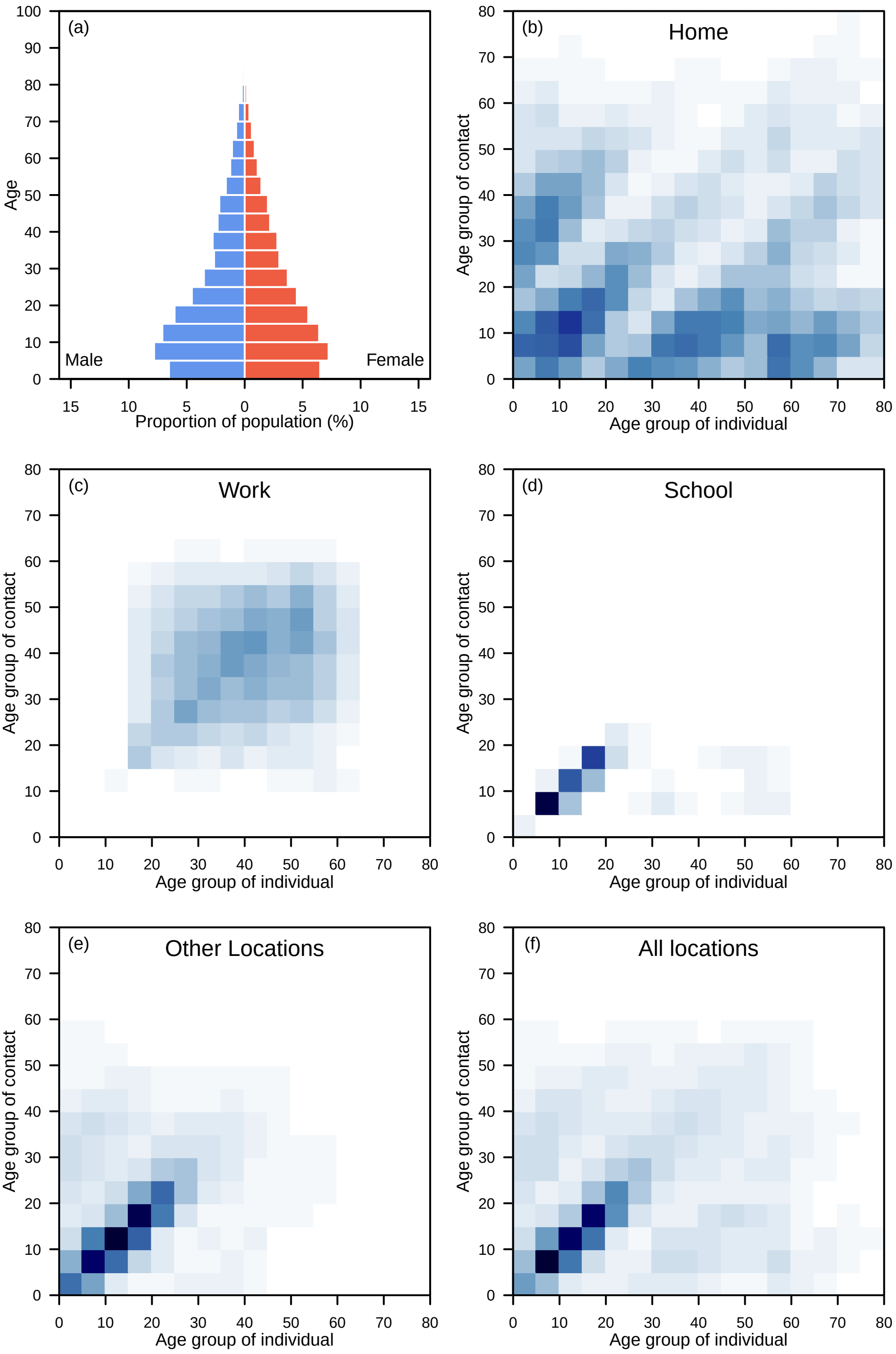
(a)
Age
Male
Female
Proportion of population (%)
(b)
Home
(c)
Work
(d)
School
(e)
Other Locations
(f)
All locations
Age group of contact
Age group of individual

Panama

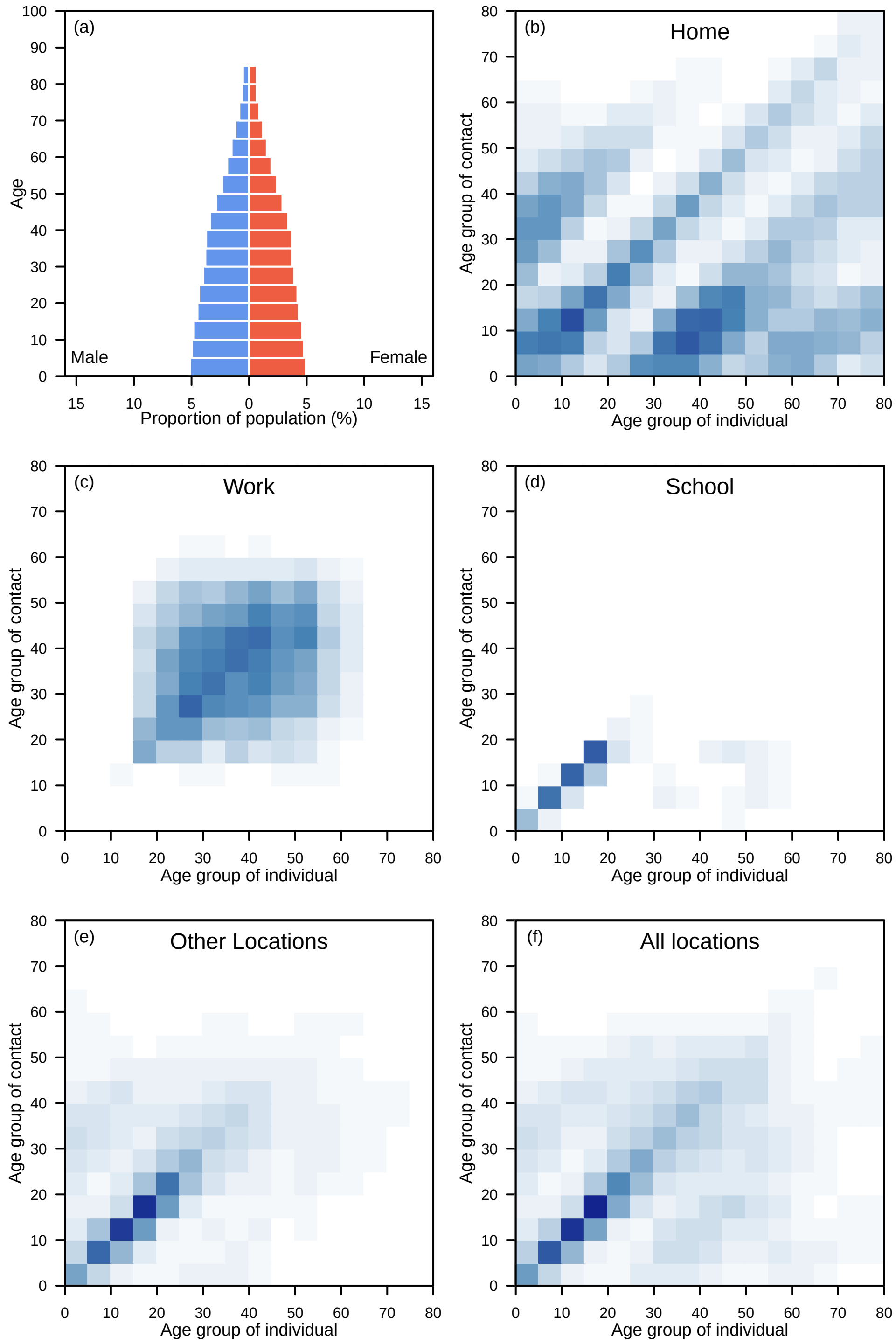

(a)
Age
Male
Female
Proportion of population (%)
(b)
Home
Age group of contact
Age group of individual
(c)
Work
Age group of contact
Age group of individual
(d)
School
Age group of contact
Age group of individual
(e)
Other Locations
Age group of contact
Age group of individual
(f)
All locations
Age group of contact
Age group of individual

Paraguay

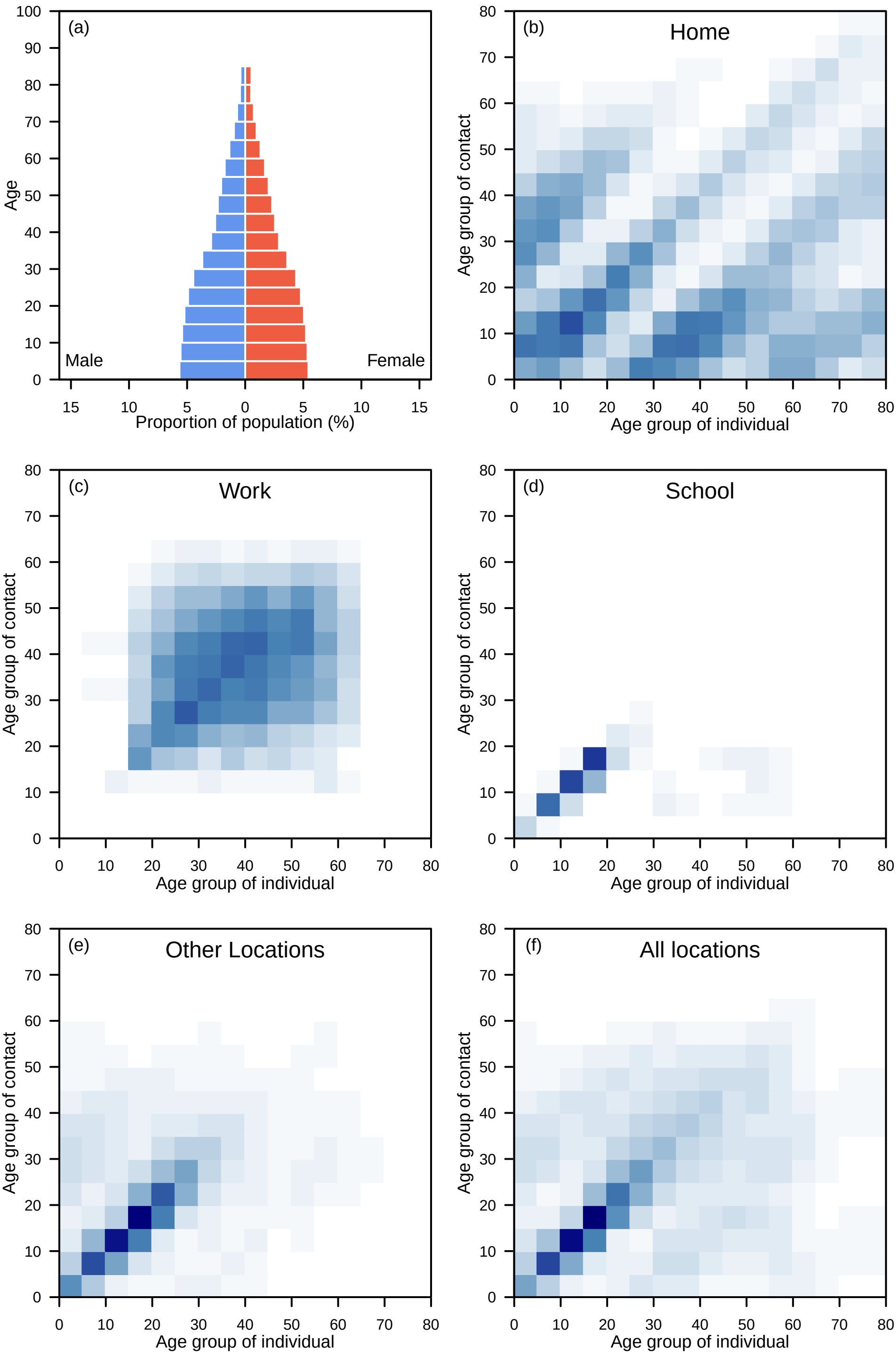

(a)
Age
Male
Female
Proportion of population (%)
(b)
Home
Age group of contact
Age group of individual
(c)
Work
Age group of contact
Age group of individual
(d)
School
Age group of contact
Age group of individual
(e)
Other Locations
Age group of contact
Age group of individual
(f)
All locations
Age group of contact
Age group of individual

Peru

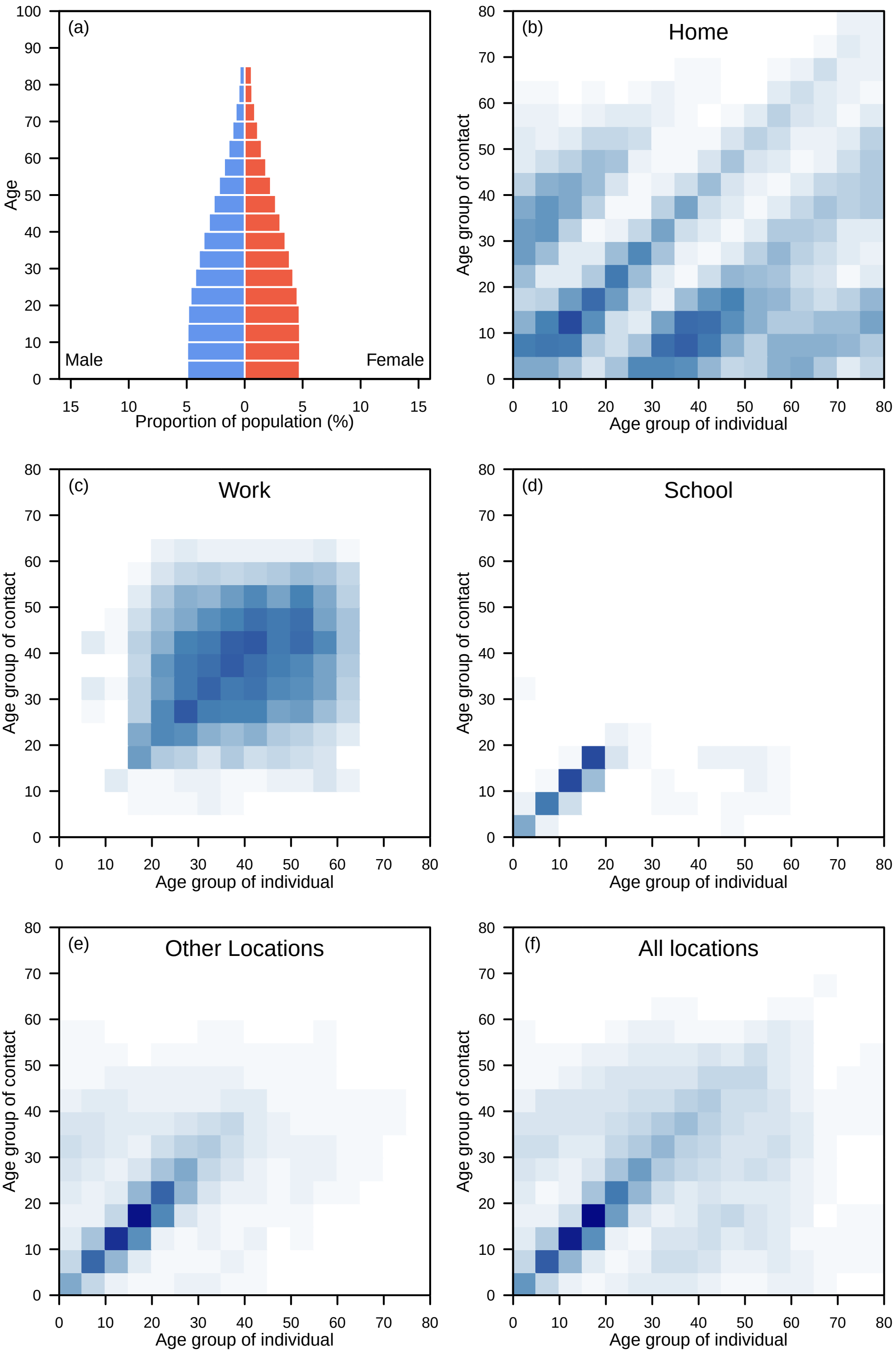

(a)
Age
Male
Female
Proportion of population (%)
(b)
Home
Age group of contact
Age group of individual
(c)
Work
Age group of contact
Age group of individual
(d)
School
Age group of contact
Age group of individual
(e)
Other Locations
Age group of contact
Age group of individual
(f)
All locations
Age group of contact
Age group of individual

Philippines

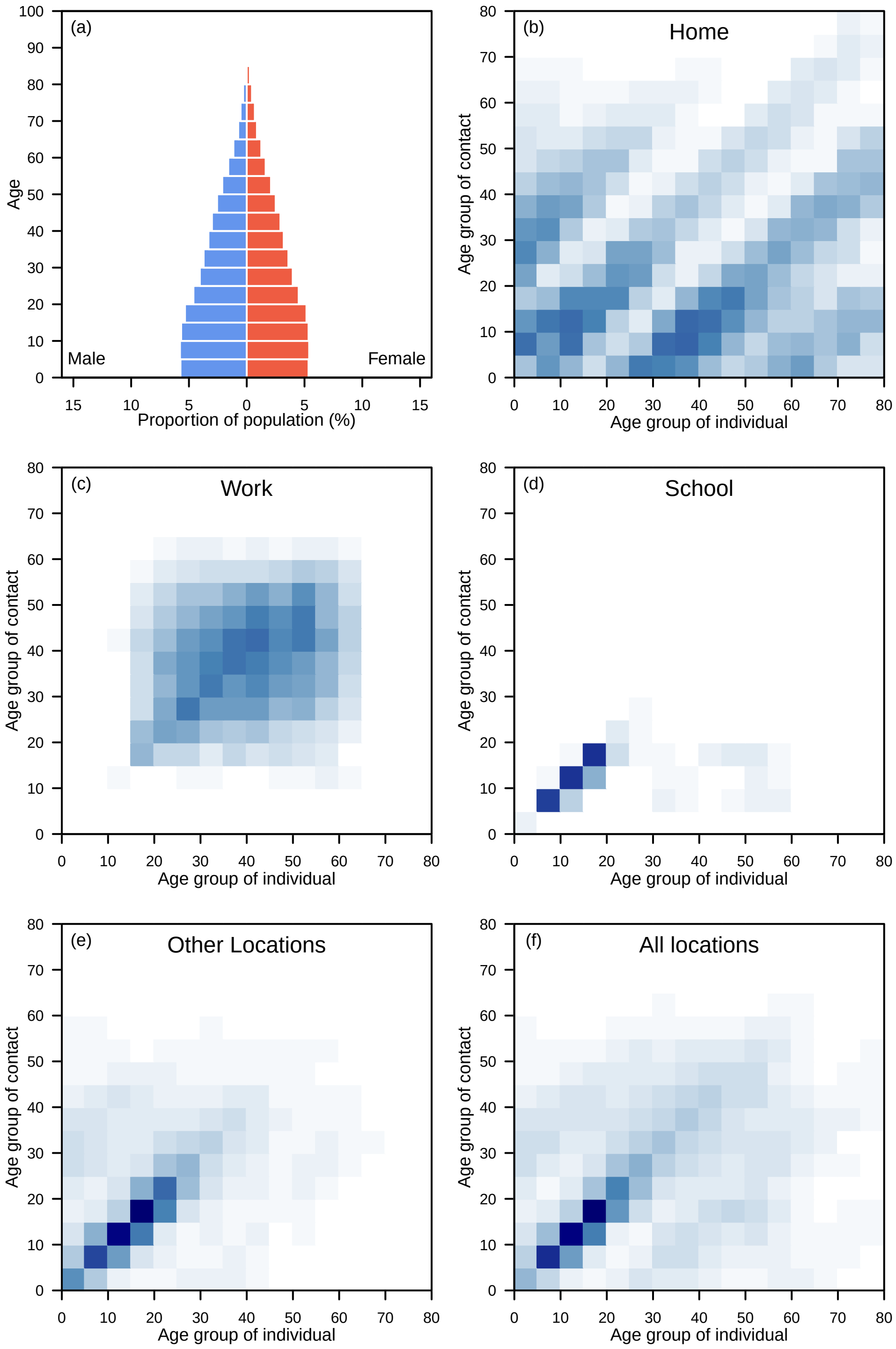

Poland

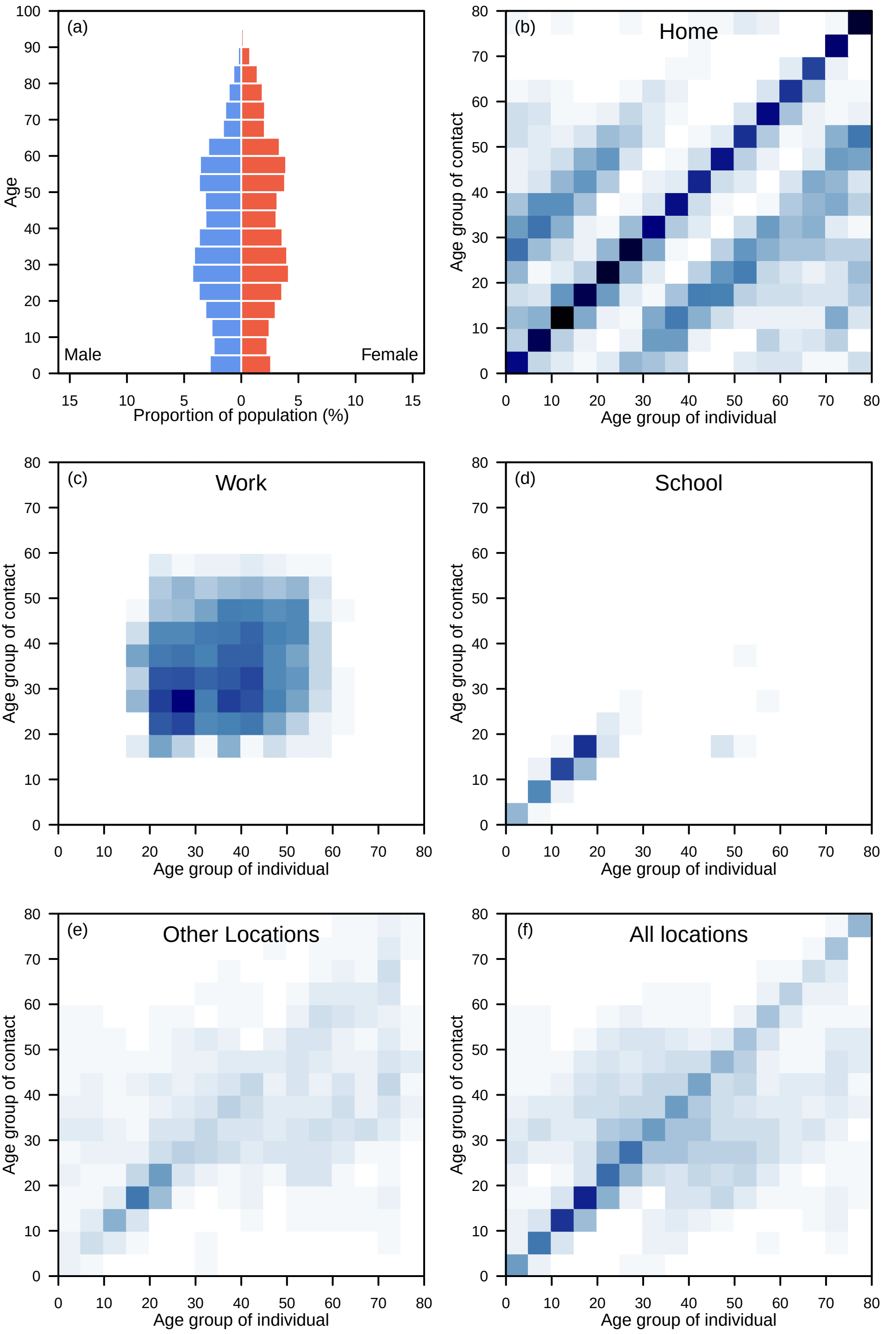

(a)
Age
Male
Female
Proportion of population (%)
(b)
Home
Age group of contact
Age group of individual
(c)
Work
(d)
School
(e)
Other Locations
(f)
All locations

Portugal

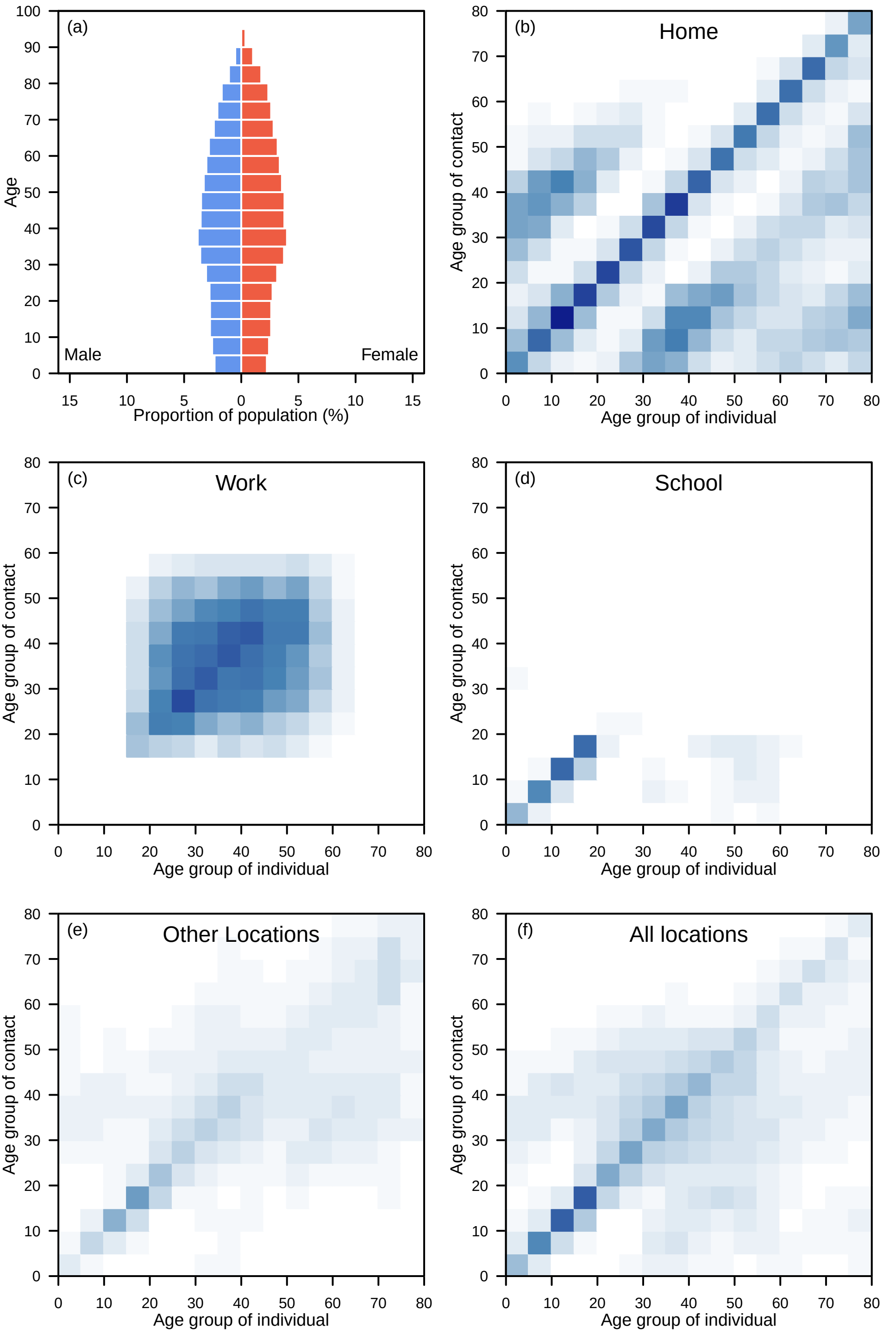

(a)
Age
Male
Female
Proportion of population (%)
(b)
Home
Age group of contact
Age group of individual
(c)
Work
(d)
School
(e)
Other Locations
(f)
All locations

Qatar

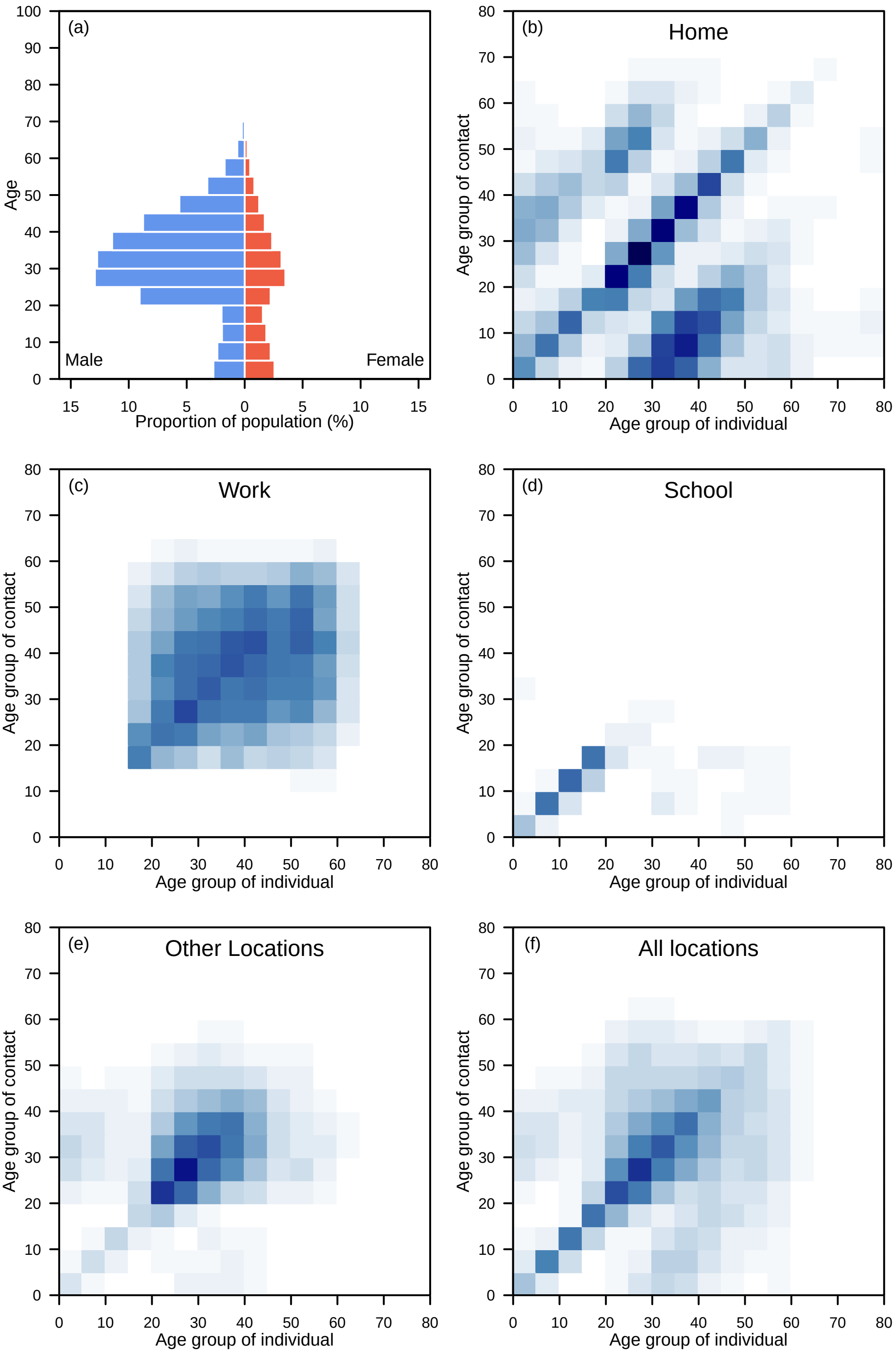
(a)
Age
Male
Female
Proportion of population (%)
(b)
Home
(c)
Work
(d)
School
(e)
Other Locations
(f)
All locations
Age group of contact
Age group of individual

Republic of Korea

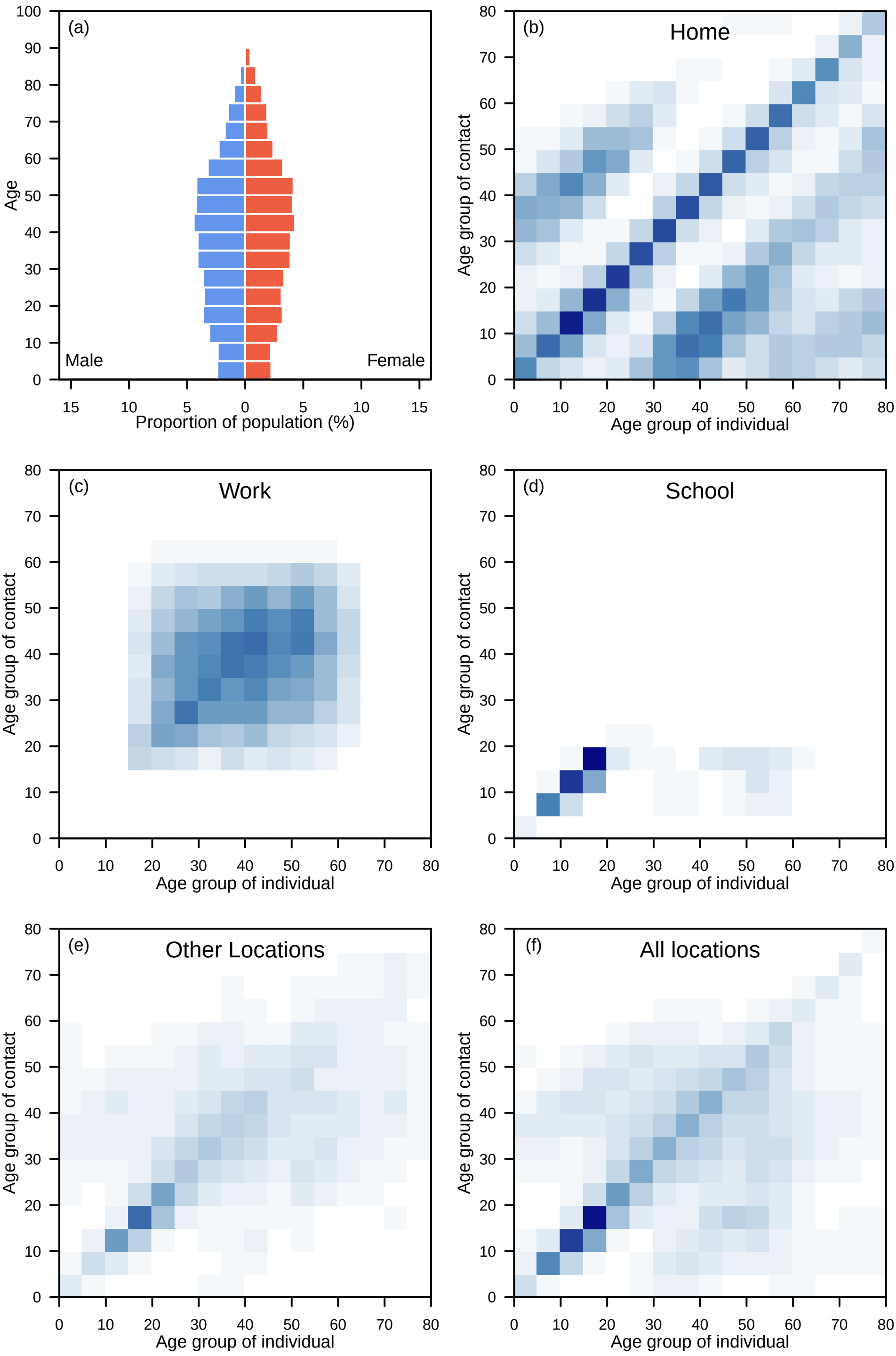

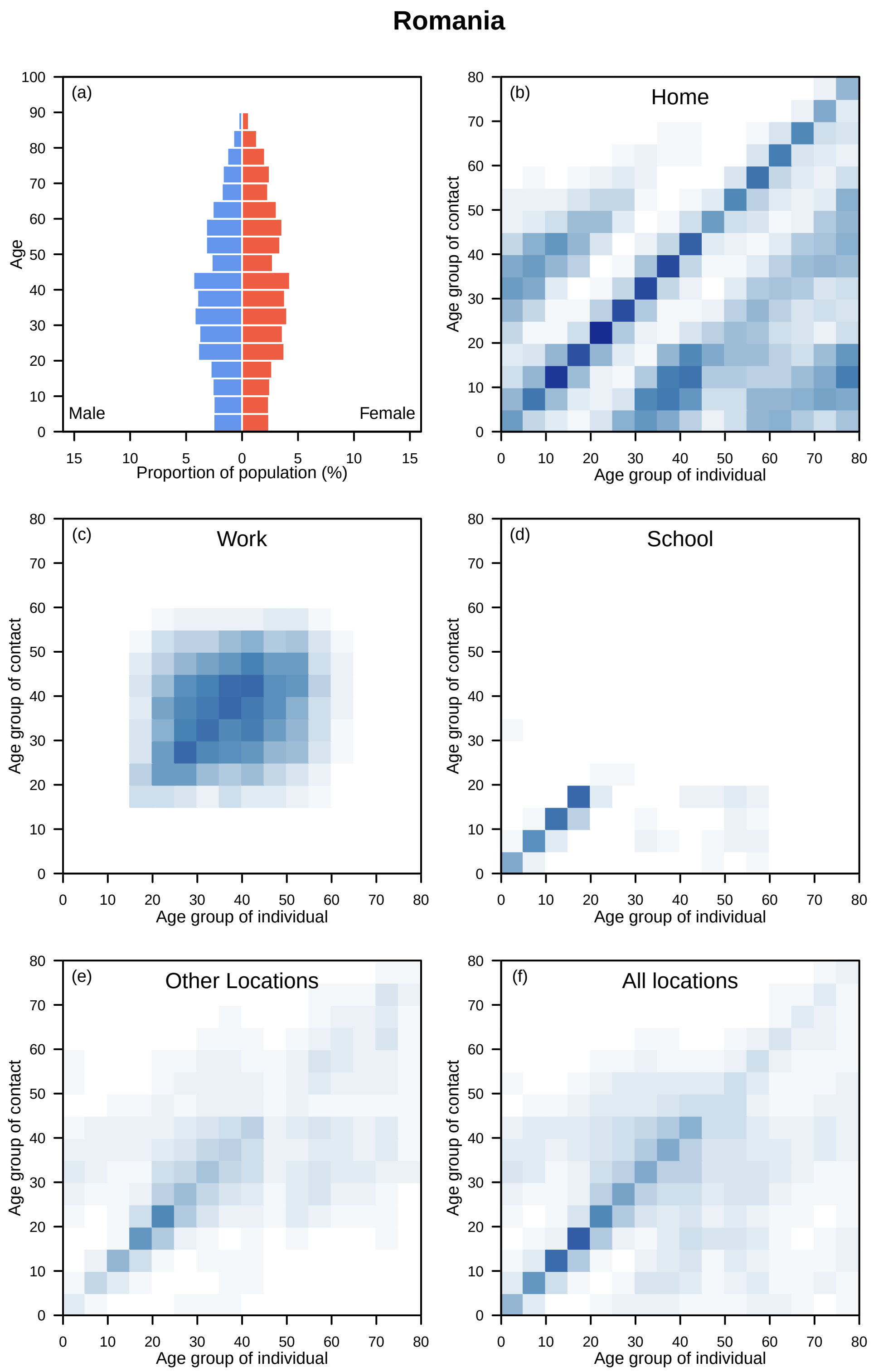
Romania
(a)
Male
Female
Age
Proportion of population (%)
(b)
Home
(c)
Work
(d)
School
(e)
Other Locations
(f)
All locations
Age group of contact
Age group of individual

Russian Federation

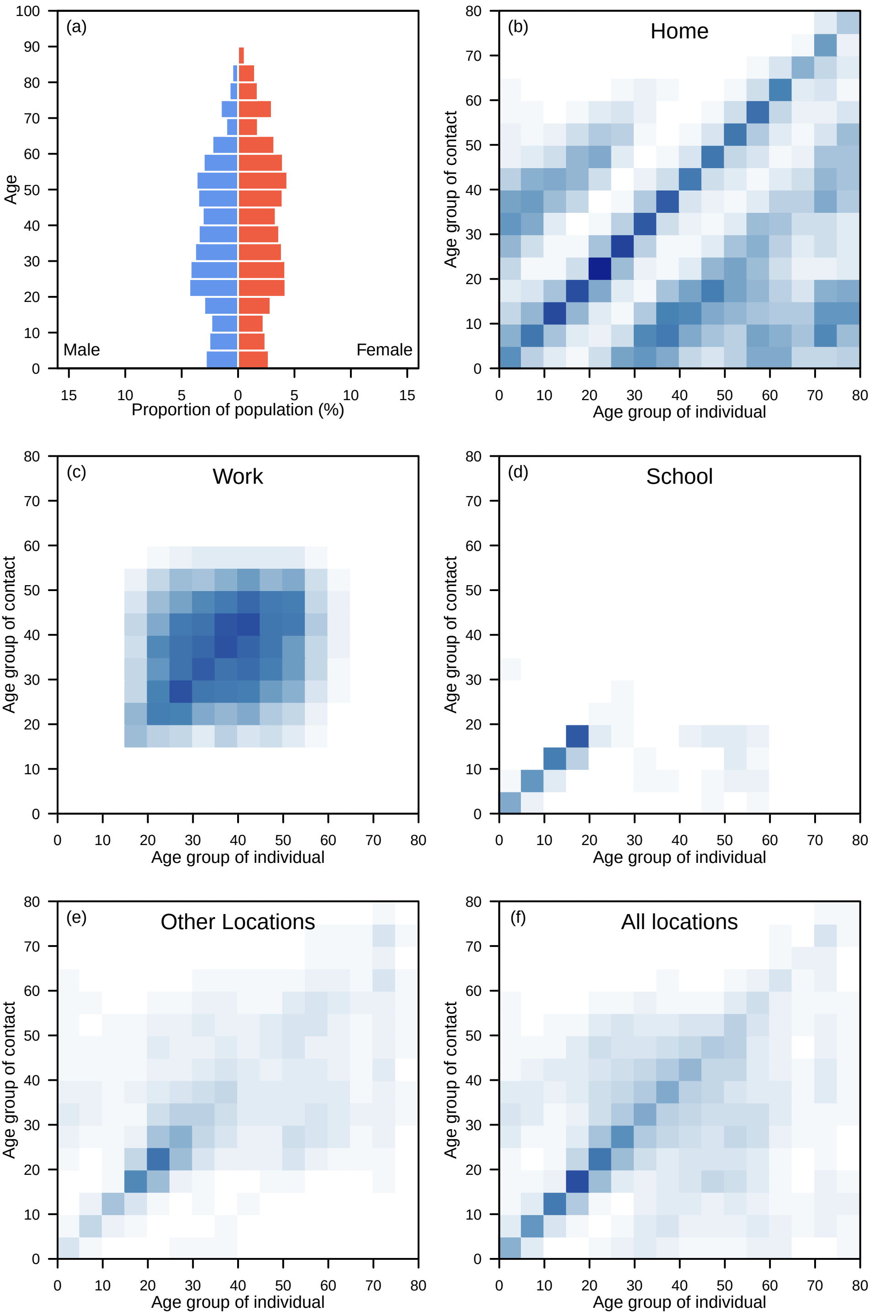

Rwanda

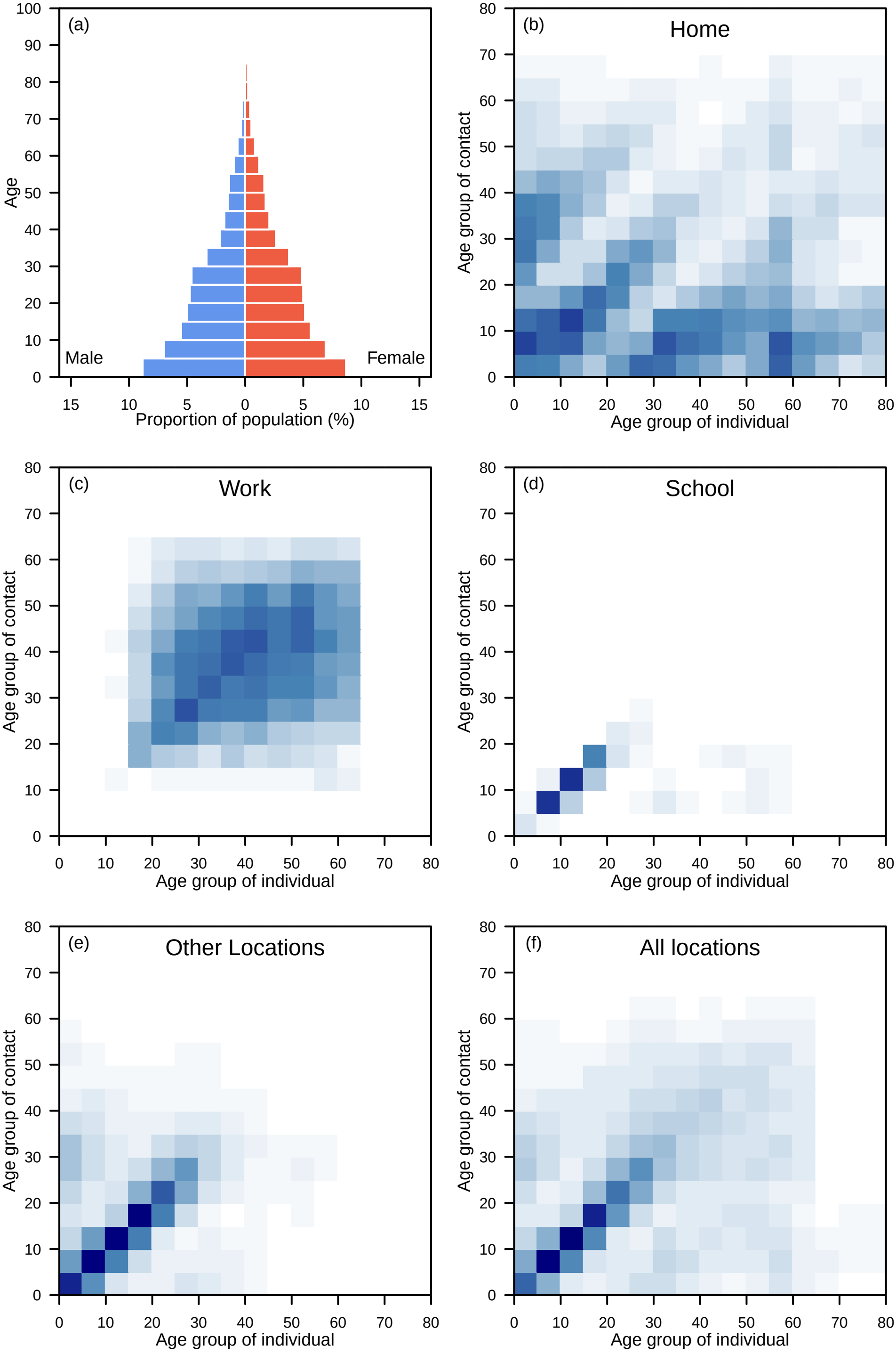

(a)
Age
Male
Female
Proportion of population (%)
(b)
Home
Age group of contact
Age group of individual
(c)
Work
Age group of contact
Age group of individual
(d)
School
Age group of contact
Age group of individual
(e)
Other Locations
Age group of contact
Age group of individual
(f)
All locations
Age group of contact
Age group of individual

Saint Lucia

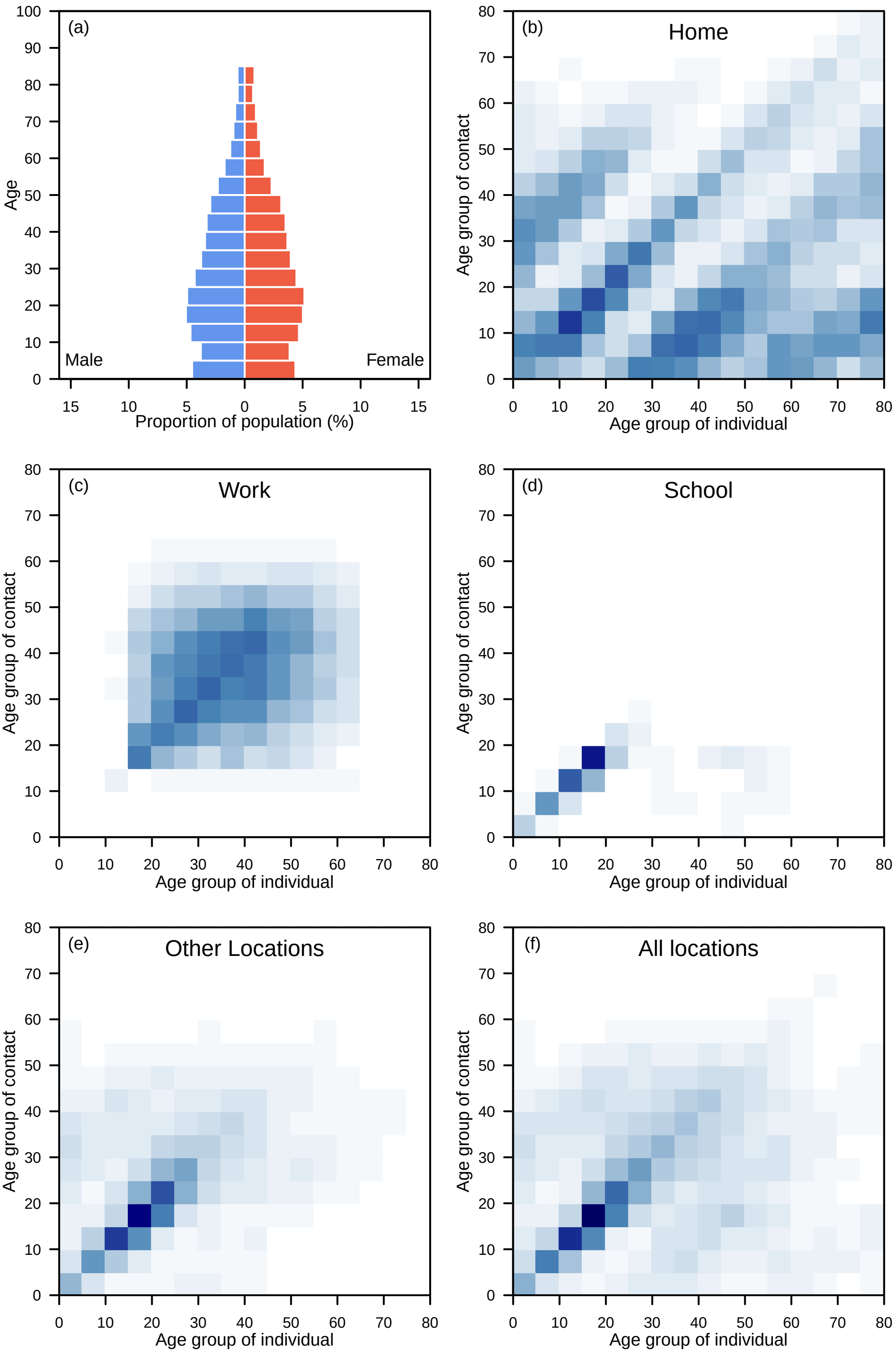

(a)
Age
Male
Female
Proportion of population (%)
(b)
Home
(c)
Work
(d)
School
(e)
Other Locations
(f)
All locations
Age group of contact
Age group of individual

Samoa

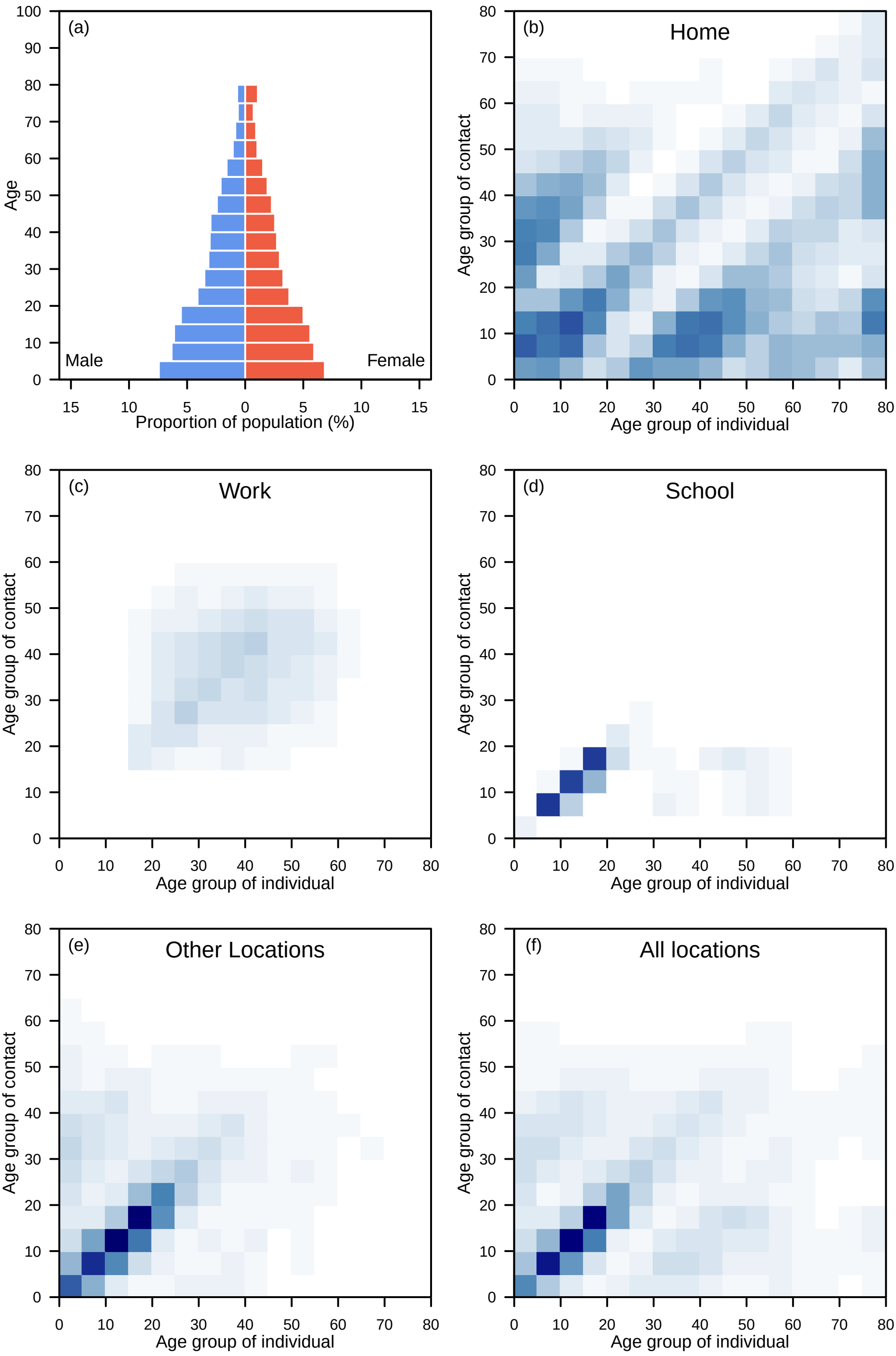

(a)
Age
Male
Female
Proportion of population (%)
(b)
Home
Age group of contact
Age group of individual
(c)
Work
Age group of contact
Age group of individual
(d)
School
Age group of contact
Age group of individual
(e)
Other Locations
Age group of contact
Age group of individual
(f)
All locations
Age group of contact
Age group of individual

Sao Tome and Principe

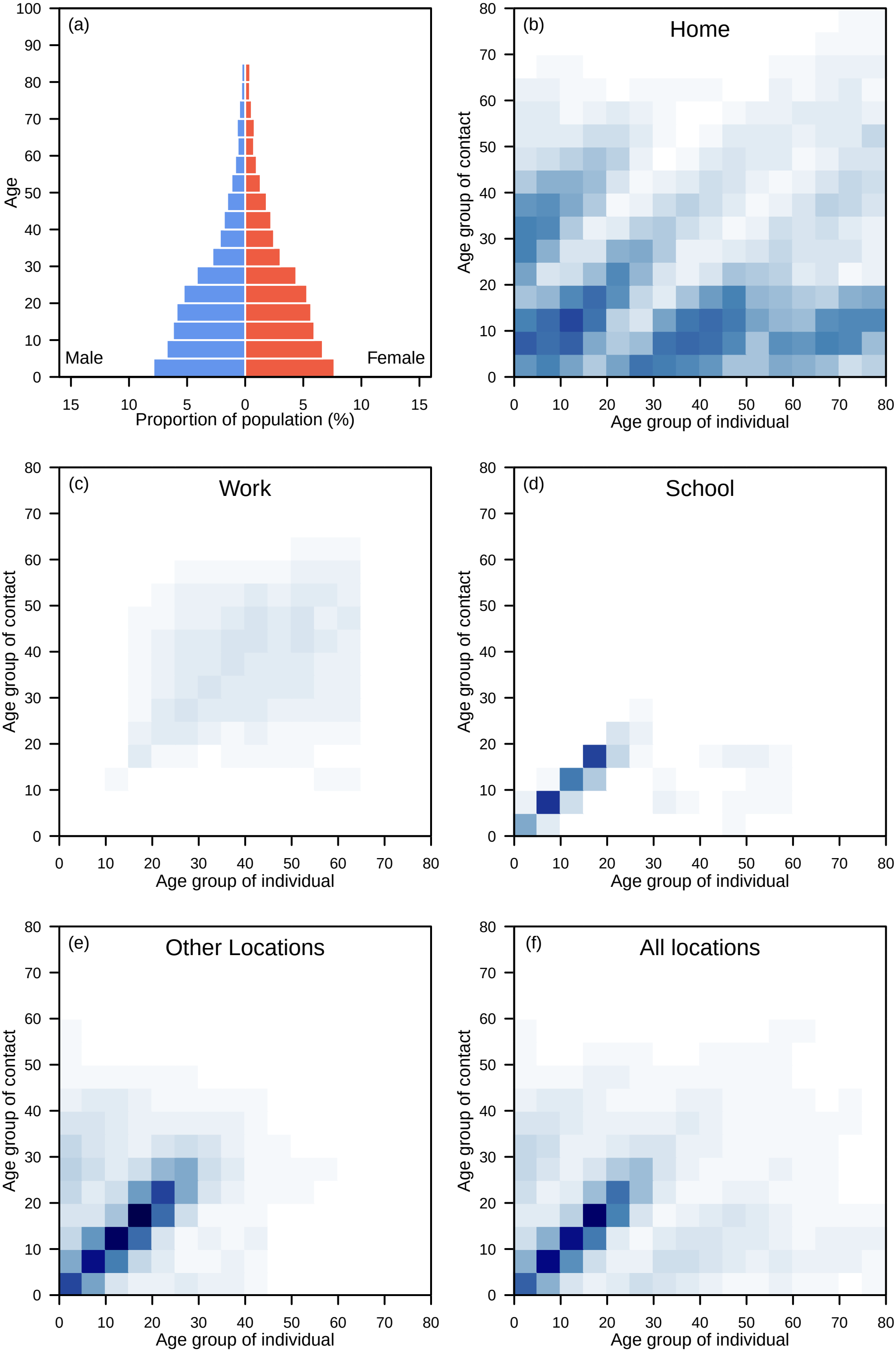

Saudi Arabia

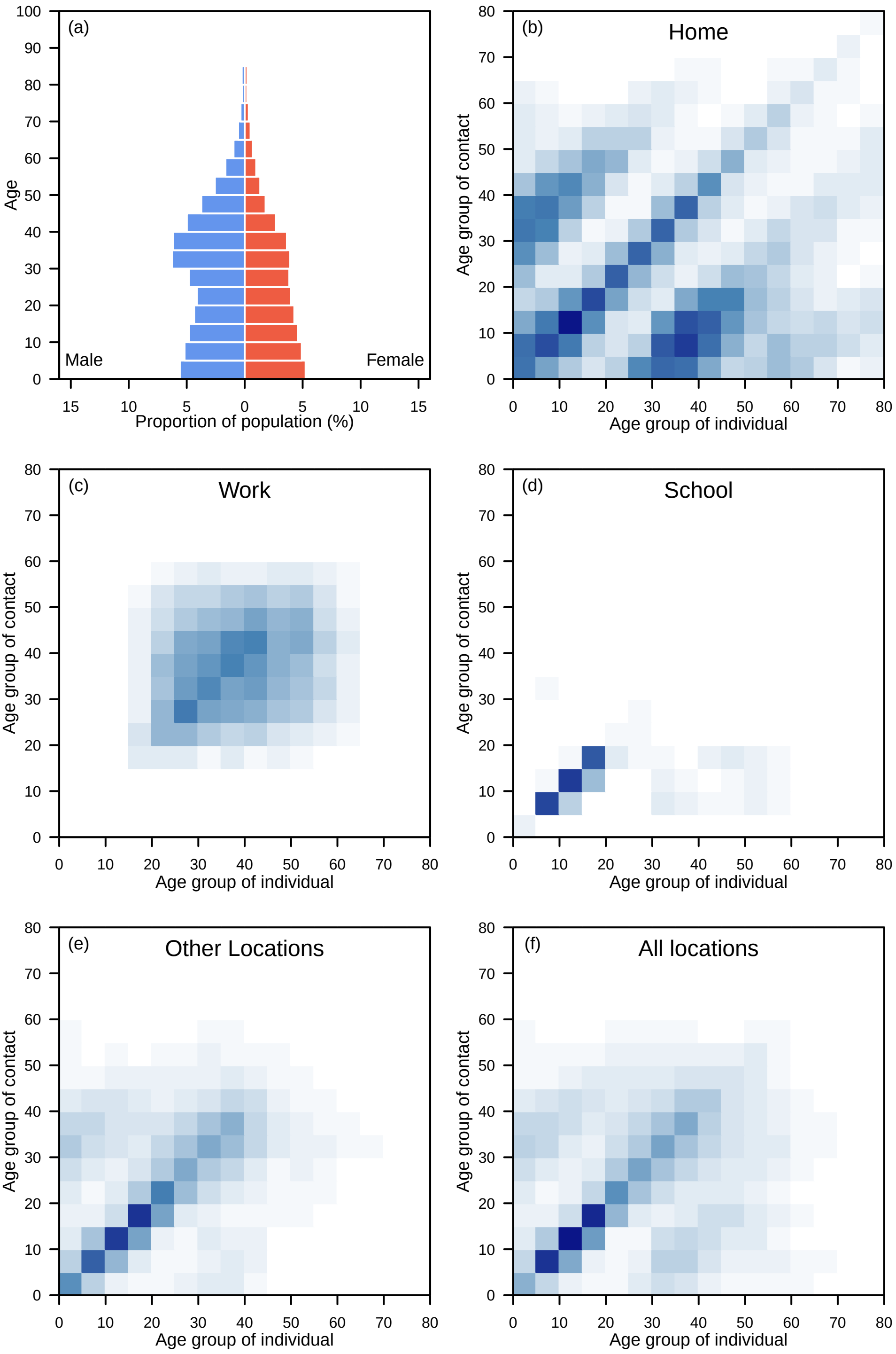

Senegal

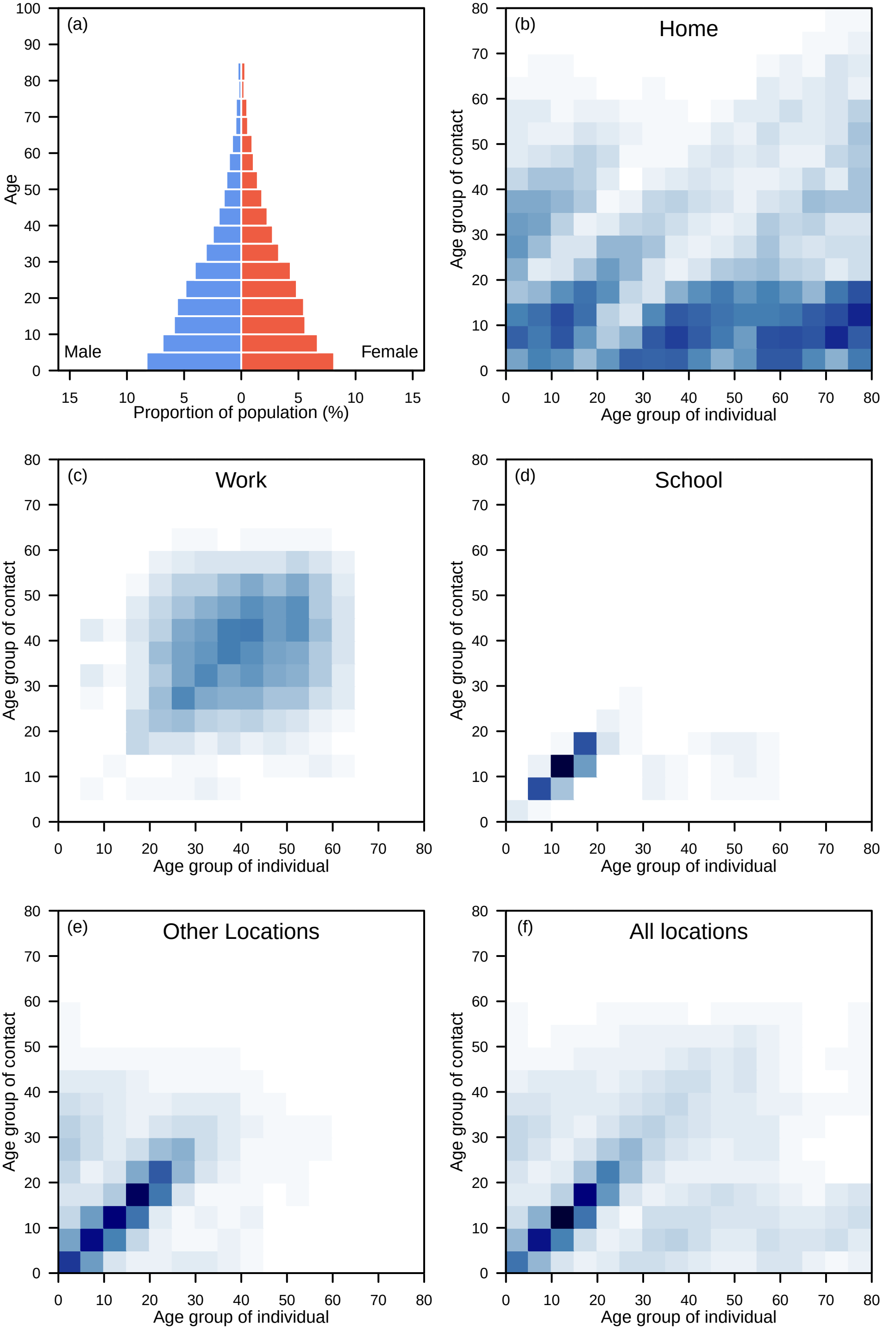

(a)
Age
Male
Female
Proportion of population (%)
(b)
Home
Age group of contact
Age group of individual
(c)
Work
Age group of contact
Age group of individual
(d)
School
Age group of contact
Age group of individual
(e)
Other Locations
Age group of contact
Age group of individual
(f)
All locations
Age group of contact
Age group of individual

Serbia

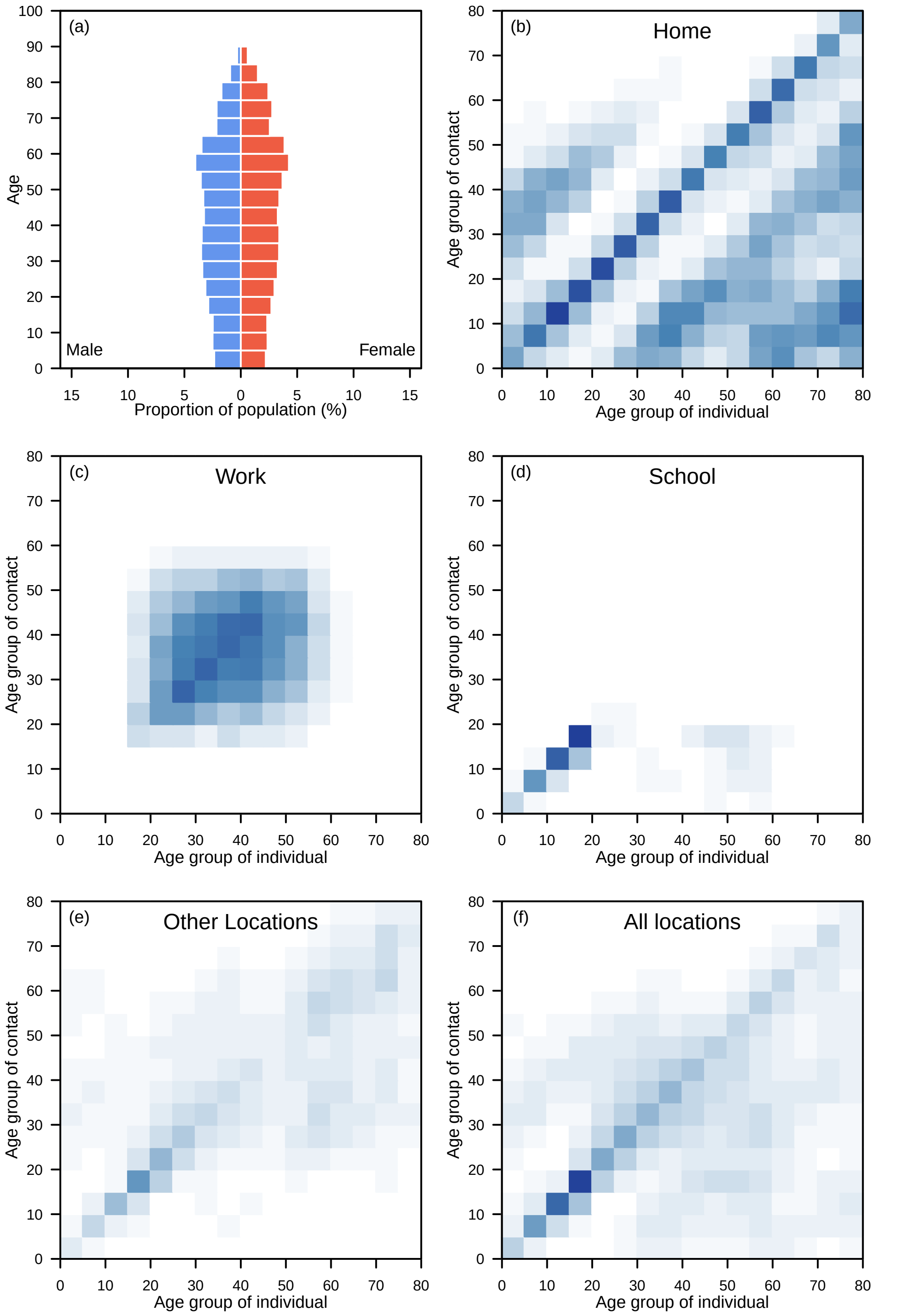

(a)
Age
Male
Female
Proportion of population (%)
(b)
Home
Age group of contact
Age group of individual
(c)
Work
Age group of contact
Age group of individual
(d)
School
Age group of contact
Age group of individual
(e)
Other Locations
Age group of contact
Age group of individual
(f)
All locations
Age group of contact
Age group of individual

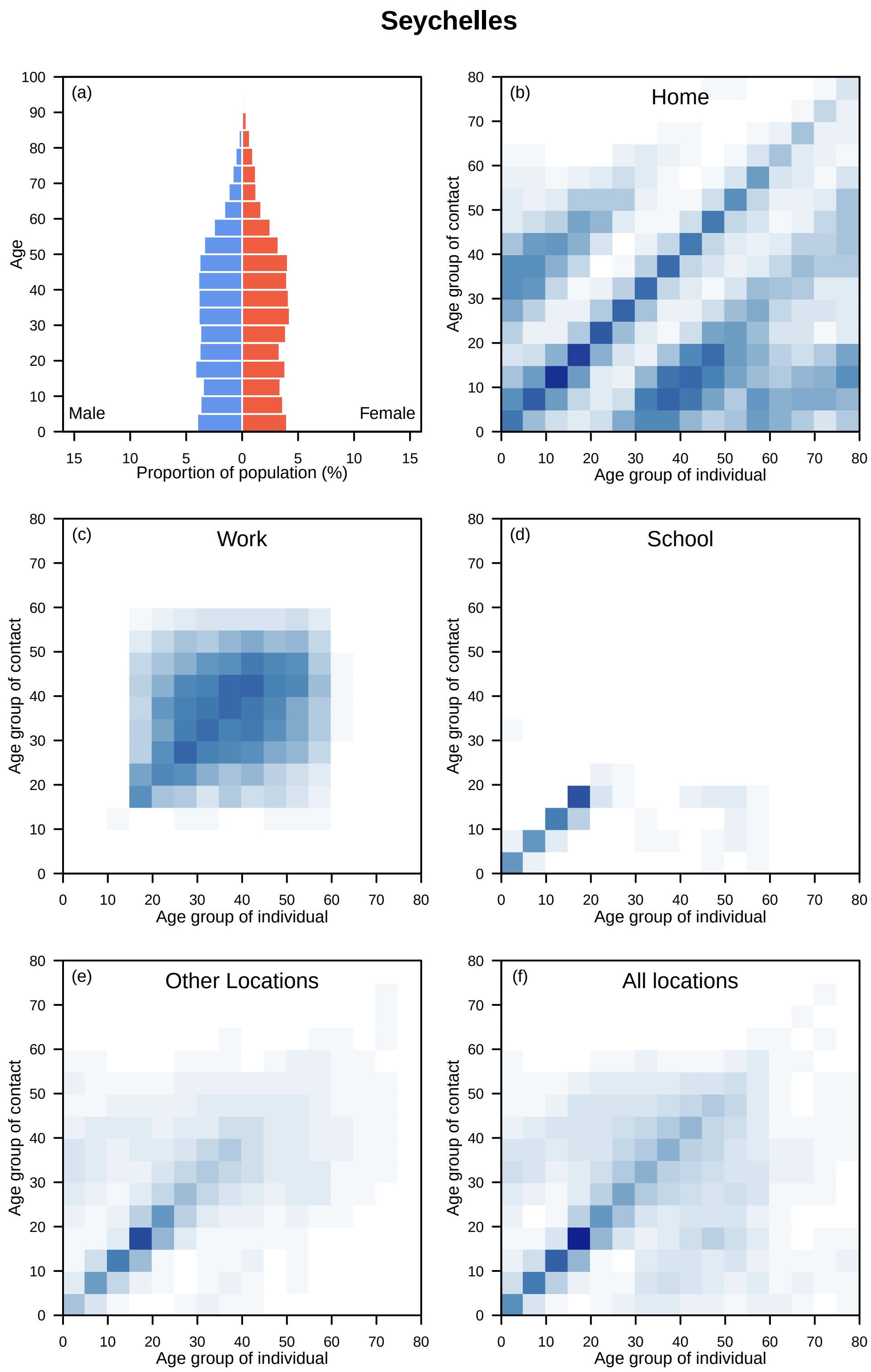
Seychelles
(a)
Male
Female
Age
Proportion of population (%)
(b) Home
(c) Work
(d) School
(e) Other Locations
(f) All locations
Age group of contact
Age group of individual

Sierra Leone

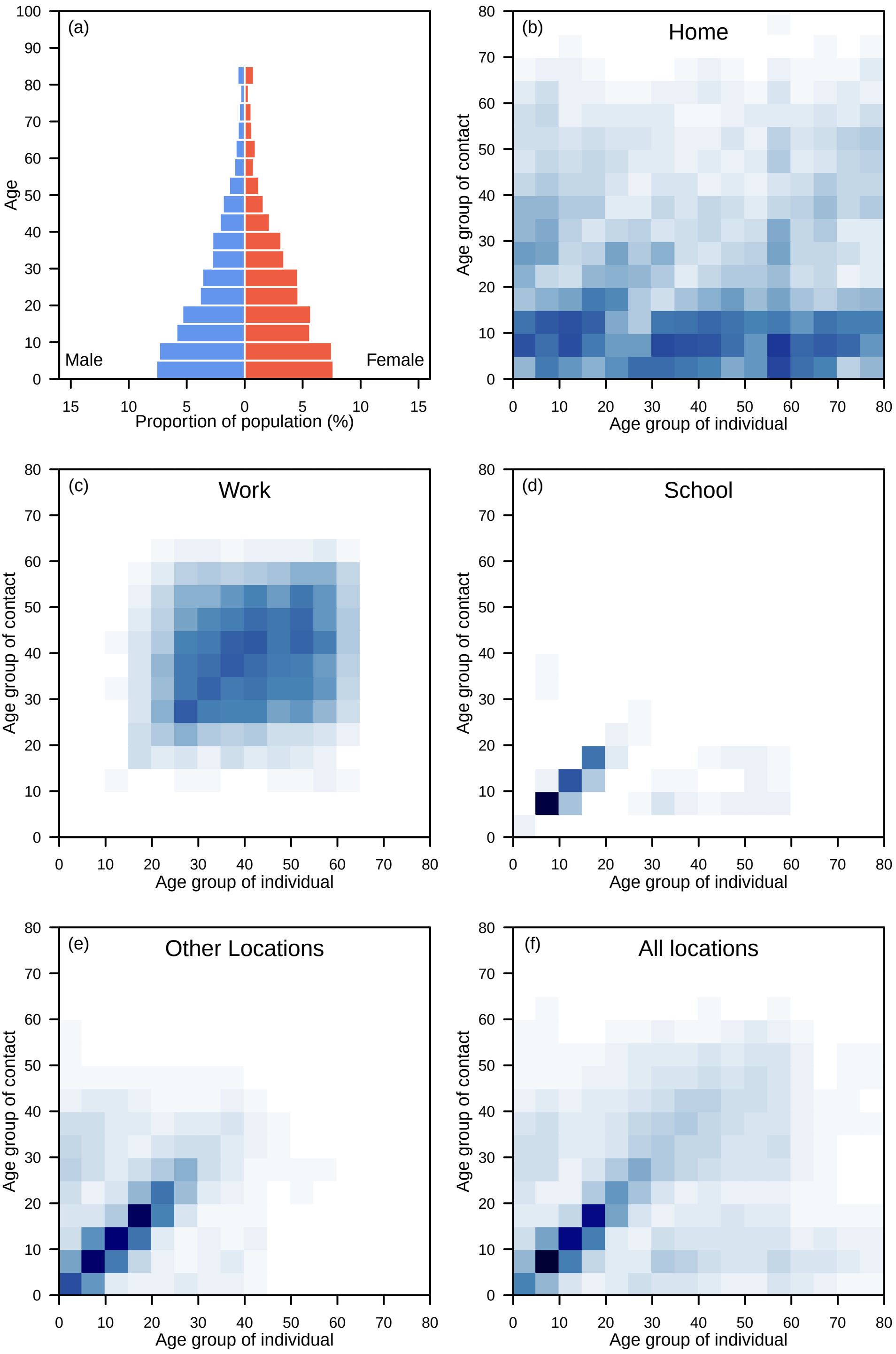

Singapore

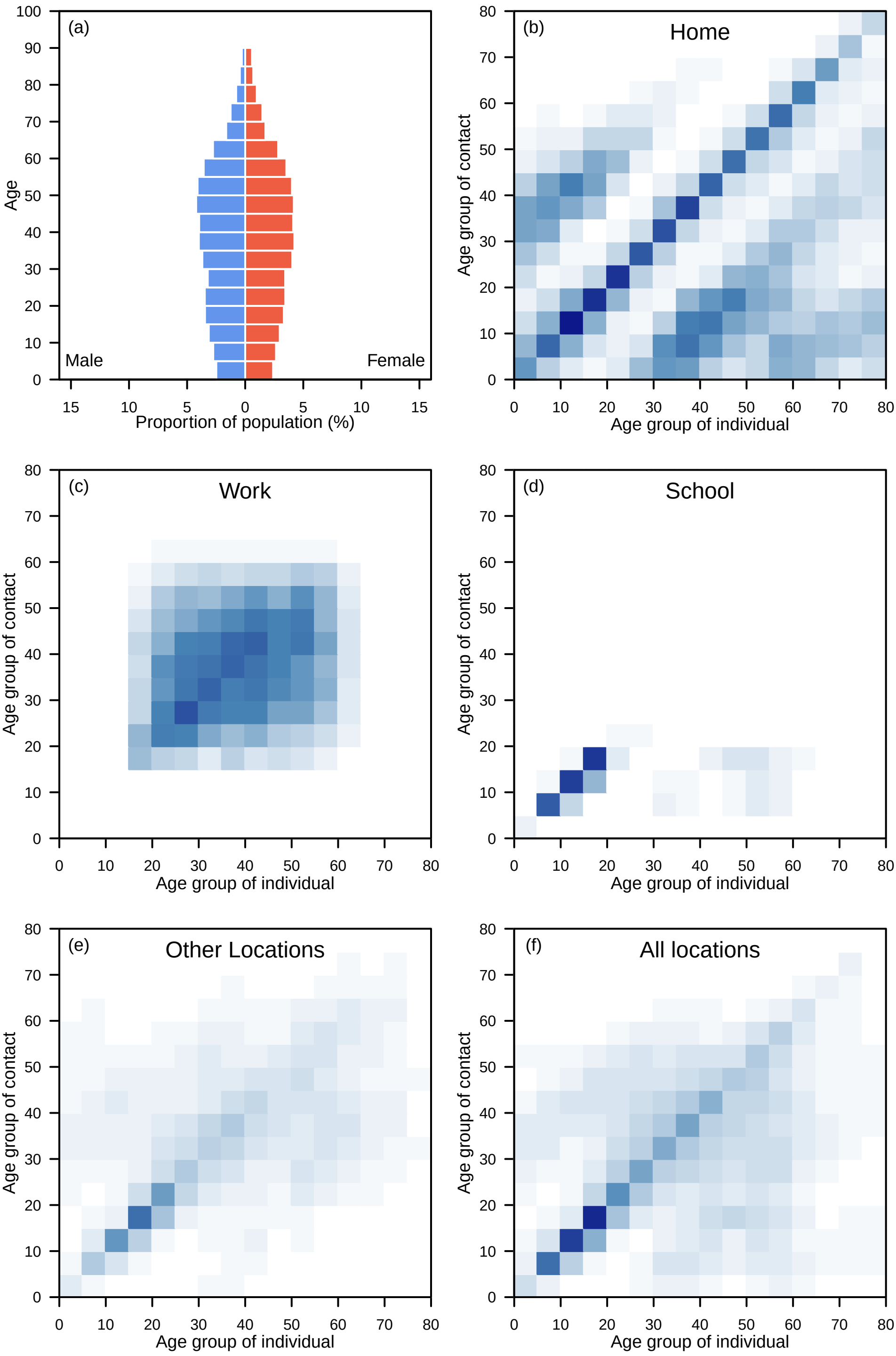

(a)
Age
Male
Female
Proportion of population (%)
(b)
Home
(c)
Work
(d)
School
(e)
Other Locations
(f)
All locations
Age group of contact
Age group of individual

Slovakia

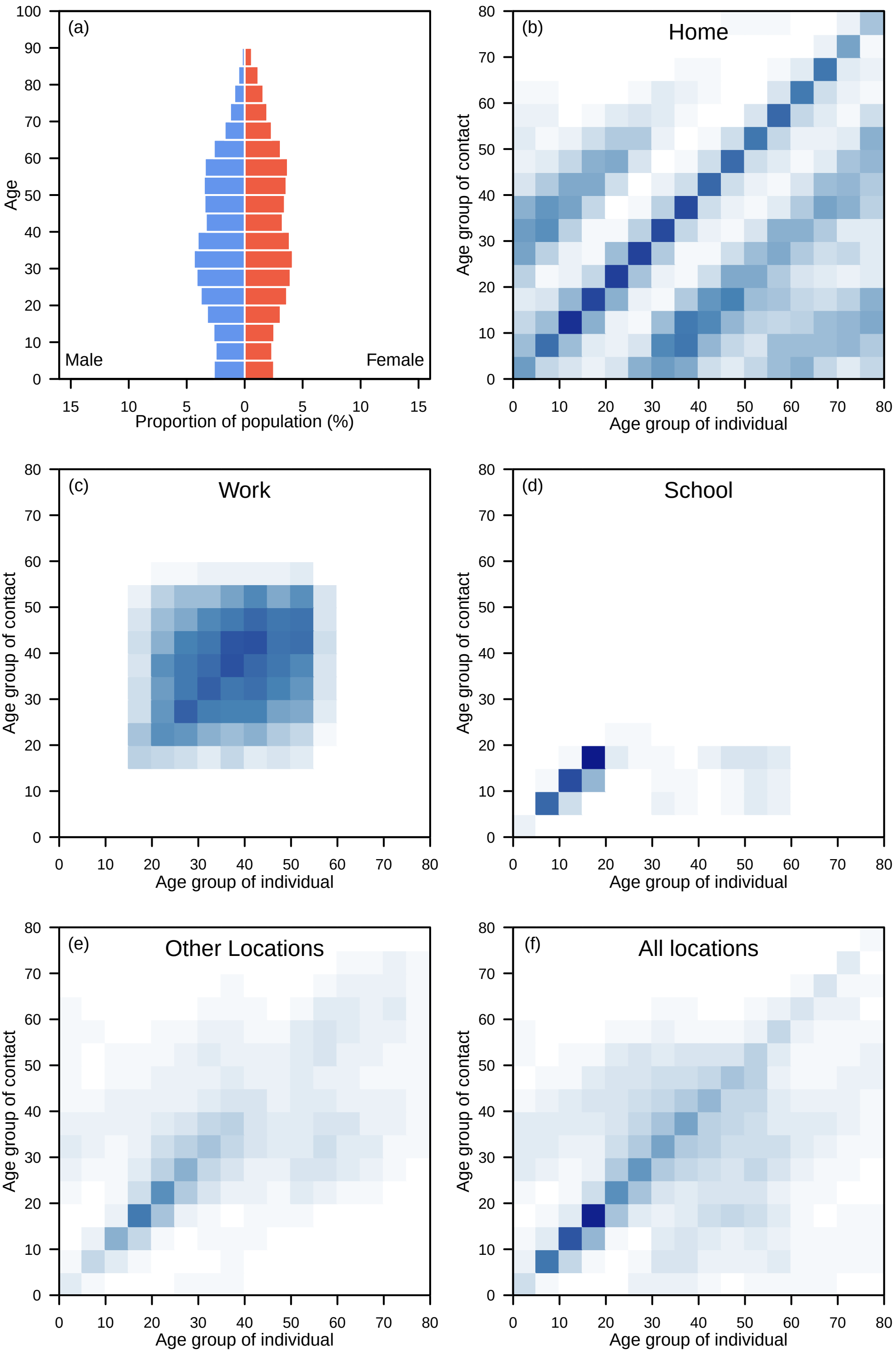

(a)
Age
Male
Female
Proportion of population (%)
(b)
Home
Age group of contact
Age group of individual
(c)
Work
Age group of contact
Age group of individual
(d)
School
Age group of contact
Age group of individual
(e)
Other Locations
Age group of contact
Age group of individual
(f)
All locations
Age group of contact
Age group of individual

Slovenia

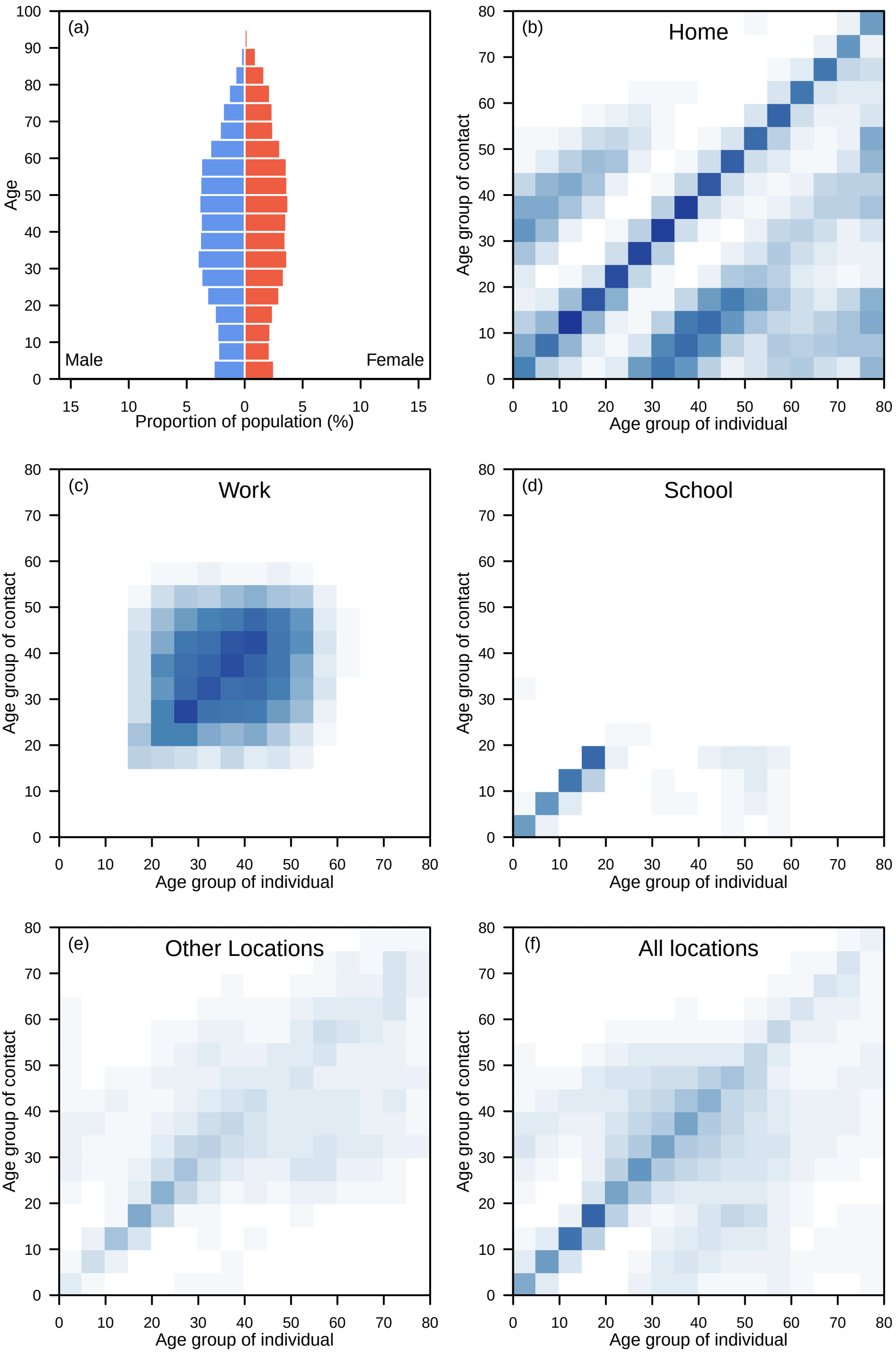

(a)
Male
Female
Age
Proportion of population (%)
(b) Home
(c) Work
(d) School
(e) Other Locations
(f) All locations
Age group of contact
Age group of individual

Solomon Islands

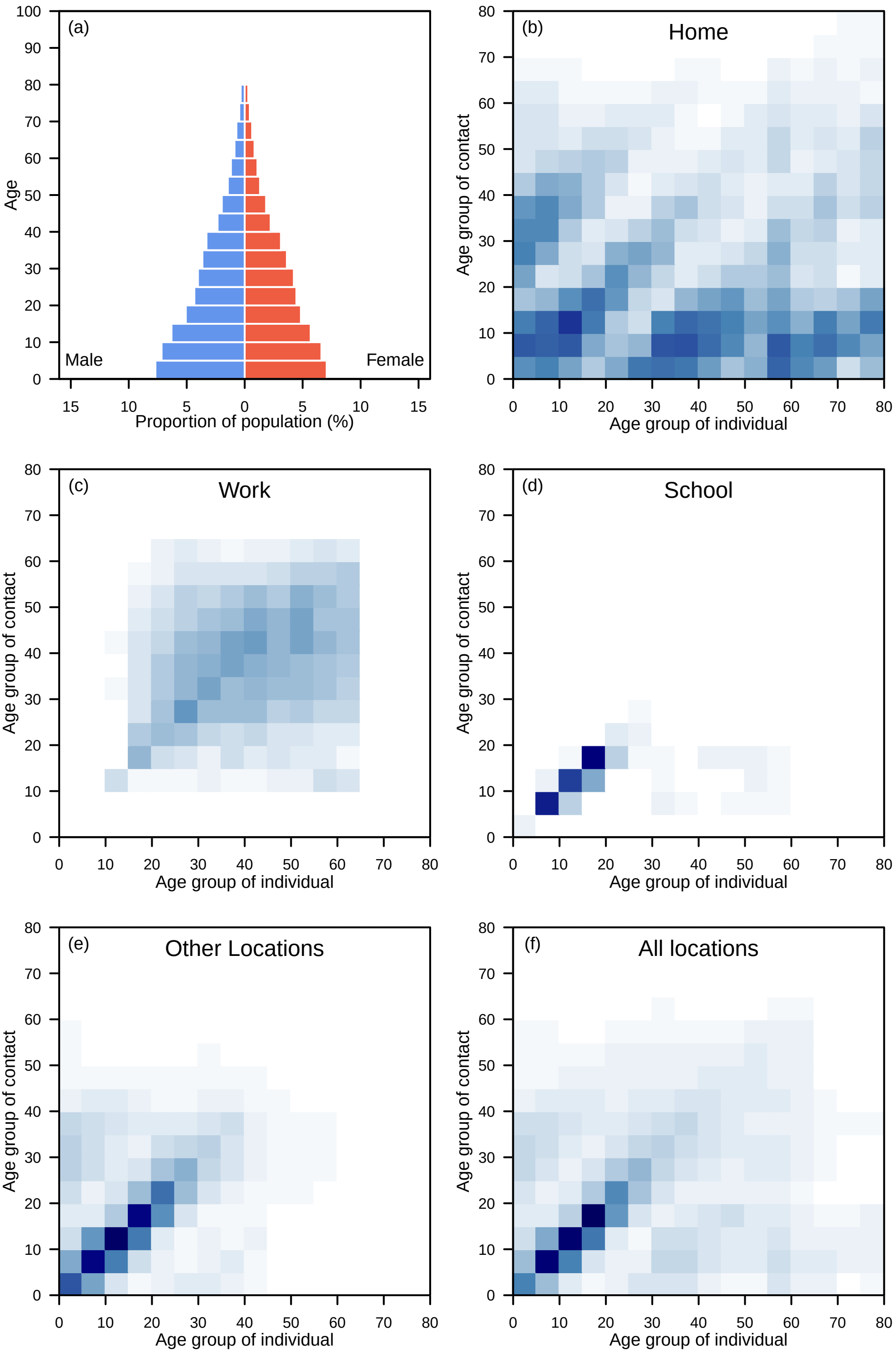

South Africa

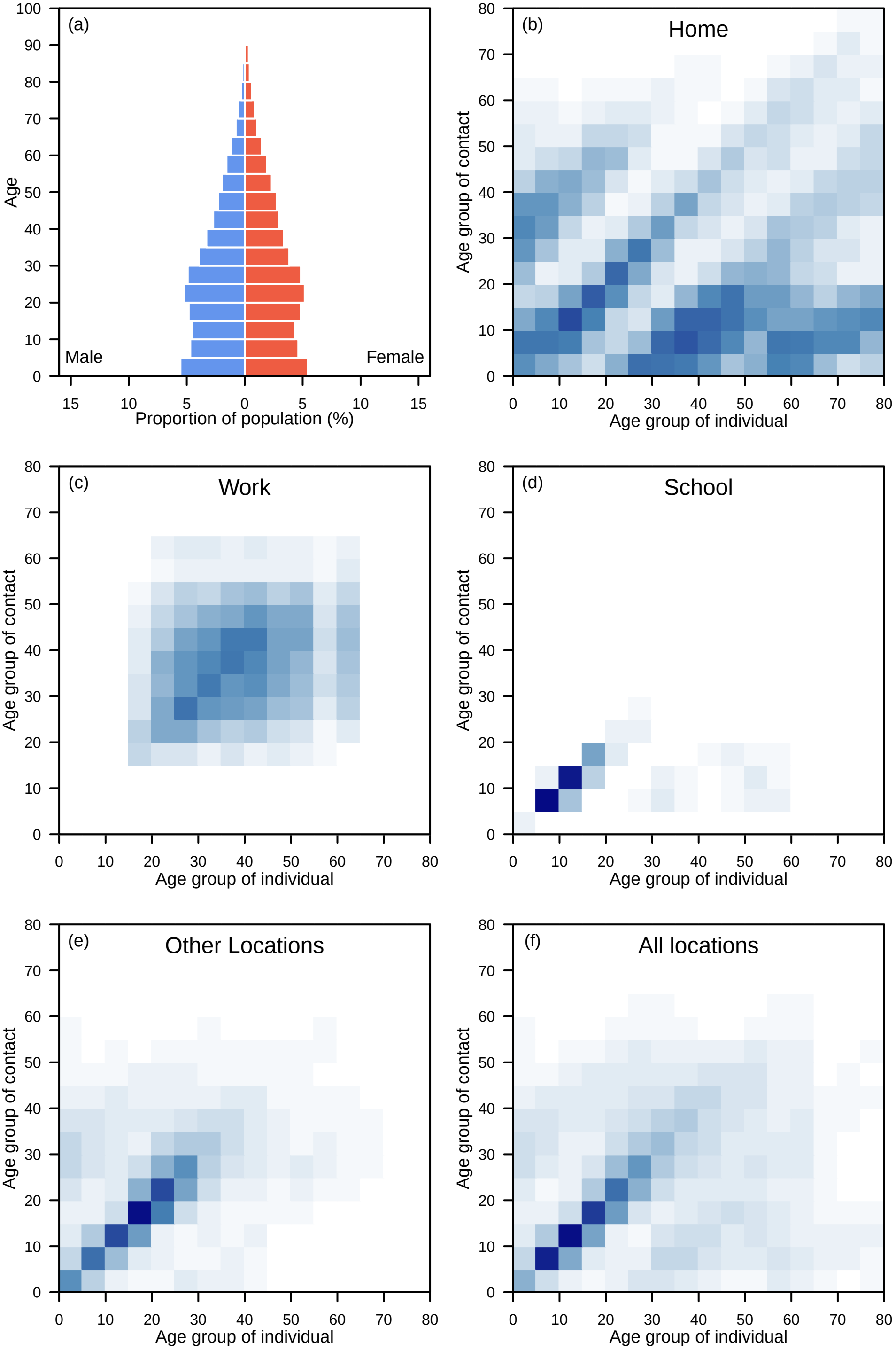

Spain

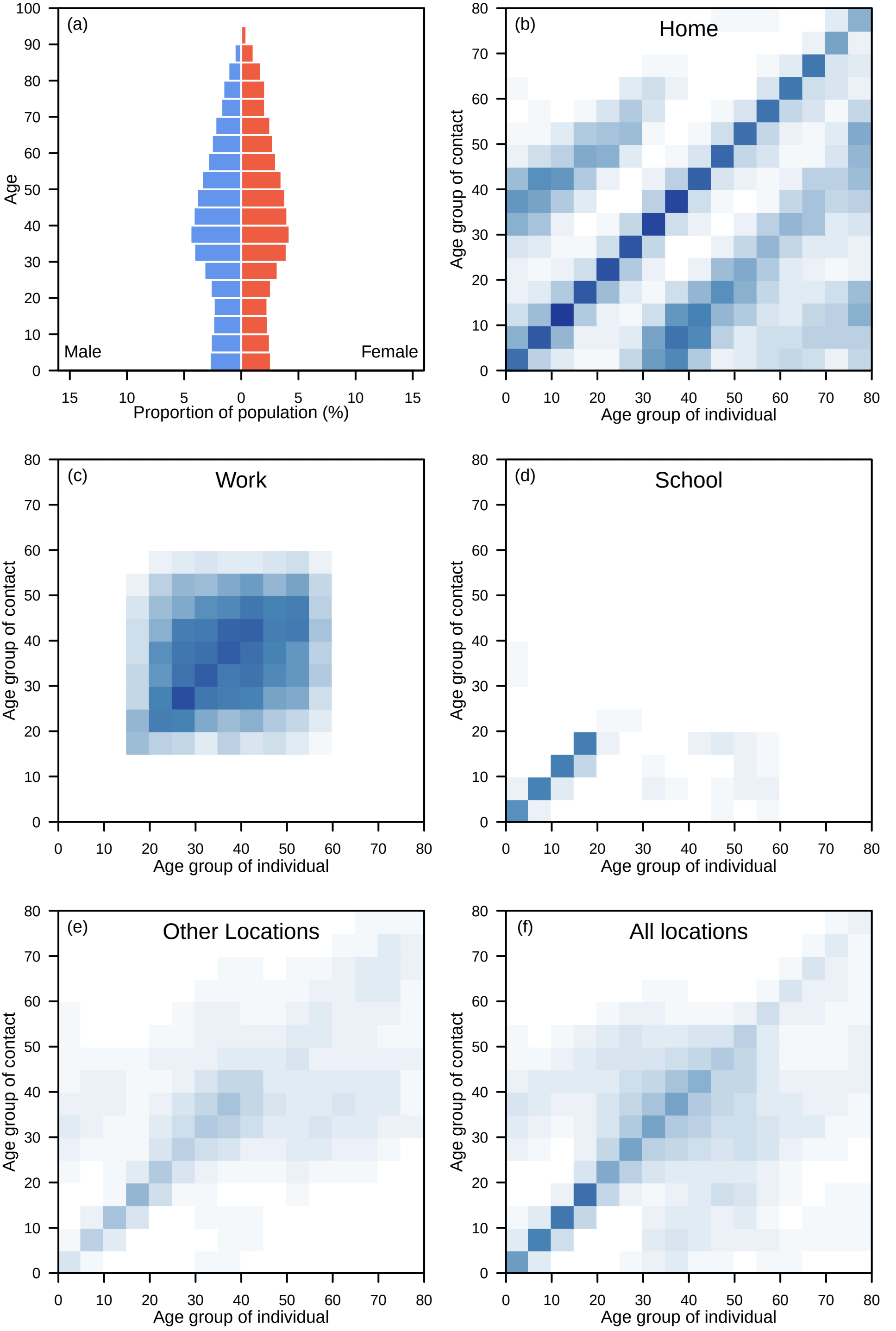

(a)
Age
Male
Female
Proportion of population (%)
(b)
Home
Age group of contact
Age group of individual
(c)
Work
(d)
School
(e)
Other Locations
(f)
All locations

Sri Lanka

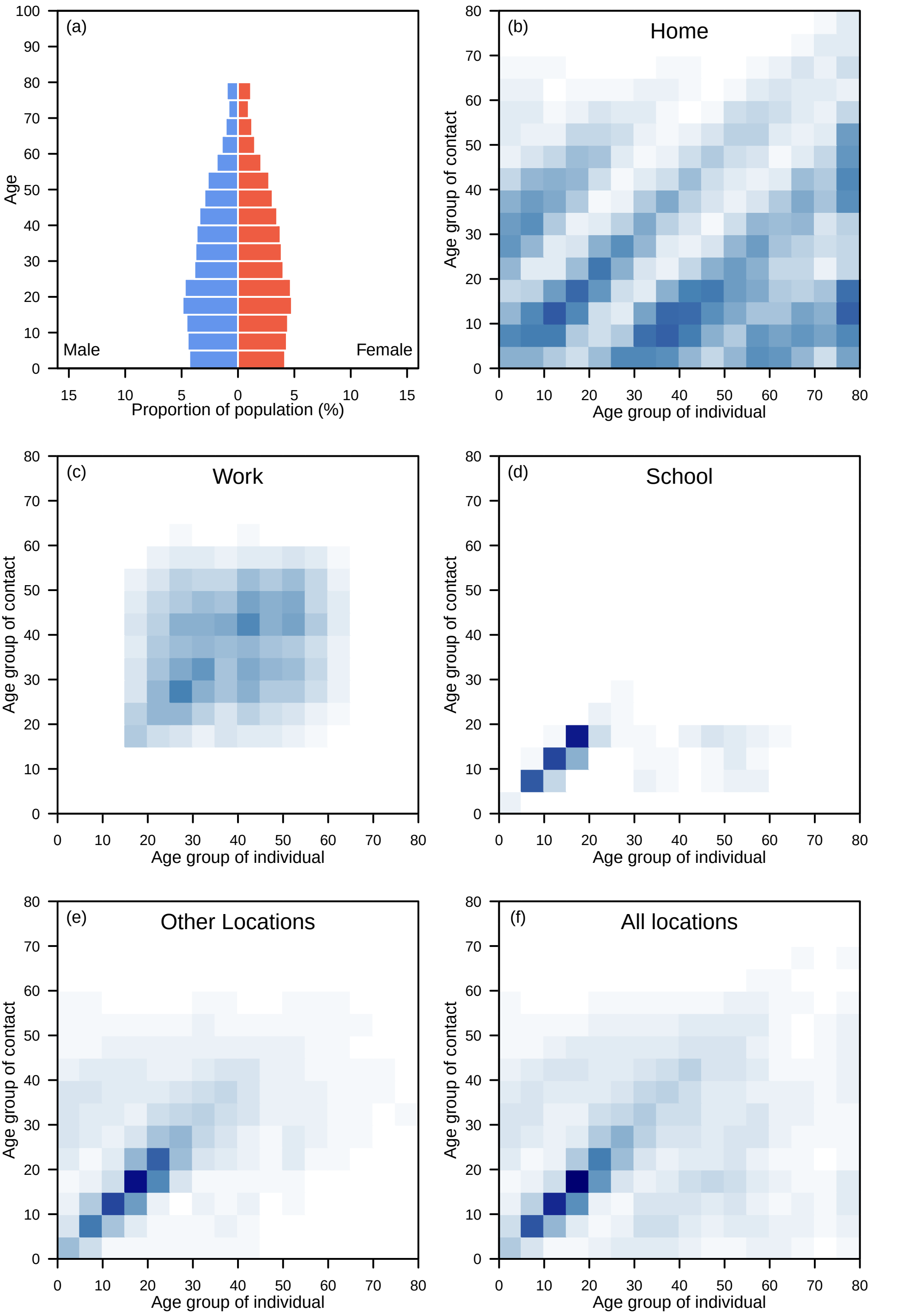

(a)
Age
Male
Female
Proportion of population (%)
(b)
Home
Age group of contact
Age group of individual
(c)
Work
(d)
School
(e)
Other Locations
(f)
All locations

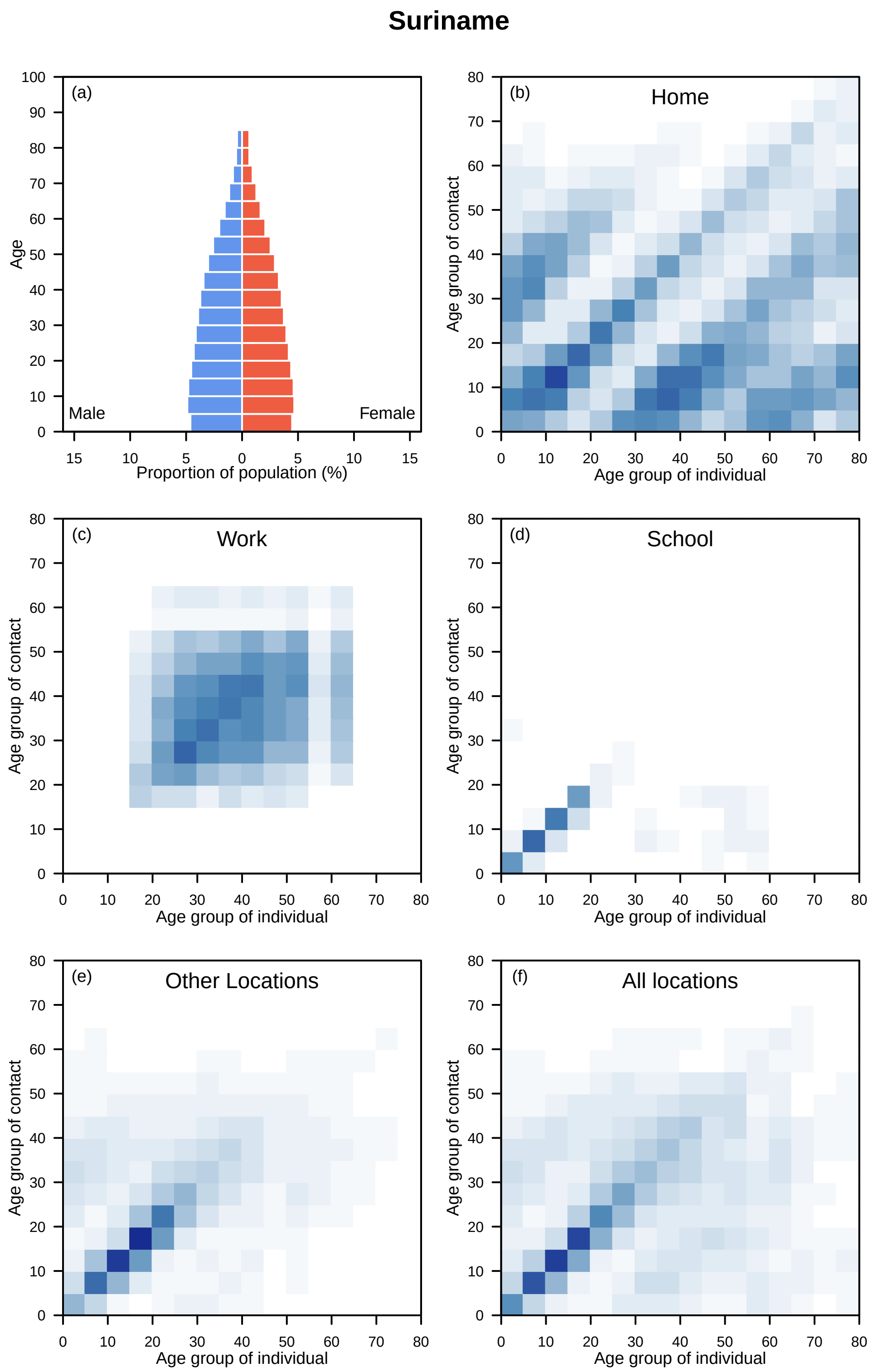

Suriname
(a)
Male
Female
Age
Proportion of population (%)
(b)
Home
(c)
Work
(d)
School
(e)
Other Locations
(f)
All locations
Age group of contact
Age group of individual

Sweden

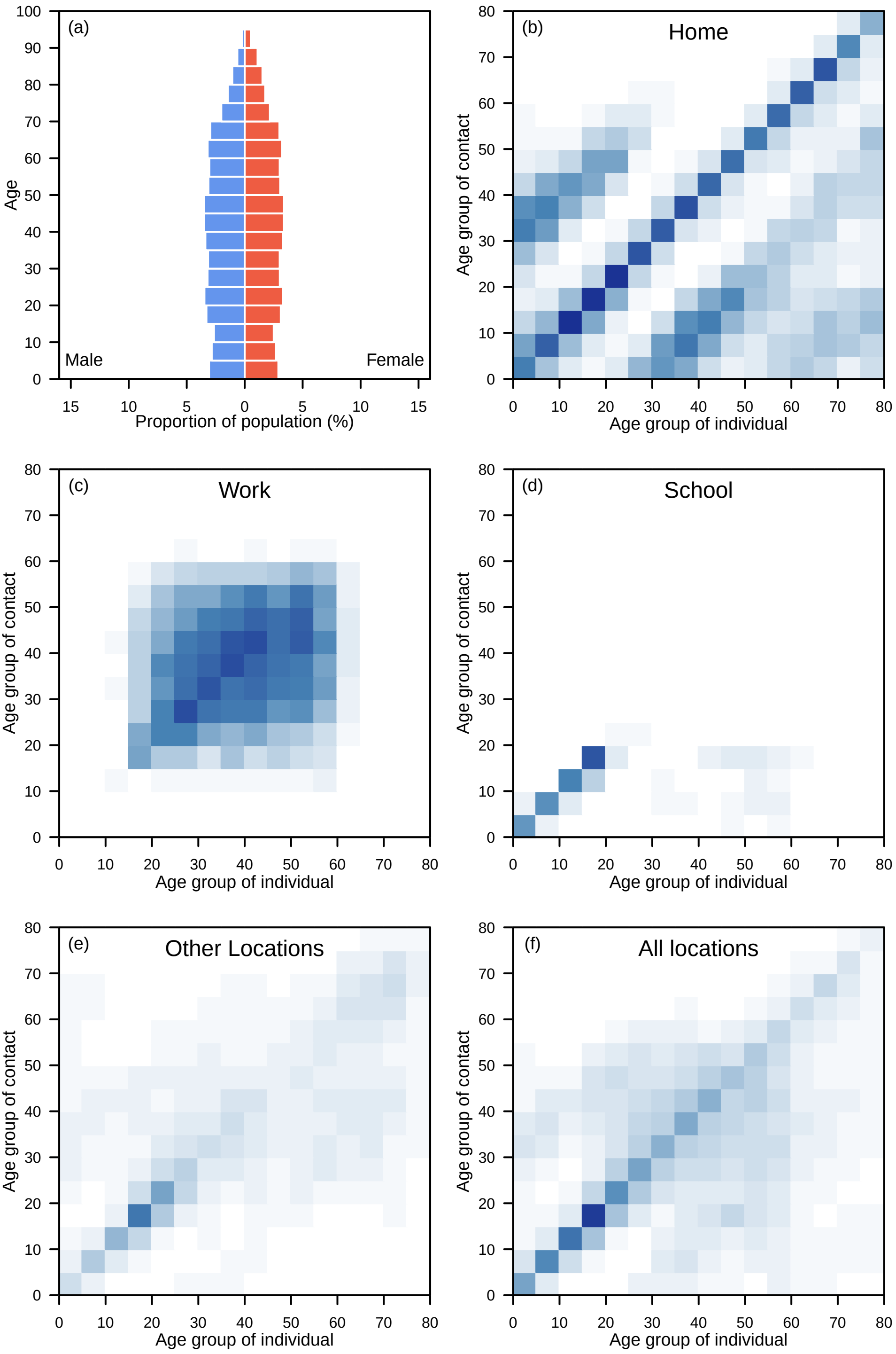

(a)
Age
Male
Female
Proportion of population (%)
(b)
Home
Age group of contact
Age group of individual
(c)
Work
Age group of contact
Age group of individual
(d)
School
Age group of contact
Age group of individual
(e)
Other Locations
Age group of contact
Age group of individual
(f)
All locations
Age group of contact
Age group of individual

Switzerland

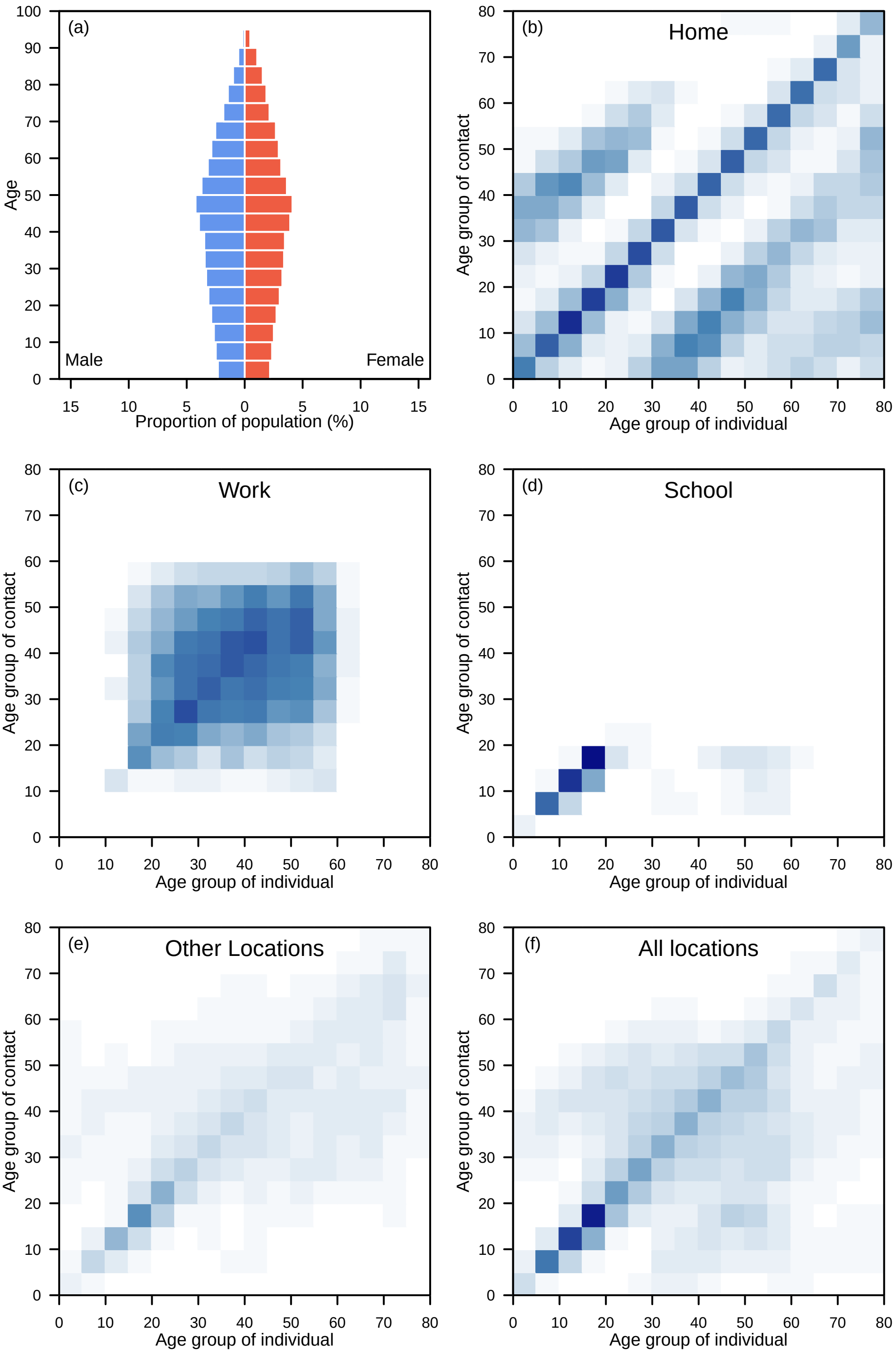

Syrian Arab Republic

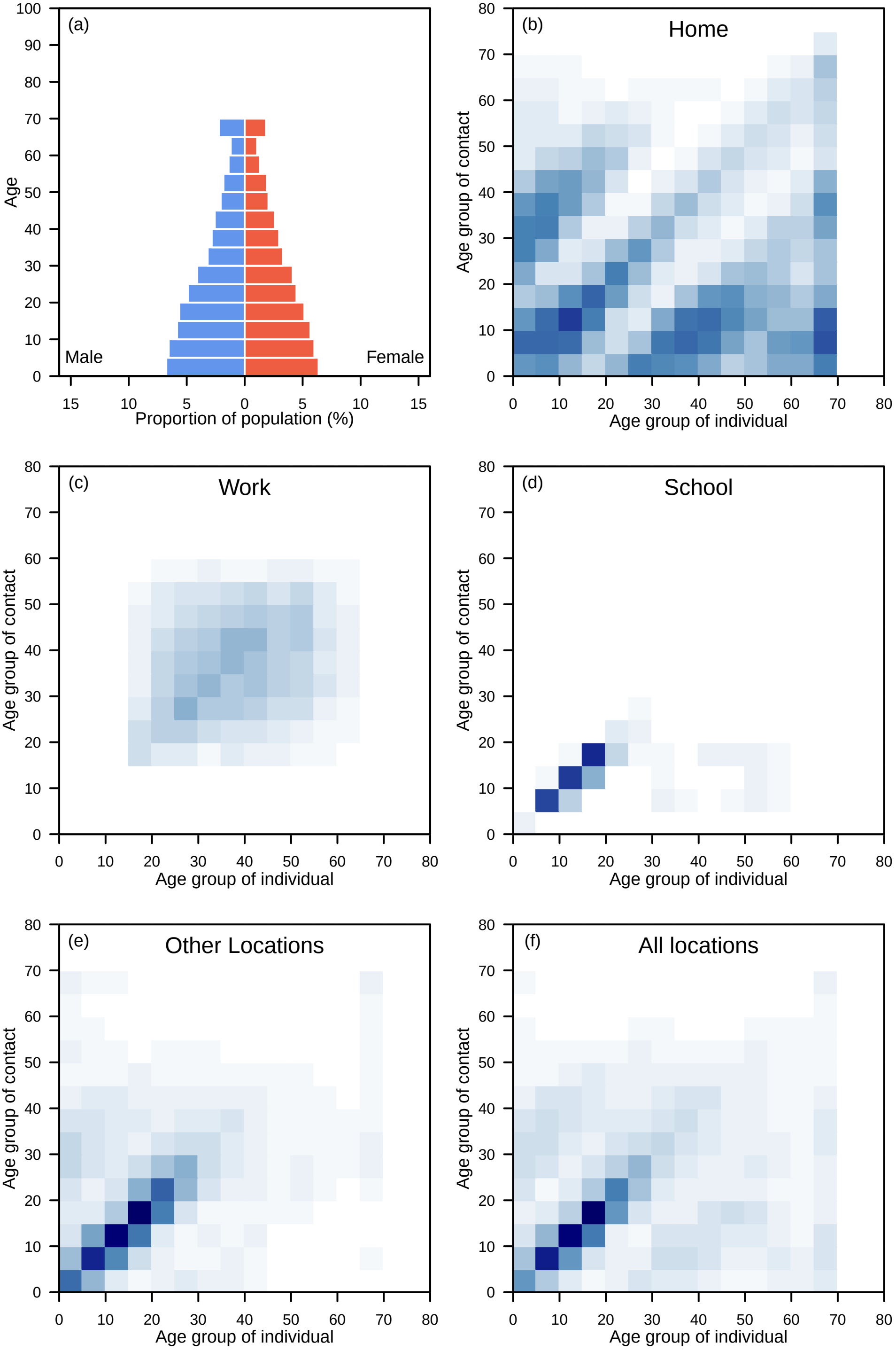

Taiwan

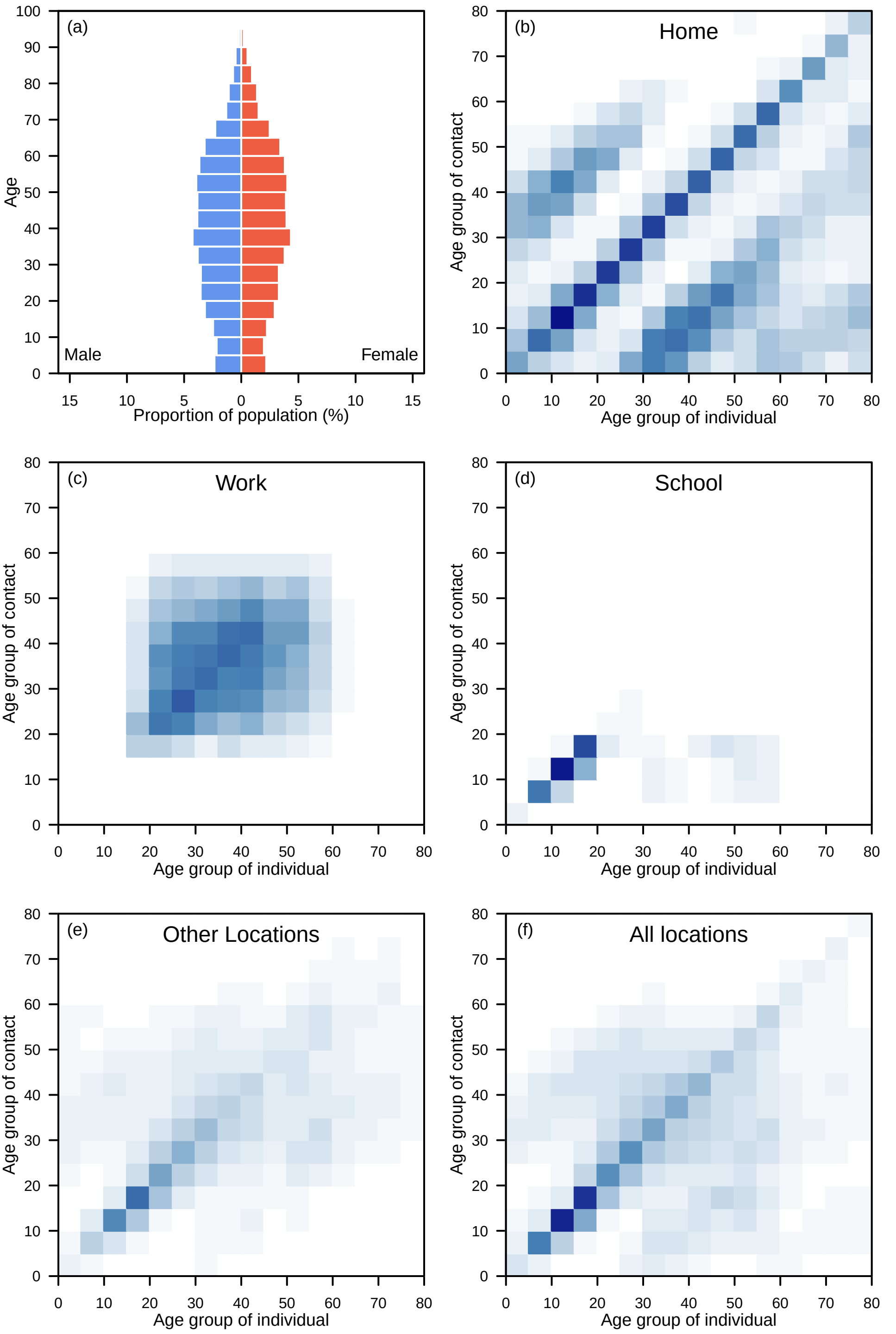

(a)
Age
Male
Female
Proportion of population (%)
(b)
Home
Age group of contact
Age group of individual
(c)
Work
Age group of contact
Age group of individual
(d)
School
Age group of contact
Age group of individual
(e)
Other Locations
Age group of contact
Age group of individual
(f)
All locations
Age group of contact
Age group of individual

Tajikistan

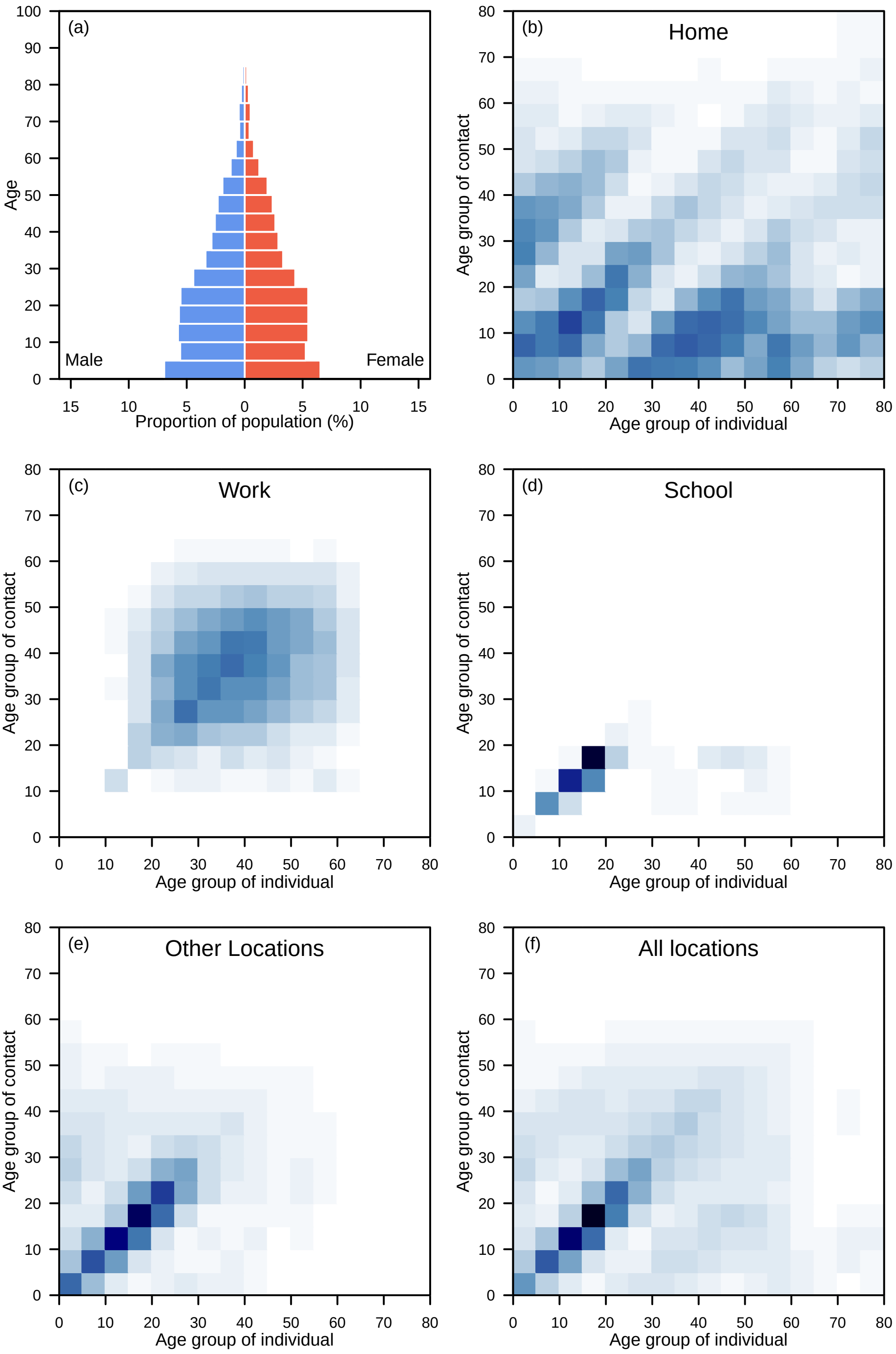
(a)
Age
Male
Female
Proportion of population (%)
(b)
Home
Age group of contact
Age group of individual
(c)
Work
Age group of contact
Age group of individual
(d)
School
Age group of contact
Age group of individual
(e)
Other Locations
Age group of contact
Age group of individual
(f)
All locations
Age group of contact
Age group of individual

TFYR of Macedonia

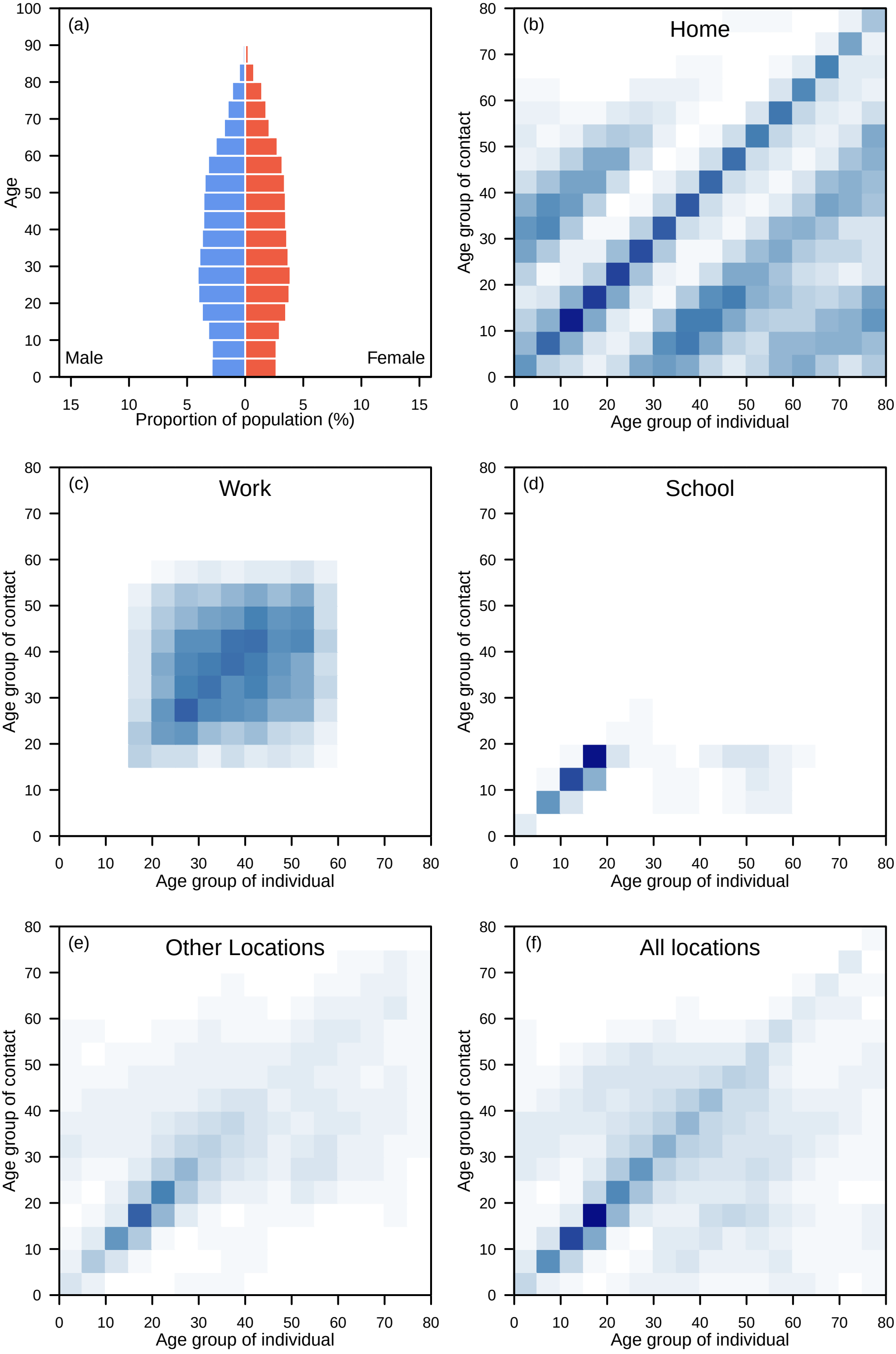

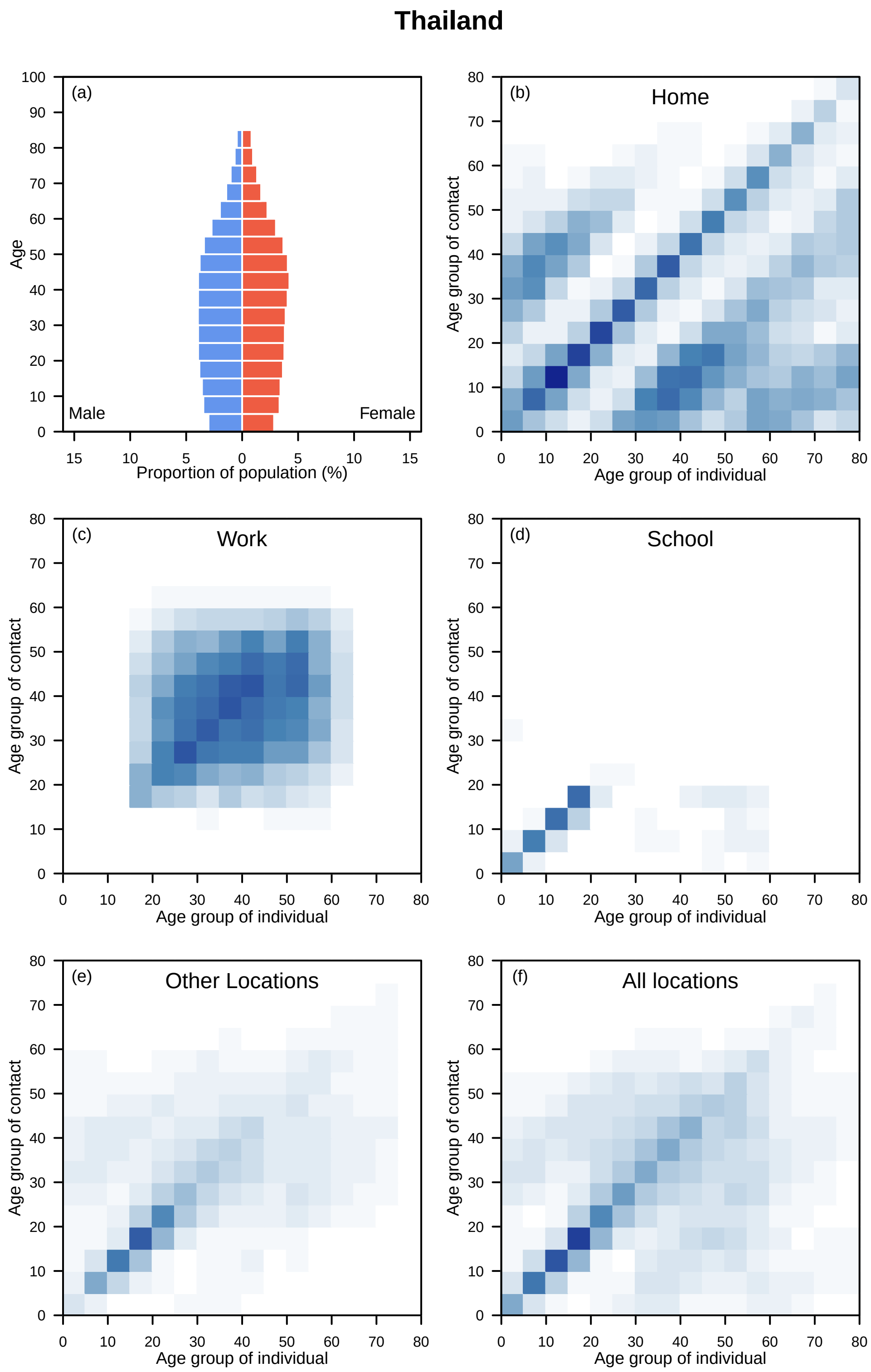
Thailand
(a)
Age
Male
Female
Proportion of population (%)
(b)
Home
Age group of contact
Age group of individual
(c)
Work
(d)
School
(e)
Other Locations
(f)
All locations

Timor−Leste

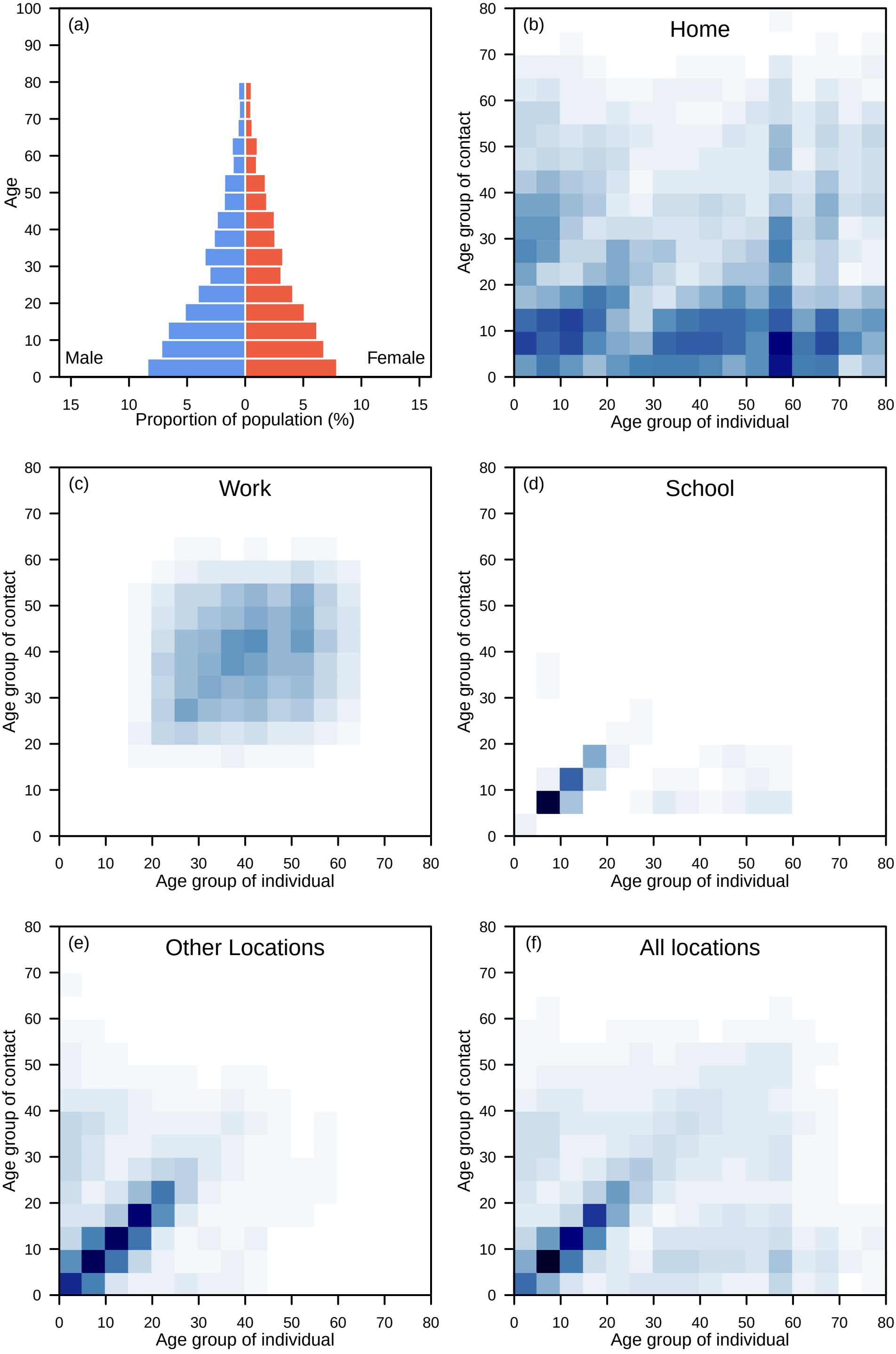

(a)
Age
Male
Female
Proportion of population (%)
(b)
Home
Age group of contact
Age group of individual
(c)
Work
Age group of contact
Age group of individual
(d)
School
Age group of contact
Age group of individual
(e)
Other Locations
Age group of contact
Age group of individual
(f)
All locations
Age group of contact
Age group of individual

Tonga

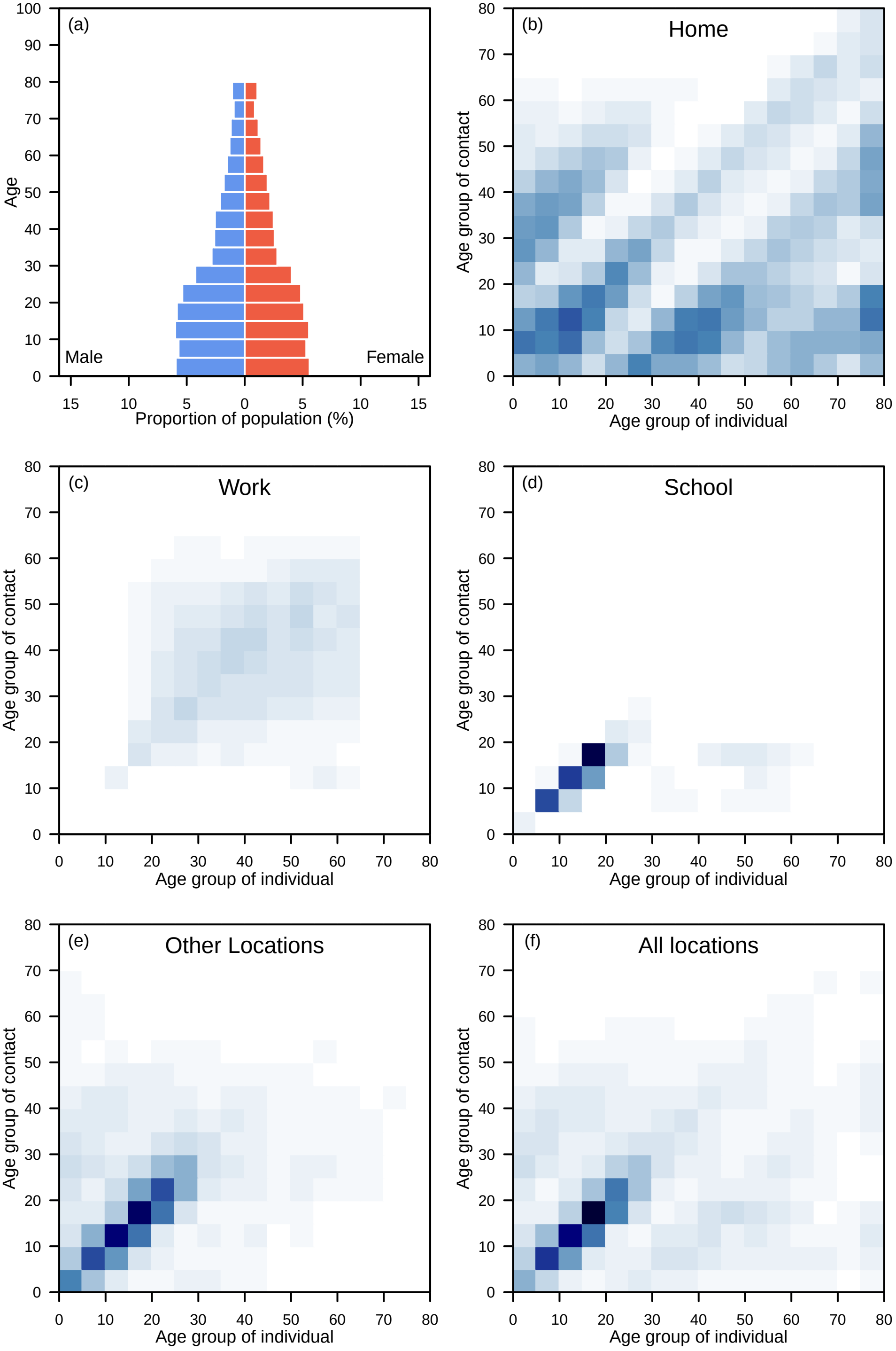

(a)
Age
Male
Female
Proportion of population (%)
(b)
Home
(c)
Work
(d)
School
(e)
Other Locations
(f)
All locations
Age group of contact
Age group of individual

Tunisia

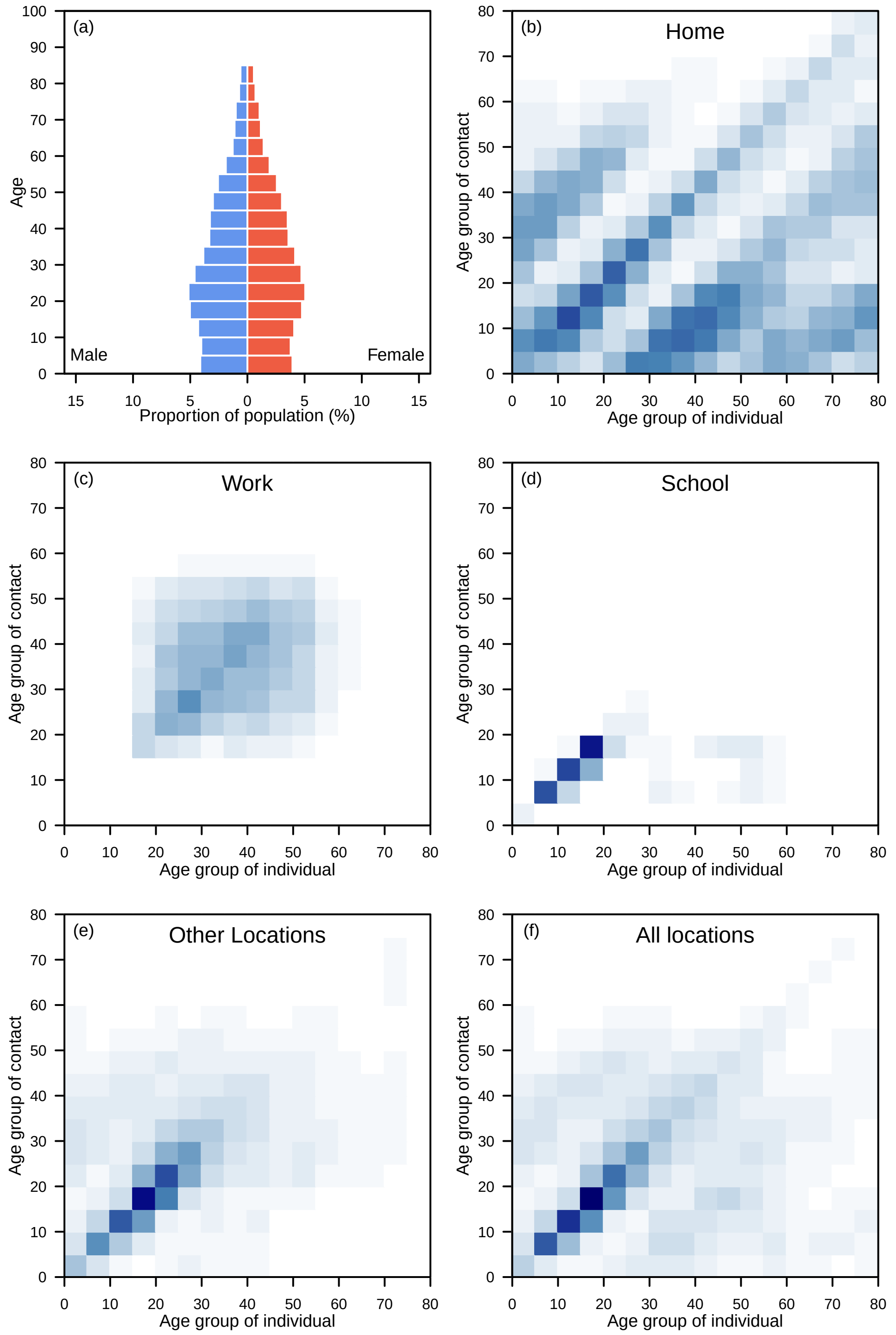

(a)
Age
Male
Female
Proportion of population (%)
(b)
Home
Age group of contact
Age group of individual
(c)
Work
Age group of contact
Age group of individual
(d)
School
Age group of contact
Age group of individual
(e)
Other Locations
Age group of contact
Age group of individual
(f)
All locations
Age group of contact
Age group of individual

Turkey

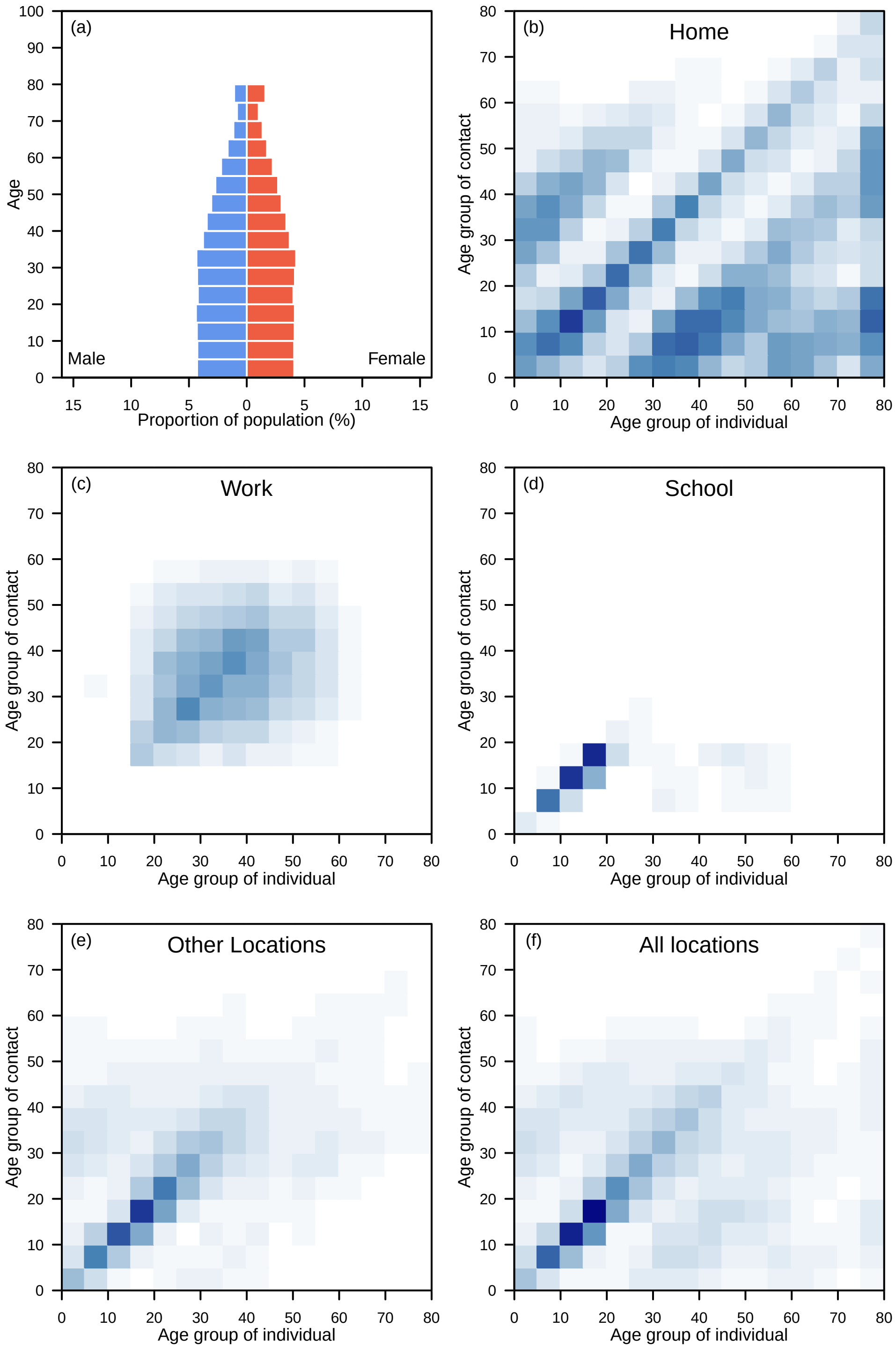

(a)
Age
Male
Female
Proportion of population (%)
(b)
Home
Age group of contact
Age group of individual
(c)
Work
Age group of contact
Age group of individual
(d)
School
Age group of contact
Age group of individual
(e)
Other Locations
Age group of contact
Age group of individual
(f)
All locations
Age group of contact
Age group of individual

Uganda

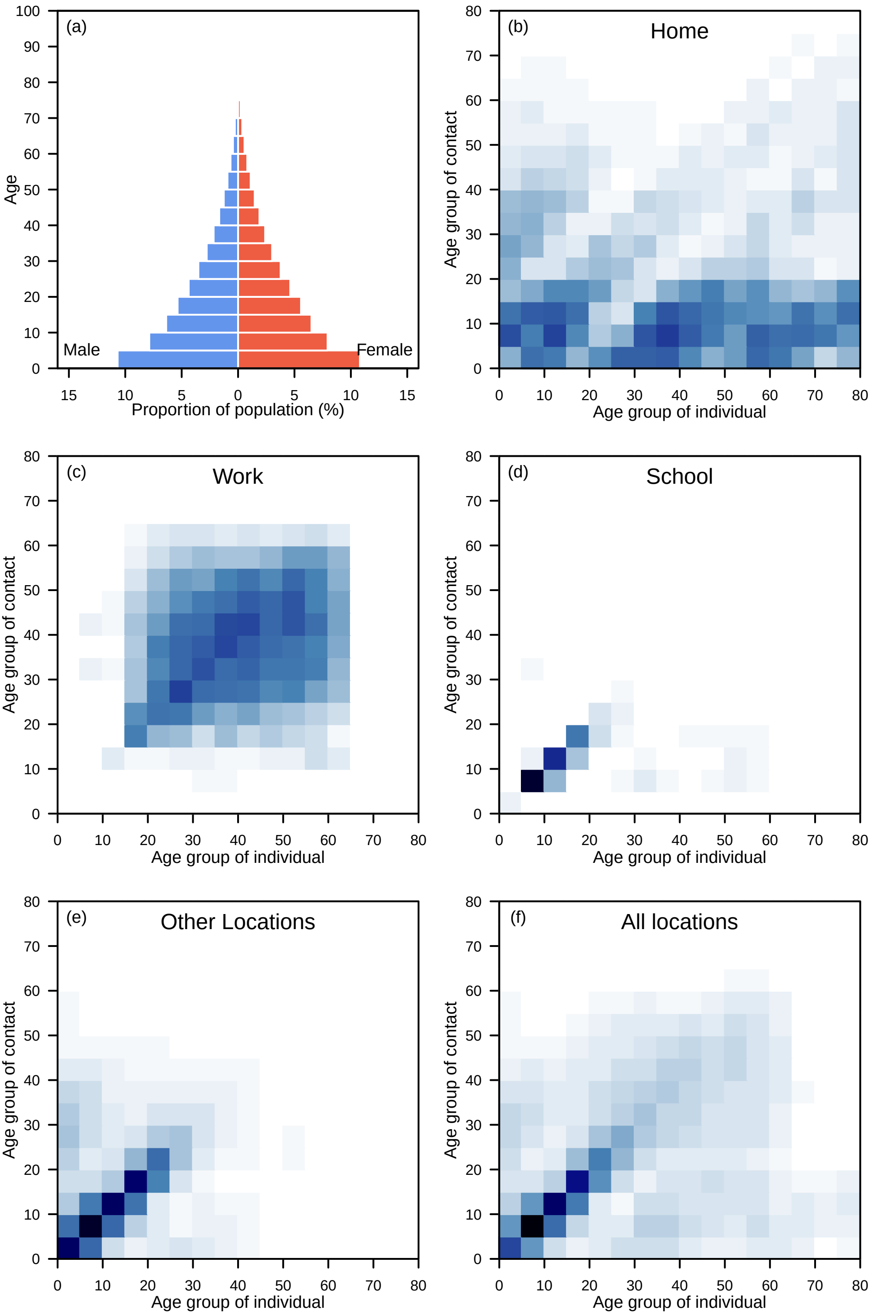

(a)
Age
Male
Female
Proportion of population (%)
(b)
Home
Age group of contact
Age group of individual
(c)
Work
Age group of contact
Age group of individual
(d)
School
Age group of contact
Age group of individual
(e)
Other Locations
Age group of contact
Age group of individual
(f)
All locations
Age group of contact
Age group of individual

Ukraine

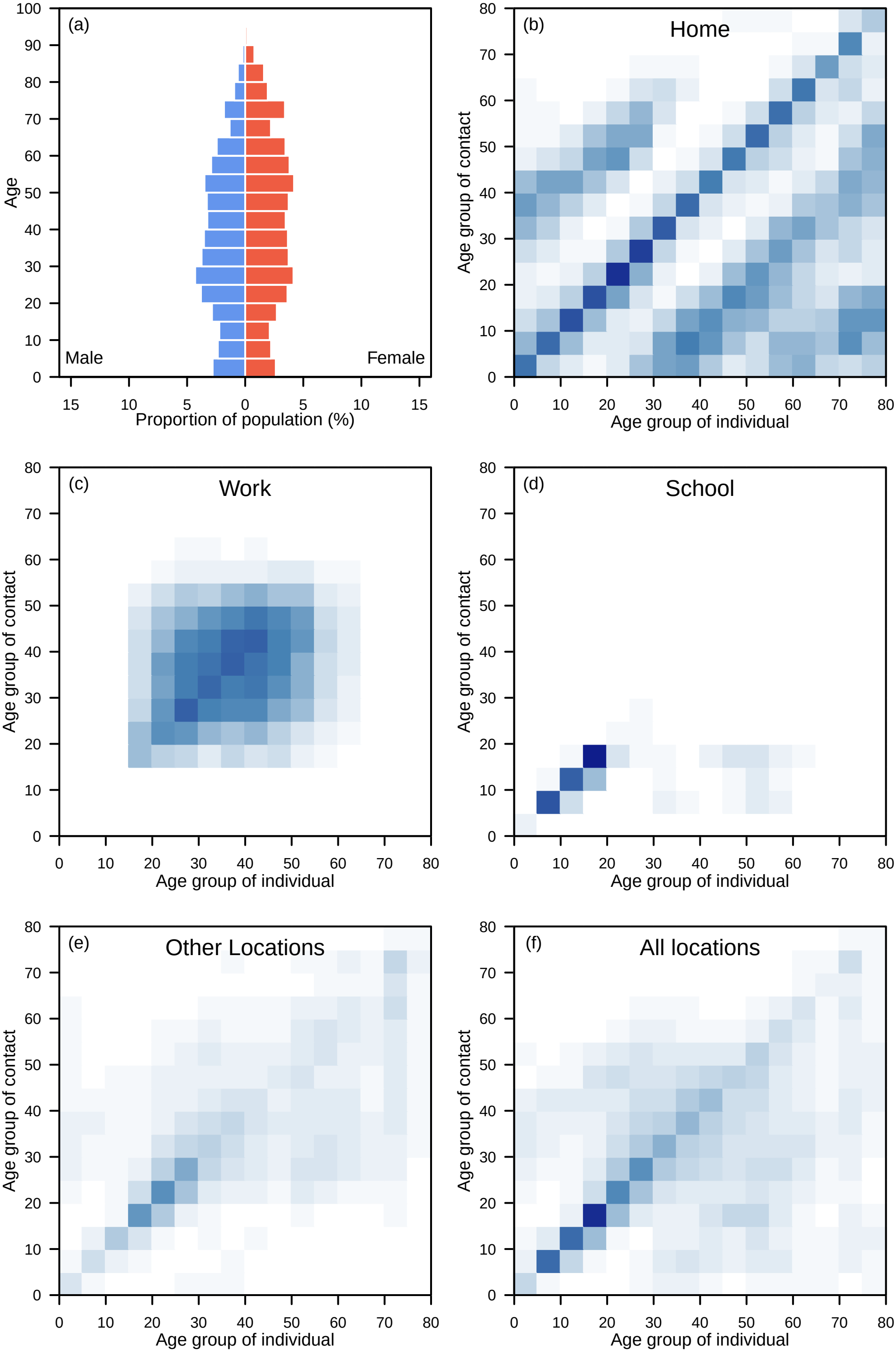

(a)
Male
Female
Age
Proportion of population (%)
(b) Home
(c) Work
(d) School
(e) Other Locations
(f) All locations
Age group of contact
Age group of individual

United Arab Emirates

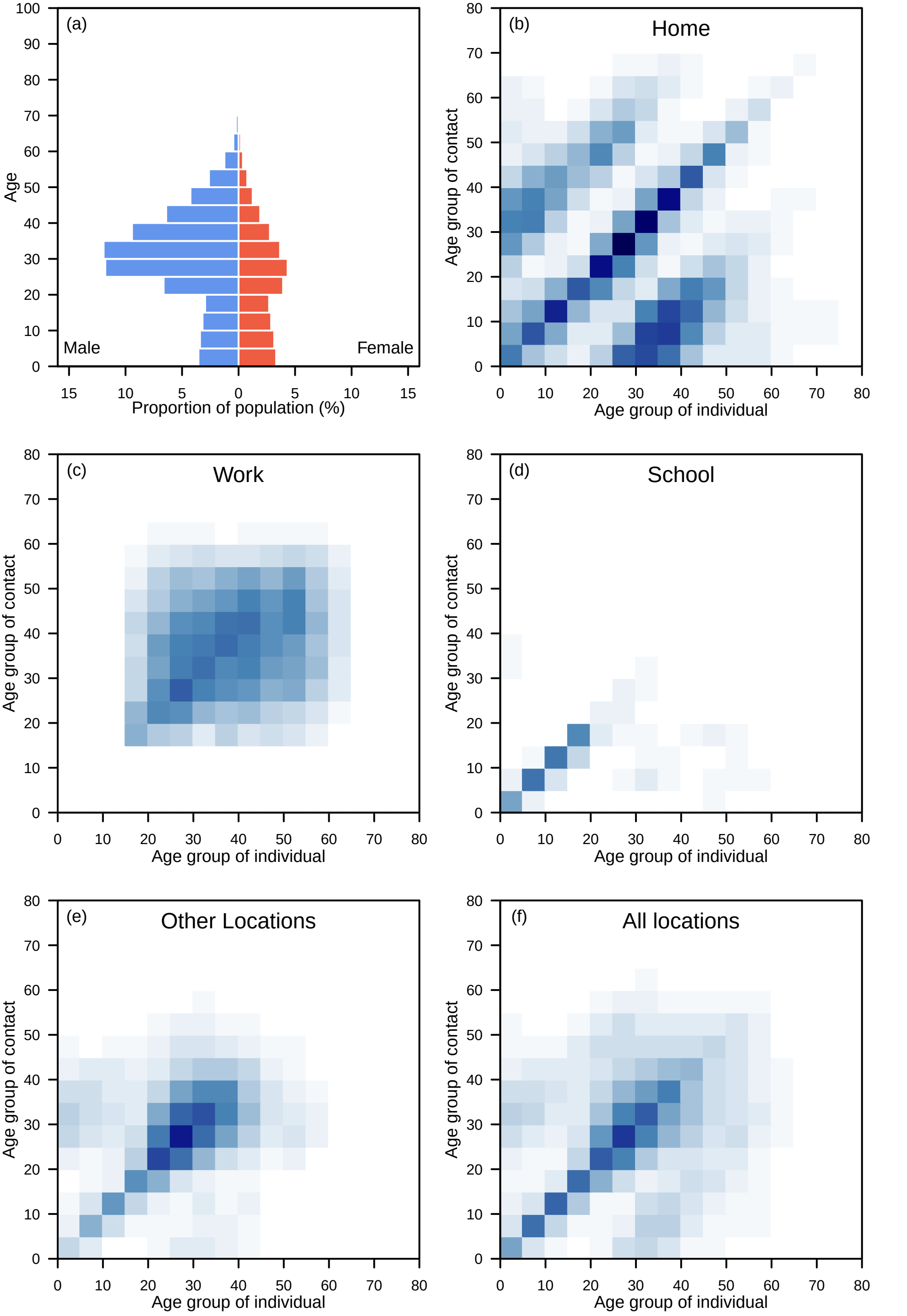

United Kingdom of Great Britain and Northern Ireland

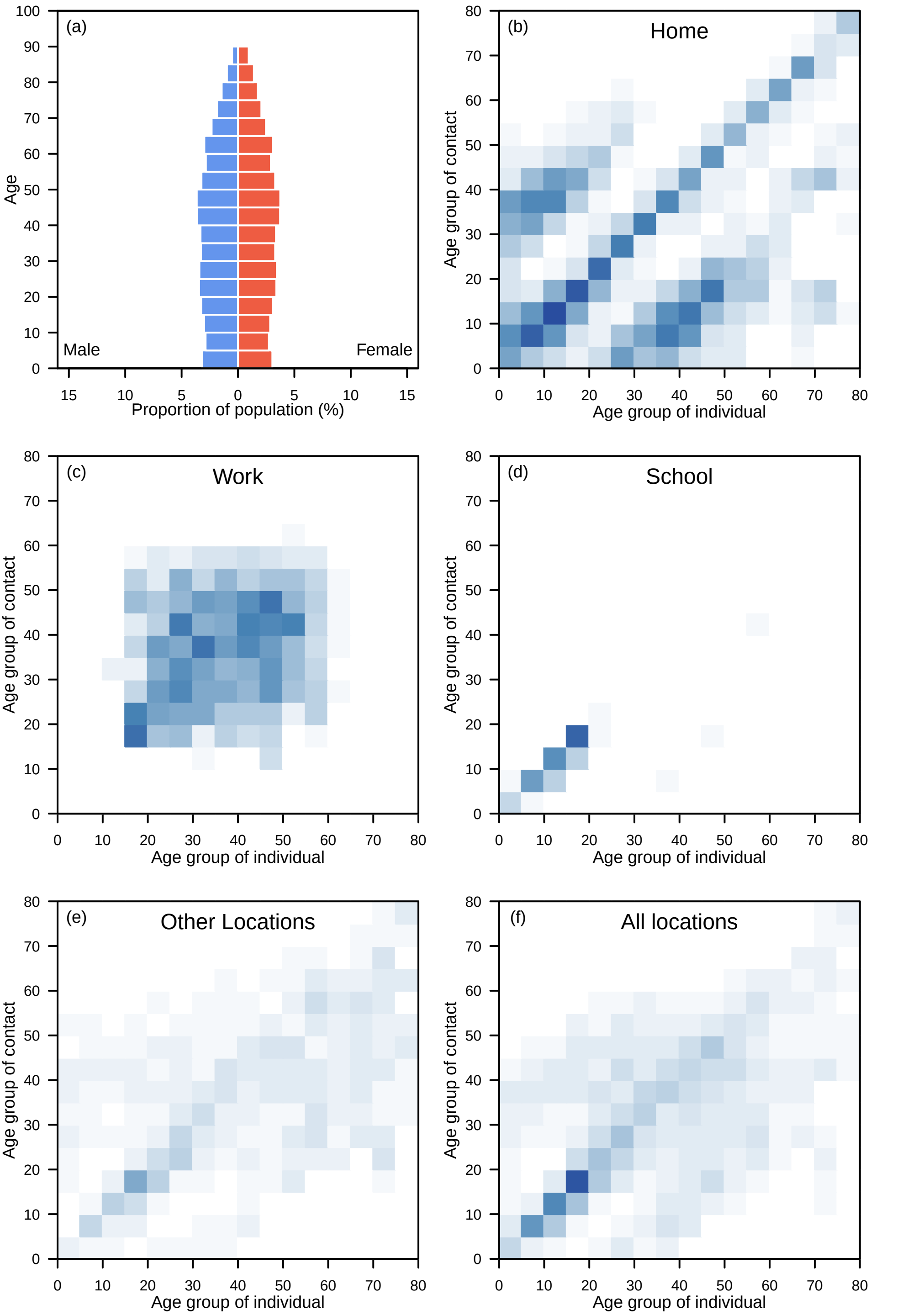

United Republic of Tanzania

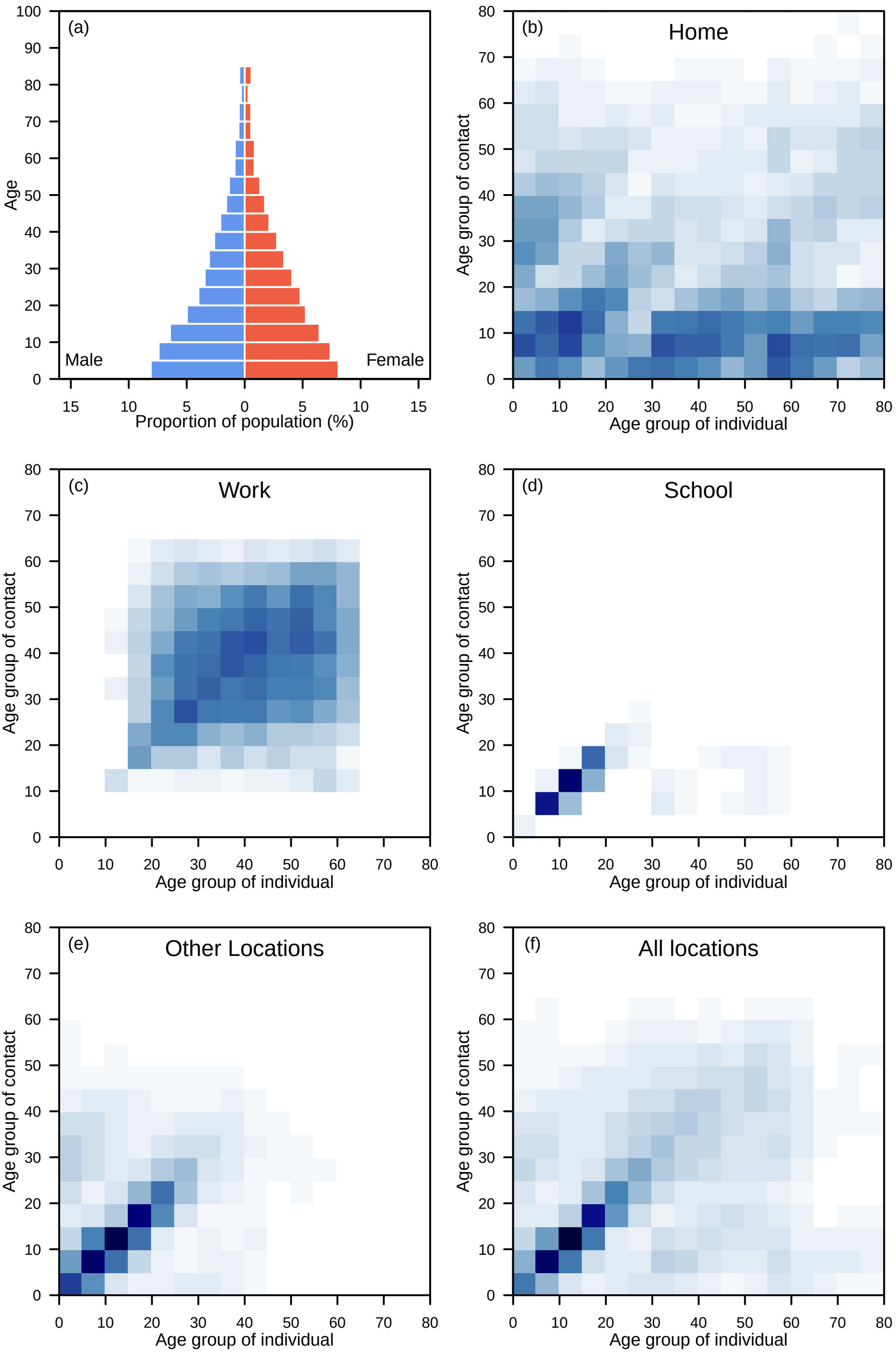

United States of America

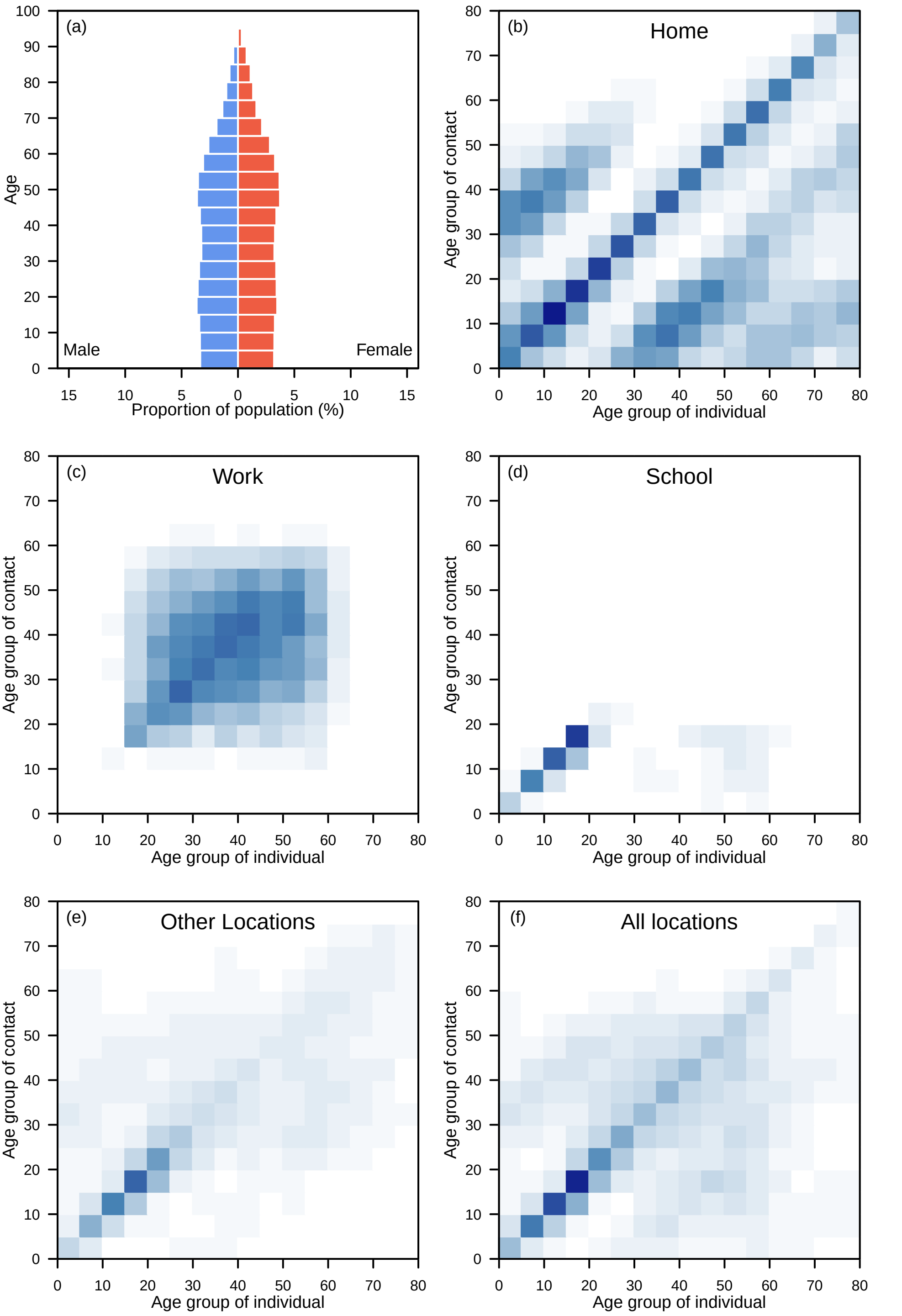

Uruguay

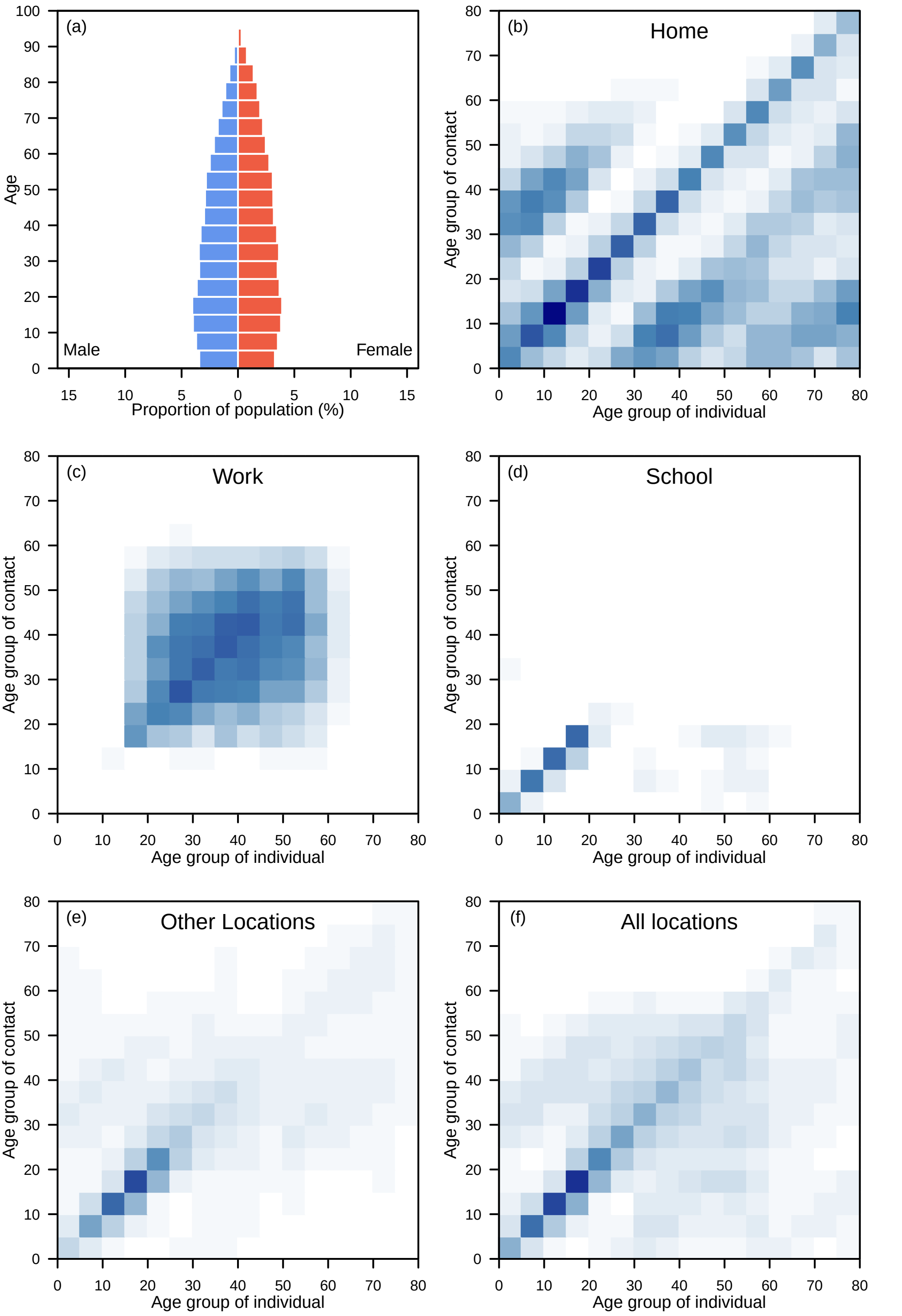

(a)
Age
Male
Female
Proportion of population (%)
(b)
Home
Age group of contact
Age group of individual
(c)
Work
Age group of contact
Age group of individual
(d)
School
Age group of contact
Age group of individual
(e)
Other Locations
Age group of contact
Age group of individual
(f)
All locations
Age group of contact
Age group of individual

Uzbekistan

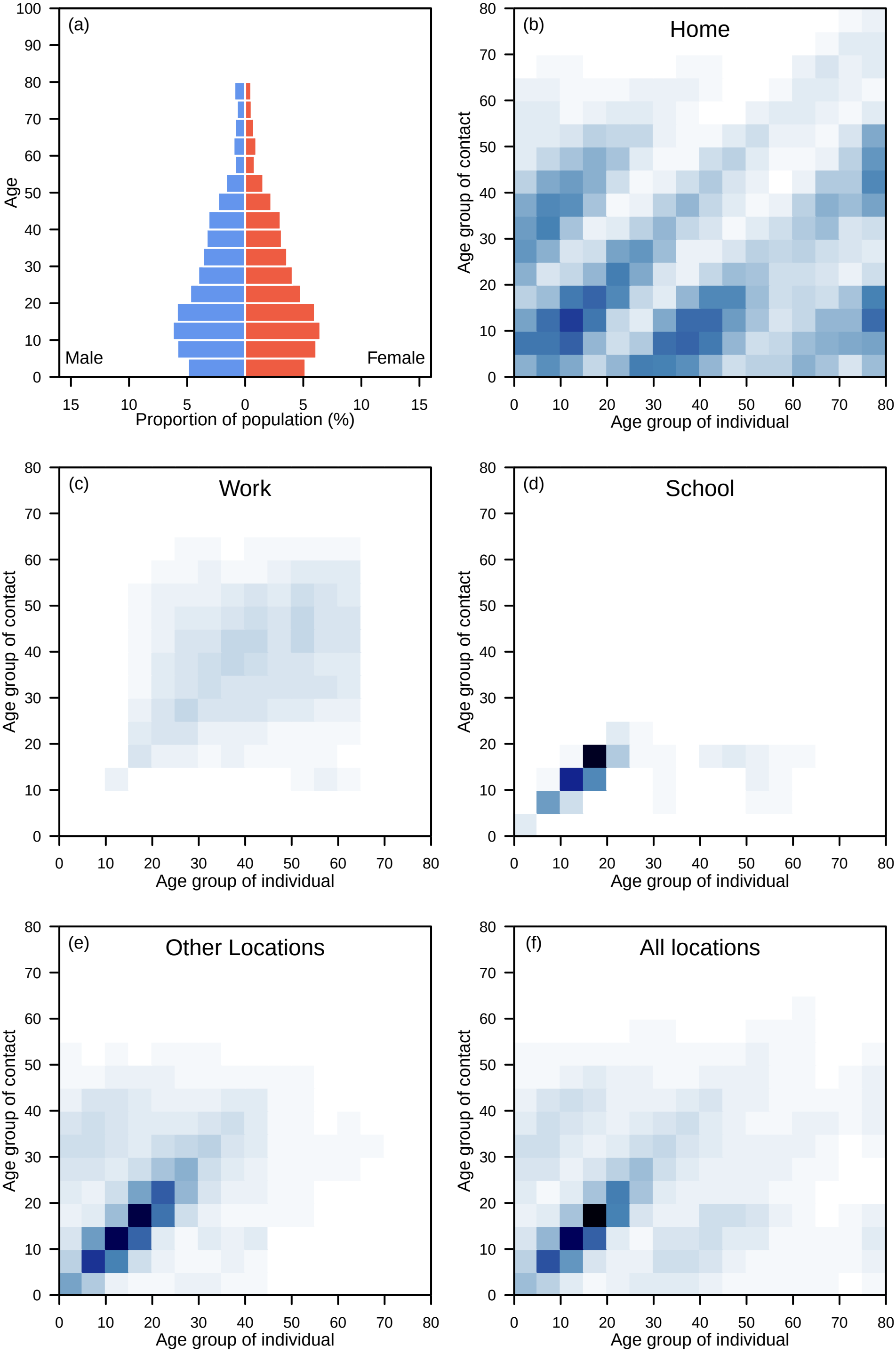

(a)
Male
Female
Age
Proportion of population (%)
(b)
Home
(c)
Work
(d)
School
(e)
Other Locations
(f)
All locations
Age group of contact
Age group of individual

Vanuatu

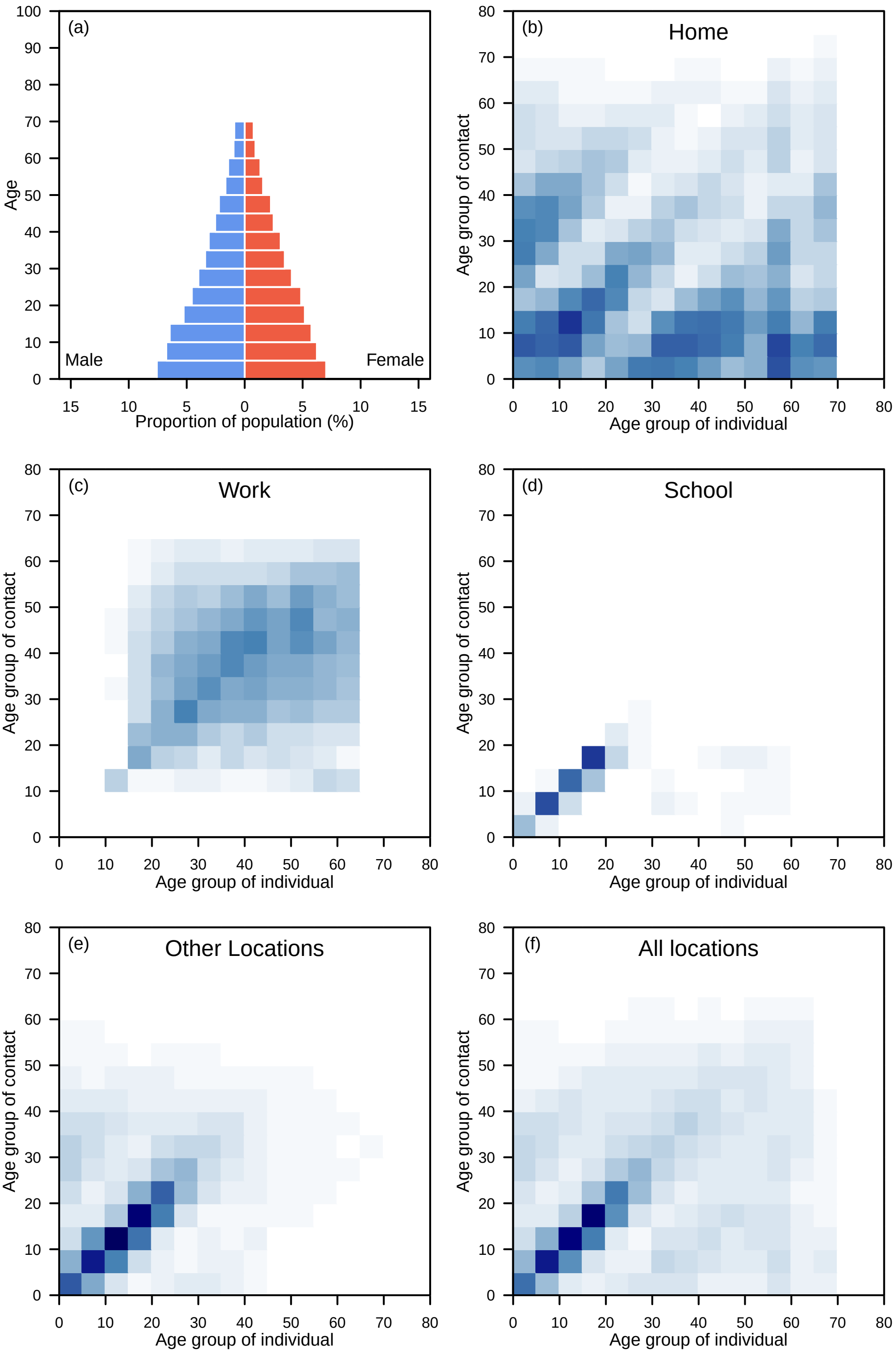

(a)
Age
Male
Female
Proportion of population (%)
(b)
Home
Age group of contact
Age group of individual
(c)
Work
(d)
School
(e)
Other Locations
(f)
All locations

Venezuela (Bolivarian Republic of)

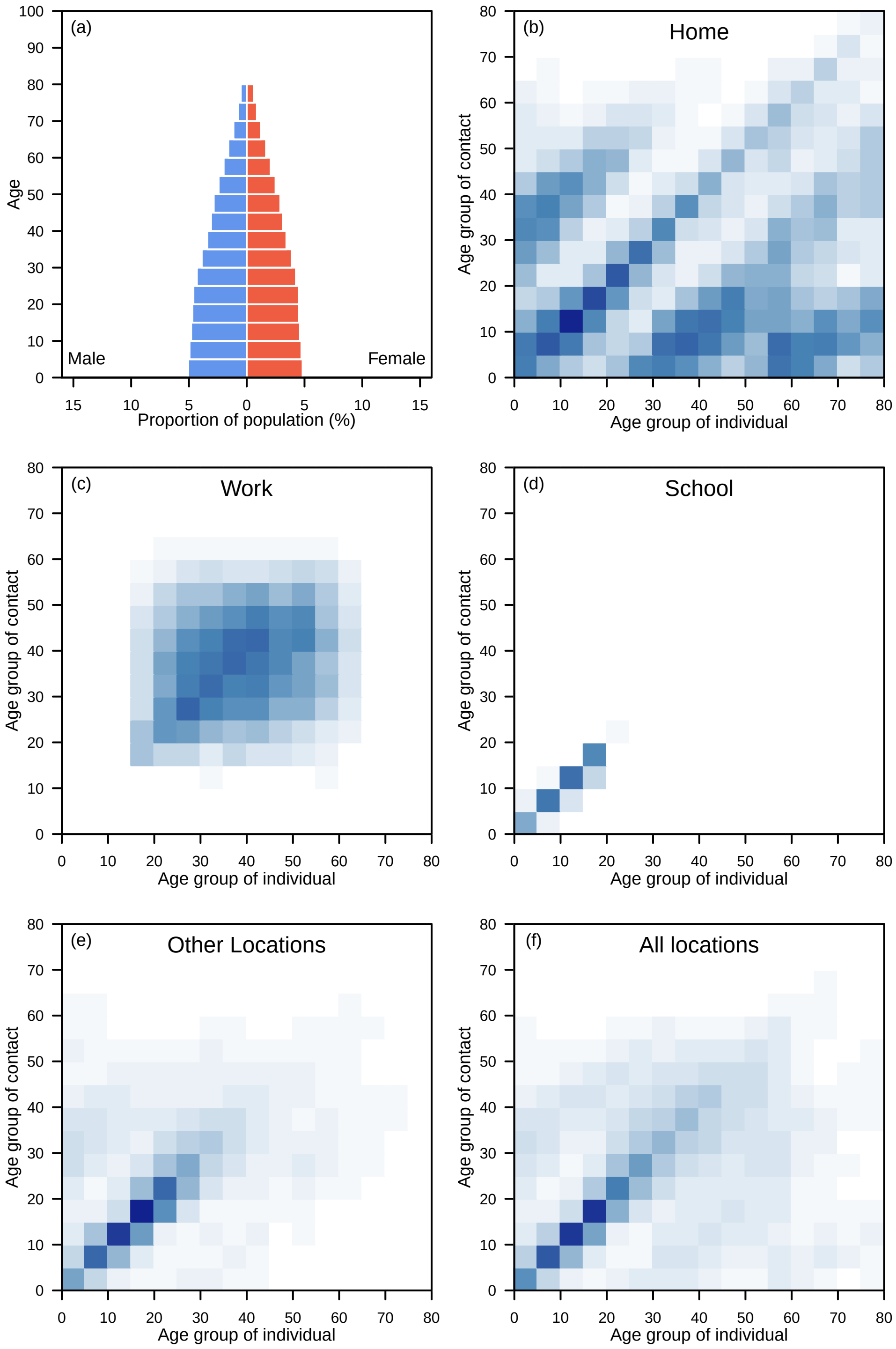

Viet Nam

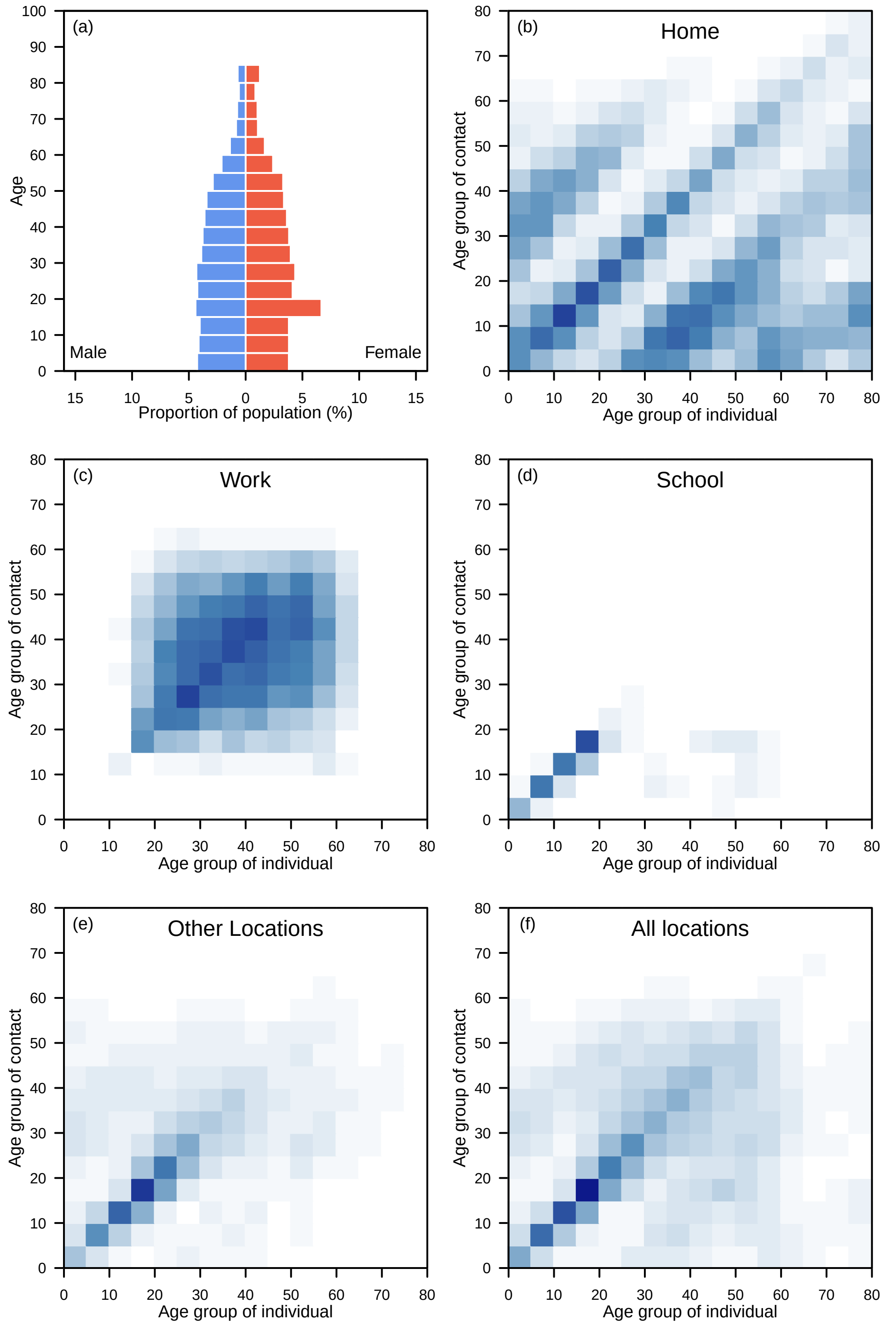

(a)
Age
Male
Female
Proportion of population (%)
(b)
Home
Age group of contact
Age group of individual
(c)
Work
Age group of contact
Age group of individual
(d)
School
Age group of contact
Age group of individual
(e)
Other Locations
Age group of contact
Age group of individual
(f)
All locations
Age group of contact
Age group of individual

Yemen

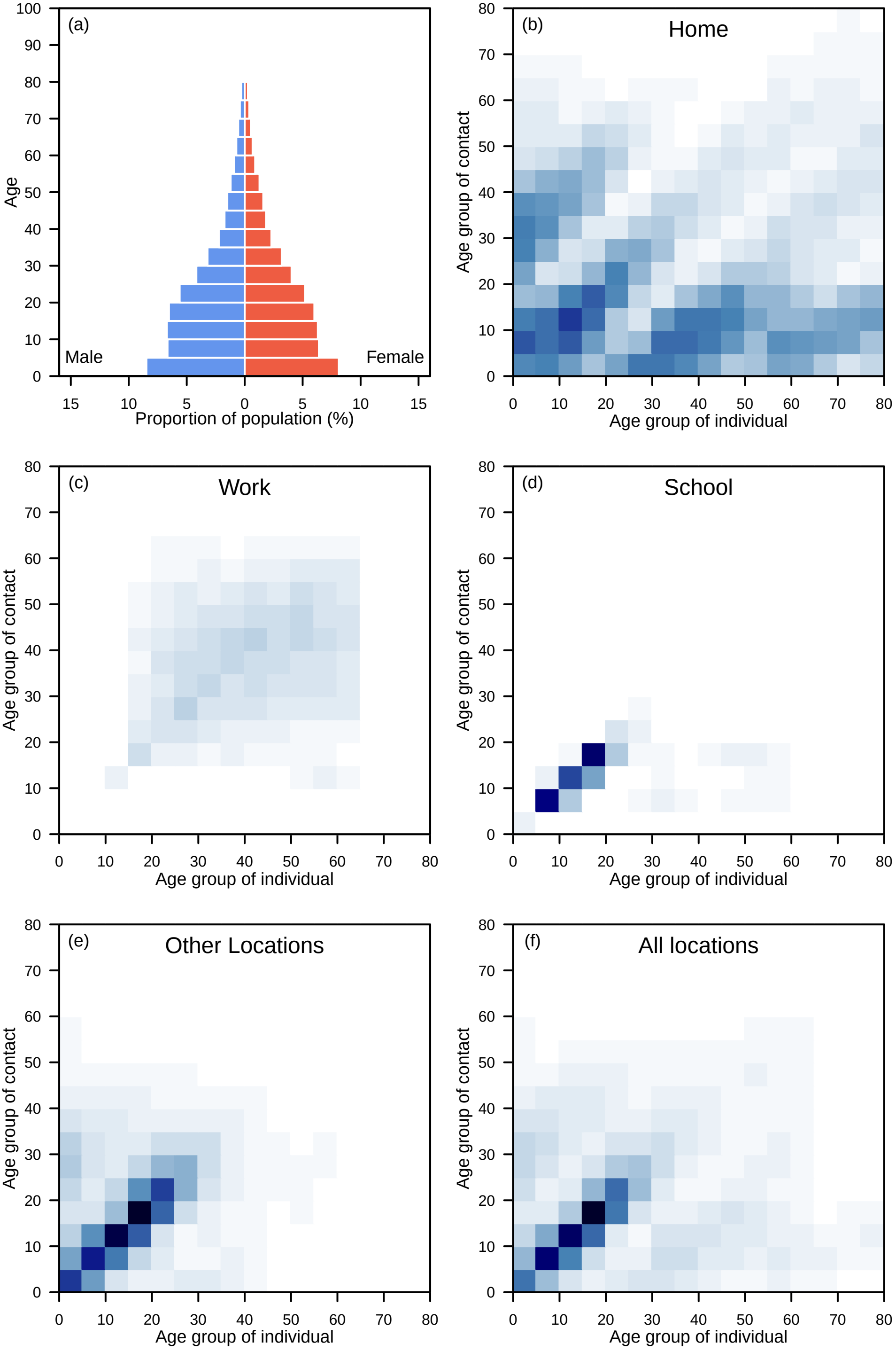

(a)
Age
Male
Female
Proportion of population (%)
(b) Home
(c) Work
(d) School
(e) Other Locations
(f) All locations
Age group of contact
Age group of individual

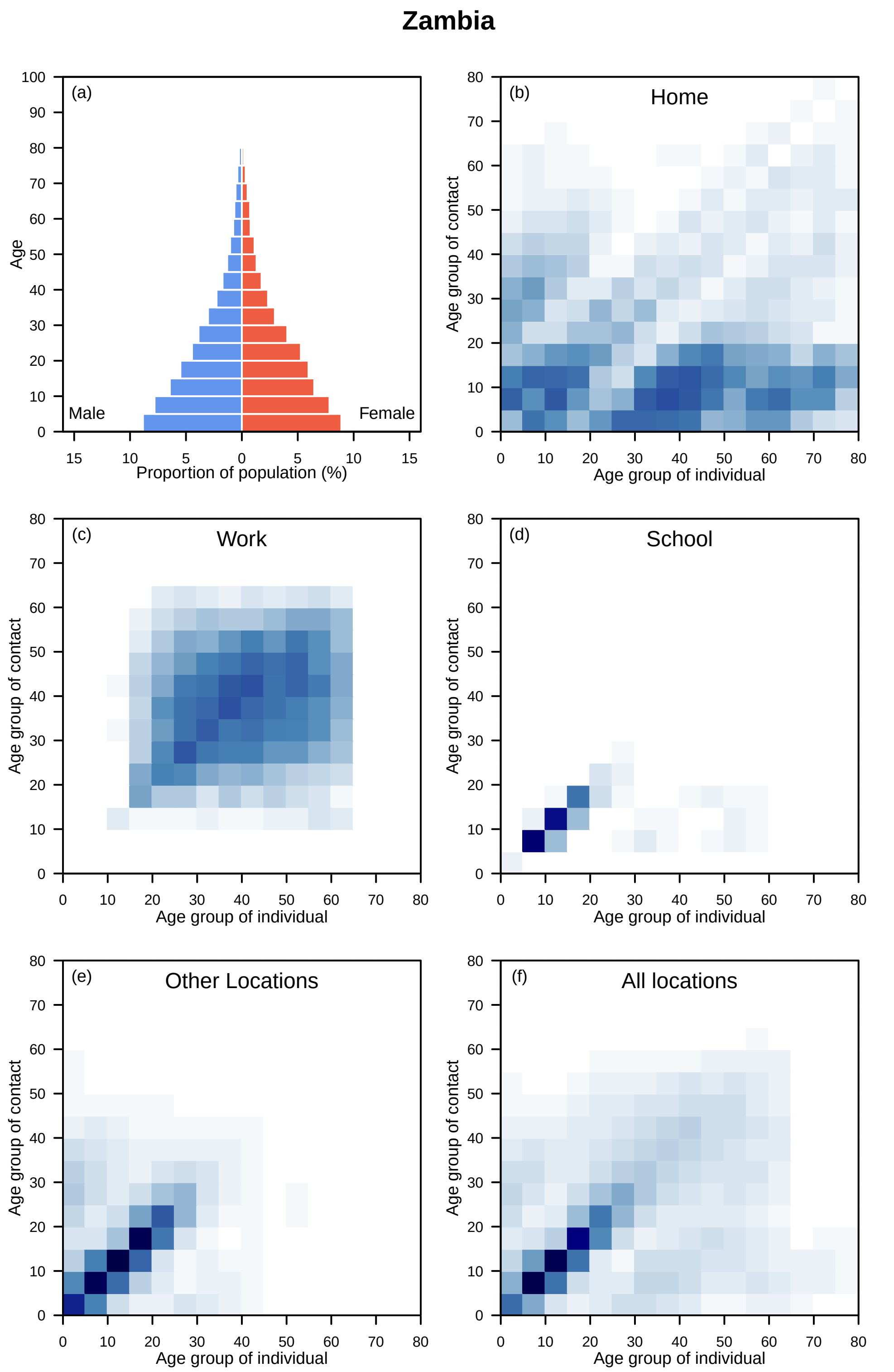

Zambia
(a)
Age
Male
Female
Proportion of population (%)
(b)
Home
Age group of contact
Age group of individual
(c)
Work
Age group of contact
Age group of individual
(d)
School
Age group of contact
Age group of individual
(e)
Other Locations
Age group of contact
Age group of individual
(f)
All locations
Age group of contact
Age group of individual

Zimbabwe

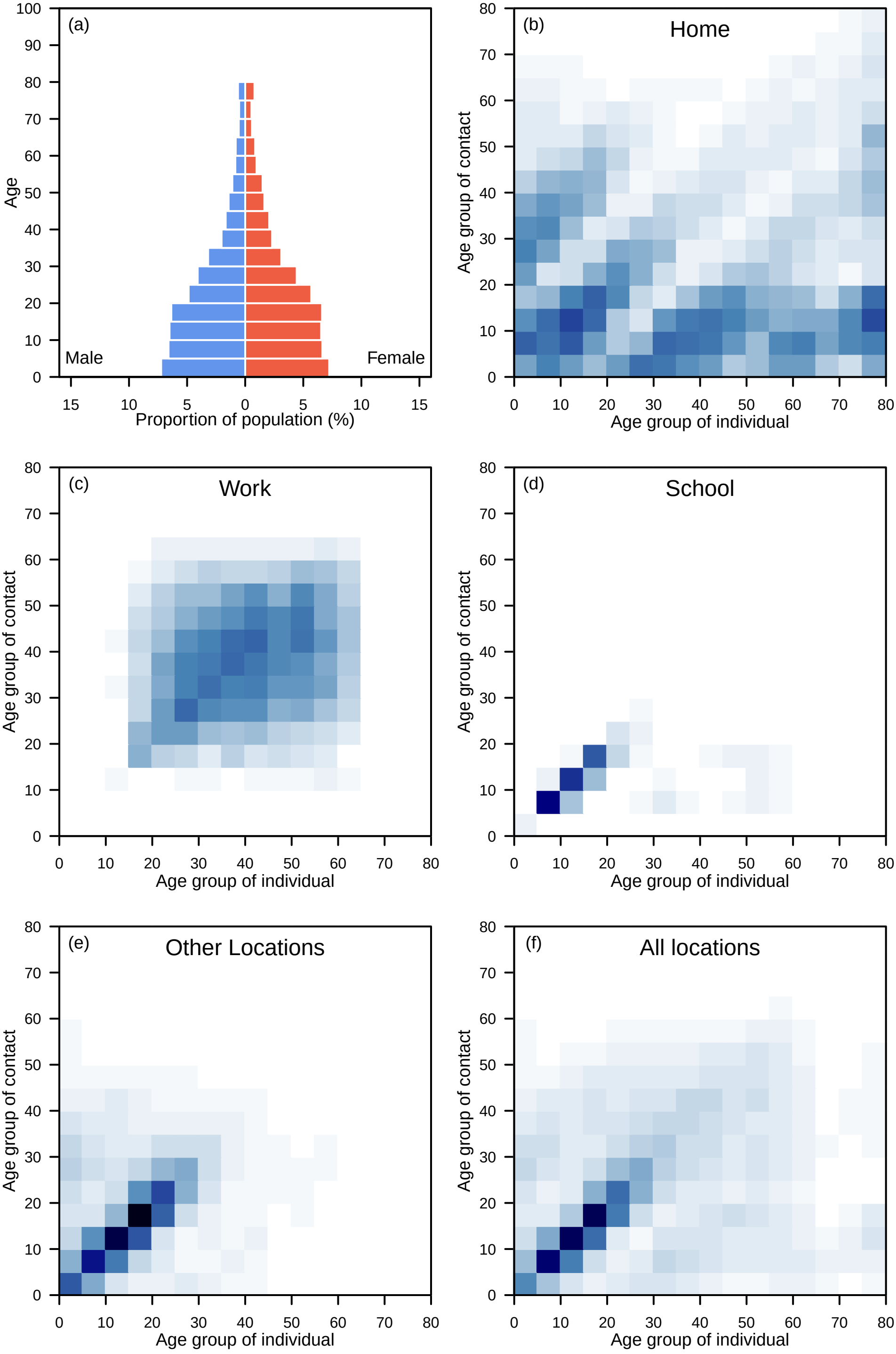

(a)
Age
Male
Female
Proportion of population (%)
(b)
Home
Age group of contact
Age group of individual
(c)
Work
Age group of contact
Age group of individual
(d)
School
Age group of contact
Age group of individual
(e)
Other Locations
Age group of contact
Age group of individual
(f)
All locations
Age group of contact
Age group of individual
